# Synthesis of new pyrazolo[3,4-*d*]pyrimidines as potential mutant EGFR/HER2 and Bcl2 inhibitors: anticancer evaluation, DFT, molecular docking and ADME studies

**DOI:** 10.1186/s13065-026-01773-6

**Published:** 2026-05-18

**Authors:** Nadia Hanafy Metwally, Zinab Atwa Saad, Mona Said Mohamed

**Affiliations:** Chemistry Department, Faculty of Science, Cairo University, Giza, 12613 Egypt

**Keywords:** Pyrazolo[3,4-*d*]pyrimidines, DFT, EGFR^T790M^/HER2 pro-inhibitors, Bcl-2 and ADME calculations

## Abstract

In this work, a novel 2-(6-methyl-4-oxo-4,5-dihydro-1*H*-pyrazolo[3,4-*d*]pyrimidin-3-yl)acetonitrile **4** was used as starting material to create a new series of pyrazolo[3,4-d]pyrimidines. DFT calculations and spectroscopic data (IR, ^1^H NMR, ^13^C NMR, and MS) were used to determine the structure of newly generated products. HepG2, MCF7, and HeLa were specific human cell lines used to evaluate these new compounds. When compared to erlotinib four specific compounds **6c**,** 8b**,** 10** and **13c** showed strong cytotoxic effects. When erlotinib was used as the reference drug, both **8b** and **10** exhibited notable effectiveness against EGFR^T790M^/ HER2. A study of proliferation of breast cancer cell line showed that **8b** increased in the percentage of DNA content in the G0-G1 phases, while **10** increased in the percentage of DNA content in the S phase. Furthermore, both **8b** and **10** decreased in Bcl2 levels in breast cancer cells. Additionally, both compounds had favourable interaction with the EGFR tyrosine kinase domain (TKD) (PDB ID: 5JEB), with binding free energies (ΔG_b_) close to -9.2 and − 7.5 kcal/mol, as revealed by molecular docking study. The molecular docking and ADME probe results were in accordance with the experimental data. In summary, the pyrazolo[3,4-*d*]pyrimidines **8b** and **10** displayed bioactivities on EGFR^T790M^/ HER2, suggesting their potential possible preclinical candidates for future preclinical studies.

**Graphical Abstract:**

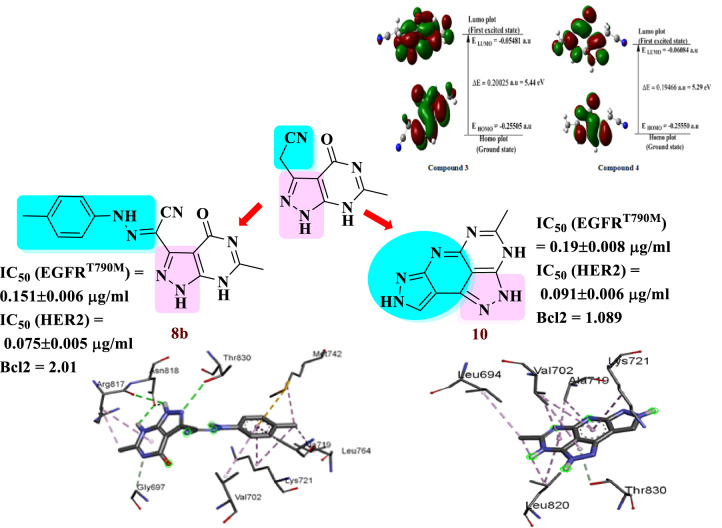

**Supplementary Information:**

The online version contains supplementary material available at 10.1186/s13065-026-01773-6.

## Introduction

**Fig. 1:**
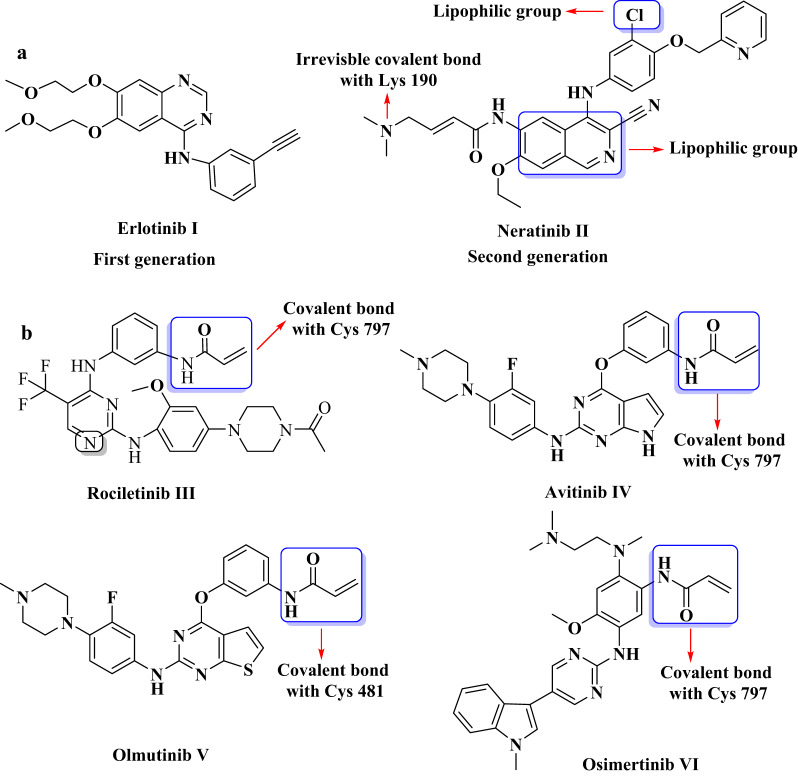
The chemical structures of **a** first- and second- generation EGFRs and **b** third generation of EGFRs

The World Health Organization forecasts that in 2022, there will be nearly 20 million new cases of cancer and around 97 million cancer-related deaths. By the year 2050, the number of new cases of cancer is expected to rise to approximately 35 million, representing a 77% increase from the initial estimate for 2022. Therefore, it is crucial to develop drugs that are not only more effective but also cause fewer side effects and unintended outcomes [1]. The epidermal growth factor receptor (EGFR) belongs to protein tyrosine kinase (PTKs) family, which consists of four receptor tyrosine kinases, ranging from HER1 to HER4 [2]. It also plays a crucial role in regulating tissue growth and maintaining cellular homeostasis [3]. Numerous cancer types, including hepatocellular carcinoma (HCC), colon, ovarian, breast, and non-small cell lung cancer (NSCLC) are associated with elevated EGFR overexpression [4, 5]. This character makes EGFR an attractive target for the development of new anticancer drugs [6–8]. The first-generation erlotinib (**I**), is among EGFR-tyrosine kinase inhibitors (EGFR-TKIs) approved by the FDA and has demonstrated activity against wild-type EGFR (EGFR^WT^) [9]. Erlotinib, works as an ATP analogue, competing with ATP binding sites inside RTKs to inhibit proliferation, cell cycle arrest, and apoptosis [10]. The EGFRT790M mutation prevents the first generation of TK inhibitors from binding ATP at higher concentrations by restoring ATP affinity to levels similar to wild type EGFR [11]. Second-generation neratinib (**II**) can overcome the resistances caused by HER2/EGFR, it achieves this by forming a covalent bond with the cysteine residues Cys-773 and Cys-805 found in the ATP-binding domains of HER1, HER2, and HER4, receptors, thereby inhibiting their function. This is due to the fact that it has a lipophilic 2-pyridinylmethyl moiety at the 4-position of the aniline ring and a lipophilic chlorine atom at the 3-position, which increases its activity against EGFR/HER2. Both the 2-pyridinylmethyl group and the chlorine atom are lipophilic, meaning they work well with the fats and oils. The compound needs this property in order to across the cell membranes and reach its destination inside the cell. The 2-pyridinylmethyl group at position-4 most likely also affects the compound’s binding affinity for the target protein. Additionally, the chlorine atom at position-3 enhances its activity, potentially through improved interaction or fit with the EGFR’s active site [12, 13] (Fig. 1a). Third-generation rociletinib (**III**) is a potent 5-trifluoro-2,4-diaminopyrimidine-based molecule made of a reactive electrophilic warhead acrylamide moiety, that forms a covalent bond with cysteine residue 797 was designed to address the risk factors and resistance mechanisms found in the first and second generations. This irreversible covalent attachment is essential for its function as a third-generation EGFR inhibitor that targets the T790M resistance mutation. Additionally, the amino-pyrimidine forms a bond with the hinge residue methionine residue 793 *via* hydrogen bonding, while its 5-substituent interacts with the gatekeeper residue (methionine in T790M) through a strong hydrophobic interaction [14]. Avitinib (**IV**), is pyrrolopyrimidine, with an acrylamide group, that functions as an electrophile, creates a strong and long-lasting covalent bond through a *Michael* addition reaction with the thiol group of Cys797 in the ATP-binding pocket [15]. Further, olmutinib (**V**), is thieno[3,2-*d*] pyrimidine core, with terminal acrylamide acting as *Michael* acceptor, covalently binds with Cys 81 in the Bruton`s tyrosine kinase (BTK) hinge segment and to Cys 797 in EGFR, inhibiting both BTK and EGFR [16, 17]. Additionally, osimertinib (**VI**) showed improved efficacy against mutant EGFR (EGFR^T790M^). The formation of an irreversible covalent bond to the thiol of cysteine797 residue in the EGFR-ATP-binding site is the mechanism of action.

This establishes a lasting bond that irreversibly inhibits the kinase function of the receptor. The pyrimidine core also in the osimertinib, establishes an essential hydrogen bond with the amide group in the backbone of methionine-793, found in the hinge area of the kinase domain. This interaction aids in effectively positioning the osimertinib structure for the covalent bond to cysteine797 [18, 19] (Fig. 1b).

**Fig. 2:**
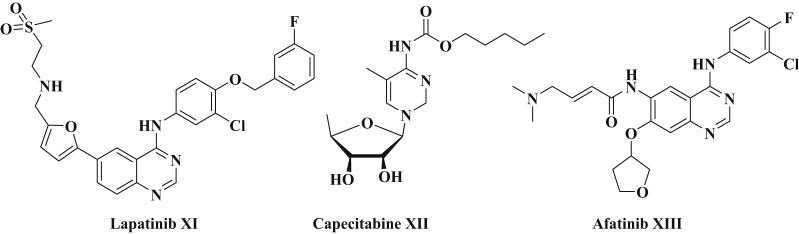
The chemical structures of approved HER2 and EDFR/HER2 drugs

The FDA also approved the combination of lapatinib (**XI**) and capecitabine (**XII**) for patients with HER2-positive metastatic breast cancer [20]. Breast, ovarian, prostate, colon and lung cancers have all been found to overexpressed EGFR/HER2. For example, the FDA has approval some dual EGFR/HER2 TK inhibitors, like lapatinib for the treatment of reast cancer as shown in Fig. 2. Afatinib **(XIII)** is an irreversible dual inhibitor of EGFR/HER2 has higher inhibitory activity than lapatinib [21] as demonstrated in Fig. 2.

B-cell lymphoma 2 (Bcl-2), a critical regulator of apoptosis, plays an essential role in ensuring cell survival by preventing programmed cell death. The human Bcl2 protein family comprises approximately 20 members, some functioning to promote apoptosis while others serve inhibit it. Over the past two decades, research has highlighted various mechanisms that contribute to progression of breast cancer, with the role of Bcl2 emerging as particularly significant. Bcl2, known as an inhibitor of apoptosis, has been strongly associated with highly invasive tumors that exhibit resistance to hormone therapy, being present in over 50% of breast cancer cases [22, 23].

**Fig. 3:**
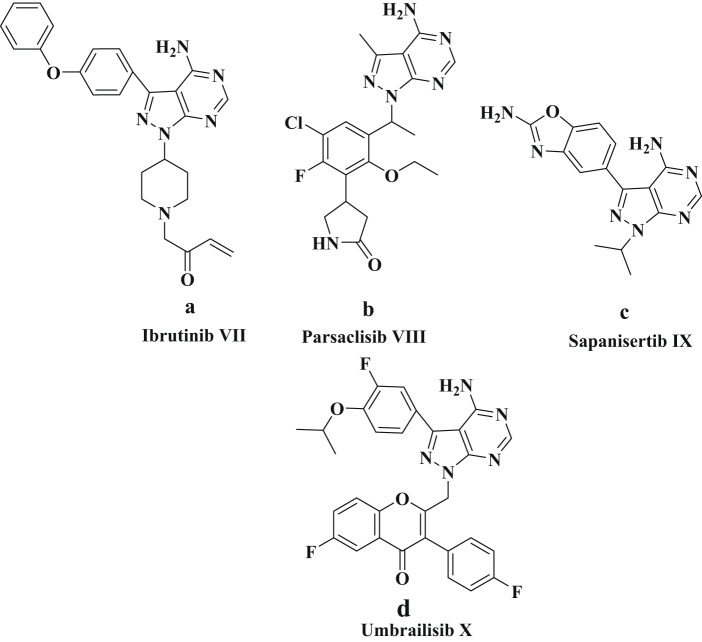
The structures of **a** approved drug and **b**, **c**, **d** clinical drugs containing pyrazolo-[3,4-d] pyramidine core

Nitrogen-containing heterocycles play a pivotal role in drug discovery, particularly in the context of market analyses of compounds like pyridine, pyrimidines and fused pyrimidine structures. They participate in a wide range of biological processes essential for life [24–26]. Moreover, pyrazole is an extensively researched structural component of pharmaceuticals that are commercially available. These compounds have demonstrated a diverse range of biological activities in clinical trials, both as standalone entities and in combination with their heterocyclic derivatives [27]. One of the critical pharmaceutical drugs in fused *N*-heterocycle chemistry is the pyrazolo-pyrimidine core, due to its structural resemblance to purines and guanine, which are integral components of DNA and RNA [28–31]. A notable example is ibrutinib, a drug used in the treatment of mantle-cell lymphoma and various forms of blood cancer, specifically chronic lymphocytic leukemia (**VII**) [32]. Moreover, three other drugs featuring this core are currently under clinical trials. The first, parsaclisib (**VIII**), is in phase II clinical trials and targets proliferative signals mediated by phosphoinoside 3-kinase (PI3K)δ in malignant B cells [33]. The second, sapanisertib (**IX**), has recently been developed as a highly selective dual inhibitor of mammalian target of rapamycin complexes 1 and 2 (mTORC1 and mTORC2), proteins responsible for regulating various cellular processes, including cell division and survival, by interacting with rapamycin and related drugs [34]. Lastly, umbralisib **(X)**, which is believed to target both PI3Kδ and casein kinase and has shown utility in treating relapsed or refractory marginal zone lymphoma (MZL) in adult patients [35] (Fig. 3).

**Fig. 4:**
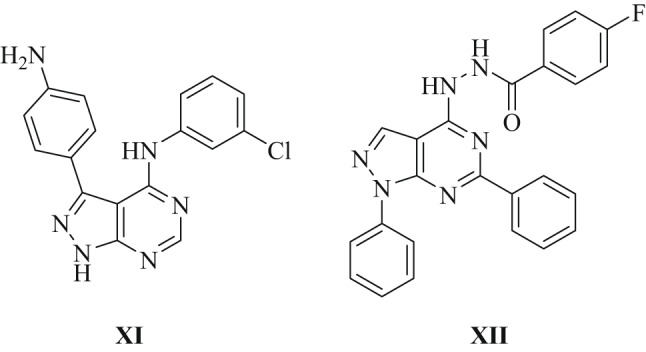
The chemical structures of approved EGFRs

Some EGFR-targeting fused pyrimidines including trizolo-pyrimidenes, and pyrido-pyrimidines, have been the subject of extensive study [36–38]. Additionally, synthesized pyrazolo[3,4-*d*] pyrimidines also demonstrate EGFR inhibitory activity, and a several derivatives have been created and examined for their potential application in cancer treatment [39–41]. As example 6-[2-(3-(4-Chlorophenyl)-5-(4-methoxybenzylidene)-6- oxo-5,6-dihydro-1,2,4-triazine-2(1 H)-yl)-2-oxoethyl]-3-methyl-1-phenyl-1 H-pyrazolo[3,4-*d*]pyrimi-din-4(5*H*)-one (**XI**) has been identified as a potent EGFR inhibitor, demonstrating an inhibitory efficacy of 81.81% [42]. Also, 4–(2-(1–(4-chlorophenyl)ethylidene)hydrazinyl)-*N*,1-diphenyl-1* H*-pyrazolo[3,4-*d*]pyrimidin-6-amine (**XII**) has exhibited inhibitory activity against both EGFR^WT^ and EGFR^790M^ [43] as illustrated in Fig. 4.

**Fig. 5:**
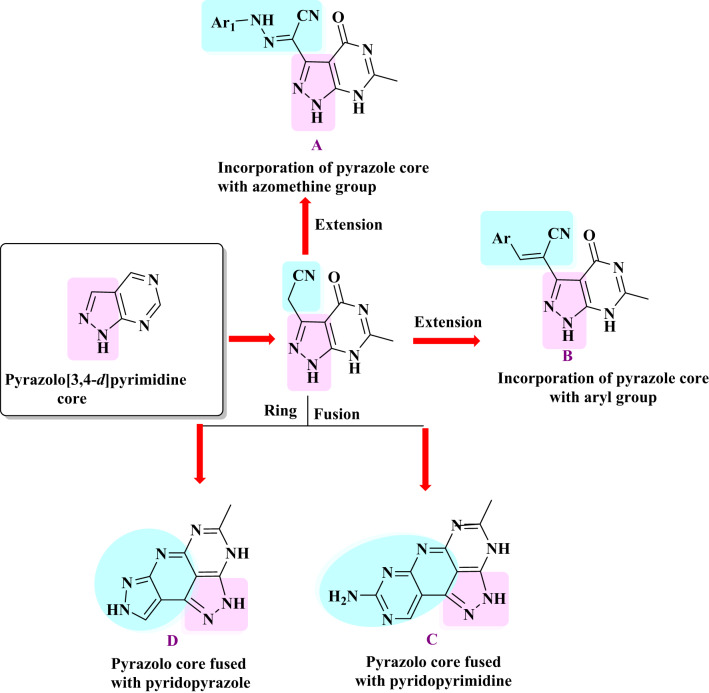
Map for the synthesis of target compounds in the current study

The finding of this study facilitated the synthesis of some heterocycles with diverse biological activities [44–55]. Among the new compounds developed were pyrazolo[3,4-*d*]pyrimidines (A and B), pyrazolopyridopyrimidine (C), and pyrazolopyridiopyrazole (D). These compounds were assessed against three different cancer cell lines, and the most cytotoxic compounds were determined against EGFR^T790M^/ HER2 and Bcl2. For the most potent compounds **8b** and **10**, the structure-activity relationship was investigated together with cell cycle apoptosis analysis. The results indicated that compounds **8b** and **10** hold potential as pre-inhibitors for breast cancer treatment by effectively inhibiting cell proliferation and inducting apoptosis. Their activity was found to be comparable to erlotinib, a reversible EGFR/HER2 inhibitor approved by the FDA for breast cancer treatment [9], as illustrated in Fig. 5.

## Results and discussion

### Chemistry

**Scheme 1:**
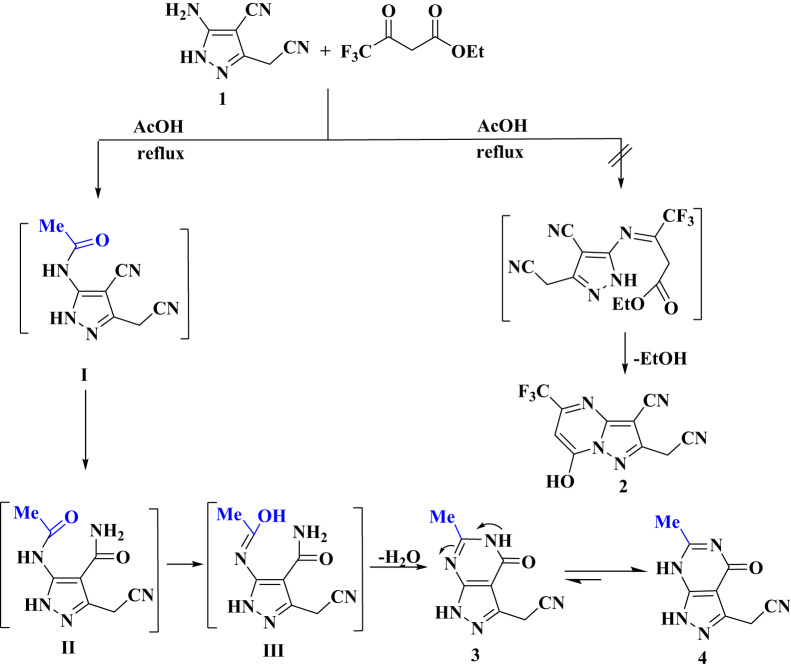
Schematic diagram of synthetic pathway of 2-(6-methyl-4-oxo-4,5-dihydro-1*H-*pyrazolo-[3,4-*d*] pyramidin-3-yl) acetonitrile** 4**

**Fig. 6:**
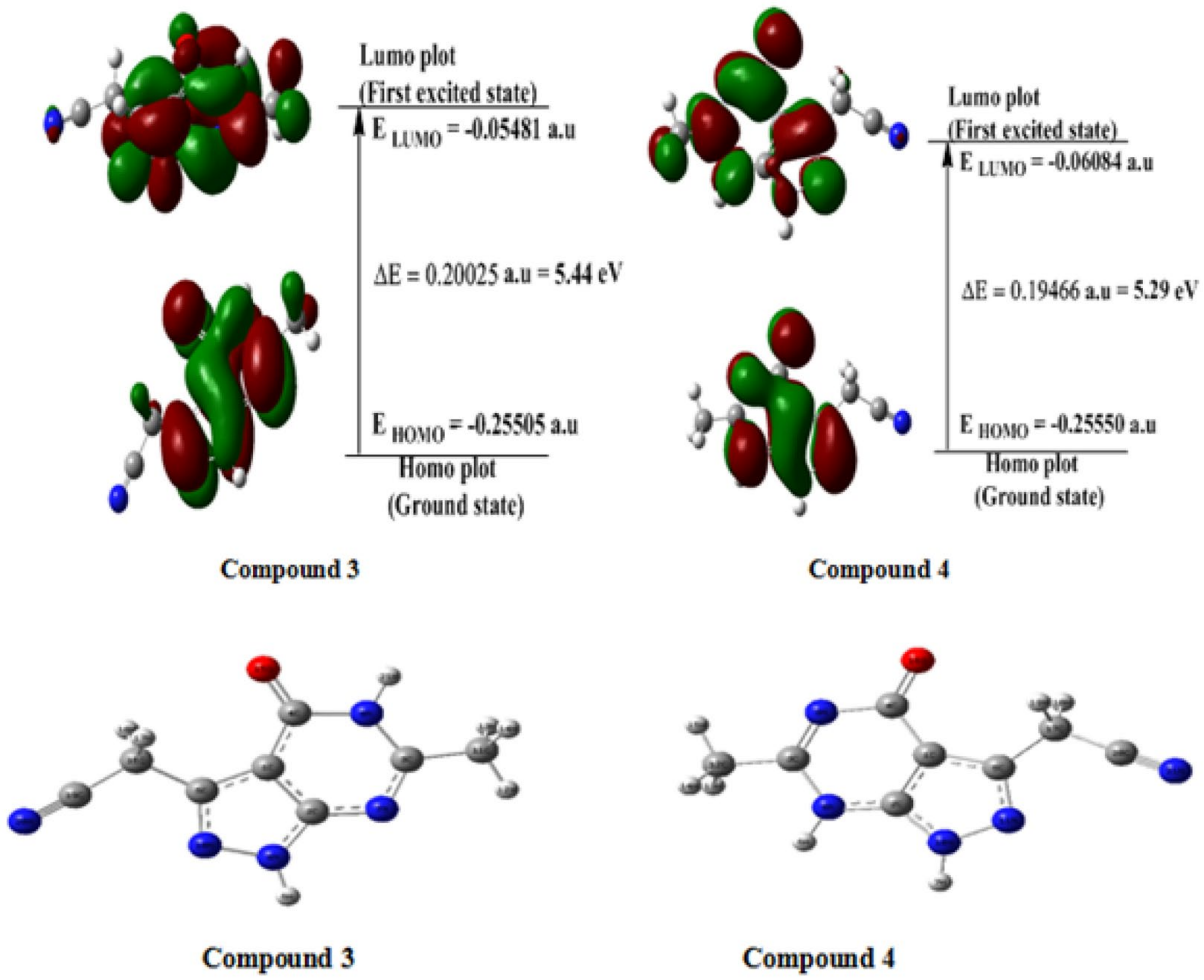
Optimized structures of **3** and **4** empolying B3LYP/6-311++G (d, p) level of calculation

The compound 5-amino-3-(cyanomethyl)-1*H*-pyrazole-4-carbonitrile (**1**) underwent a reaction with trifluoroethyl acetoacetate under reflux in glacial acetic acid, leading to the formation of **4**, instead of the expected product **2** as shown in Scheme 1. A detailed of spectral data confirmed that the structure of **4** corresponds to a pyrazolo[3,4-*d*]pyrimidine derivative as shown in Scheme 1. The IR spectrum of **4** showed absorption bands at υ_max_ = 3290 and 3250 cm^− 1^ for NH groups, as well as the cyano and carbonyl groups at 2232 and 1693 cm^− 1^, respectively. The ^1^H NMR spectrum of **4**, revealed a singlet signal for CH_2_ protons at 4.14 ppm, along with two D_2_O exchangeable signals at 11.21 and 13.41 ppm. A singlet signal at 2.11 ppm for methyl proton was also observed. ^13^C NMR spectrum for **4** displayed a characteristic signal at 16.26, 22.80, 78.36, 112.56, 116.71, 143.35, 143.83 and 169.04. Mas spectrum of **4** exhibited a molecular ion peak at 189.35 (M^+^, 100%), confirming its molecular weight. The synthetic pathway for compound **4** involves an initial acetylating of compound **1** to form the acetyl derivative **I**, which subsequently undergoes acid hydrolysis to form intermediate **II**. Enolization of **II** generates the enol form **III**, which undergoes dehydrative cyclization to give amide intermediate **3** isomerizes into the keto-form **4**. Density functional theory calculations (DFT) [56] were employed to elucidate the thermodynamic preference for the reaction product. The calculations revealed that compound **4** is energetically more stable than compound **3**. The electronic properties of compounds **3** and 4, namely Homo and Lumo were analyzed. The respective energy gaps for compounds **3** and **4** were calculated to be 5.44 eV and 5.29 eV, respectively, indicating a slightly greater stability for compound **4**. The optimized molecular structures corresponding to the lowest energy conformations for compounds **3** and **4** are depicted in Fig. 6.

**Scheme 2:**
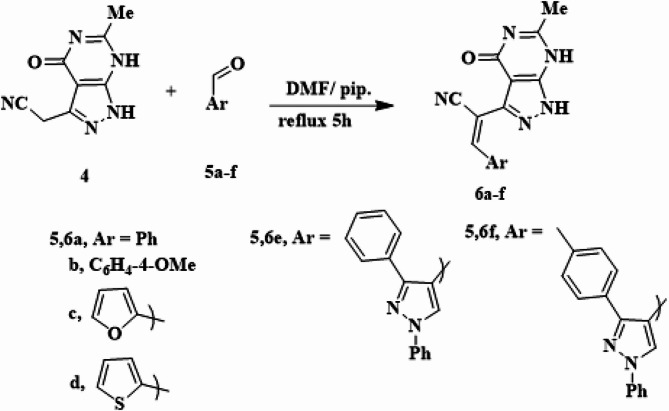
Synthesis of compounds **6a-f** by the reaction of 4 with different aldehydes **5a-f**

Compound **4** was condensed with different aromatic aldehydes **5a-f** in *N*,* N*-dimethylformamide (DMF) with a few drops of piperidine, leading to the formation of arylmethylene derivatives **6a-f** as shown in Scheme 2. The structural elucidation of compounds **6a-f** was approved using spectral data. As example, the IR spectrum of **6b**, selected as a representative from the prepared series **6a-f**, exhibited absorption peaks at 3227, 3118, 2233 and 1697 cm^− 1^, corresponding to the 2NH, nitrile and carbonyl functional groups, respectively. The ^1^H-NMR spectrum was characterized by the appearance of a new signal corresponding to the methoxy proton at 3.84 ppm. Additionally, signals attributed to aryl protons appeared at chemical shifts of 7.11, and 7.90 ppm, respectively. Furthermore, two sets of exchangeable D_2_O signals were observed at 11.22 and 13.79 ppm.

**Scheme 3:**
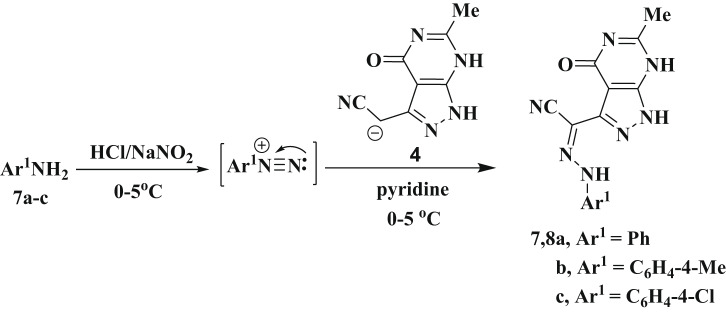
Synthetic route for the preparation of arylazo derivatives **8a-c**

Furthermore, the coupling of **4** with aryl diazonium salts **7a-c**, which prepared by diazotizing primary aromatic amine using sodium nitrite at temperature between 0 and 5 °C, in pyridine at 0–5 °C, yielded the arylhydrazono derivatives **8a-c** (Scheme 3). The structures of **8a-c** were verified through spectral data. Compound **8b** showed characteristic peaks in its IR spectrum corresponding to three NH, one nitrile, and one carbonyl groups at 3388, 3246, 3126, 2227, and 1694 cm^− 1^, respectively. The ^1^H NMR chart displayed two singlet signals for the methyl protons at 2.11 and 2.25 ppm. Additionally, three amino signals exchangeable with D_2_O were observed at 11.15, 11.44 and 13.42 ppm, respectively. Signals for the aryl protons were detected at 7.11 and 7.41 ppm.

**Scheme 4:**
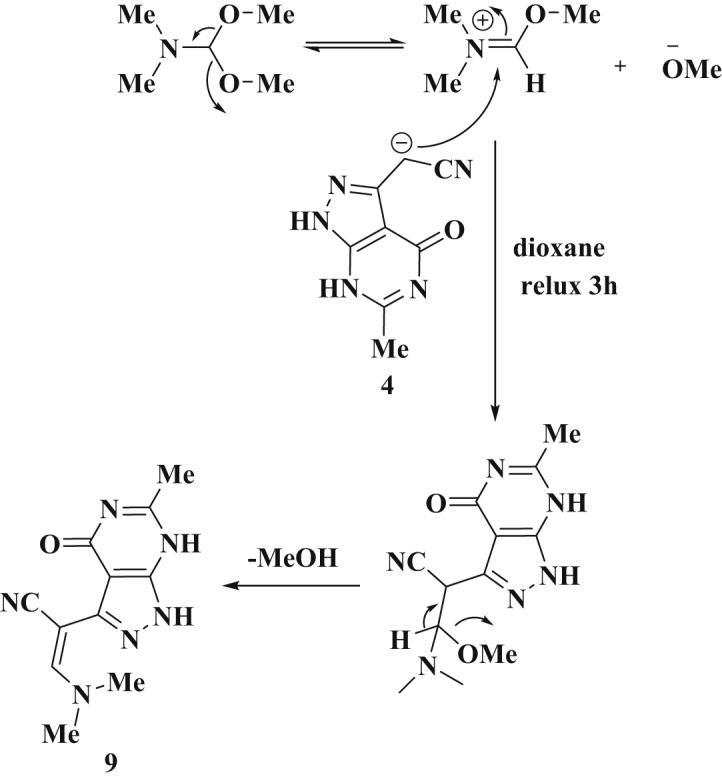
The proposed formation of the enamine derivative **9** from compounds **4**

Compound **4** underwent a reaction with *N*,* N*-dimethylformamide-dimethylacetal (DMF/DMA) in dioxane under reflux conditions for 3 h afforded the enamine derivative **9** as depicted in Scheme 4. The structure of the enamine **9** was proved by IR, ^1^H NMR, ^13^C NMR and MS. IR spectrum, which identified peaks for two NH, nitrile, and carbonyl functions at wavelengths 3228, 3125, 2231 and 1693 cm^− 1^, respectively. A singlet signal at 7.39 ppm corresponds to the -CH proton, and is situated adjacent to two singlet signals from the 2NH protons at 10.45 and 13.20 ppm were observed in its ^1^H NMR spectrum. Further, three singlet signals were appeared at chemical shift at 2.04, 3.20 and 3.32 ppm for three methyl protons, respectively. It is proposed that compound **9** is generated by nucleophilic attack of the active methylene in compound **4** (containing acidic proton), on the electrophilic central carbon of DMF-DMA. Accordingly, the condensation reaction occurs to produce enamine derivatives by the eliminating two methanol molecules, as illustrated in Scheme 4 [57, 58].

**Scheme 5:**
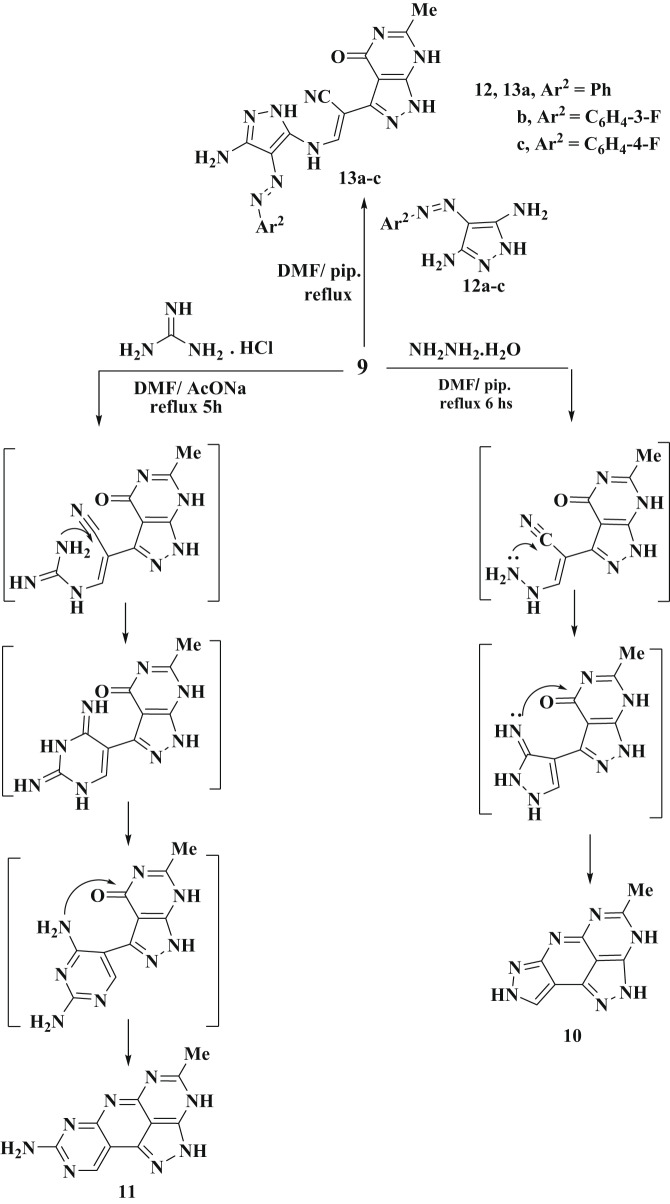
The suggested formation of compounds **10**,** 11** and **13a-c** from enamine **9**

The enamine derivative **9** reacts with hydrazine hydrate in a DMF with a few drops of piperidine resulting in the formation of the pyrazole derivative **10**, as illustrated in Scheme 5. The structure of **10** was validated by ^1^H NMR spectrum, which displayed a new singlet signal at δ = 7.83 ppm for the pyrazole proton. IR showed a broad peak of three amino groups at 3397 cm^− 1^. The mass spectrum exhibits a molecular ion peak at m/z = 213.09 (M^+^), which corresponding to a molecular formula C_9_H_7_N_7_. The proposed mechanism of the formation of product **10** involves the initial addition of the hydrazine amino group to the enamine double bond, which was then followed by the elimination of dimethylamine and a water molecule, ultimately yielding the final product **10**. Enamine **9** undergoes a reaction with guanidine hydrochloride in DMF containing sodium acetate, yielding the compound **11**. The ^1^H NMR spectrum of this product revealed a resonance of H-pyrimidine at δ = 8.88 ppm, along with signals from methyl and amino protons in their expected region. Its IR chart displayed an absorption band at wavelength equal 3432 cm^− 1^ due to the amino and two NH functions. The proposed synthesis pathway for product **11** starts with the addition of an amino group from guanidine across the enamine double bond, leading to the elimination of dimethylamine. This is followed by nucleophilic addition involving the amino function to the nitrile group. The intermediate thus formed undergoes cyclization, resulting in the formation of aminopyrimidine derivative **11** (Scheme 5). We explored the reactivity of compound **9** with heterocyclic amines, by reacting it with arylazo-aminopyrazoles **12a-c** in DMF, using piperdine as a base, this process, conducted under reflux conditions, resulted in the formation of a single product in each instance, identified as **13a-c**, as depicted in Scheme 5. The structure of isolated products **13a-c** was determined through their spectroscopic data. These compounds exhibited absorption bands for NH and NH_2_ groups in the range of 3218–3419 cm^− 1^, and carbonyl groups between 1689 and 1694 cm^− 1^. Additionally, their ^1^H NMR spectra exhibited singlet signals for methyl protons within the range of 2.15–2.17 ppm, alongside the expected signals for amino and aryl protons.

### Biology activity

#### Anticancer evaluation

Compounds **4**,** 6a**,** 6b**, **6c**,** 6d**,** 6e**,** 6f**,** 8a**, **8b**,** 8c**,** 10**,** 11**,** 13b** and **13c** were subjected to the MTT assay, a colorimetric method, to assess their cytotoxic effects on three selected human cell lines: HepG2, MCF7, and HeLa (refer to Table 1). Erlotinib was utilized as the standard drug for comparison. The cytotoxic activity of compounds is expressed as an IC_50_ values (µM) concentration where IC_50_ indicates the concentration required to inhibit cell growth by 50%.

**Table 1:** Cytotoxic activity of compounds **4**,** 6a**,** 6b**, **6c**,** 6d**,** 6e**,** 6f**,** 8a**, **8b**,** 8c**,** 10**,** 11**,** 13b**, and **13c** against selected human cancer cell lines

Comp. No.	In vitro cytotoxicity IC_50_ (µM)			In vitro cytotoxicity IC_50_ (µM)			
	HepG2	MCF7	HeLa	WI38	SI1	SI2	SI3
**4**	33.45 ± 2.3	43.37 ± 2.7	38.67 ± 2.5				
**6a**	71.23 ± 4.1	68.39 ± 3.8	58.82 ± 3.3				
**6b**	50.91 ± 3.2	29.71 ± 2.1	45.28 ± 2.8				
**6c**	12.68 ± 1.1	16.26 ± 1.3	23.80 ± 1.8	47.92 ± 2.8	3.77	2.94	2.01
**6d**	51.84 ± 2.9	60.61 ± 3.5	84.81 ± 4.5				
**6e**	62.81 ± 3.5	56.45 ± 3.2	49.33 ± 3.0				
**6f**	25.75 ± 1.8	21.25 ± 1.9	44.24 ± 2.6				
**8a**	19.79 ± 1.6	35.62 ± 2.3	27.28 ± 2.2				
**8b**	10.38 ± 0.9	9.38 ± 0.7	13.89 ± 1.0	61.48 ± 3.6	5.92	6.55	4.42
**8c**	40.80 ± 2.7	28.89 ± 2.1	38.06 ± 2.5				
**10**	11.61 ± 0.9	17.70 ± 1.4	32.92 ± 2.2	46.41 ± 3.9	3.99	2.622	1.40
**11**	72.51 ± 3.8	78.37 ± 4.2	63.03 ± 3.7				
**13b**	37.31 ± 2.2	42.37 ± 2.5	56.59 ± 3.3				
**13c**	19.96 ± 1.4	7.87 ± 0.7	23.69 ± 1.9	59.78 ± 3.3	**2.99**	7.59	2.52
**Erlotinib**	11.31 ± 0.66	4.27 ± 0.26	8.27 ± 0.42				

Additionally, it highlights the effect of compounds **6c**, **8b**, **10**, and **13c** on the normal cell WI38

IC_50_ (µM) are the mean of three independent replicates ± SD.

The data in Table 1 reveals that the cytotoxic effects of the unsubstituted compound **4** were moderate across the three cell lines. Introducing a furan moiety into compound **6c**, significantly improved the IC_50_ values, registering 12.68 ± 1.1, 16.26 ± 1.3, and 23.80 ± 1.8 µM for the HepG2, MCF7, and HeLa cell lines, respectively, when compared to erlotinib (IC_50_ =11.31 ± 0.66, 4.27 ± 0.26 and 8.27 ± 0.42 µM). Additionally, compound **8b**, featuring a methyl group positioned at the 4-position of the phenyl core, exhibited the most potent cytotoxic activity in these cell lines compared to compound **8a** (the phenyl derivative) and compound **8c** (the 4-Cl derivative). Moreover, the compound **10**, in its cyclized form, demonstrated the highest cytotoxic activity in the HepG2 cell line, with activity comparable to that of erlotinib of (11.31 ± 0.66 µM). The 4-fluoro derivative **13c** demonstrated an IC_50_ value of 7.87 ± 0.7 µM, in the MCF7 cell line. Conversely, compounds **11** and **13b** (the 3-fluoro derivative) exhibited weak to moderate activity across all three cell lines.

### Effect of compounds 6c, 8b, 10, and 13c on normal cell

The cytotoxicity effects of compounds **6c**,** 8b**,** 10** and **13c** were further assessed against the human normal lung fibroblast cell line (WI38) in *vitro* (Table 1). These compounds displayed a favourable safety profile, with IC_50_ values recorded at 47.92 ± 2.8 µM, 61.48 ± 3.6, 46.41 ± 3.9, and 59.78 ± 3.3 µM, respectively.

### P-Value calculations

The p-Values were reported as 000 for the three human cell lines HePG2, MCF7 and HeLa, indicating a very highly level statical significance. For the normal cell lines WI38, the p-value was 001, which denotes a high level of significance. Additional details regarding these calculations are provided in the Supplementary Table file.

### EGFR^T790M^ and HER2 kinase inhibition assay

The cytotoxicity of compounds **6c**, **8b**, **10** and, **13c** was investigated through EGFR^T790M^ and HER2 kinase inhibition assays to elucidate their anticancer modes of action using erlotinib as a reference compound against EGFR^T790M^ and HER2 kinase. The results are shown in Table 2 and showed that compound **8b** and **10** have very active in this assay against EGFR^T790M^ and HER2 kinase and moderate activities for compound **6c** and **13c**.

**Table 2:** EGFR^T790M^/ HER2 inhibitory activity of compounds **6c**,** 8b**,** 10** and **13c**

Comp. no.	EGFR^T790M^ IC_50_ (µM)	HER2 IC_50_ (µM)
**6c**	1.845 ± 0.02	0.696 ± 0.011
**8b**	0.491 ± 0.006	0.243 ± 0.005
**10**	0.891 ± 0.008	0. 427 ± 0.006
**13c**	0.906 ± 0.015	0.487 ± 0.012
**Erlotinib**	0.124 ± 0.002	0.086 ± 0.002

### Cell cycle analysis

**Fig. 7:**
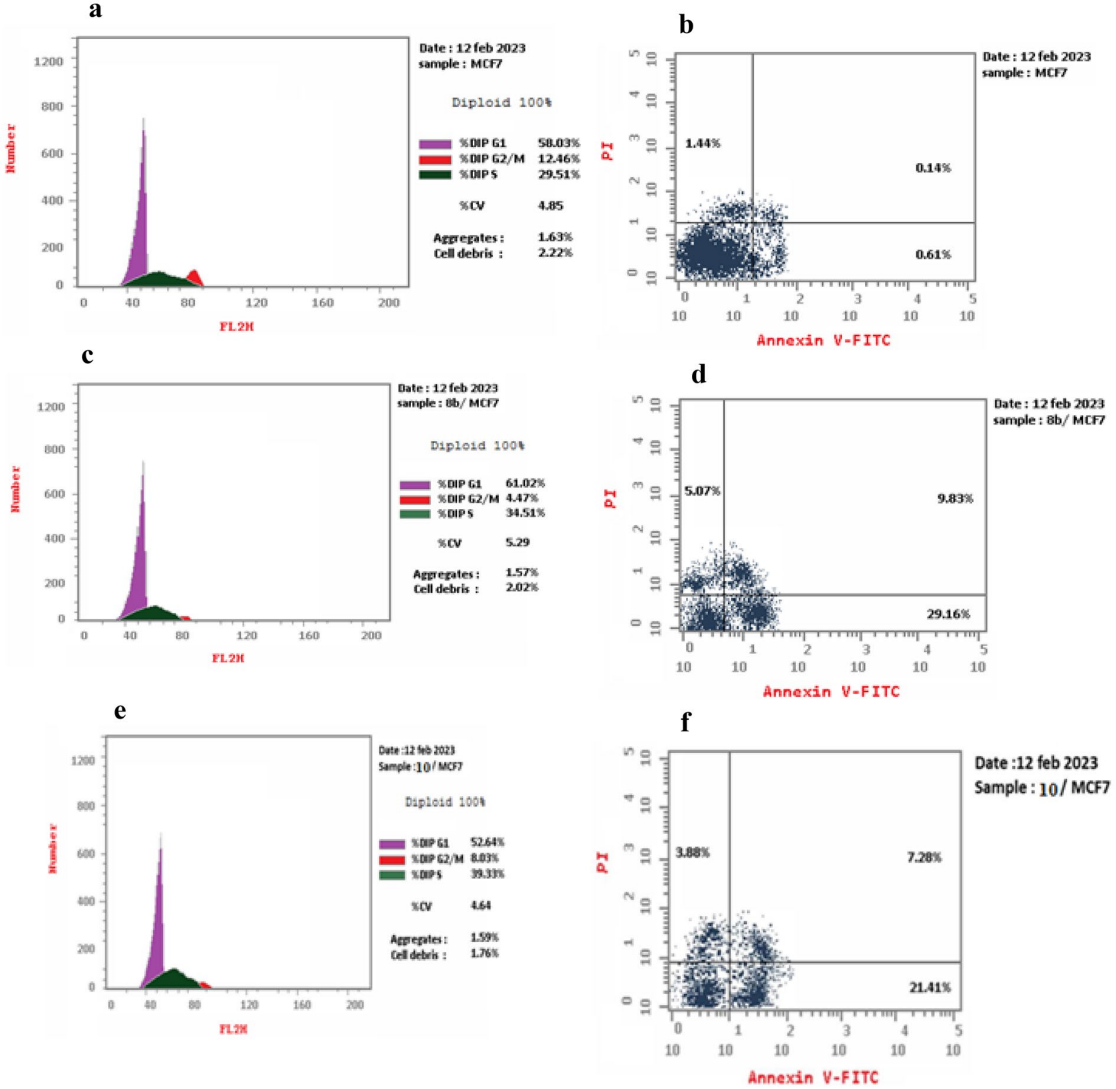
Cell cycle of (**a**, **b**) control MCF7, (**c**, **d**) compounds **8b**/MCF7 and (**e**, **f**) Compounds **10**/MCF7 by flow cytometry using PI staining method

Compounds **8b** and **10** exhibit the highest cytotoxic activity and the dual EGFR^T790M^/ HER2 kinase pro-inhibitors were subjected to further investigation regarding their effects on cell cycle arrest in the MCF7 breast cancer cell line [59]. Compound **8b** increased the percentage of DNA content in the G0-G1phase from 58.03% to 61.02% and in the S phase from 29.51% to 34.51%. In contrast, compound **10** increased the percentage of DNA content in the S phase from 29.51% to 39.33% (Fig. 7a-f). These finding suggest that compounds **8b** and **10** can be arrest breast cancer cell growth at G1/S and S phases, respectively.

### Apoptosis analysis

Compounds **8b** and **10** underwent additional investigated to assess their ability to induce apoptosis by treating MCF7 cells with these compounds, using Annexin V/ propidium iodide (PI) double staining assay as detected by flow cytometry (Table 3). Compounds **8b** and **10** demonstrated significant increases in the early percentages of 29.16% and 21.41%, along with late percentages of 9.83% and 7.28%, respectively. This indicates a 47.8-fold and 35-fold increase in early apoptosis induction, and a 70.2-fold and 52-fold increase in late-stage apoptosis, respectively. Furthermore, it was observed that compounds **8b** and **10**, resulted in significant induction levels of 38.99 and 28.69, respectively.

**Table 3:** Percentage of cell death induced in MCF7 cells treated with compounds **8b** and **10**

Comp. no.	Apoptosis %			Necrosis
	Total	Early	Late	
**8b/ MCF7**	44.06	29.16	9.83	5.07
**10/ MCF7**	32.57	21.41	7.28	3.88
**Cont. MCF7**	2.19	0.61	0.14	1.44

### Bcl2 (anti-apoptotic protein) inhibition assay

The activity of Bcl2 in MCF7 cells was evaluated using compounds **8a** and **10** with the Elisa assay (Further details can be found in the supplementary file). A notable decrease in Bcl2 expression levels was observed with compound **8b** (4.29 ng/mg) in comparison to the untreated control (4.57 ng/mg). Relative to the control, compound **10** also led to a reduction in Bcl2 expression level from 4.57 ng/mg to 4.41 ng/mg. This data suggests that compound **8b** exerts a more significant impact on Bcl2 expression level than compound **10**. Additionally, both compounds **8b** and **10** act as inducers of apoptosis, showing their ability to induce cancer cell death through the regulation of the mitochondrial apoptosis pathway in breast cancer cell lines, as outlined in Table 4.

**Table 4:** Determination of Bcl2 expression level in MCF7 tested cancer cells

Group	Protein quantifi- cation	O.D (R1)	O.D (R2)	O.D (R3)	Aver. Abs	Log (aver. Abs)	Conc. (ng/mg)	Conc. (ng/mg/ protein)
Untreated/MCF7	0.61	1.92	1.99	2.07	1.99	0.445952	2.79	4.57
Treated **8b**/MCF7	0.63	1.87	1.95	1.99	1.93	0.432367	2.70	4.29
Treated **10**/MCF7	*0.6*	*1.83*	*2.06*	*1.79*	*1.89*	*0.423073*	*2.64*	*4.41*

### Molecular docking study

#### Ligand structure preparation

The molecular geometry of compounds **8b**, **10**, and erlotinib was fully optimized utilized density functional theory with the Becke’s three-parameter exchange functional and the gradient corrected functional of Becke, Johnson, and Lee (DFT/B3LYP) using the largest basis set 6-311 + + G (d, p) [60, 61]. The Auto Dock tools version 1.5.6rc3 [62] were utilized for mapping prior to molecular docking simulation.

#### Protein receptors preparation

The crystal structure of the target receptor`s TKD domain, specifically that of the EGFR or HER family of receptor tyrosine kinases (PDB ID: 5JEB) [63, 64] was obtained from the PDB (http://www.rcsb.org/pdb/).

#### Method of molecular docking and analysis

Molecular docking and binding affinity calculations were performed using Auto Dock Vina [65].

#### Docking validation

The docking procedure was validated using re-docked co-crystallized complex of receptor tyrosine kinases (PDB ID: 5JEB) following the same protocol. This was conducted to confirm that inhibitor binds precisely to the active site cleft and demonstrates minimal deviation when compared to the actual co-crystallized complex [66, 67]. Subsequently, the re-docked complex was superimposed onto the reference co-crystallized complex using PyMOL 2.3, and the root mean square deviation (RMSD) was calculated equal to 1.120 Å, indicating a high level of reliability of the docking protocol applied in this study.

#### Discussion of molecular docking

**Fig. 8:**
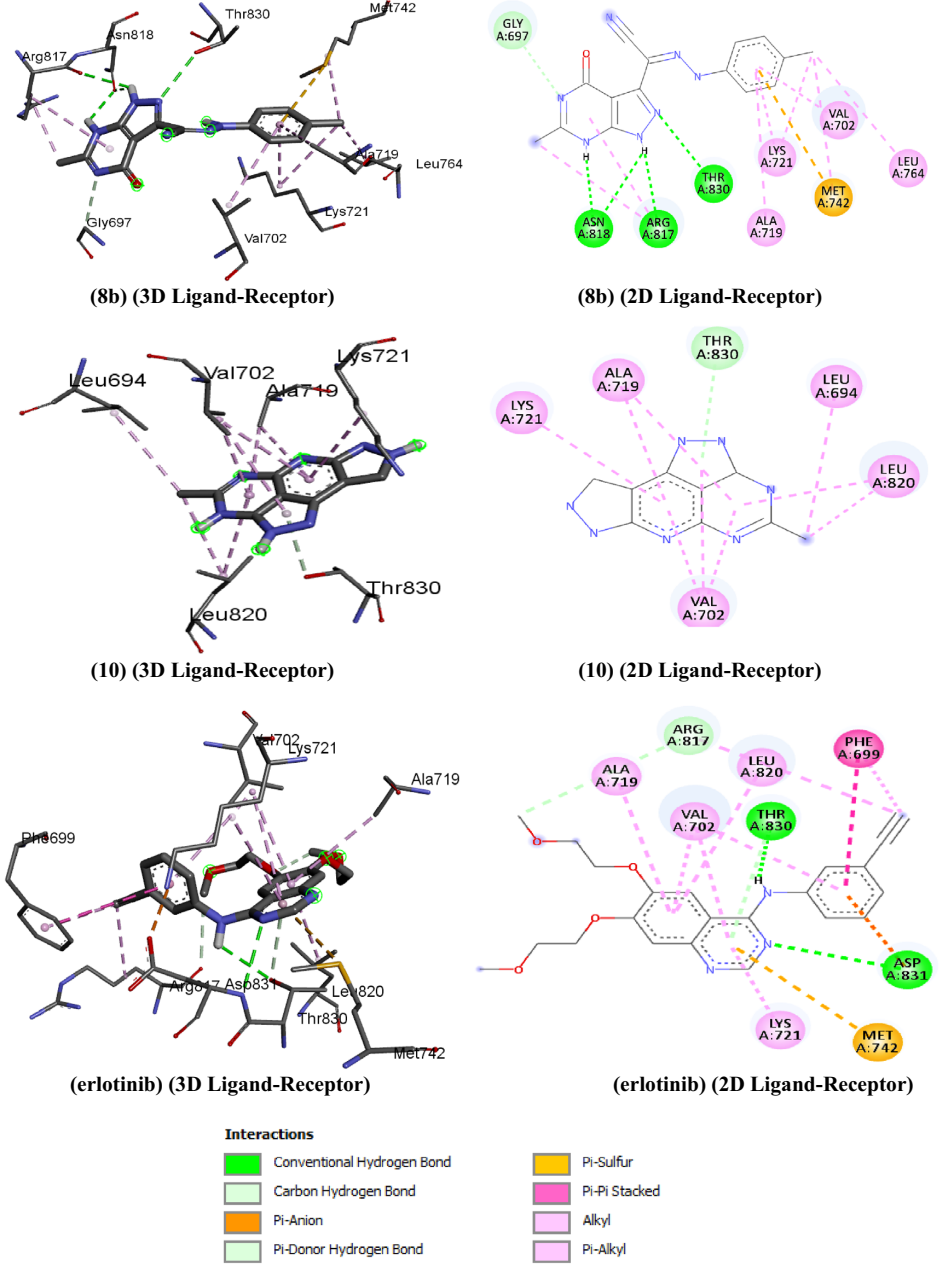
The proposed binding mode of newly synthesized compounds **(8b** and **10)** and erlotinib as reference docked in the active site of EGFR tyrosine kinase domain **(TKD**) **(PDB ID: 5JEB)** receptor (2D and 3D ligand-receptor interactions). (erlotinib), (3D Ligand-Receptor), (erlotinib), (2D Ligand-Receptor)

Utilizing docking interaction methodology, the molecular docking scores and binding interactions, of the novel synthetic ligands **8b**, **10**, in conjunction with the control erlotinib at the active site of the TKD receptor (5JEB) were studied (as illustrated in Scheme 3 and Scheme 5). An analysis of these outcomes reveals that target **8b** and **10** displayed binding free energies (ΔG_b_) within the range of -9.2 to -7.5 kcal/mol, while the control erlotinib demonstrates a binding free energy of -7.6 kcal/mol. The graphical depiction in Fig. 8, illustrates the arrangement of the pyrazole and pyrimidine moieties within the catalytic anionic site (CAS) of the enzyme, linked by hydrogen bonds (HBs) between the NH of the pyrazole active centres and the enzyme residues. Compound **8b** established HBs, with ASN818, ARG817, THR830, and ASN818, whereas compound **10** does not engage in HBs interactions. Conversely, erlotinib forms HBs with THR830 and ASP831, exhibiting binding energies of -9.2, -7.5, and − 7.6 kcal/mol. In the examination involving compounds **8b**, **10**, and erlotinib, the configuration of a methyl group, phenyl, and pyrimidine rings demonstrated several interactions through alkyl, π-alkyl and π- (anion or sulphur) bonds with VAL702, LYS721, MET742, LEU764, LEU820, LEU694, ALA719, VAL702, LEU802, LYS721 and, PHE699 amino acid residues. The examination of binding site patterns and binding energies in docking studies between TKD enzyme and compounds **8b** and **10**, in comparison to erlotinib, revealed that **8b** exhibited superior properties relative to **10**, positioning it as compelling candidate for TKD pro-inhibitors. The results obtained from the theoretical study were consistent with the experimental finding (Table 5).

**Table 5:** Molecular docking scores and interaction modes between **8b** and **10** and erlotinib with EGFR tyrosine kinase domain (TKD) (PDB ID: 5JEB) receptor

Ligand	Binding energy (Kcal/mol)	Interactions				
		H-bond	Hydrophobic			Electrostatic or Other
			Alkyl	π-alkyl	π- anion / π sulfur	Carbon H bond / π- π stack
**8b**	-9.2	ASN818, ASN818, ARG817, THR830	VAL702, LYS721, MET742, LEU764	VAL702, ALA719, LYS721	MET742	GLY697 /
**10**	-7.5	–	LEU820, LEU694	VAL702, ALA719, LYS721 LEU820		THR830 /
**Erlotinib**	-7.6	THR830, ASP831	ALA719, VAL702, LEU802, LYS721, PHE699	ALA719, VAL702, LEU802, LYS721, PHE699	MET742	ARG817/ PHE699

#### In silico ADME prediction

The examination of absorption, distribution, metabolism, and excretion (ADME) properties is of considerable significance in the domain of drug discovery [68–70]. In this study, the pharmacokinetic properties of the highly cytotoxic compounds, **8b** and **10**, were investigated employing the free website http://www.swissadme.ch. The characteristics of a boiled egg showed that compounds **8b** and **10** were effectively absorbed in the gastrointestinal tract without causing adverse side effects on the brain. Furthermore, the results demonstrated a remarkably high oral bioavailability (refer to supplementary figures for detailed data).

The evaluation of drug likeness for compounds **8b** and**10** showed compliance with Lipinski’s rule (MWT ≤ 500, number of H bond acceptors ≤ 10, number of H bond donors ≤ 5, calculated logP ≤ 5) and Veber`s rule (TPSA ≤ 140) as detailed in Table 6.

**Table 6:** Lipinski’s parameters and TPSA of the more potent cytotoxic compounds **8b** and **10**

Comp. No.	Lipinski’s parameters and veber					
	TPSA (Ǻ^2^)	Log *P*	MWT	nHBD	nHBA	No. of viol
**8b**	122.61	1.65	307.31	3	5	0
**10**	98.93	0.60	213.20	3	4	0

#### Structure-activity relationship (SAR)

**Fig. 9:**
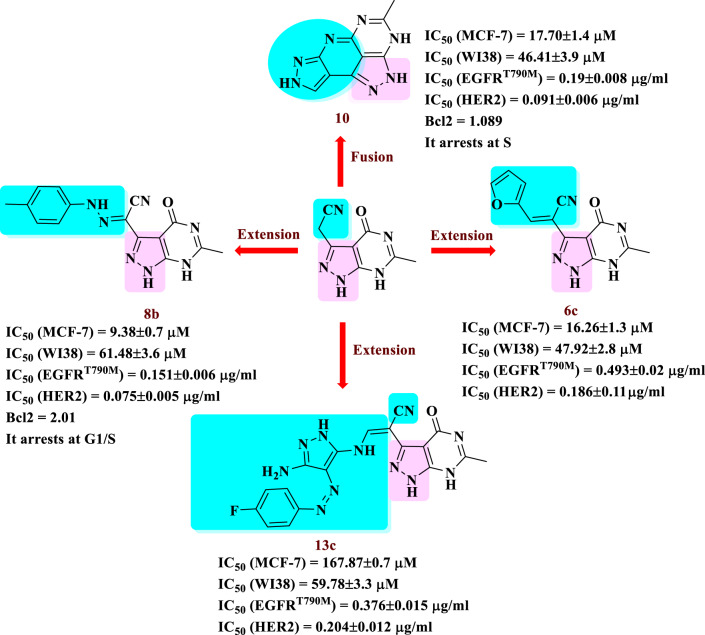
The SAR diagram illustrated the cytotoxicity, enzyme inhibition activity of most potent compounds **6c**, **8b**, **10** and **13c**

A summary of the structure-activity relationship can be derived from the biological assay results obtained. The synthesized compounds **4**, **6a-f**,** 8b**, **8c**,** 10**, **11**, **13b**, and **13c** exhibited diverse levels of cytotoxic activity, ranging from strong to weak. Compound **4** displayed moderate cytotoxic activity against the three cancer cell lines. Modifying compound **4** by introducing a phenyl group, as in compound **6a**, resulted in reduced cytotoxic activity. Conversely, the addition of an OMe donating group to compound **4**, as exemplified by **6b**, produced moderate cytotoxic activity. When a heteroyl ring was integrated, cytotoxic activity displayed variable intensities. The incorporation of a furan moiety into compound **4**, observed in **6c**, significantly enhanced cytotoxic activity across all the three tested cell lines. This resulted in IC_50_ values of 12.68 ± 1.1 (HepG2), 16.26 ± 1.3 (MCF-7), and 23.80 ± 1.8 (HeLa). Conversely, introduction of the pyrazolyl ring resulted in a reduction in cytotoxic activity. On the other hand, the methyl phenyl azo moiety in the phenyl ring of compound **8b** significantly enhanced its anti-tumor properties, achieving IC_50_ = 10.38 ± 0.9 (HepG2), 9.38 ± 0.7 (MCF7), and 13.89 ± 1.0 (HeLa). Furthermore, the four cyclic fused ring as pyrazolo[3,4-*d*]pyrimidine **10**, demonstrated good anti-cancer activity in the same three cell lines. Notably, compound **13c** displayed significant activity, likely due to the presence of a 4-flourophenylazoaminopyrazole moiety. Evaluation of the inhibitory effects on protein kinases EGFR^T970M^, HER2 and Bcl2, deduced that compounds **8b** and **10**, which displayed the most potent cytotoxic activity, also demonstrated substantial kinase inhibition. Results from the Bcl2 assay demonstrated that these compounds induced cell death through Bcl2 activation, ultimately leading to apoptosis. In MCF7 cells, compounds **8b** and **10** effectively triggered cell death via apoptotic pathways. Further evidence from the Annexin V- FITC/ PI assays revealed that these compounds led to increased cell population percentages in both early and late apoptosis stages. Additionally, DNA-flow cytometry analysis revealed that compounds **8b** and **10** caused arrest in the cell cycle at the G1/S and S phases, respectively. These findings align with their ability to inhibit EGFR^T790M^ and HER2 activity successfully (as illustrated in Fig. 9).

## Conclusion

A series of pyrazolo[3,4-*d*]pyrimidines and their related fused derivatives were synthesized and characterized by spectroscopic techniques alongside DFT analysis. The cytotoxic activity of most synthesized compounds was assessed against HepG2, MCF-7 and HeLa cell lines. Among the tested compounds, **6c**,** 8b**,** 10**,** and 13c** demonstrated significantly enhanced cytotoxic effects compared to the others. Specifically, treatment of MCF7 cells with compounds **8b** and **10** resulted in a reduction in expression of EGFR^T790M^, HER2, and Bcl2, leading to cell death through DNA fragmentation. These compounds also induced cell cycle arrest in different phases, with compound **8b** halting the cell cycle at the G1/S and compound **10** at the S phases. Compounds **8b** and **10** Molecular docking studies conducted using the AutoDock Vina program revealed that both compounds exhibited strong interactions with EGFR^T790M^/HER2 targets. These findings highlight the potential of newly synthesized pyrazolo[3,4-*d*]pyrimidine derivatives to act as promising agents for targeting EGFR/HER enzymes, paving the way for the development of novel anticancer therapies, particularly for breast cancer.

### Experimental

All melting points were determined on an Electrothermal (9100) apparatus and are uncorrected. The IR spectra were recorded as KBr pellets on a Perkin Elmer 1430 spectrophotometer. The NMR spectra were recorded with a Varian Mercury VXR-300 NMR spectrometer at 300 and 75 MHz (^1^H and ^13^C NMR spectra, respectively) using DMSO-*d*6 as solvents and results are expressed as δ values. Mass spectra were regional centre for fungi and their applications, Al-Azhar University. Elemental analyses were carried out at the Microanalyses Centre at Cairo University and were performed on Vario EL III Elemental CHNS analyzer.

### General procedure for synthesis of 2-(6-methyl-4-oxo-4,5-dihydro-1*H*-pyrazolo[3,4-*d*]pyrimidin-3-yl)acetonitrile 4

An equivalent amount of 5-amino-3-(cyanomethyl)-1*H*-pyrazole-4-carbonitrile **(1)** (0.735 gm, 5 mmol) was heated with 4,4,4-trifluoroethyl acetoacetate **(2)** (0.92 gm, 5 mmol) in glacial acetic acid for 12 h. The solid that formed was filtered, washed with ethanol, and recrystallized from dioxane.

### 2-(6-Methyl-4-oxo-4,7-dihydro-1*H*-pyrazolo[3,4-*d*]pyrimidine-3-yl)acetonitrile (4)

Pale brown crystals; yield 61%; m.p. 280–282 °C; IR (KBr)*v*_max_/ cm^− 1^; 3290 (NH), 3250 (NH), 2232 (CN), 1693 (CO); ^1^H NMR (DMSO) δ = 2.11 (s, 3 H, CH_3_), 4.14 (s, 2 H, CH_2_ ), 11.21 (s, 1H, NH), 13.41 (s, 1H, NH); ^13^C NMR (DMSO) δ = 16.26, 22.80, 78.36, 112.56, 116.71, 143.35, 143.83, 169.04; m/z = 189.35 (M^+^, 100%), 174.95 (74.31%), 163.63 (53.46%), 161.64 (45.21%), 160.21 (64.98%), 157.23 (46.81%), 138.90 (44.45%), 127.20 (79.88%), 112.24 (33.01%), 84.50 (62.01%), 64.33 (46.86%); Anal. Calcd. C_8_H_7_N_5_O: C, 50.79; H, 3.73; N, 37.02. Found: C, 50.62; H, 3.95; N, 37.19%.

### General procedure for synthesis of compounds 6a-f

A mixture of 2-(6-methyl-4-oxo-4,7-dihydro-1*H*-pyrazolo[3,4-*d*]pyrimidine-3-yl)acetonitrile **(4)** (0.472 gm, 2.5 mmol) and aromatic aldehydes (**5a-f**) (2.5 mmol) in DMF/ piperidine was refluxed for 5–6 h. The precipitate was filtered then washed with water and recrystallized from DMF.

### 2-(6-Methyl-4-oxo-4,7-dihydro-1*H*-pyrazolo-[3,4-*d*]-pyrimidine-3-yl)-3-phenyl-acrylonitrile (6a)

Buff crystals; yield 78%; m.p.> 300 °C; IR (KBr)*v*_max_/ cm^− 1^; 3118 (NH), 3231 (NH), 2234 (CN), 1698 (CO); ^1^H NMR (DMSO) δ = 2.13 (s, 3 H, CH_3_), 7.47–7.60 (m, 5 H, Ar), 7.68 (s, 1H, CH), 10.92 (s, 1H, NH), 13.86 (s, 1H, NH); m/z = 277.91 (M^+^, 4.0%), 276.87 (18.81%), 275.76 (36.76%), 261.77 (3.67%), 250.75 (5.67%), 234.90 (37.64%), 233 − 86 (100%), 207.88 (11.67%), 189.95 (10.74%), 179.82 (30.27%), 150.91 (13.53%), 146.75 (6.56%), 131.91 (6.16%), 118.87 (10.74%), 104.88 (26.40%), 76.87 (38.73%); Anal. Calcd C_15_H_11_N_5_O (277.10): C, 64.97; H, 4.00; N, 25.26. Found: C, 64.72; H, 4.15; N, 25.10%.

### 3-(4-Methoxyphenyl)-2-(6-methyl-4-oxo-4,7-dihydro-1*H*-pyrazolo[3,4-*d*]-pyrimidine-3-yl) acrylonitrile (6b)

Buff crystals; yield 82%; m.p.> 300 °C; IR (KBr)*v*_max_/ cm^− 1^; 3227 (NH), 3118 (NH), 2233 (CN), 1697 (CO); ^1^H NMR (DMSO) δ = 2.14 (s, 3 H, CH_3_), 3.84 (s, 3 H, OCH_3_), 7.11 (d, 2 H, *J* = 6.9, Ar), 7.90 (d, 3 H, *J* = 6.9 Ar and CH), 11.22 (s, 1H, NH, D_2_O-exchangeable), 13.79 (s, 1H, NH, D_2_O*-*exchangeable); m/z = 307.74 (M^+^, 39.84%), 272.56 (35.04%), 250.18 (60.67%), 241.01 (61.18%), 226.61 (54.29%), 190.02 (34.02%), 166.47 (64.74%), 157.80 (100%), 149.75 (41.52%), 122.74 (55.82%), 109.23 (79.31%), 86.17 (64.31%), 52.85 (84.23%); Anal. Calcd. C_16_H_13_N_5_O_2_: C, 62.53; H, 4.26; N, 22.79. Found: C, 62.38; H, 4.40; N, 22.53%.

### 3-(Furan-2-yl)-2-(6-methyl-4-oxo-4,7-dihydro-1*H*-pyrazolo[3,4-*d*]pyrimidin-3-yl) acrylonitrile (6c)

Deep brown crystals; yield 80%; m.p.> 300 °C; IR (KBr)*v*_max_/ cm^− 1^; 3419 (NH), 2224 (CN), 1618 (CO); ^1^H NMR (DMSO) δ = 2.45 (s, 3 H, CH_3_), 6.67–7.77 (m, 3 H, furan), 8.05 (s, 1H, CH), 11.04 (s, 1H, NH), 13.32 (s, 1H, NH); m/z = 267.16 (M^+^, 75.11%), 254.52 (41.09%), 240.48 (38.30%), 215.30 (33.38%), 189.40 (57.41%), 157.06 (41.76%), 126.34 (100%), 92.33 (44.09%), 78.86 (71.28%), 63.36 (66%), 50.70 (57.77%); Anal. Calcd. C_13_H_9_N_5_O_2_: C, 58.43; H, 3.39; N, 26.21. Found: C, 58.55; H, 3.53; N, 26.34%.

### 2-(6-Methyl-4-oxo-4,7-dihydro-1*H*-pyrazolo[3,4-*d*]pyrimidin-3-yl)-3-(thiophen-2-yl) acrylonitrile (6d)

Brown crystals; yield 88% ; m.p.> 300 °C; IR (KBr)*v*_max_/ cm^− 1^; 3293 (NH), 3254 (NH), 2231 (CN), 1692 (CO); ^1^H NMR (DMSO) δ = 2.14 (s, 3 H, CH_3_), 7.26 (m, 1H, thiophene), 7.78–7.93 (m, 2 H, thiophen), 8.16 (s,1H, CH) 11.15 (s, 1H, NH), 13.54 (s, 1H, NH); ^13^C NMR (DMSO) δ = 22.90, 97.63, 113.08, 116.13, 128.51, 133.42, 136.19, 136.39, 137.95, 145.03, 169.25; m/z = 283.35 (M^+^, 10%), 275.85 (11.39%), 264.77 (4.19%), 255.64 (2.09%), 242.85 (3.50%), 233.69 (42.77%), 215.81 (2.34%), 208.68 (5.09%), 189.85 (42.44%), 162.83 (33.61%), 146.80 (46.63%), 105.90 (56.43%), 76.86 (100%), 68.96 (40.33%); Anal. Calcd. C_13_H_9_N_5_OS (283.05): C, 55.11; H, 3.20; N, 24.72; S, 11.32. Found: C, 55.63; H, 3.32; N, 24.59; S, 11.17%.

### 3-(1,3-Diphenyl-1*H*-pyrazol-4-yl)-2-(6-methyl-4-oxo-4,7-dihydro-1*H*-pyrazolo[3,4-*d*pyrimidin-3-yl)acrylonitrile (6e)

Pale yellow crystals; yield 65%; m.p.> 300 °C; IR (KBr)*v*_max_/ cm^− 1^; 3432 (NH), 3235 (NH), 2231 (CN), 1693 (CO); ^1^HNMR (DMSO) δ = 2.14 (s, 3 H, CH_3_), 7.45–7.54 (m, 5 H, Ar), 7.55–7.72 (m, 5 H, Ar), 7.95 (s, 1H, pyrazole), 9.17 (s, 1H, CH), 11.20 (s, 1H, NH), 13.80 (s, 1H, NH); ^13^CNMR (DMSO) δ = 22.92, 113.21, 115.14, 116.45, 119.53, 127.91, 128.46, 128.81, 129.15, 130.01, 131.06, 134.99, 138.86, 153.80, 169.36; m/z = 419.74 (M^+^, 30.76%), 380.64 (47.89%), 367.92 (52.05%), 289.61 (80.76%), 265.22 (100%), 225.26 (54.92%), 189.20 (29.90%), 142.01 (79.54%), 125.22 (91.59%), 86.19 ( 53.71%), 53.79 (61.96%); Anal. Calcd. C_24_H_17_N_7_O: C, 68.72; H, 4.09; N, 23.38. Found: C, 68.59; H, 4.22; N, 23.24%.

### 2-(6-Methyl-4-oxo-4,7-dihydro-1*H*-pyrazolo[3,4-*d*]pyrimidin-3-yl)-3-(1-phenyl-3-(*p*-tolyl)-1*H*-pyrazol-4-yl)acrylonitrile (6f)

Yellow crystals; yield 70%; m.p.> 300 °C; IR (KBr)*v*_max_**/** cm^− 1^; 3429 (NH), 3287 (NH), 2231 (CN), 1692 (CO); ^1^HNMR (DMSO) δ **=** 2.14 (s, 3 H, CH_3_), 2.38 (s, 3 H, CH_3_), 7.32 (d, 2 H, *J* = 8.1, Ar), 7.44 (t, 1H, Ar), 7.57–7.62 (m, 6 H, Ar), 7.94 (s, 1H, pyrazole), 9.15 (s, 1H, CH), 11.22 (s, 1H, NH), 13.80 (s, 1H, NH); ^13^C NMR (DMSO) δ = 20.99, 22.88, 113.20, 115.02, 116.43, 119.45, 127.79, 128.21, 128.36, 128.64, 129.68, 129.95, 135.13, 138.66, 138.84, 153.76, 169.29; m/z = 433.34 (M^+^, 2.73%), 390.0 (5.90%), 369.47 (2.15%), 321 60 (46.99%), 291.71 (13.36%), 263.82 (7.88%), 234.04 (13.20%), 189.80 (92.23%), 162.83 (57.38%), 146.89 (100%), 124.80 (32.39%), 90.92 (28.66% ), 74.80 (36.79%); Anal. Calcd. C_25_H_19_N_7_O (433.17): C, 69.27; H, 4.42; N, 22.62. Found: C, 69.34; H, 4.29; N, 22.75%.

### Synthesis of compounds 8a-c

To a cold solution of **4** (0.472 gm, 2.5 mmol) in pyridine, was added the appropriate diazonium salt of aromatic amine (2.5 mmol), which prepared as usual by diazotizing the corresponding aromatic amines **7a-c** (2.5 mmol) in concentrated hydrochloric acid with sodium nitrite (0.173 gm, 2.5 mmol). The addition was carried out drop wise with stirring at 0–5 °C. After complete addition, the mixture stirring in ice bath for few minutes, the precipitated solid was collected by filtration, washed with water, dried, and recrystallized from an ethanol-dioxane mixture to give compounds **8a-c**.

### 6-Methyl-4-oxo-*N*-phenyl-4,7-dihydro-1*H*-pyrazolo[3,4-*d*]pyrimidine-3-carbo-hydrazonoyl cyanide (8a)

Orange crystals; yield 80%; m.p 260–262 °C; IR (KBr)*v*_max_/ cm^− 1^; 3292 (NH), 3253 (NH), 3129 (NH), 2231 (CN), 1691 (CO); ^1^H NMR (DMSO) δ **=** 2.10 (s, 3 H, CH_3_), 7.38–7.95 (m, 5 H, Ar), 11.22 (s, 1H, NH), 11.45 (s, 1H, NH), 13.46 (s, 1H, NH); m/z = 293.65 (M^+^, 37.69%), 272.19 (100%), 252.08 (69.23%), 225.33 (27.21%), 207.58 (33.20%), 189.40 (12.44%), 133.78 (34.65%), 122.40 (40.36%), 104.29 (37.95%), 82.45 (35.47%), 71.56 ( 11.80%); Anal. Calcd. C_14_H_11_N_7_O: C, 57.33; H, 3.78; N, 33.43. Found: C, 57.55; H, 3.65; N, 33.58%.

### 6-Methyl-4-oxo-*N*-(*p*-tolyl)-4,7-dihydro-1*H*-pyrazolo[3,4-*d*]pyrimidine-3-carbo-hydrazonoyl cyanide (8b)

Orange crystals; yield 81%; m.p. 215–217 °C; IR (KBr)*v*_max_/ cm^− 1^; 3428 (2NH), 3257 (NH), 2232 (CN), 1692 (CO); ^1^H NMR (DMSO) δ = 2.11 (s, 3 H, CH_3_), 2.25 (s, 3 H, CH_3_), 7.11 (d, 2 H, *J* = 8.1, Ar), 7.41 (d, 2 H, *J* = 7.8, Ar), 11.15 (s, 1H, NH), 11.44 (s, 1H, NH), 13.42 (s, 1H, NH); ^13^C NMR (DMSO) δ = 20.37, 22.79, 66.37, 112.53, 114.83, 129.64, 140.76, 168.98; m/z = 307.40 (M^+^, 71.99%), 290.29 (65.74%), 262.25 (52.13%), 236.01 (56.13%), 189.49 (28.08%), 157.86 (100%), 122.39 (24.26%), 99.43 (36.92%), 72.98 (47.18%); Anal. Calcd. C_15_H_13_N_7_O: C, 58.63; H, 4.26; N, 31.90. Found: C, 58.50; H, 4.33; N, 31.85%.

### *N*-(4-Chlorophenyl)-6-methyl-4-oxo-4,7-dihydro-1*H*-pyrazolo[3,4-*d*] pyrimidine-3-carbohydrazonoyl cyanide (8c)

Orange crystals; yield 86%; m.p. 220–222 °C; IR (KBr)*v*_max_/ cm^− 1^ 3294 (NH), 3256 (NH), 3129 (NH), 2231 (CN), 1693 (CO); ^1^H NMR (DMSO) δ = 2.12 (s, 3 H, CH_3_), 7.19 (d, 2 H, *J* = 8.1, Ar), 7.45 (d, 2 H, *J* = 8.1, Ar), 11.13 (s, 1H, NH), 11.40 (s, 1H, NH), 13.56 (s, 1H, NH); m/z = 427.16 (M^+^, 1.89%), 321.93 (15.64%), 291.80 (4.36%), 275.81(5.09%), 234.06 (12.593%), 223.43 (5.16%), 189.90 (26.22%), 162.84 (15.61%), 147.08 (16.86%), 109.83 (100%), 80.86 (39.92%), 68.88 (38.11%); Anal. Calcd.C_14_H_10_ClN_7_O (327.06): C, 51.31; H, 3.08; Cl, 10.82; N, 29.92. Found: C, 51.43; H, 3.23; N, 29.79%.

### General procedure for synthesis of compound 9

(0.945 gm, 5 mmol) of **4** was refluxed with (0.62 ml, 5 mmol) DMF-DMA in dry dioxane for 4 h. The solid product was filtered washed with absolute ethanol and recrystallized from DMF.

### 3-(Dimethylamino)-2-(6-methyl-4-oxo-4,7-dihydro-1*H*-pyrazolo[3,4-*d*]pyrimidin-3-yl)-acrylonitrile (9)

Red crystals; yield 82%; m.p.> 300 °C; IR (KBr)*v*_max_/ cm^− 1^; 3228 (NH), 3125 (NH), 2231(CN), 1693 (CO); ^1^H NMR (DMSO) δ = 2.04 (s, 3 H, CH_3_), 3.20 (s, 3 H, CH_3_), 3.32 (s, 3 H, CH_3_), 7.39 (s, 1H, CH), 10.45 (s,1H, NH), 13.20 (s, 1H, NH); ^13^CNMR (DMSO) δ = 21.0, 22.75, 24.0, 66.43, 113.73, 147.0, 152.79, 156.85, 168.76; m/z = 244.16 (M^+^, 5.67%), 228.13 (8.10%), 201.30 (9.20%), 193.86 (8.10%), 173.85 (5.78%), 156.68 (67.64%), 124.77 (14.40%), 110.26 (44.40%), 72.76 (60.76%), 67.56 (100%), 56.95 (55.42%); Anal. Calcd. C_11_H_12_N_6_O (244.11): C, 54.09; H, 4.95; N, 34.41. Found: C, 54.22; H, 4.82; N, 34.55%.

### General procedure for synthesis of compound 10

Enamine derivative **9** (1.22 gm, 5 mmol) was refluxed with hydrazine hydrate (5 mmol) in DMF containing few drops of piperidine, the product so formed was washed and recrystallized from DMF to furnish **10**.

### 3-(3-Amino-1*H*-pyrazol-4-yl)-6-methyl-1,7-dihydro-4*H*-pyrazolo[3,4-*d*]pyrimidin-4-one (10)

Orange crystals; yield 60%; m.p.> 300 °C; IR (KBr)*v*_max_/ cm^− 1^; 3419 3119 (3NH); ^1^H NMR (DMSO) δ **=** 2.15 (s, 3 H, CH_3_), 7.83 (s, 1H, pyrazole), 10.94 (s, 1H, NH), 13.54 (s,1H, NH), 13.68 (s,1H, NH); m/z = 213.09 (M^+^, 25.61%), 182.29 (100%), 161.88 (68.98%), 148.61 (74.06%), 124.42 (43.07%), 109.28 (40.79%), 78.92 (73.85%), 99.43 (36.92%), 72.98 (47.18%), 56.43 (55.07%), 40.82 (42.99%); Anal. Calcd. C_9_H_7_N_7_: C, 50.70; H, 3.31; N, 45.99. Found: C, 50.53; H, 3.18; N, 45.84%.

### General procedure for synthesis of compound 11

An equimolar amount of enamine derivative **9** (1.22 g, 5 mmol) and guanidine hydrochloride (5 mmol) was heated under reflux in DMF with few drops of piperidine. Solid product was collected by filtration, washed with ethanol, and recrystallized from DMF to give **11**.

### 3-(2,4-Diaminopyrimidin-5-yl)-6-methyl-1,7-dihydro-4*H*-pyrazolo[3,4-*d*]-pyrimidin-4-one (11)

Orange crystals; yield 70%; m.p.> 300 °C; IR (KBr)*v*_max_/ cm^− 1^: 3397 (2NH and NH_2_); ^1^HNMR (DMSO) δ = 2.19 (s, 3 H, CH_3_), 6.51 (s, 2 H, NH_2_), 8.88 (s, 1H, CH, pyrimidine), 10.85 (s, 1H, NH), 13.50 (s, 1H, NH); m/z = 241.47 (M^+^ +1, 97.56%), 233.27 (69.28%), 225.73 (100%), 211.93 (66.32%), 190.04 (38.22%), 182.68 (58.56%), 124.49 (49.48%), 123.07 (80.45%), 106.79 (41.29%), 82.56 (33.27%), 56.30 (31.28%); Anal. Calcd. C_10_H_8_N_8_: C, 50.00; H, 3.36; N, 46.65. Found: C, 50.15; H, 3.49; N, 46.53%.

### General procedure for synthesis of compound 13a-c

A mixture of enaminone **9** (0.61 gm, 2.5 mmol) and arylazo aminopyrazoles**12a-c** (2.5 mmol) was refluxed in DMF containing a catalytic amount of piperidine for 4 h. The solid product so formed was allowed to cool, filtered, washed with ethanol, and recrystallized from DMF to afford compounds **13a-c**.

### 3-(2,7-Diamino-3-(phenyldiazenyl)-pyrazolo[1,5-*a*]-pyrimidin-6-yl)-6-methyl-1,7-dihydro-4*H*-pyrazolo[3,4-*d*]-pyrimidin-4-one (13a)

Orange crystals; yield 75%;m.p.> 300 °C; IR (KBr)*v*_max_/ cm^− 1^; 3417 (NH), 3218 (3NH), 2232 (CN), 1691 (CO); ^1^HNMR (DMSO) δ = 2.16 (s, 3 H, CH_3_), 6.94 (s, 2 H, NH_2_), 7.32–7.78 (m, 5 H, Ar), 7.94 (s, 1H, CH, pyrimidine), 10.45 (s, 2 H, 2NH), 11 (s, 1H, NH),14.15 (s, 1H, NH);^13^CNMR (DMSO) δ = 23.26, 66.46, 96.77, 120.73, 120.93, 121.04, 121.21, 127.83, 129.24, 131.46, 140.56, 150.80, 151.05, 156.46, 168.92, 171.36; Anal. Calcd. C_18_H_15_N_11_O (401.15): C, 53.86; H, 3.77; N, 38.39. Found: C, 53.72; H, 3.80; N, 38.26%.

### 3-(2,7-Diamino-3-((3-fluorophenyl)diazenyl)pyrazolo[1,5-*a*]pyrimidin-6-yl)-6-methyl-1,7-dihydro-4*H*-pyrazolo[3,4-*d*]pyrimidin-4-one (13b)

Orange crystals; yield 80%; m.p.> 300 °C; IR (KBr)*v*_max_/ cm^− 1^; 3412 (NH), 3223 (3NH), 2230 (CN), 1694 (CO); ^1^H NMR (DMSO) δ **=** 2.15 (s, 3 H, CH_3_), 7.39 (s, 2 H, NH_2_), 7.48–8.66 (m, 5 H, Ar, and CH), 10.48 (s, 2 H, 2NH), 10.93 (s, 1H, NH),13.95 (s, 1H, NH); ^13^C NMR (DMSO) δ = 22.94, 78.86, 94.33, 114.94, 120.48, 120.59, 131.28, 150.97, 168.71; Anal. Calcd.C_18_H_14_FN_11_O (419.14): C, 51.55; H, 3.36; F, 4.53; N, 36.74. Found: C, 51.68; H, 3.23; N, 36.61%.

### 3-(2,7-Diamino-3-((4-fluorophenyl) diazenyl)-pyrazolo[1,5-*a*]-pyrimidin-6-yl)-6-methyl-1,7-dihydro-4*H*-pyrazolo[3,4-*d*]-pyrimidin-4-one (13c)

Orange crystals; yield 78%; m.p.> 300 °C; IR (KBr)*v*_max_/ cm^− 1^; 3419 (NH), 3226 (3NH), 2233 (CN), 1689 (CO);^1^H NMR (DMSO) δ **=** 2.17 (s, 3 H, CH_3_), 6.26 (s, 2 H, NH_2_), 7.28–8.64 (m, 5 H, Ar, and CH), 10.49 (s, 2 H, 2NH), 10.94 (s, 1H, NH),13.96 (s, 1H, NH); m/z = 418.72 (M^+^ -1, 15.56%), 400.59 (75.83%), 335.34 (100%), 268.26 (80.28%), 227.01 (63.03%), 191.32 (58.11%), 130.47 (69.56%), 105.93 (96.49%), 93 (56.16%), 80.99 (91.89%), 62.05 (56.20%), 55.20 (42.51%);Anal. Calcd. C_18_H_14_FN_11_O: C, 51.55; H, 3.36; F, 4.53; N, 36.74. Found: C, 51.42; H, 3.49; N, 36.61%.

### Biological section

#### MTT assay

The four cell lines, Human lung fibroblast (WI38), HepG2, MCF7 and HeLa were obtained from ATCC through Holding company for biological products and vaccines (VACSERA), Giza, Egypt. Erlotinib was used as a standard anticancer drug for the purpose of comparison. MTT assay was used to assess the inhibitory effect of compounds on cell growth. This colorimetric assay relies on the conversion of the yellow tetrazolium bromide (MTT) to a purple formazan derivative by succinate dehydrogenase mitochondria of living cells. The cell lines were cultured in RPMI-1640 medium supplemented with 10% fetal bovine serum. The antibiotics used consisted of 100 units/ml of penicillin and 100 µg/ml of streptomycin, maintained at a temperature of 37 °C in a 5% CO_2_ incubator. The cell lines were seeds in a 96-well plate at a density of 1.0 × 10^4^ cells/well. At 37 °C for 48 h under a 5% CO_2_. Following incubation, the cells were exposed to varied concentration of compounds and then incubated for 24 h. After 24 h of drug, 20 µl of a 5 mg/ml MTT solution was added and then incubated for 4 hs. 100 µl of dimethyl sulfoxide (DMSO) was added to each well to dissolve the purple formazan that had formed. The colorimetric assay is measured and recorded at an absorbance of 570 nm using a plate reader (EXL 800, USA). The relative cell viability in percentage was calculated as (A570 of the treated sample/A570 of the untreated sample) X 100 [71].

### EGFR^T790^ and HER2 inhibitory assay

Compounds **8b** and **10** were subjected to an Elisa assay to evaluate their in *vitro* inhibitory activity against EGFR ^**T790**^, and HER2. The enzyme was diluted to a concentration of 1 ng/µl using 1x Kinase assay buffer as specified in1. The main mixture was prepared by blending 6 µl 5x Kinase assay buffer 1, 1 µl of ATP (500 µM), 1 µl PTK substrate Poly (Glu: Tyr 4:1) (10 mg/ml) and 17 µl water, which was then incubated with the synthesized compounds at 30 °C for 45 min. Subsequently, add 50 µl of Kinase-Glo Max reagent and allow it to incubate at room temperature for 15 min, before measuring luminescence using a microplate reader [72].

### In Vitro cell cycle analysis

Cell samples of MCF7 cell line, which obtained from ATCC through Holding company for biological products and vaccines (VACSERA), Giza, Egypt were prepared, collected, and set in 66% ethanol on ice, before being stored at + 4 °C for a duration of at least 2 hs. Cells that had been prepared were transferred from 4 °C to room temperature, resuspended with removing supernatant, then washed by gently resuspending in 1 mL 1X PBS. Again, pellet the cells at 500 x g for 5 min and remove the supernatant. Next, the cells gently resuspend in 200 µL 1X Propidium Iodide and RNase staining solution. Incubate at 37 °C in the dark for 20–30 min. Tubes containing samples were placed on ice and dark were prepared for flow cytometry analysis. Propidium iodide fluorescence was collected in FL2 using a 488 nm laser illumination (ab139418 Propidium Iodide Flow Cytometry Kit for cell cycle analysis).

### Apoptosis assay (annexin V-FITC and PI staining)

Once the plasma membrane`s inner layer phosphatidylserine (PS) is relocated to the cell surface, it can be readily identified using a fluorescent Annexin V dye conjugate, as this dye has a high affinity to for PS. The one-step staining procedure takes 10 min and the evidence can be analyzed by flow cytometry or fluorescence microscopy. The kit is able to distinguish between apoptosis and necrosis when Annexin V-FITC and PI staining are both conducted [73].

### Bcl2 (anti-apoptotic protein) inhibition assay

Compounds **8b** and **10** were also investigations warranted using Elisa technique to determine the concentrations of Bcl2 in MCF7 cell line treated with compounds **8b** and **10**. Prepare 7 wells for the standard and one well for the blank, then add 100 µl of the samples or standard and allow them to stand at room temperature for one hour. Each well should be treated with 100 µl of the working solution of detection reagent A, then covered and incubated at 37 °C for one hour. Followed this, the solution should be aspirated and the wells washed. Following the wash step, add 100 µl of the working solution of detection reagent B to each well and repeat the washing process. Subsequently, 90 µl of substrate solution should be added to each, followed by incubation at 37 °C for 10 to 20 min. Shielded from light, the solution turns blue. The solution turned yellow after adding the stop solution, and was thoroughly mixed by gently tapping the plate. Activate the microplate reader and then take the measurement at a wavelength 450 nm (details of procedure and standard curve in supplementary file of Tables).

### Ligand structure preparation

The molecular geometry of compounds **8b**, **10**, and reference erlotinib was fully optimized utilized density functional theory with the Becke’s three-parameter exchange functional and the gradient corrected functional of Becke, Johnson, and Lee (DFT/B3LYP) with the largest basis set 6-311 + + G (d, p). The hybrid functional B3LYP of a large basis set 6-311 + + G (d, p) was selected for their accuracy, consistency, flexibility, excellent performance, and good experimental correlation. No symmetry constrains were applied in the geometry optimization. The same level of theory was performed to compute vibrational frequencies of each compound equating with the real minima of the potential energy surface. The geometrically optimized structural derivatives are saved in pdb file format. Such Pdb files converted to *PDBQT* file format using Auto Dock tools version *1.5.6rc3*, which includes the following steps; root detection and selection of the torsion tree, followed by saving as a pdpqt file, for mapping prior to molecular docking simulation.

### Protein receptors preparation

The first step in the preparation involves specifying the appropriate protein for docking with the proposed molecules, **8b**, **10**, and reference erlotinib. The crystal structure of the target receptor`s TKD domain, specifically that of the EGFR or HER family of receptor tyrosine kinases (PDB ID: 5JEB) was obtained from the PDB (http://www.rcsb.org/pdb/). The second step involves the removal of both proteins from heteroatoms, and this is then followed by molecular mechanics energy minimization using Swiss-PdbViewer. The third step involved the use of *Autodock Tools* version 1.5.6rc3, were polar hydrogen atoms, the Kollman charges were added. The AD4 type atoms were assigned, and the protein was saved in the PDBQT file format.

### Optimal active site prediction

The estimation of the active sites of the developed target proteins was achieved using the Computed Atlas of Surface Topography of proteins (CASTp 3.0 server). The findings were obtained by uploading the PDB file to the website and identifying the location of the active site. The residues at the active site were compressed in order to determine the grid box dimensions suitable for use with the Auto Dock tool.

### Molecular docking and analysis

Molecular docking and binding affinity calculations were performed using Auto Dock Vina. The command line terminal was employed to execute the Auto Dock Vina program. The protein and ligand names were specified in pdbqt format within the configuration file. The dimensions of the grid box encompass the center and sizes of the grid box tailored for the specific protein. All ligand bonds were assumed to be capable of rotation, while the TKD receptor was assumed to be rigid. A grid box size of 80 × 80 × 80 A° was chosen, positioned centrally within the substrate-binding pocket. When running Auto Dock *via* a command terminal, two resultants’ files were produced: a log file with information about the binding affinities of the ligands, and an output file details about the structural arrangement of the docked structures are required. The proposed derivatives` van der waal and hydrogen bond interactions were visualized and analyzed using Discovery Studio Visualizer software (windows version 21.1.0.20218), which created 2D and 3D images of the ligand-receptor complex structures, signifying these interactions. The amino acid residues that interacted with each other were labelled, assigned names, and subsequently saved as an image file.

## Supplementary Information

Supplementary Material 1.

Supplementary Material 2.

Supplementary Material 3.

## Data Availability

All data generated or analyzed in current study are included in this article and its supplementary files (NMR spectra, IR spectra, MS spectra, Figures and Tables).
