# Supplementary material for: Synthesis of new pyrazolo[3,4-d]pyrimidines as potential mutant EGFR/HER2 and Bcl2 inhibitors: anticancer evaluation, DFT, molecular docking and ADME studies
Source: BMC Chem. 2026 May 18;20(1):104. doi: 10.1186/s13065-026-01773-6 (PMC13188323; doi:10.1186/s13065-026-01773-6)
Supplement: Supplementary file 1 — Supplementary Material 1. [file 13065_2026_1773_MOESM1_ESM.docx]

# Synthesis of new pyrazolo[3,4-d]pyrimidines as potential mutant EGFR/HER2 and Bcl2 inhibitors: Anticancer evaluation, DFT, molecular docking and ADME studies

***Nadia Hanafy Metwally*^1^, Zinab Atwa Saad^1^ and Mona Said Mohamed^1^***

*^1^Chemistry Department, Faculty of Science, Cairo University, Giza****,*** *12613, Egypt*

**E-mail:* [*mnadia@sci.cu.edu.eg*](mailto:mnadia@sci.cu.edu.eg%60)


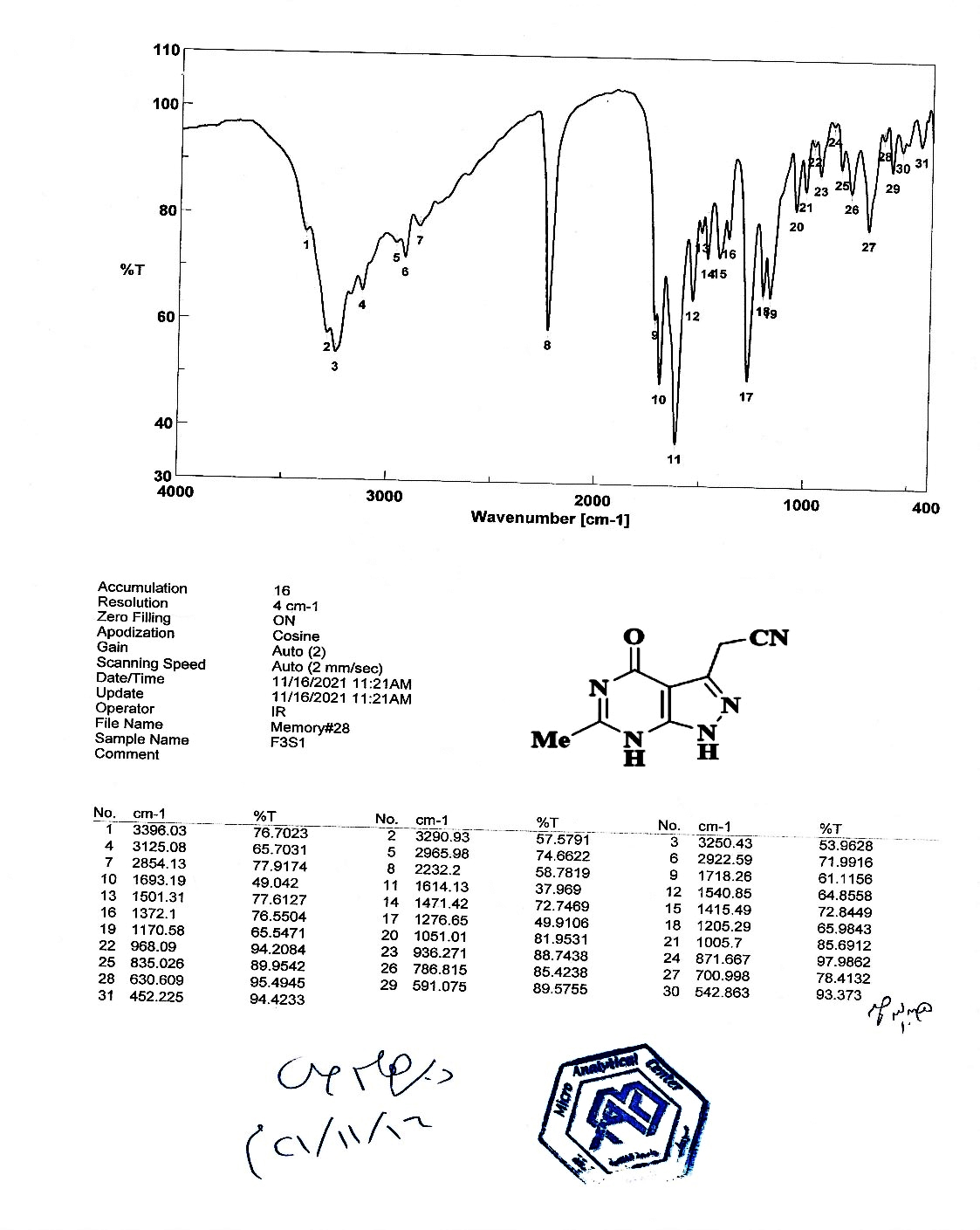


**S1. IR of compound 4**


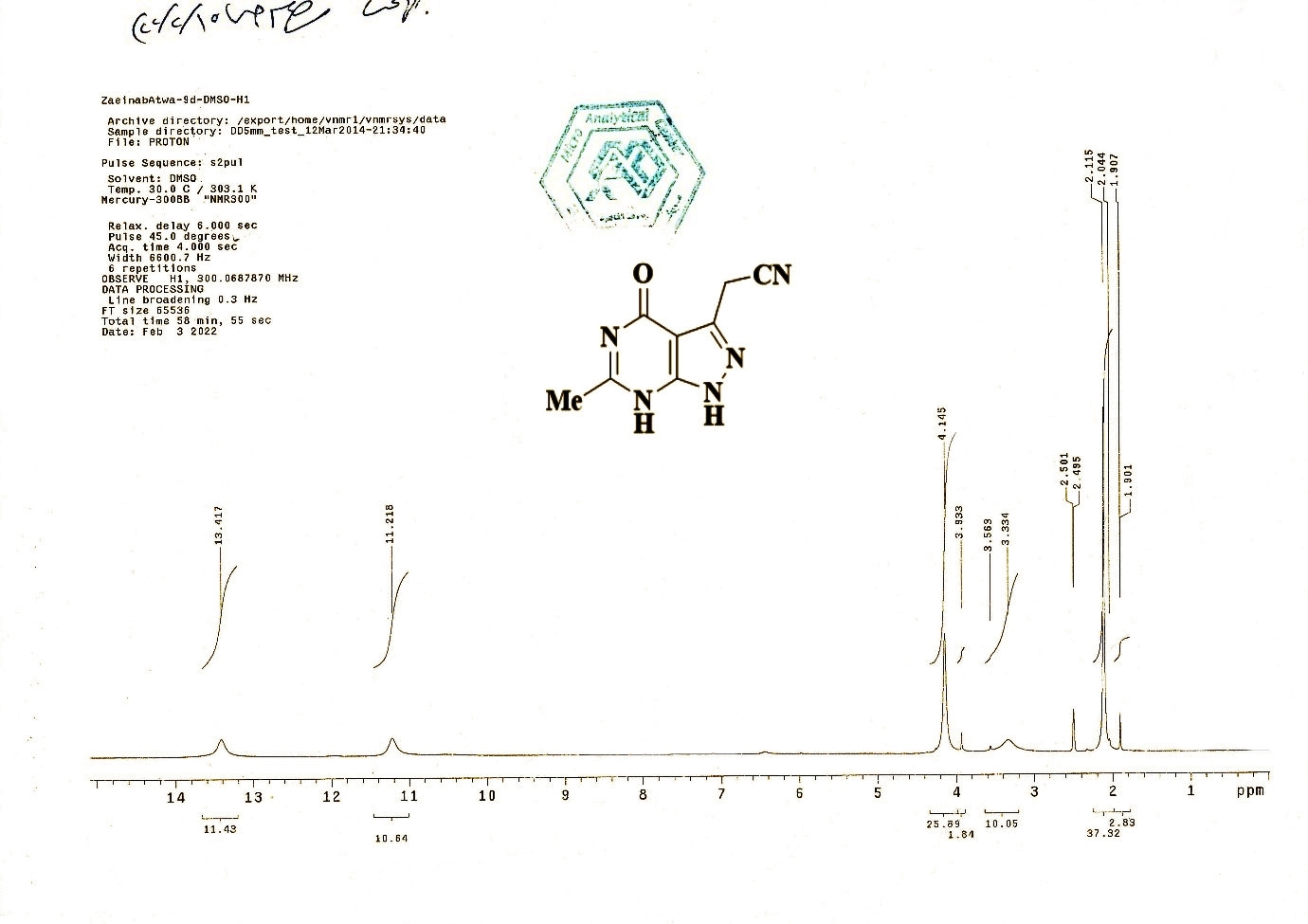


**S2. ^1^H NMR of compound 4**


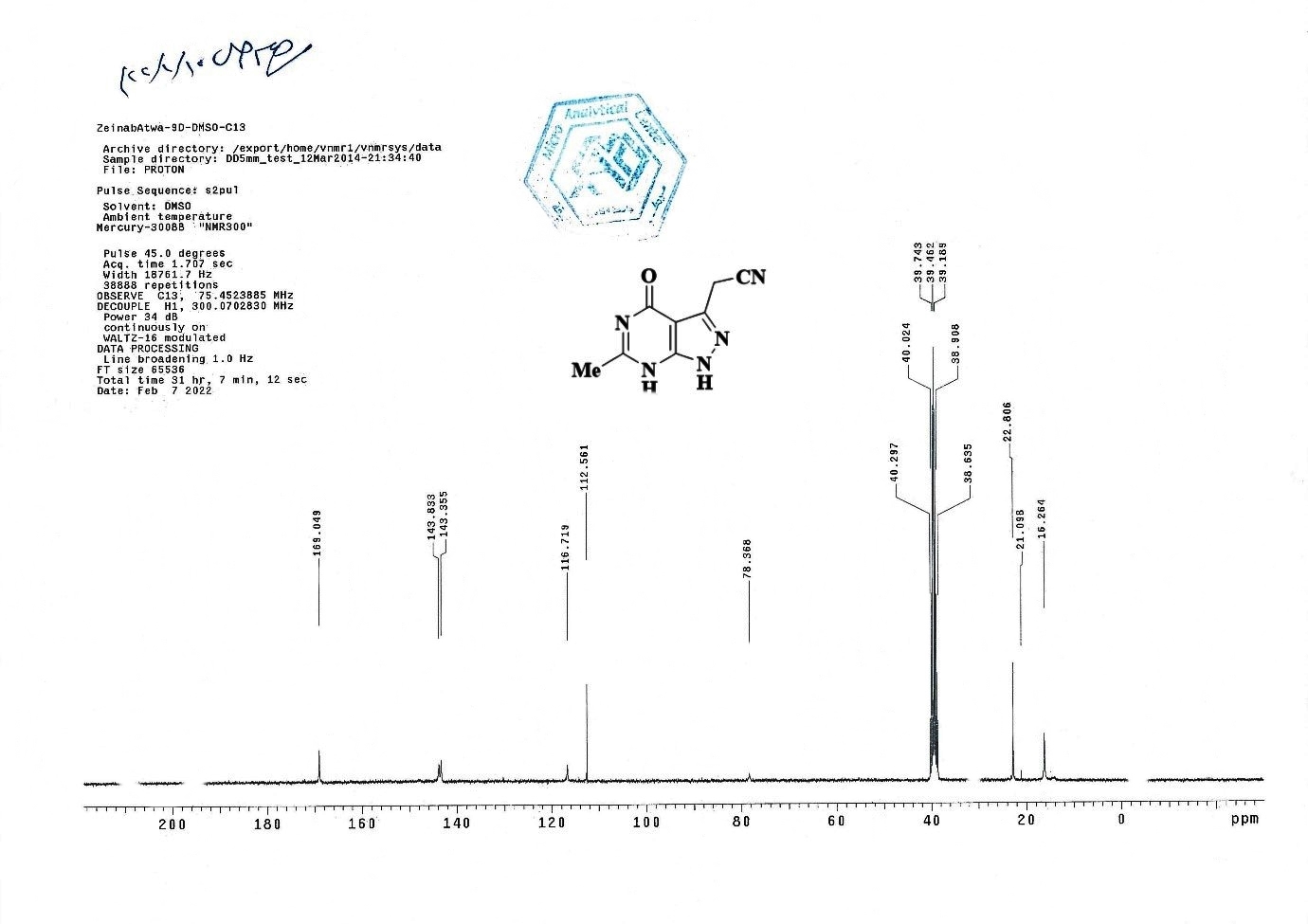


**S3. ^13^C NMR of compound 4**


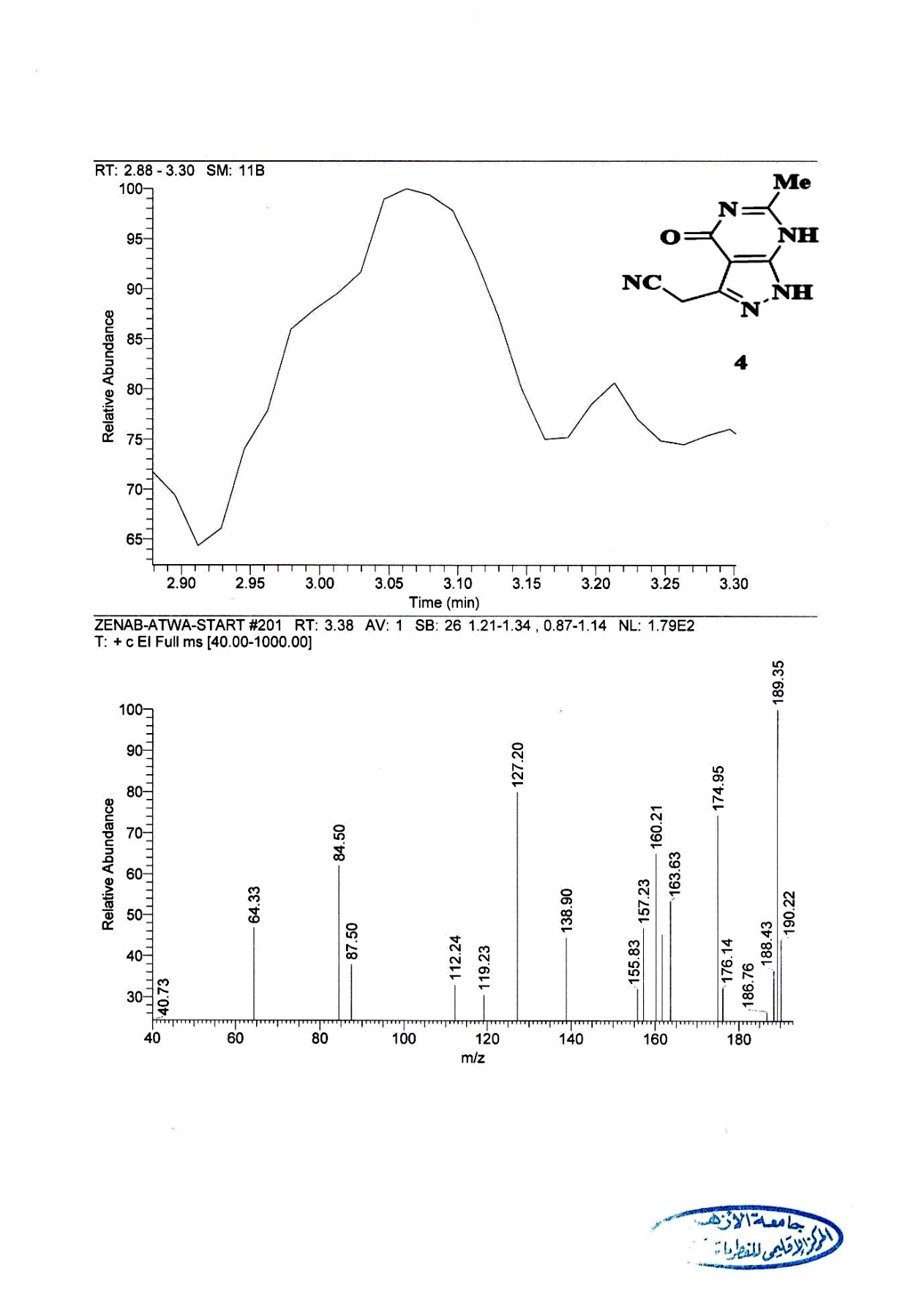


**S4. MS of compound 4**


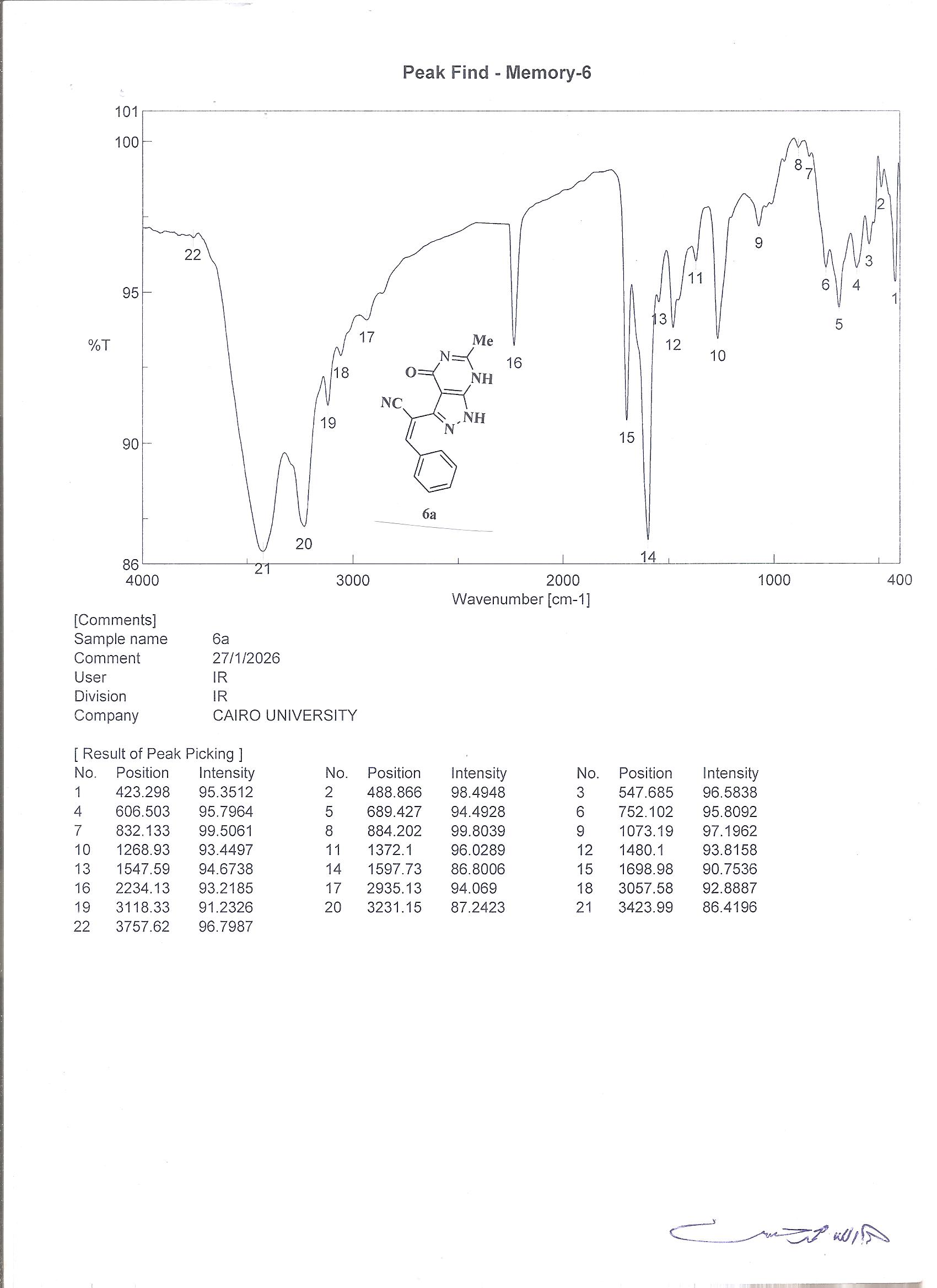


**S5. IR of compound 6a**


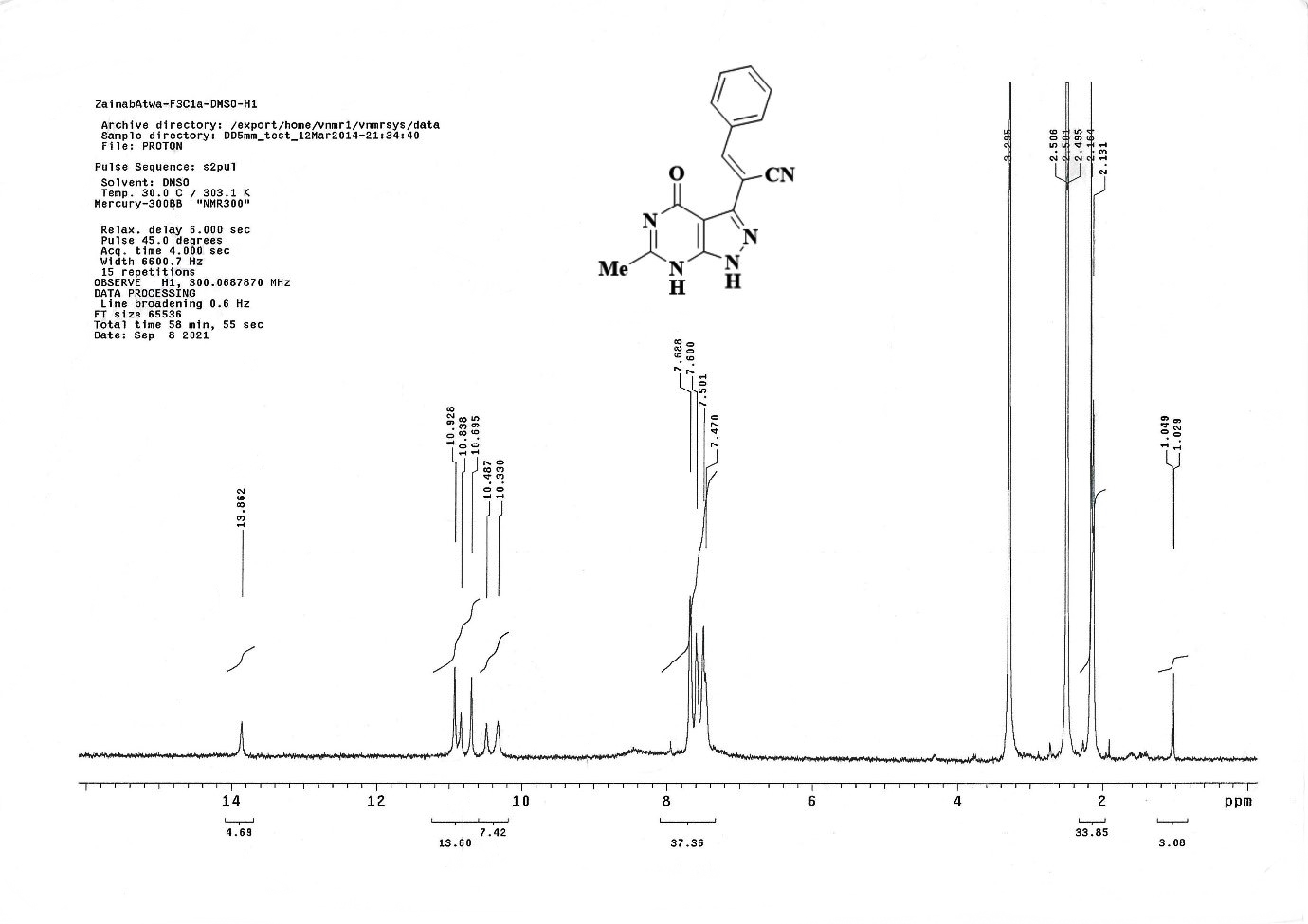


**S6.^1^H NMR of compound 6a**


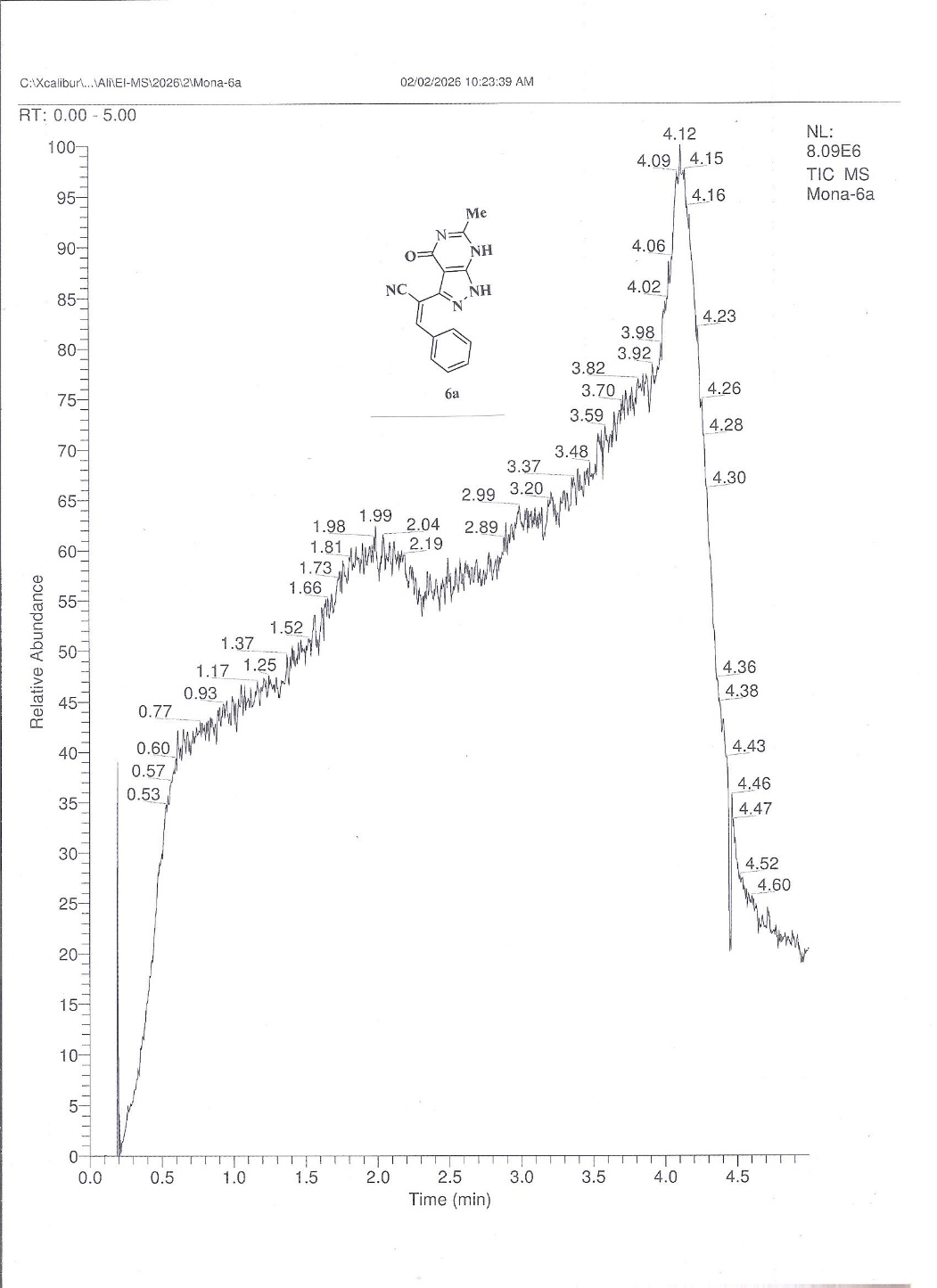


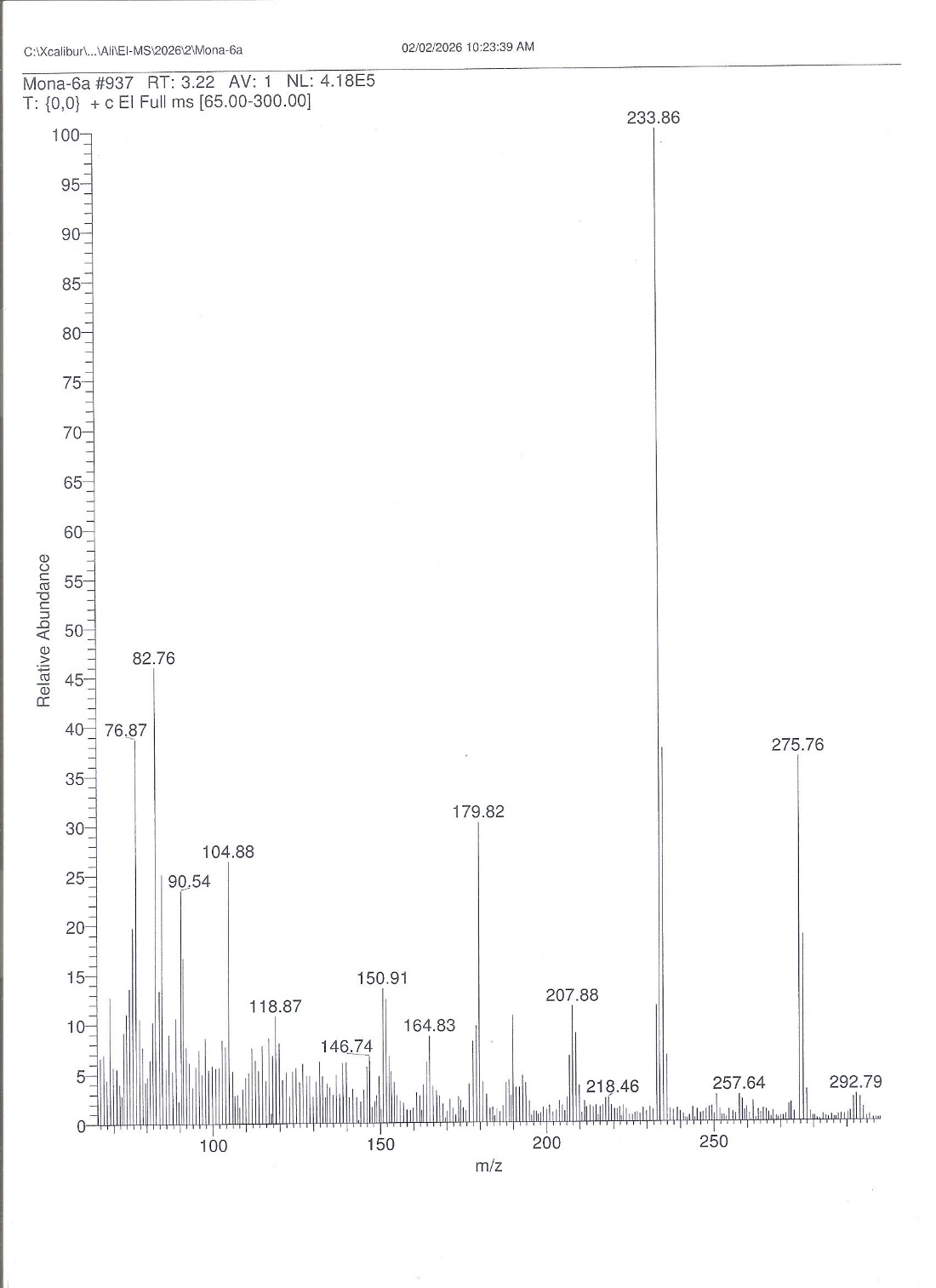


**S7. MS of compound 6a**


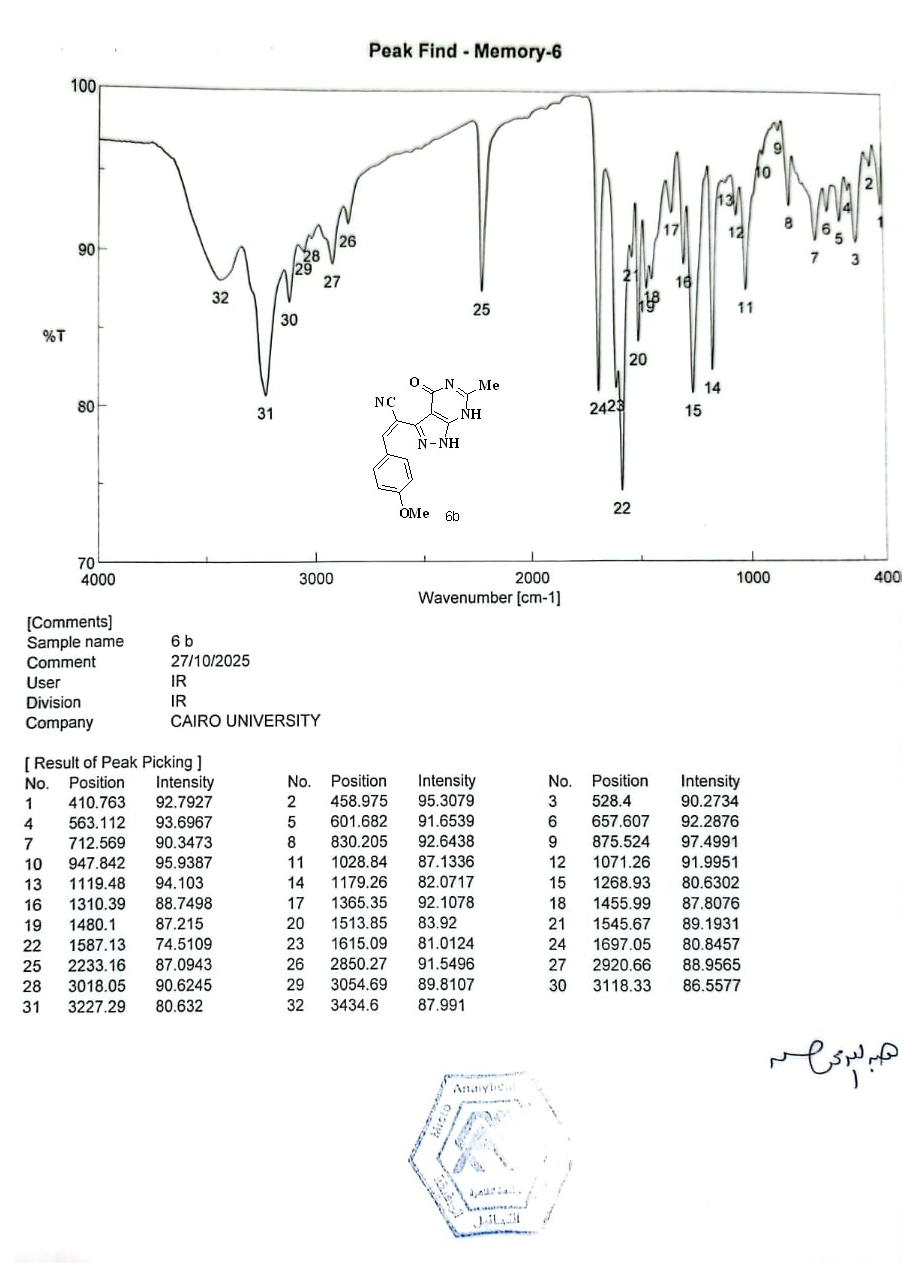


**S8. IR of compound 6b**


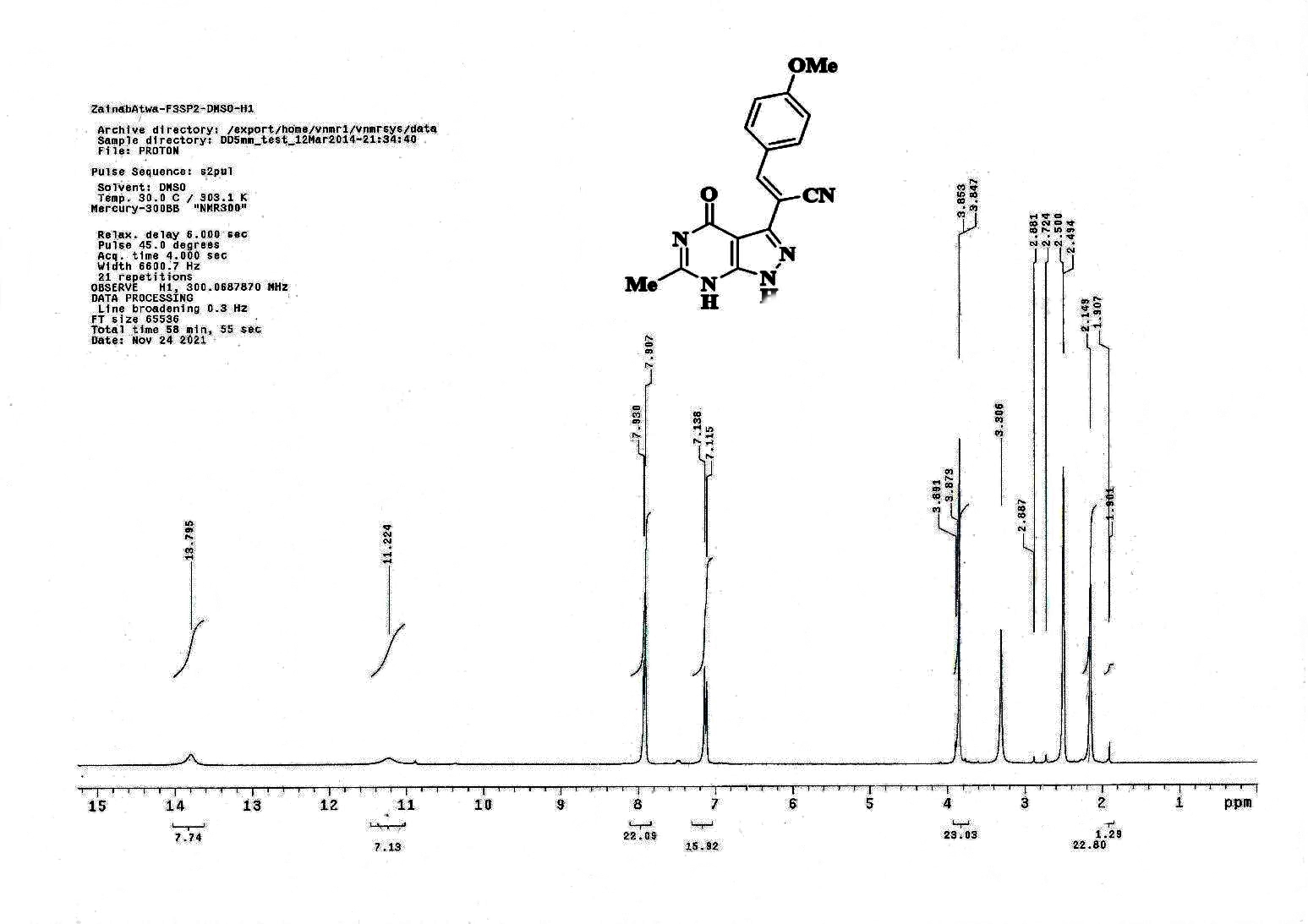


**S9. ^1^H NMR of compound 6b**


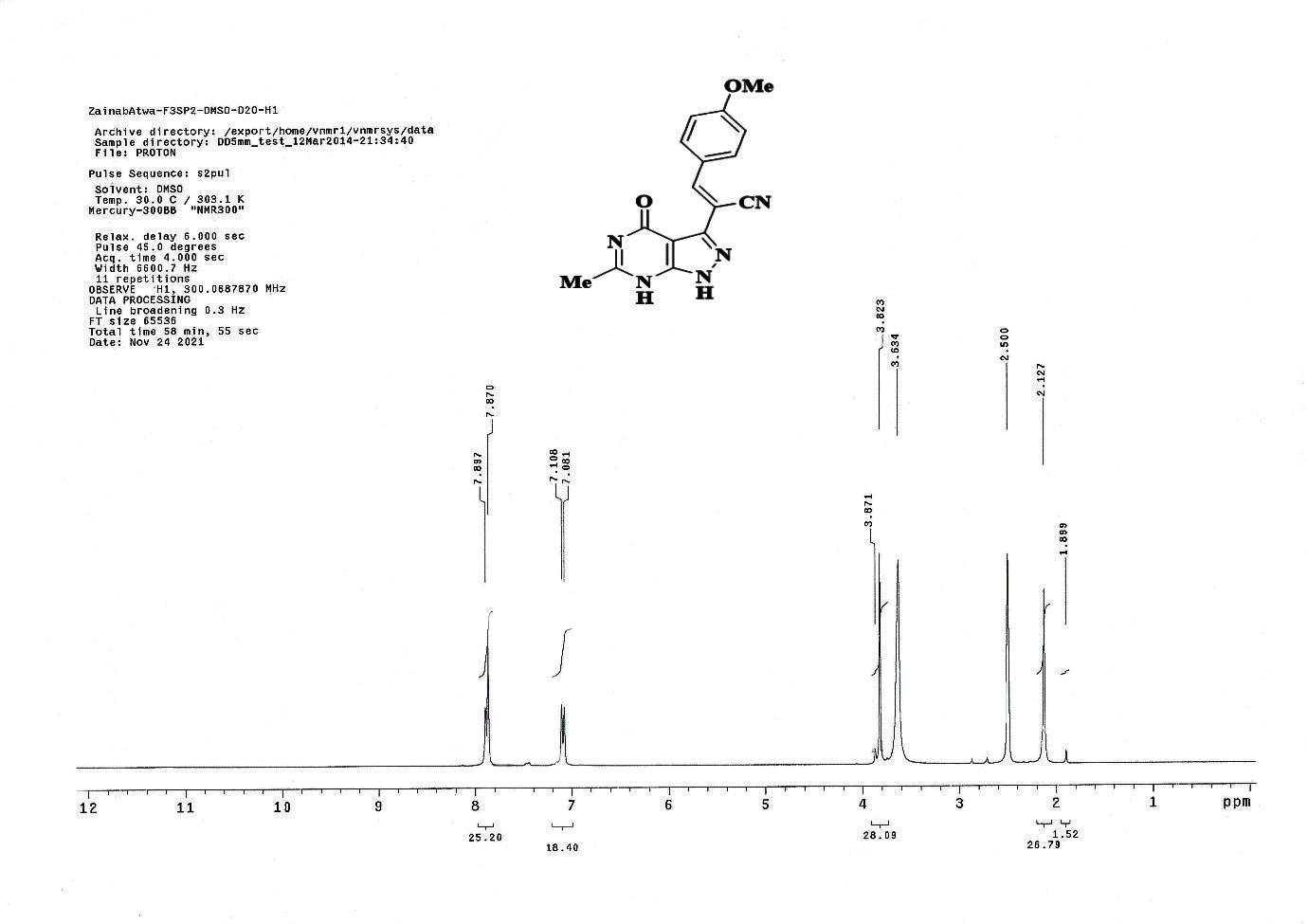


**S10. ^1^H NMR (D_2_O) of compound 6b**


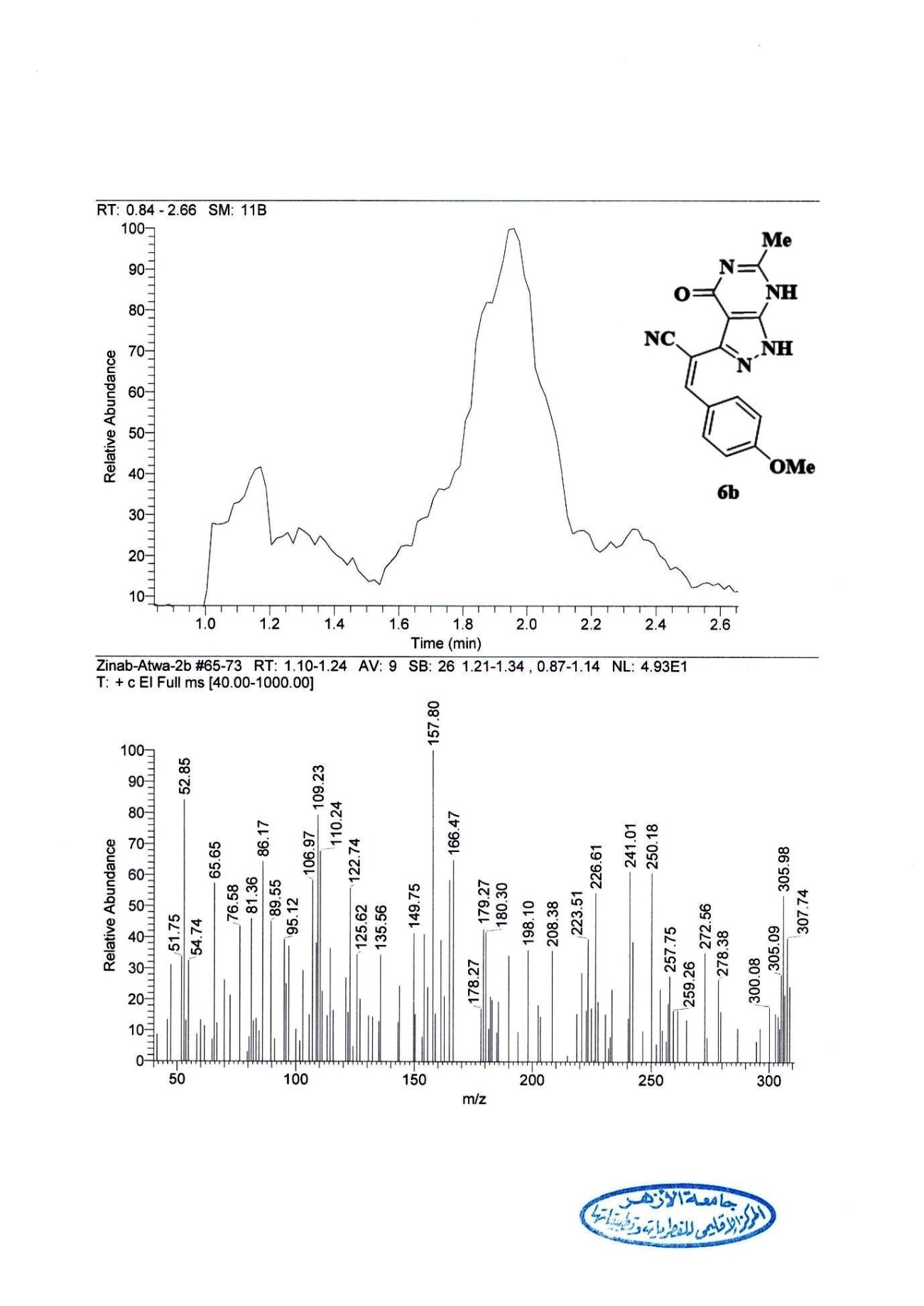


**S11. MS of compound 6b**


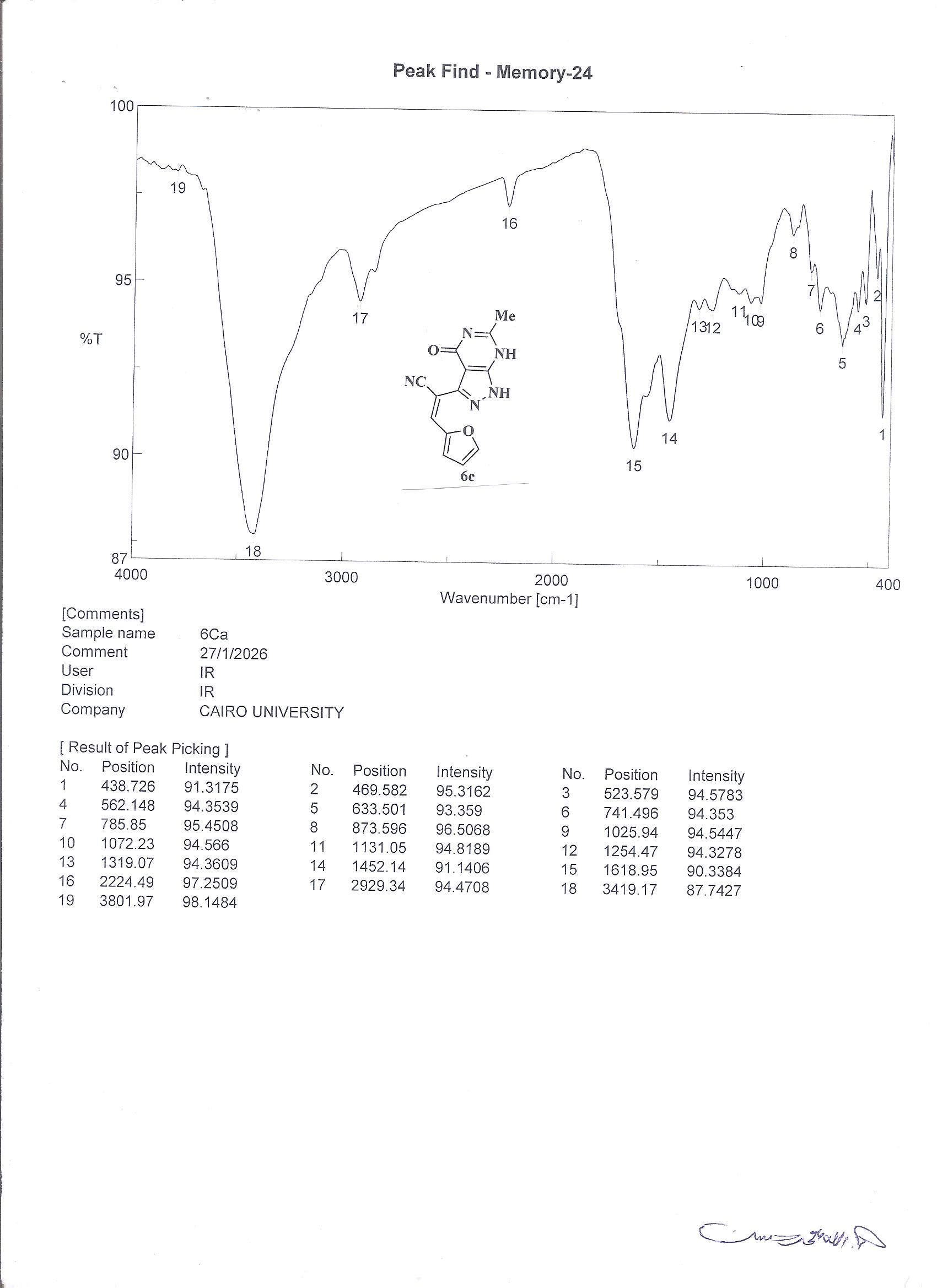


**S12. IR of compound 6c**


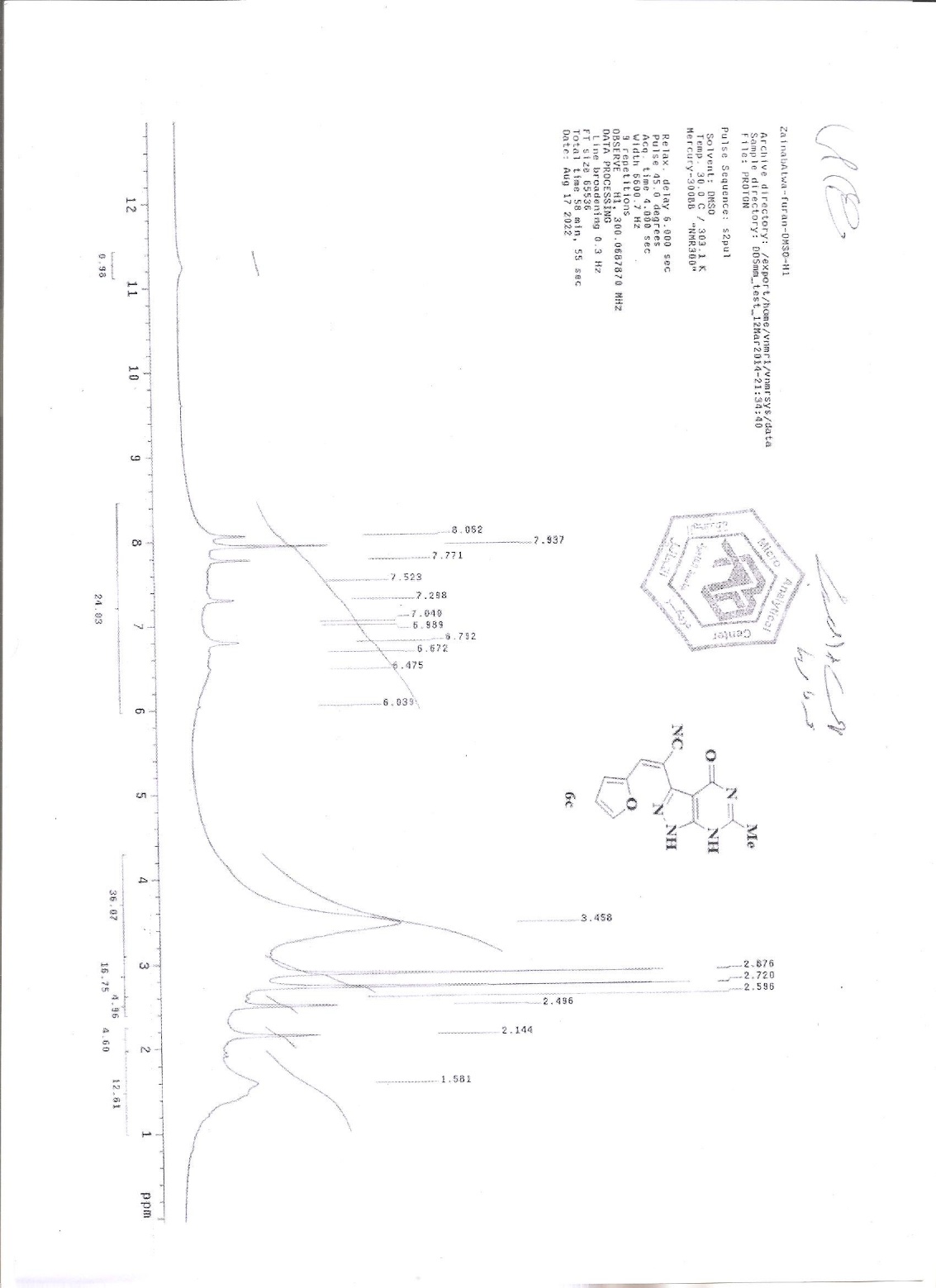


**S13. ^1^H NMR of compound 6c**

**S14. MS of compound 6c**


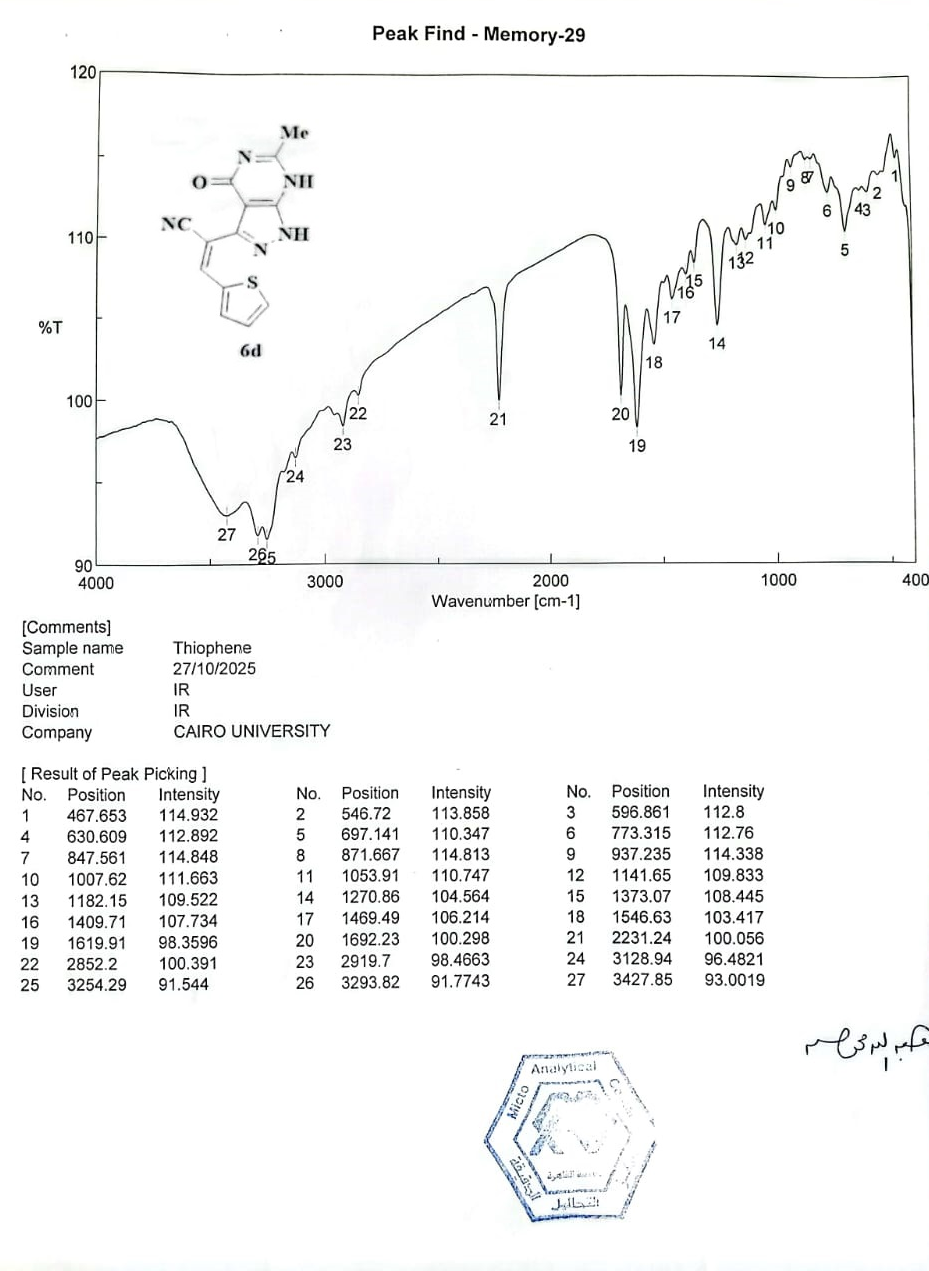


**S15. IR of compound 6d**

**
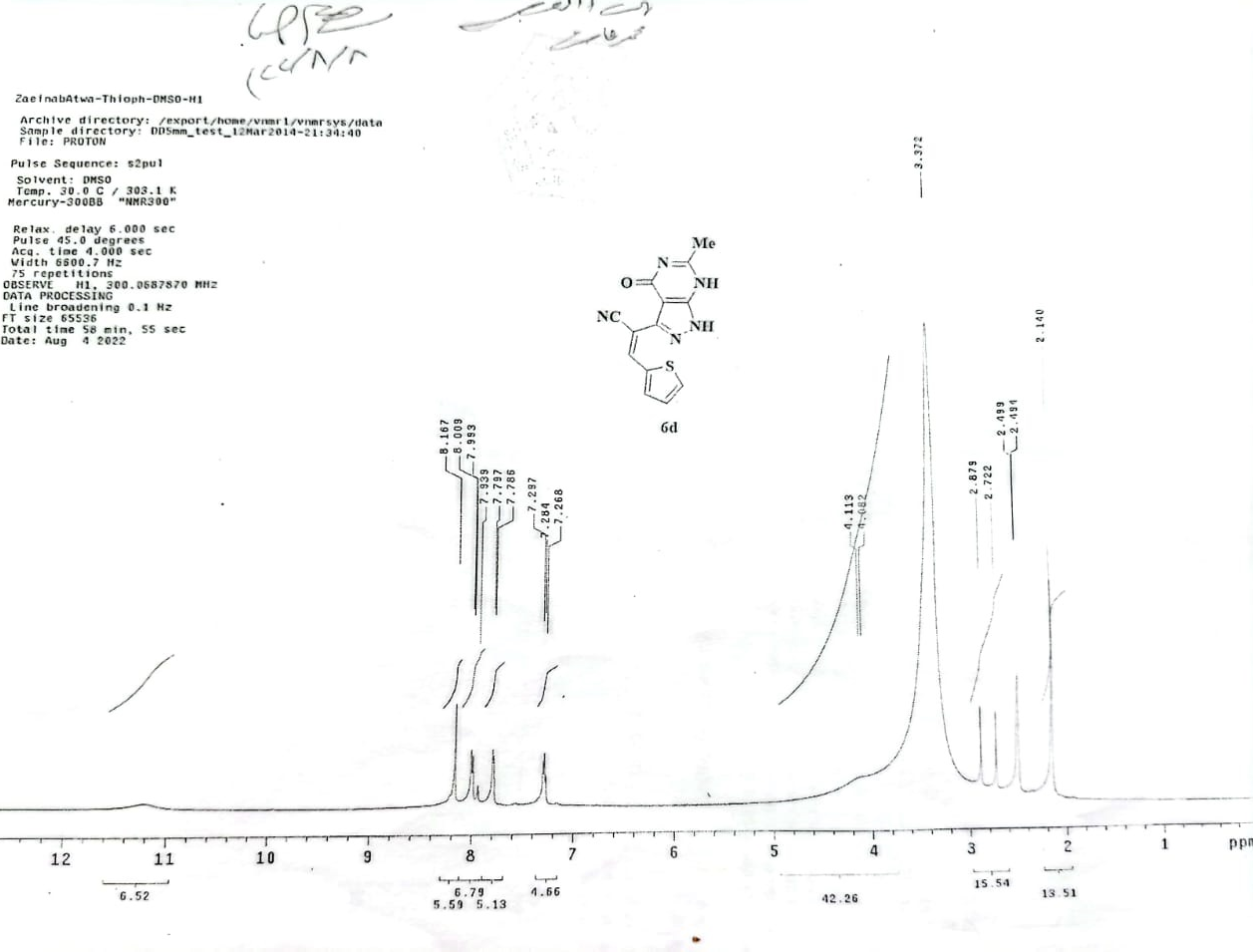
**

**S16. ^1^H NMR of compound 6d**


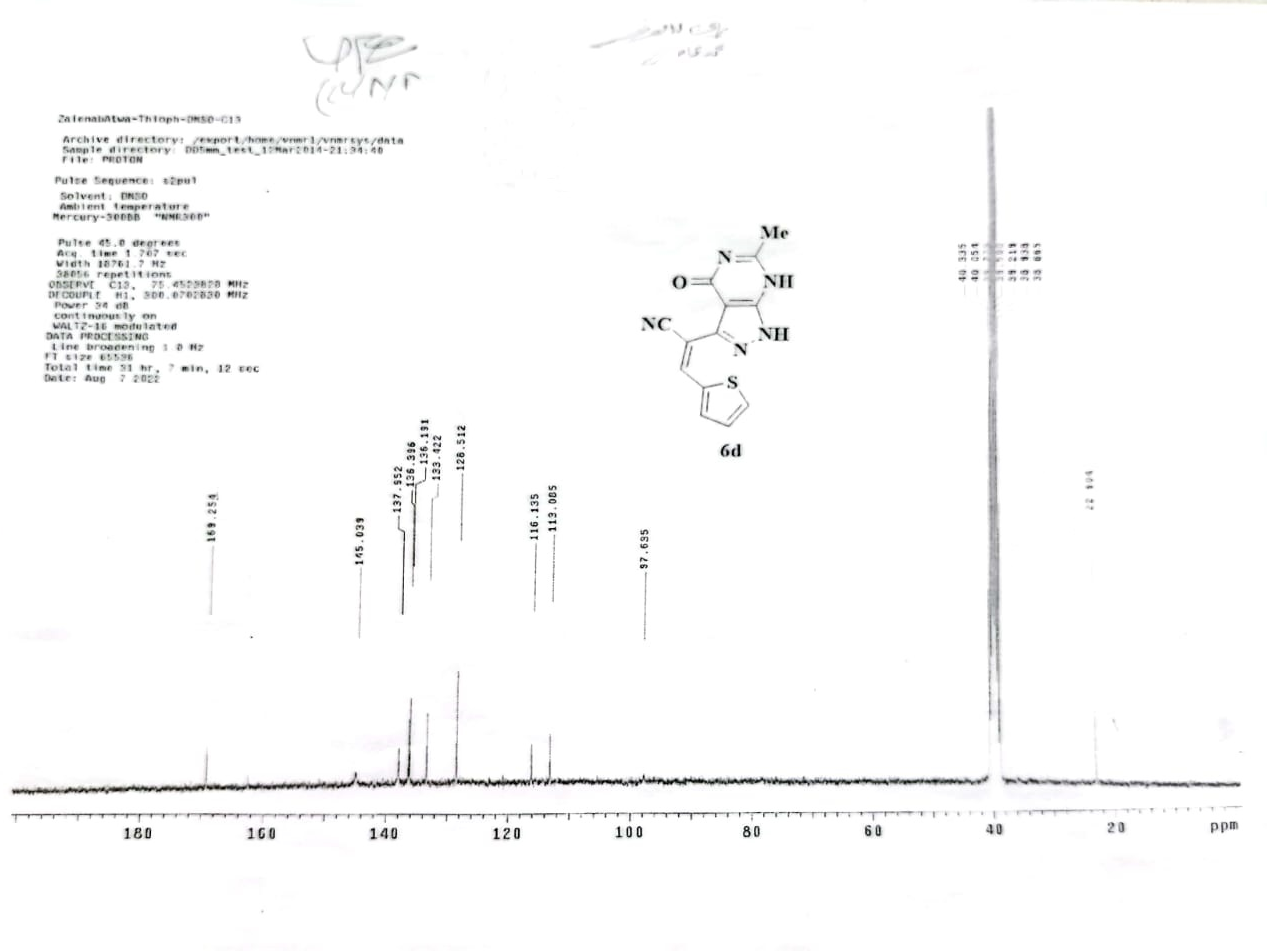


**S17. ^13^C NMR of compound 6d**


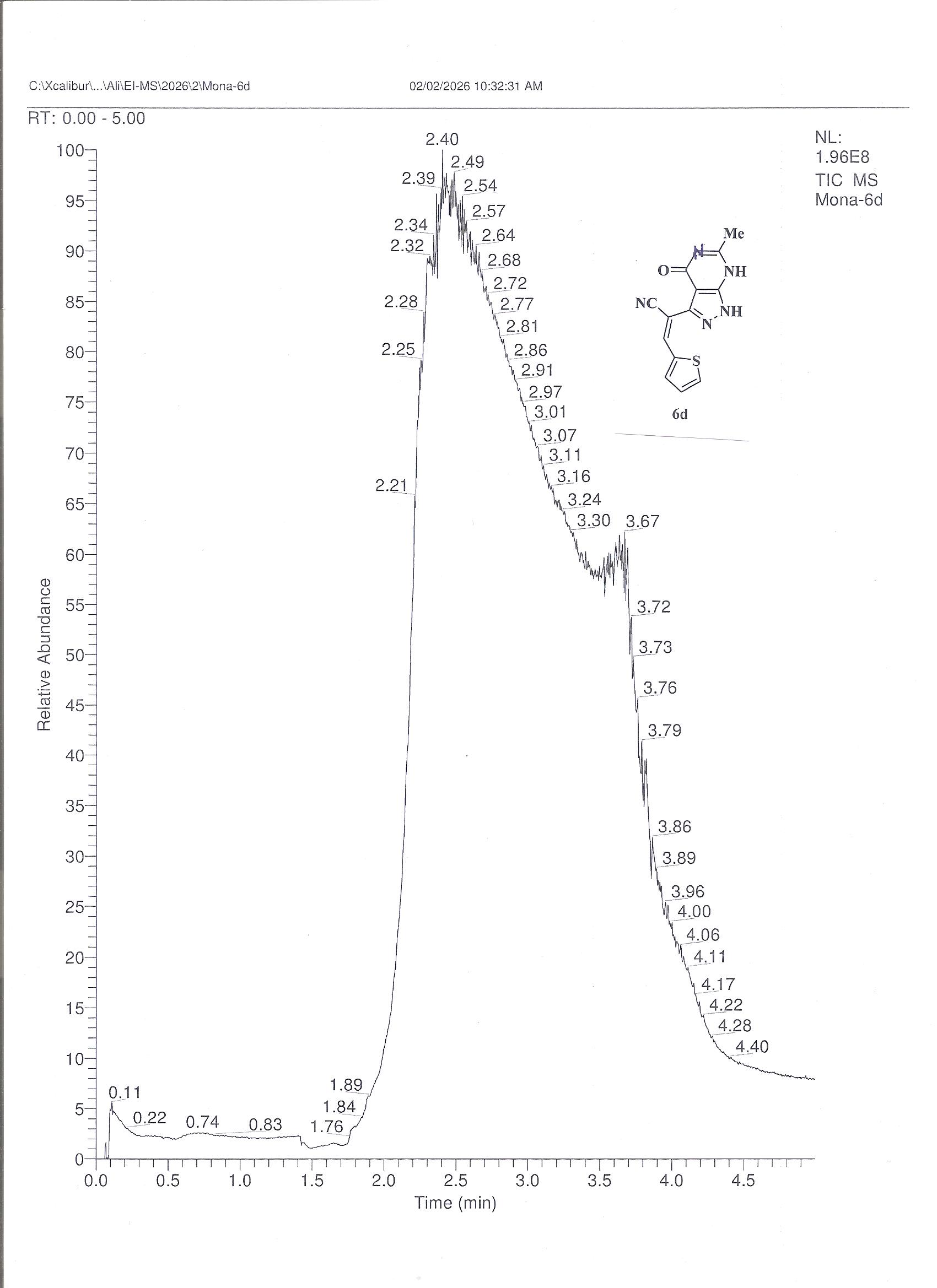


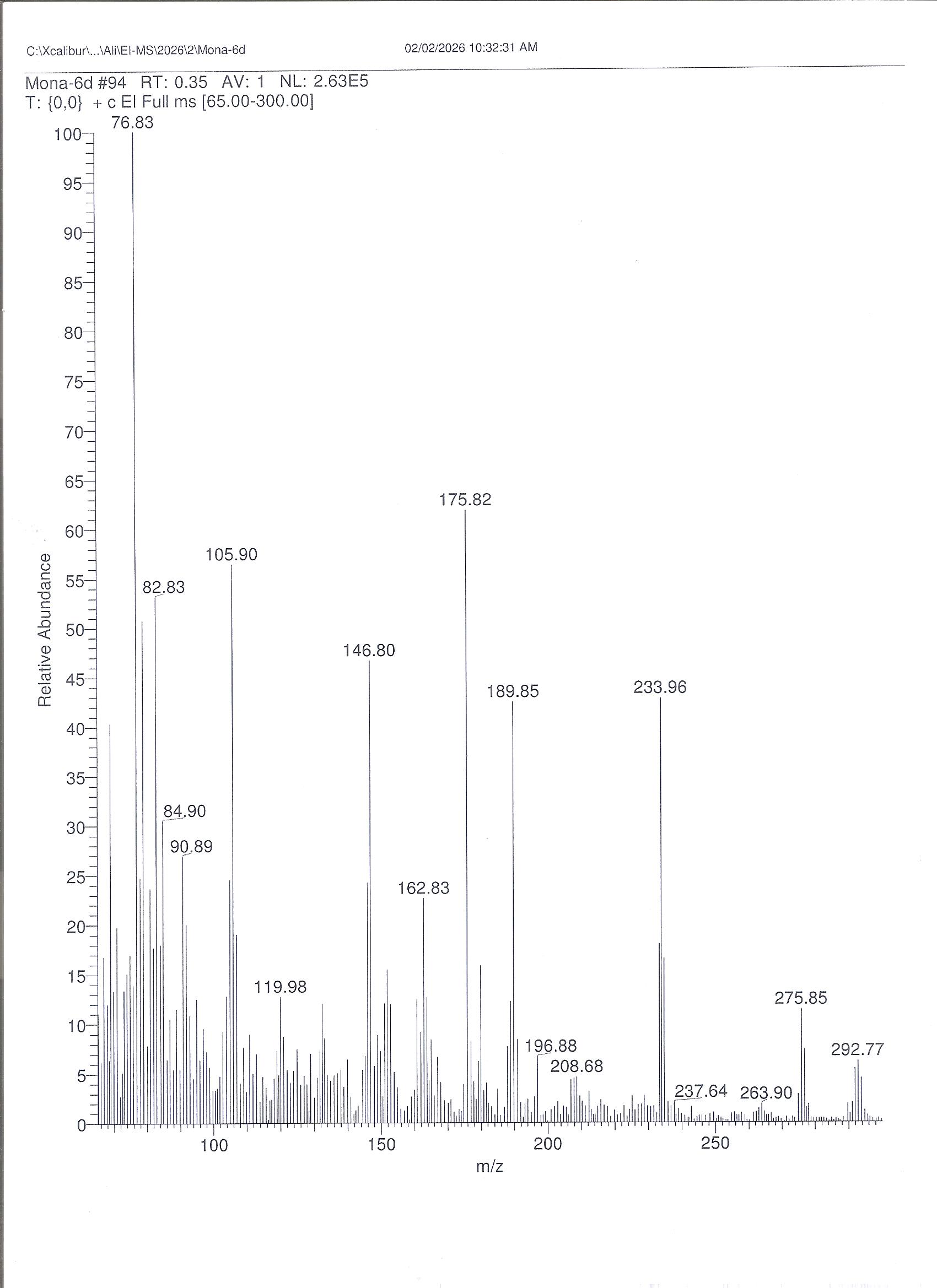


**S18. MS of compound 6d**


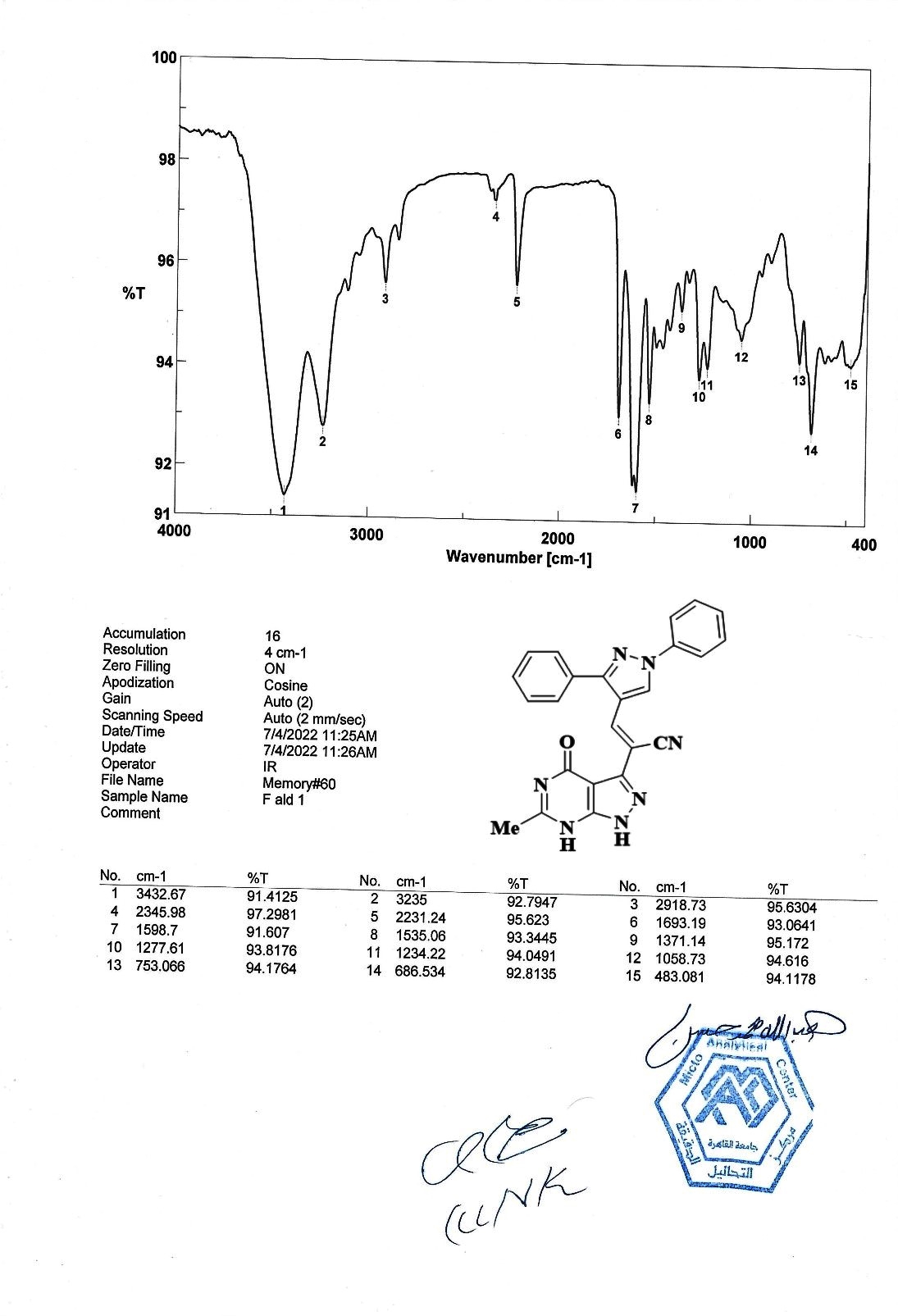


**S19. IR of compound 6e**


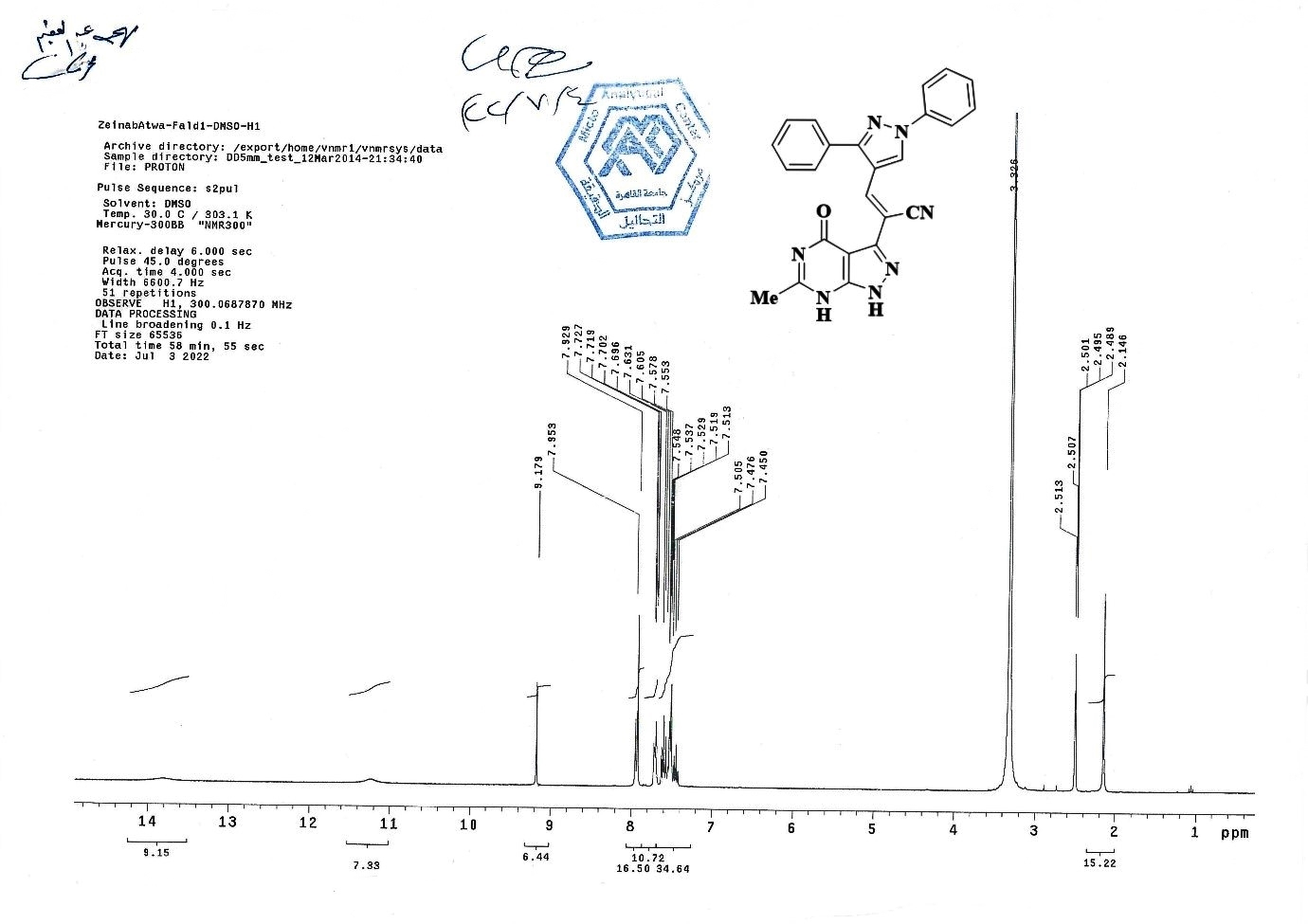


**S20. ^1^H NMR of 6e**


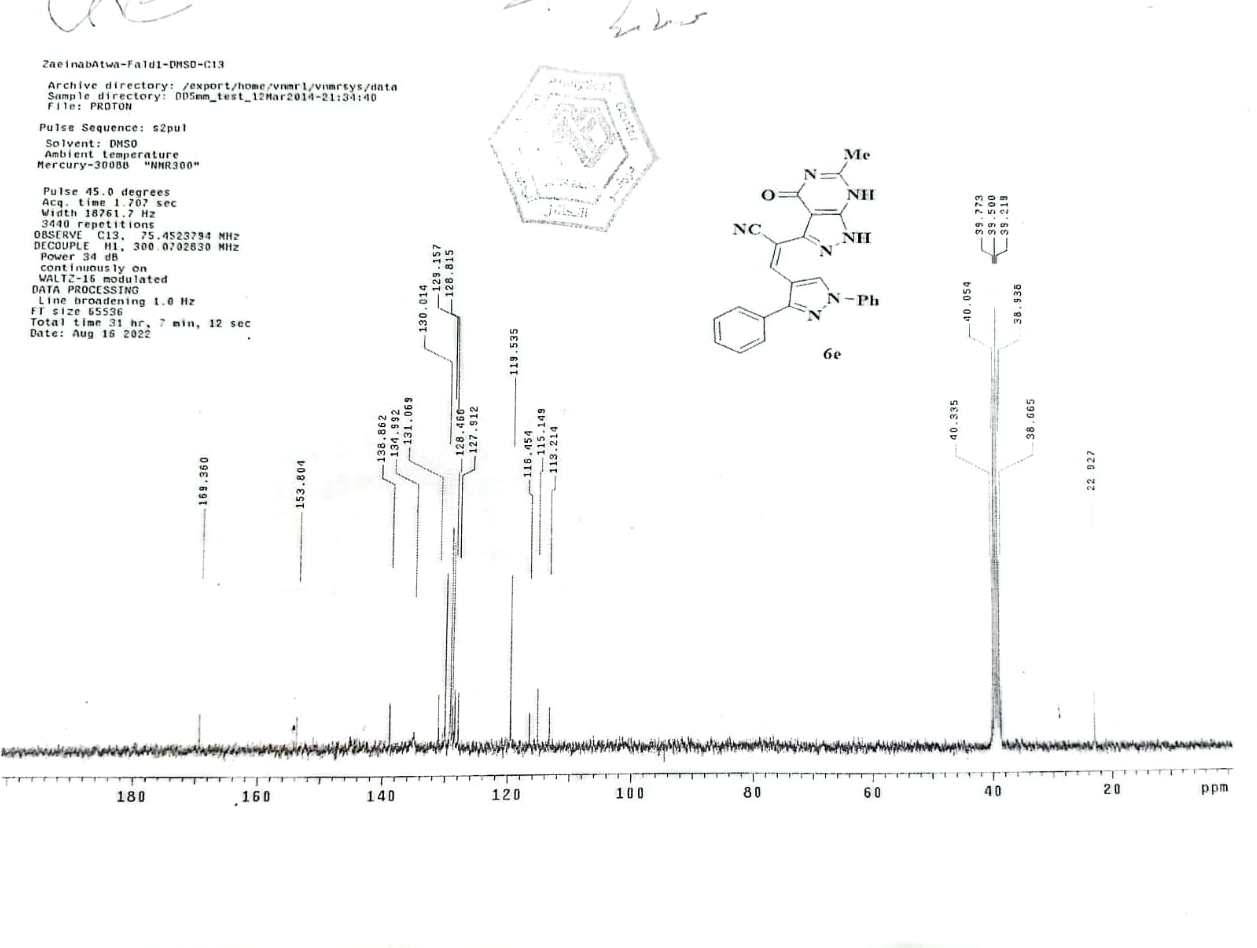


**S21. ^13^C NMR of 6e**


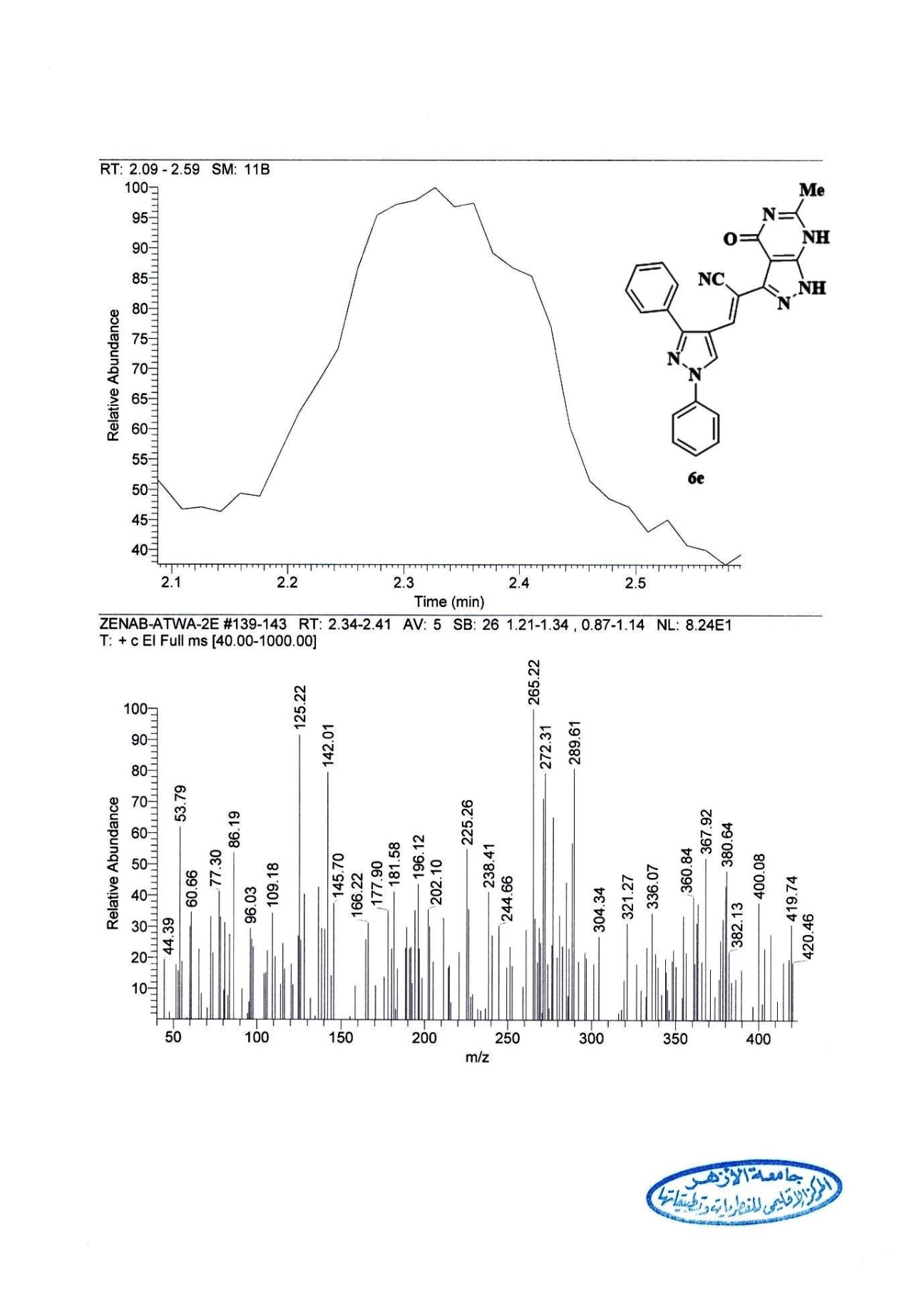


**S22. MS of compound 6e**


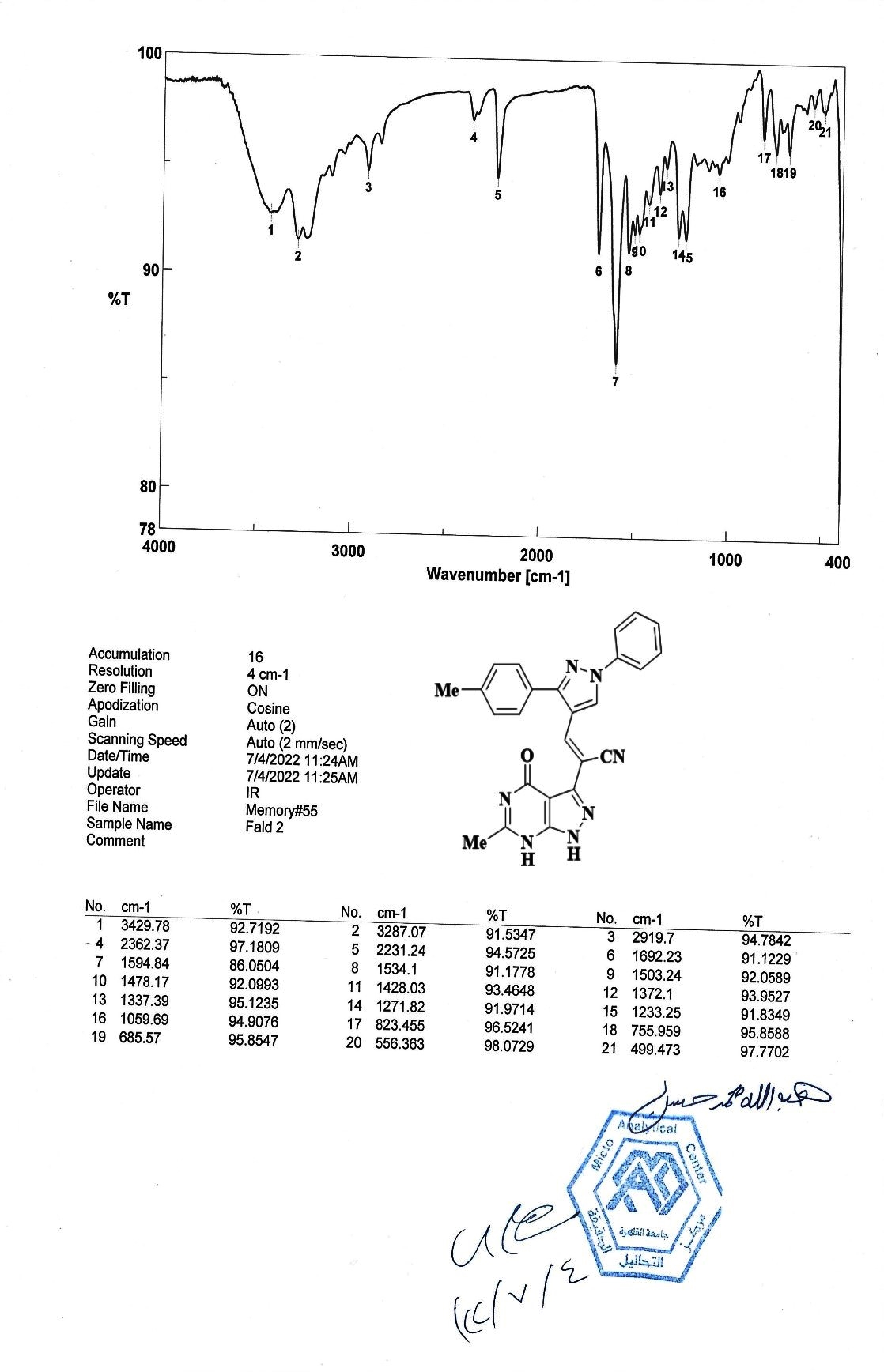


**S23. IR of compound 6f**


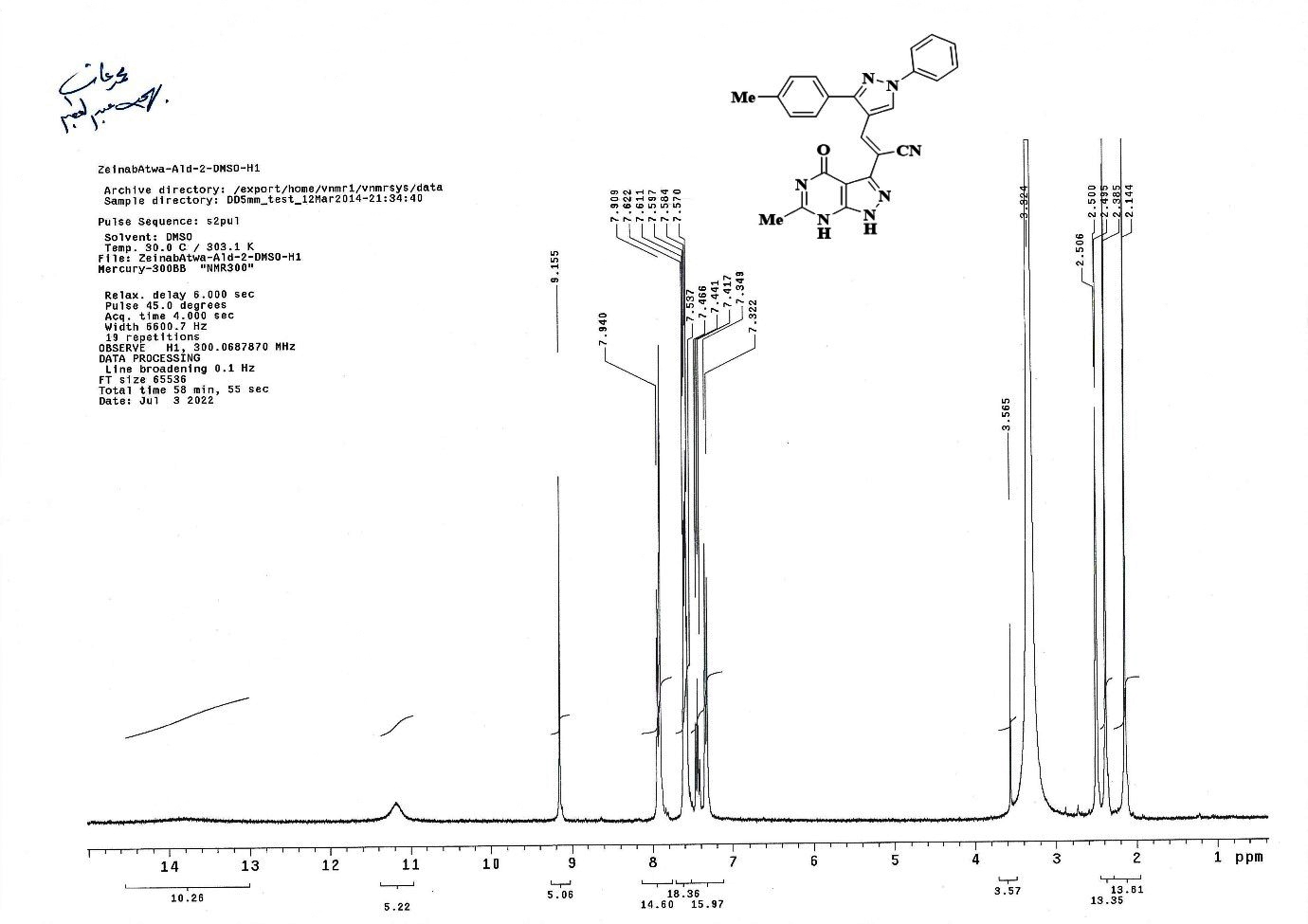


**S24. ^1^H NMR of compound 6f**


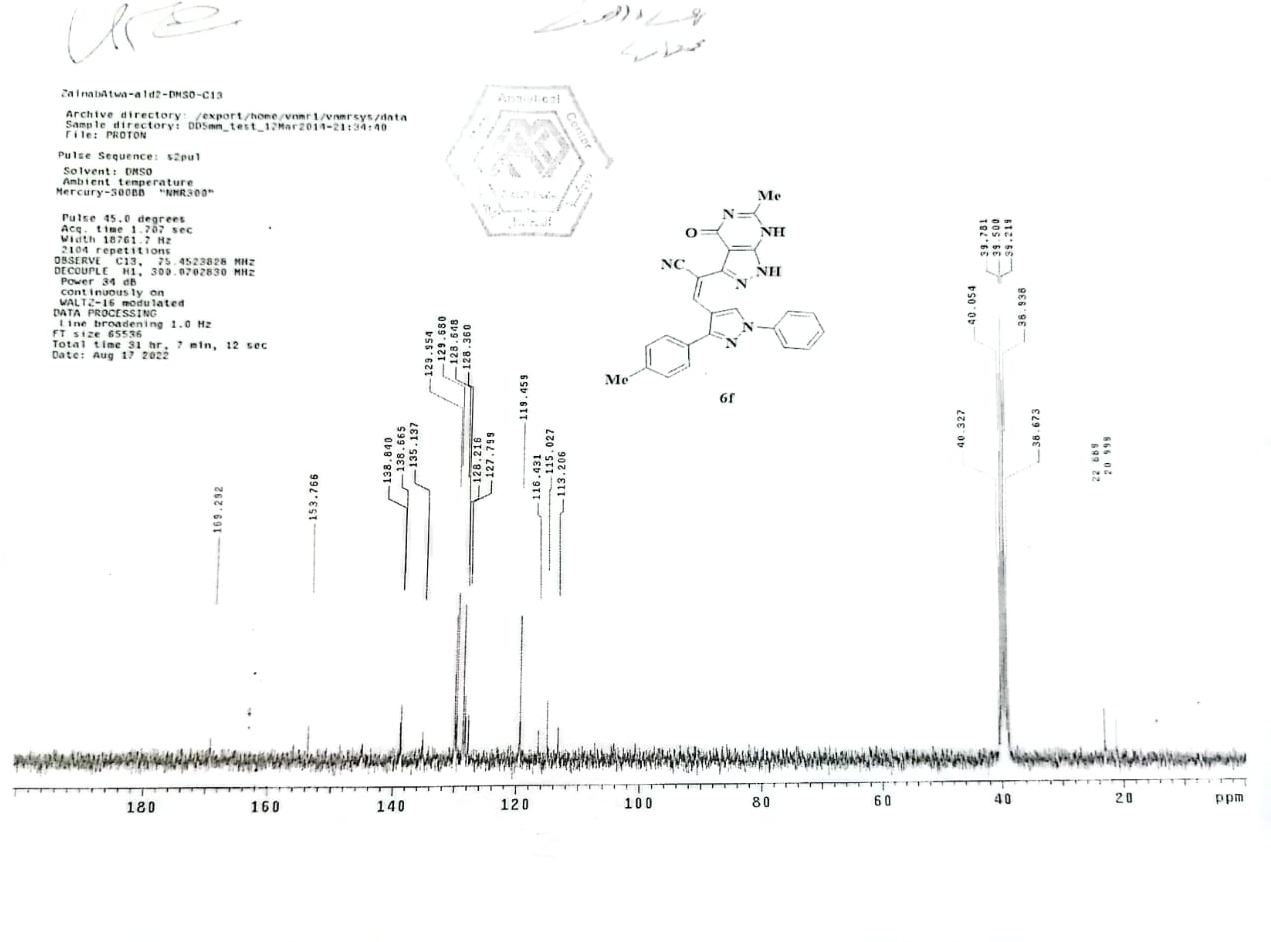


**S25. ^13^C NMR of compound 6f**


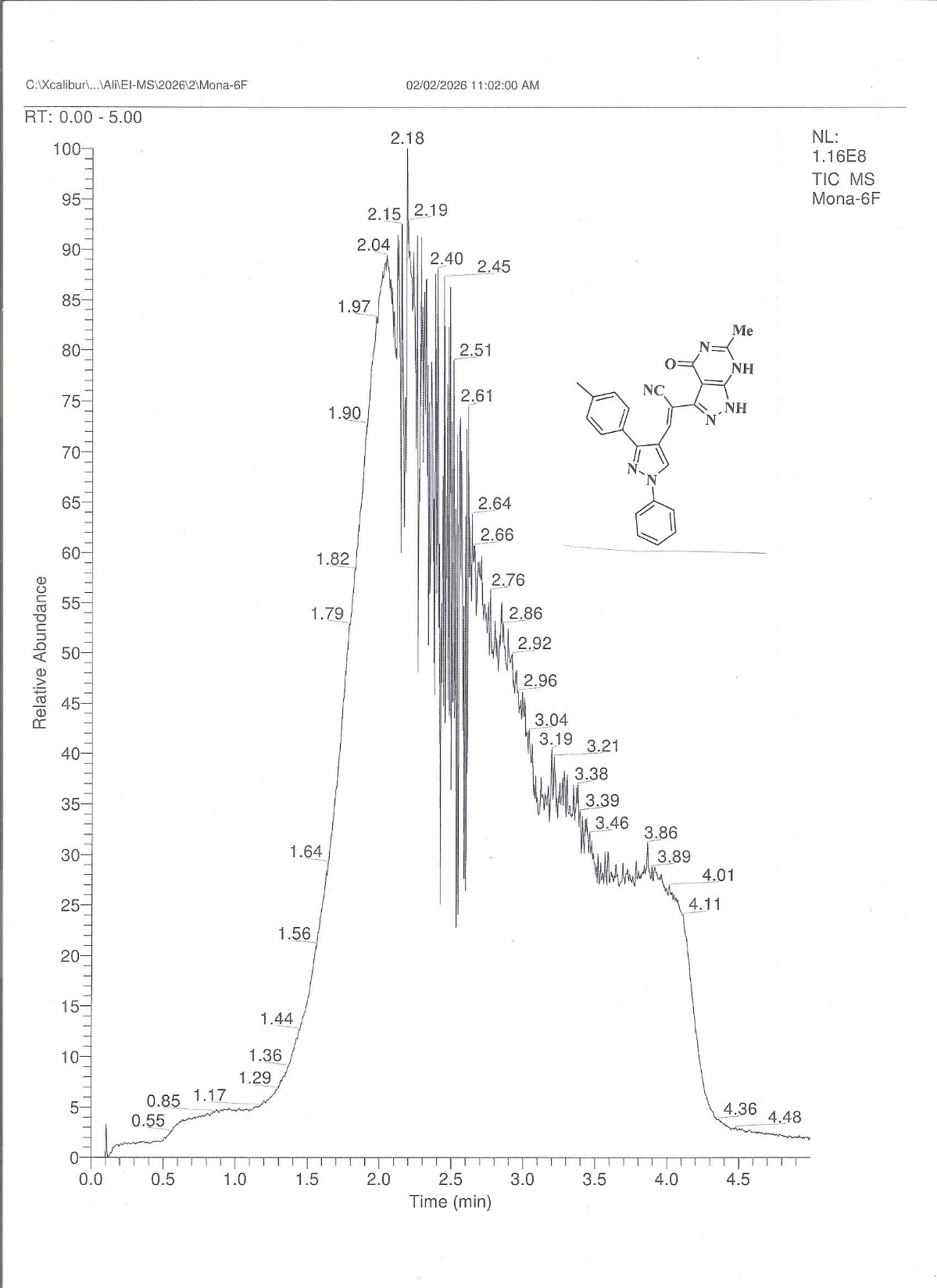


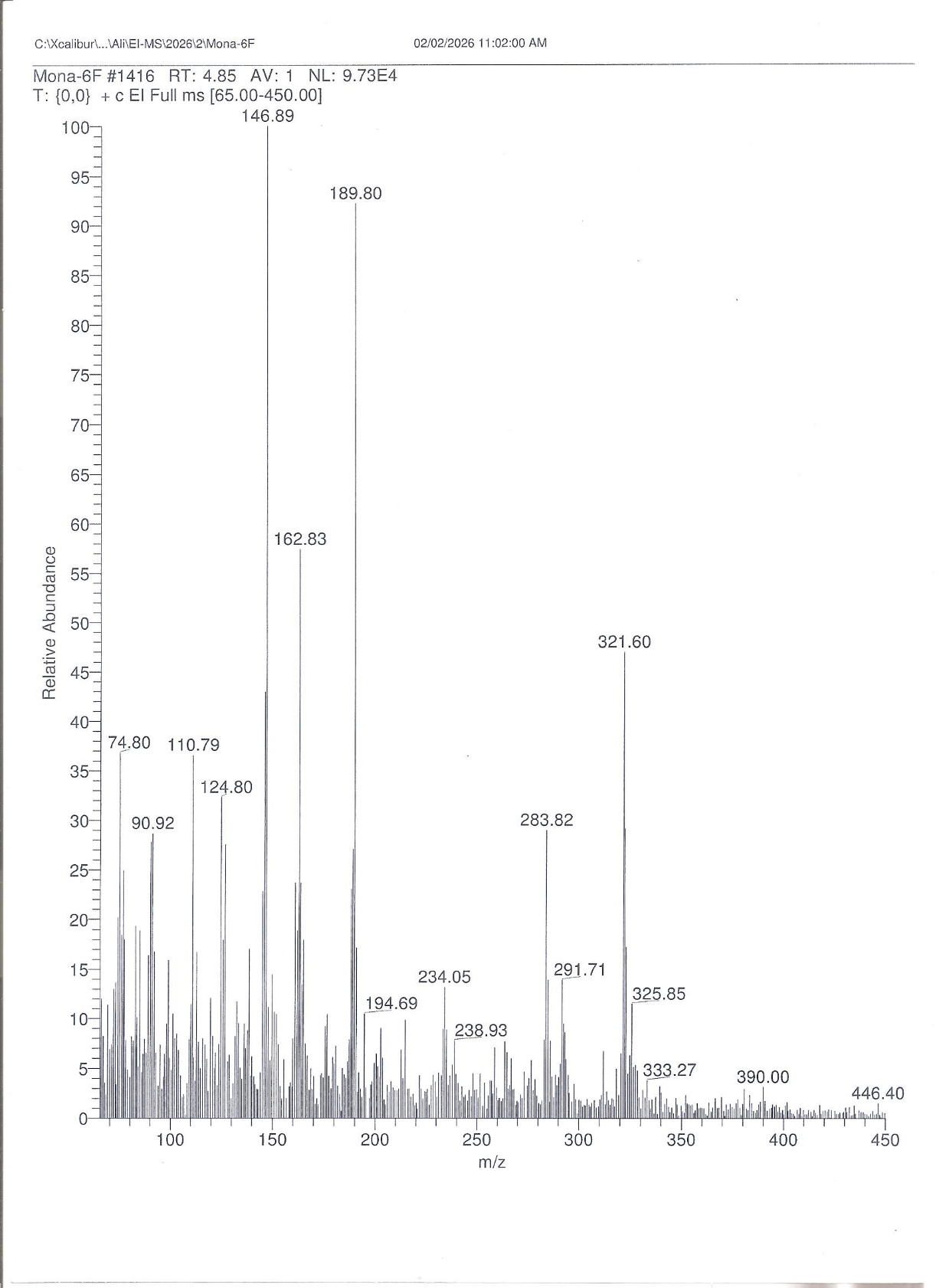


**S26. MS of compound 6f**


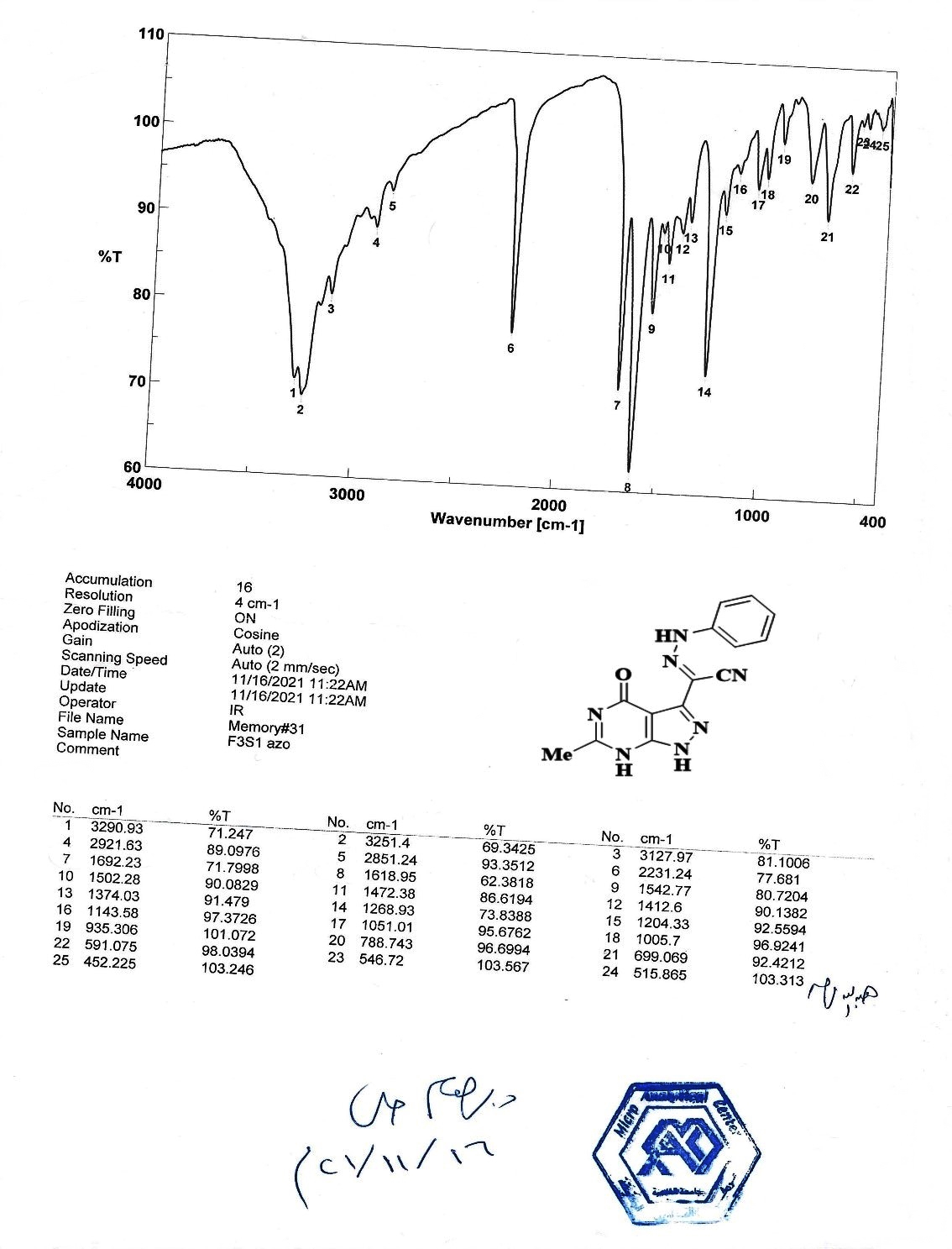


**S27. IR of 8a**


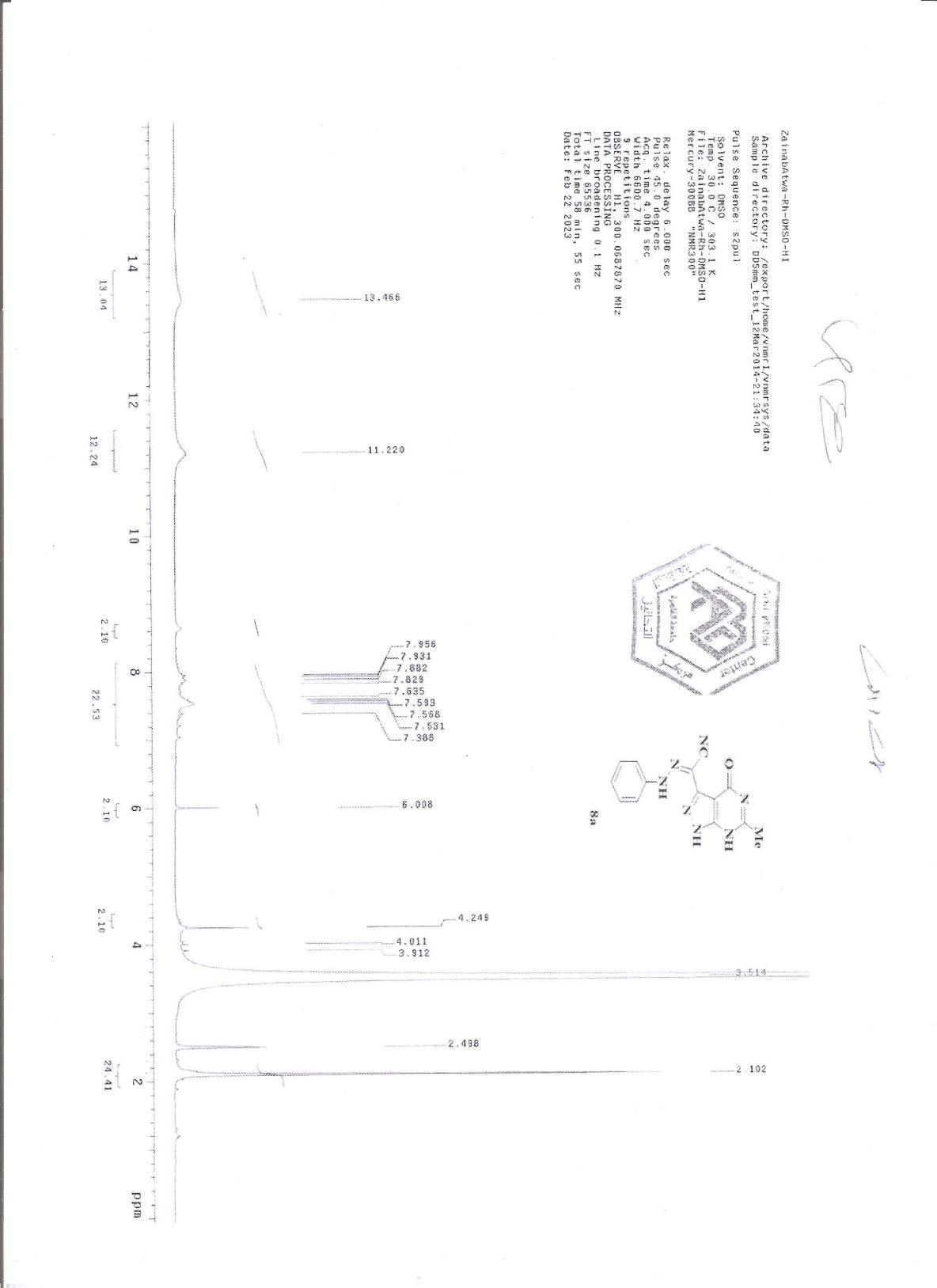


**S28. ^1^H NMR of compound 8a**


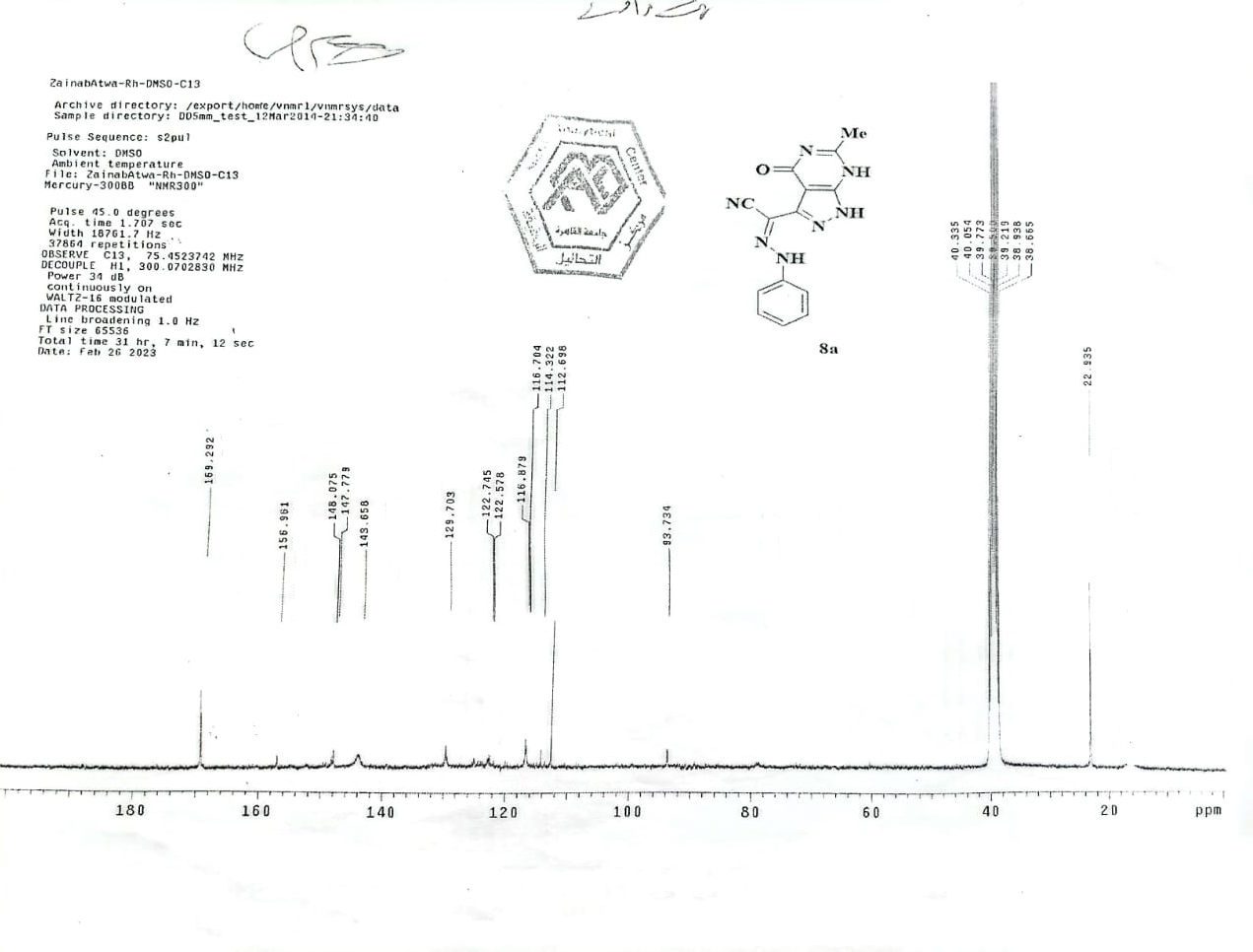


**S29. ^13^C NMR of compound 8a**


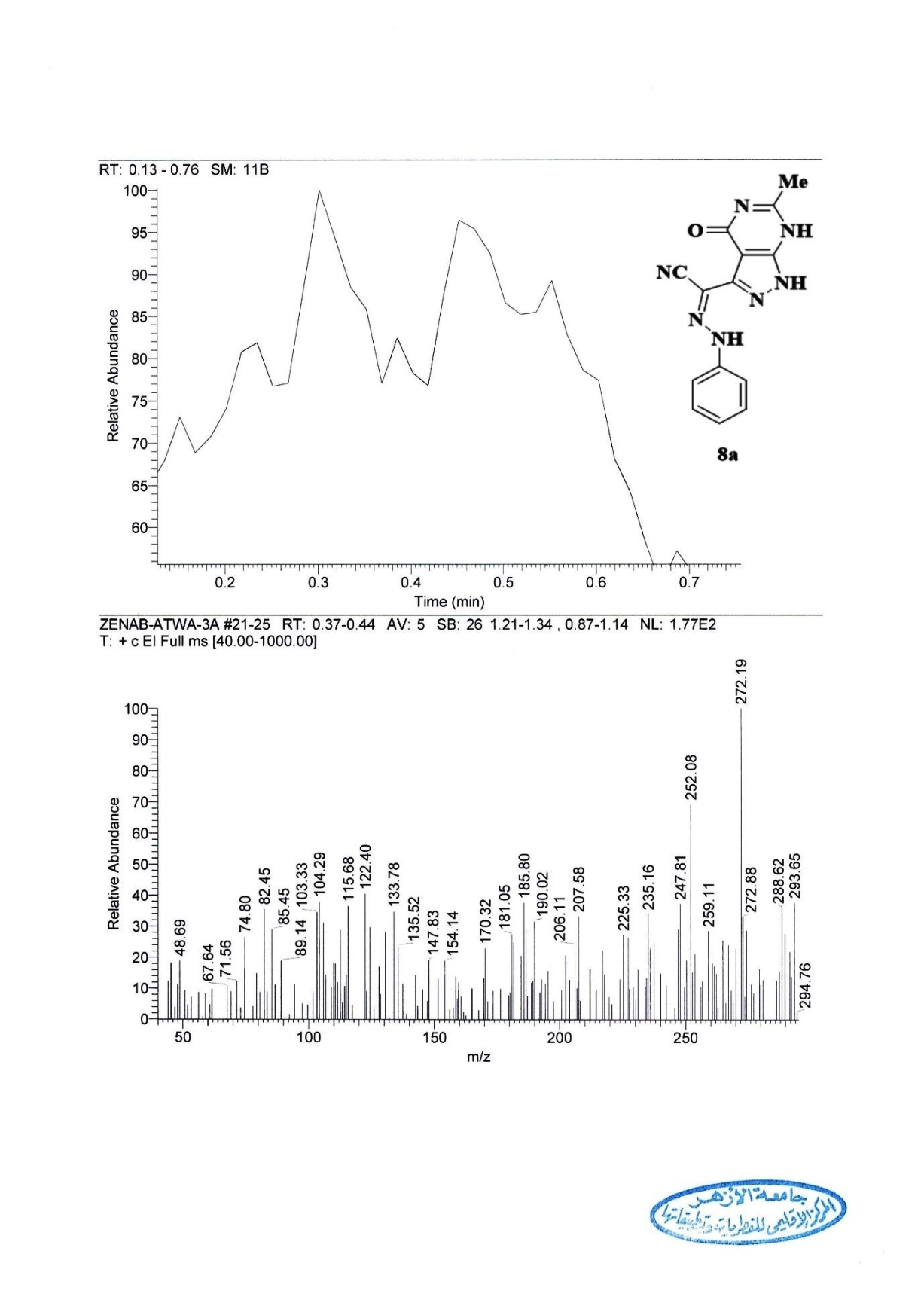


**S30. MS of compound 8a**


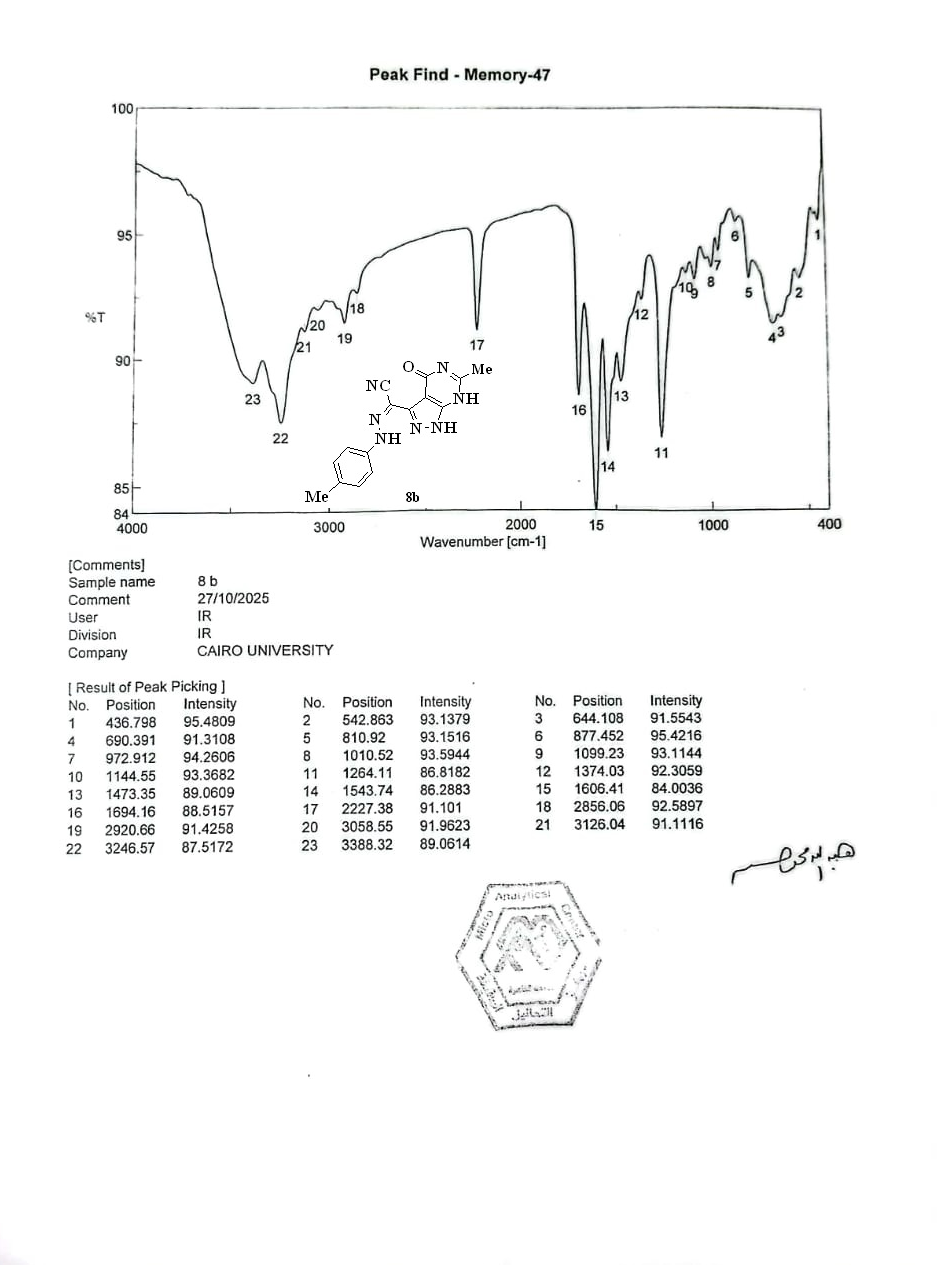


**S31. IR of 8b**


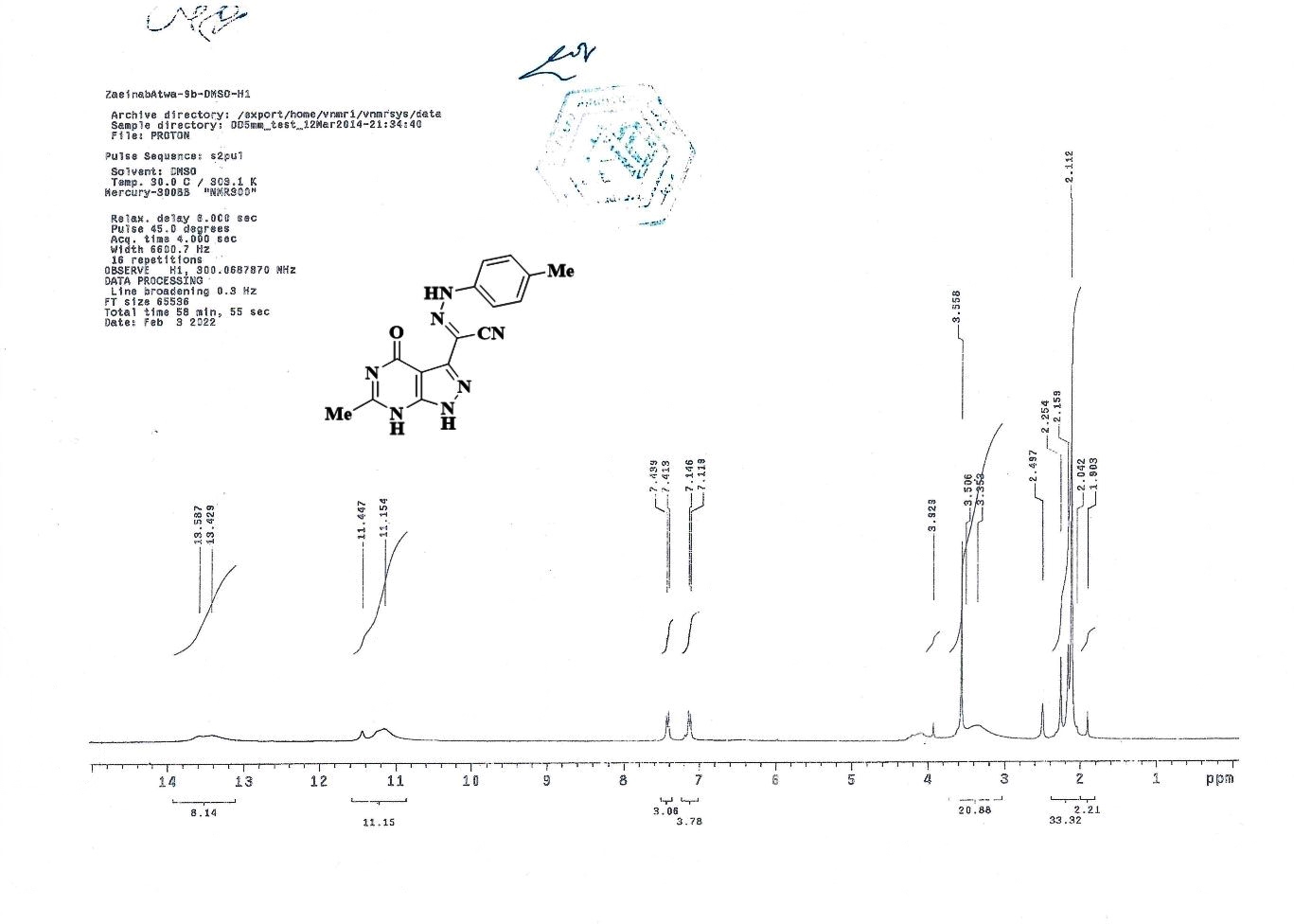


**S32. ^1^H NMR of compound 8b**


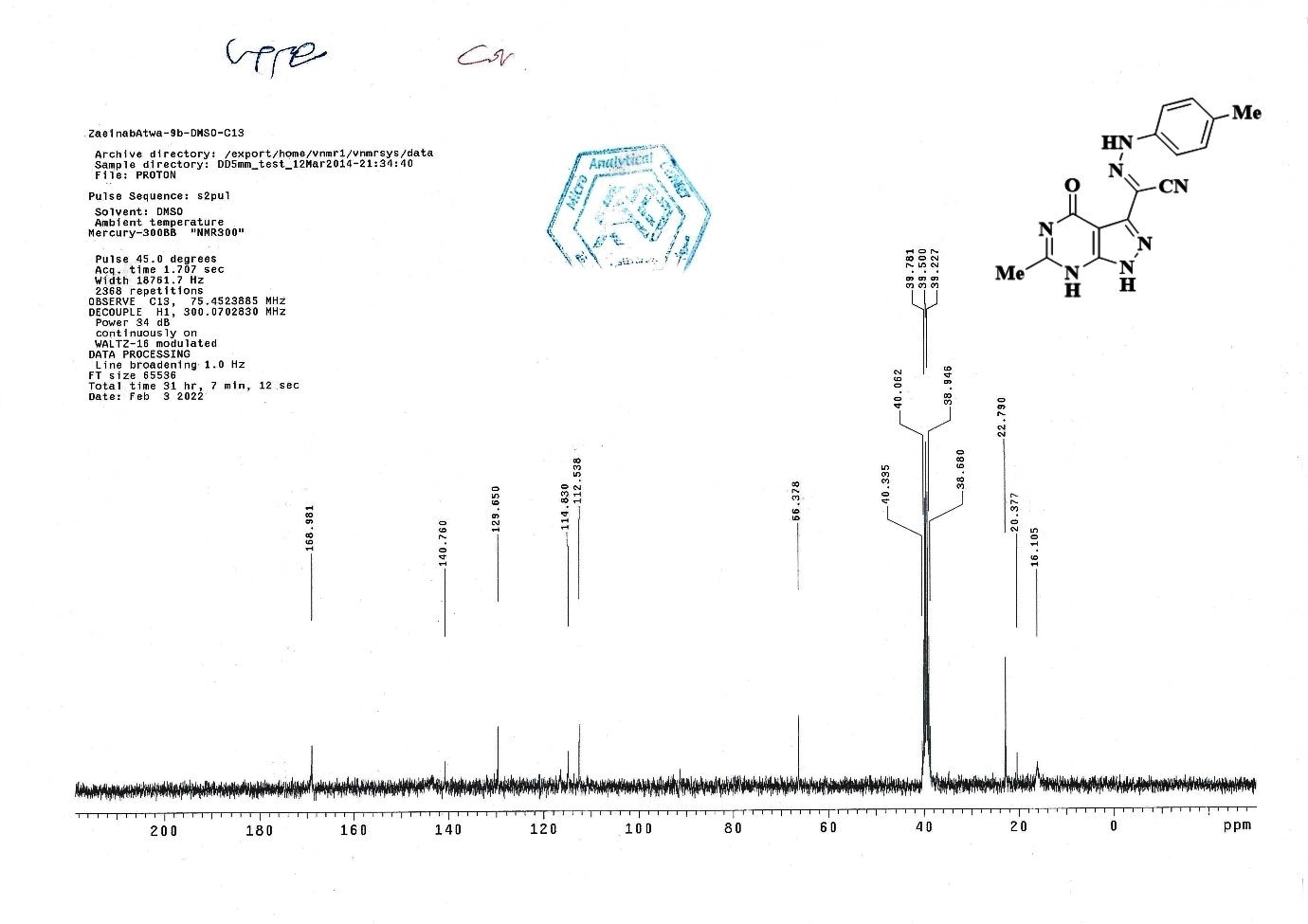


**S33. ^13^C NMR of compound 8b**


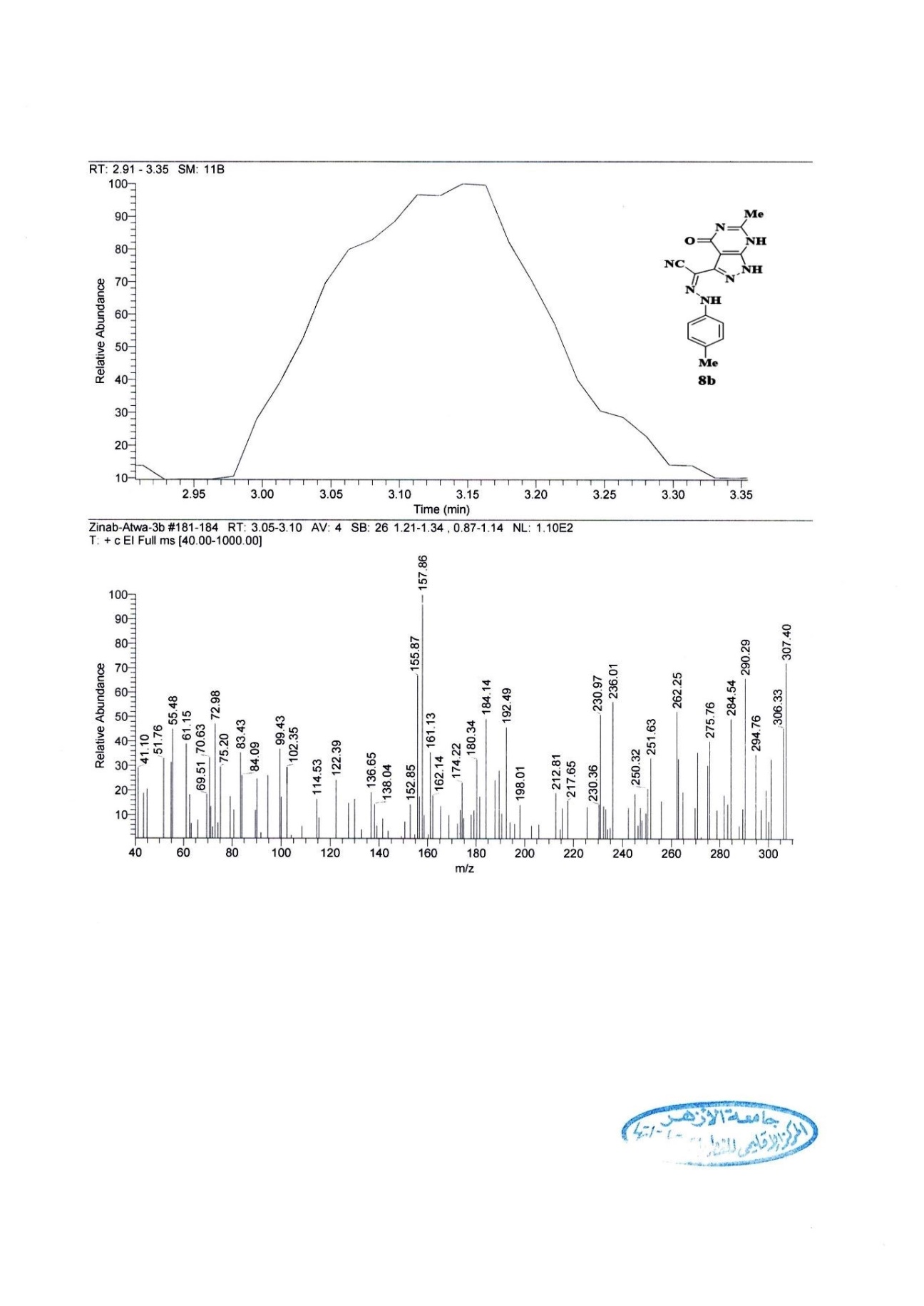


**S34. MS of compound 8b**


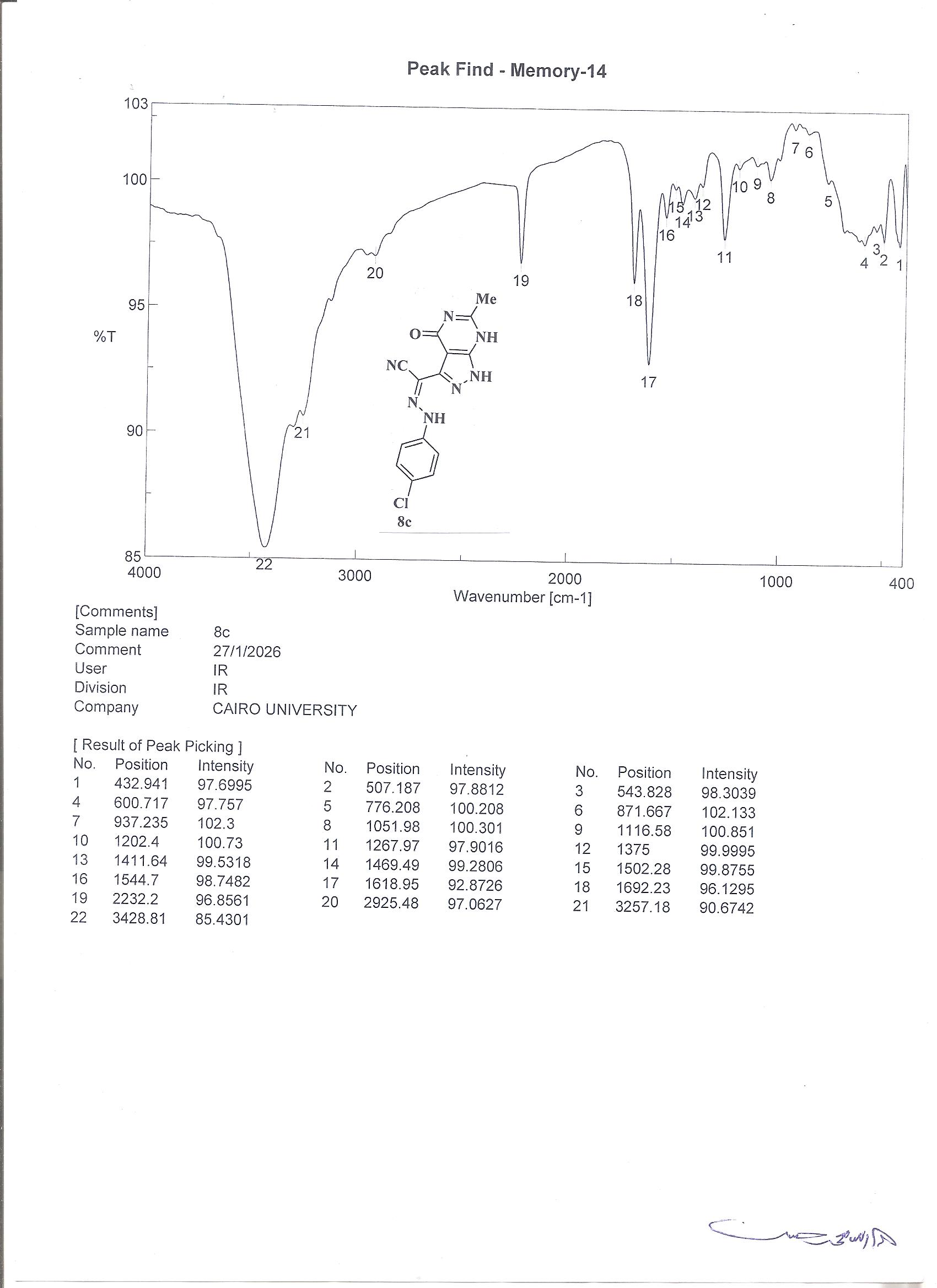


**S35. IR of compound 8c**


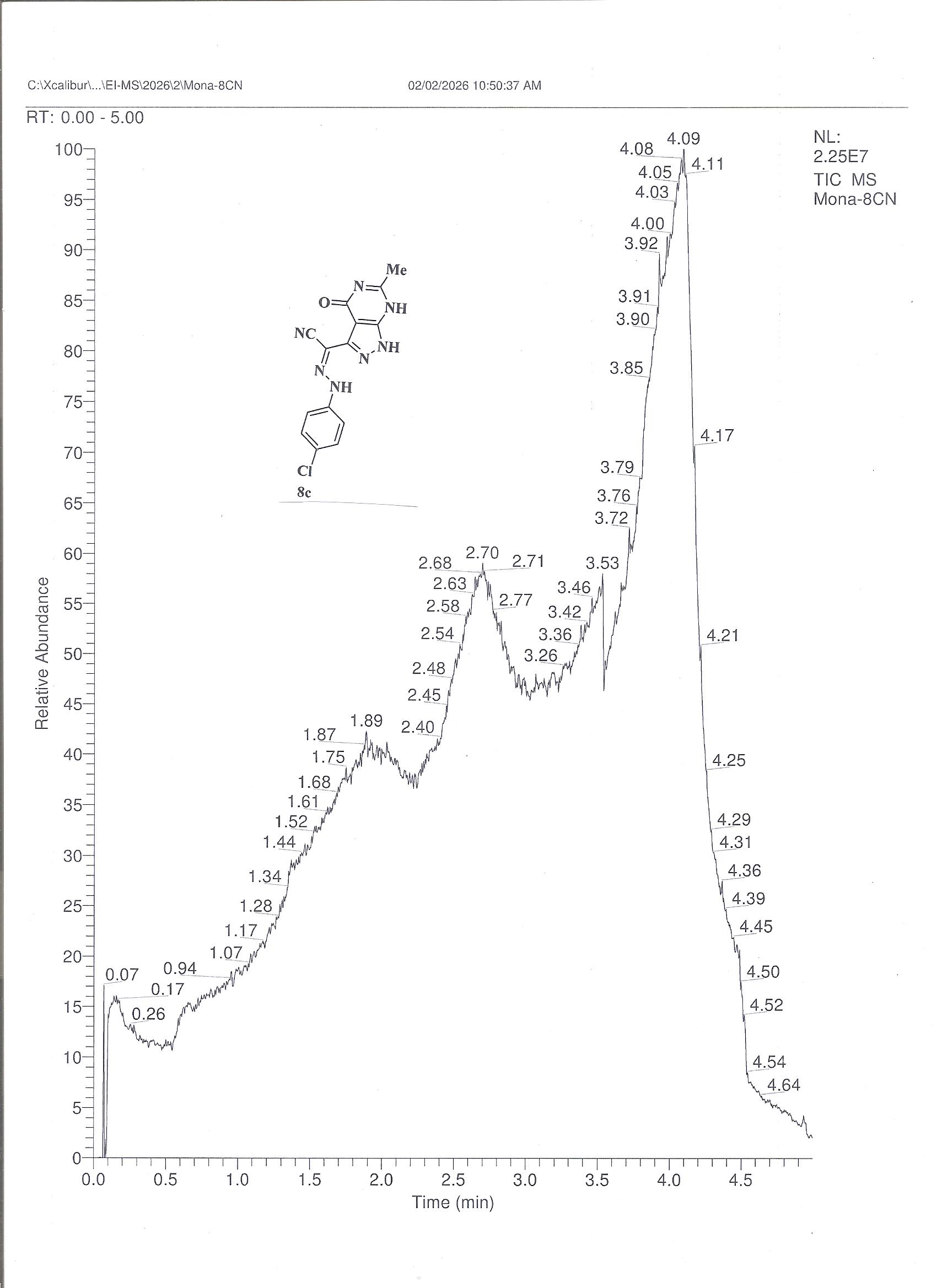


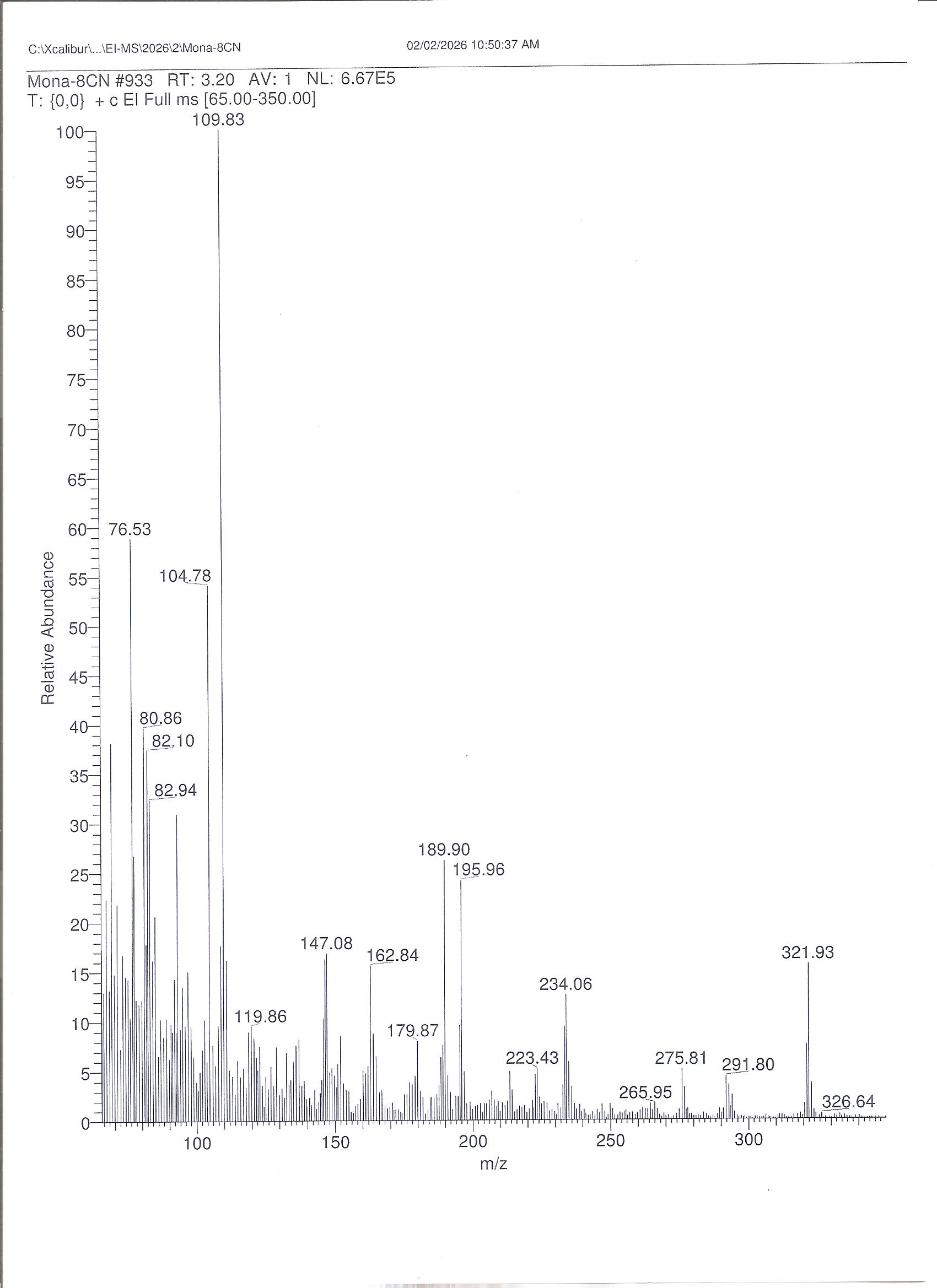


**S36. MS of compound 8c**


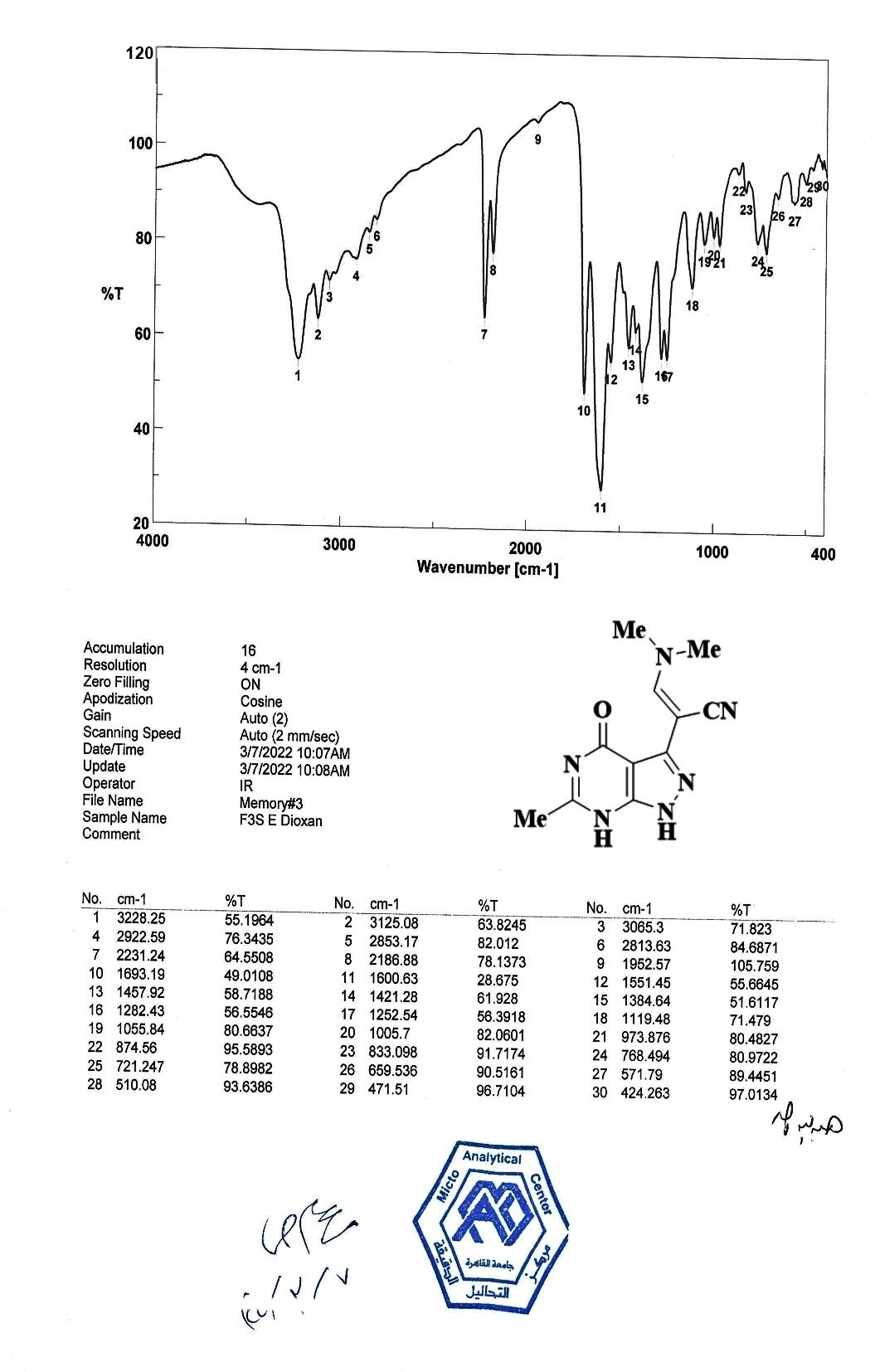


**S37. IR of compound 9**


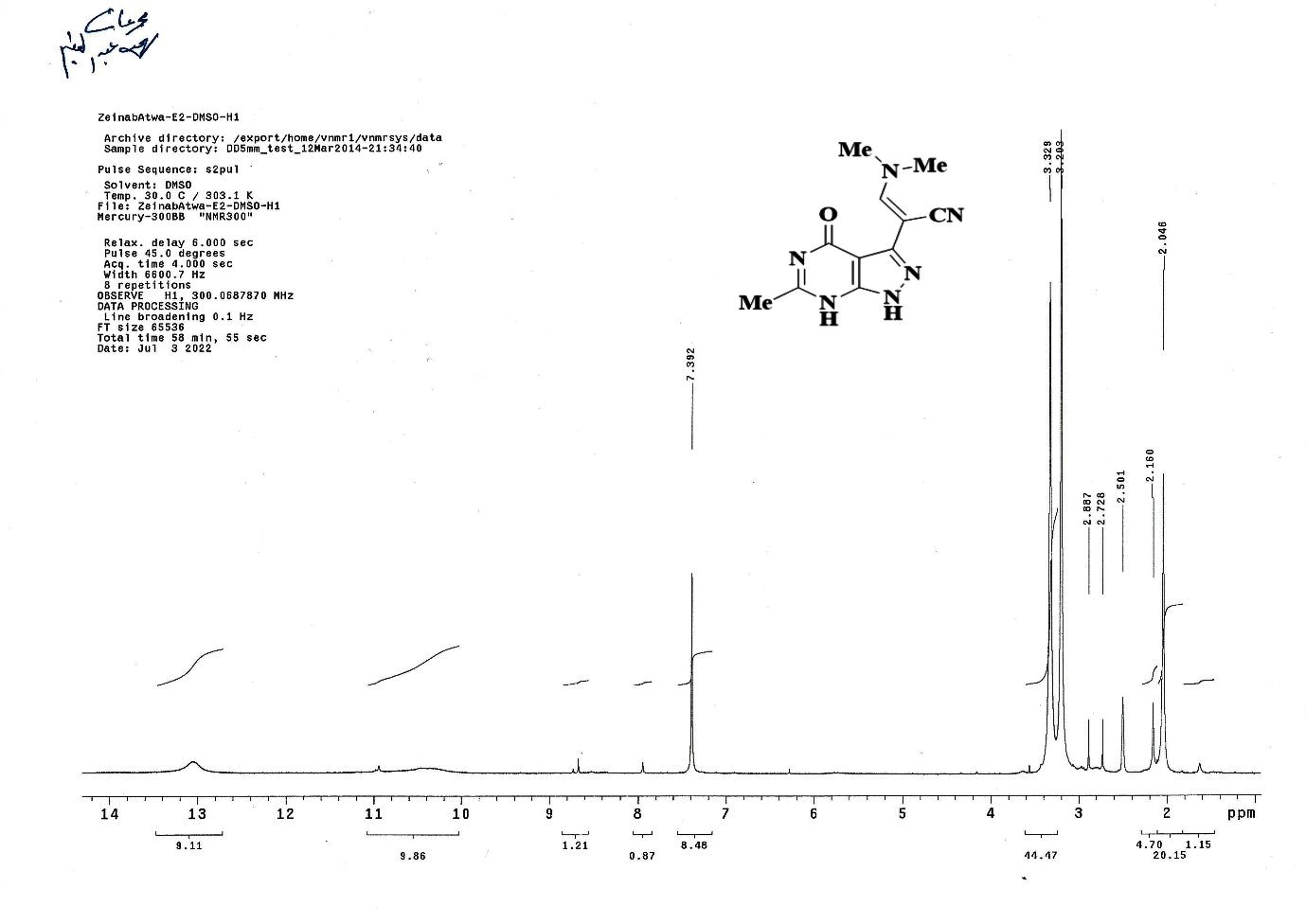


**S38. ^1^H NMR of 9**


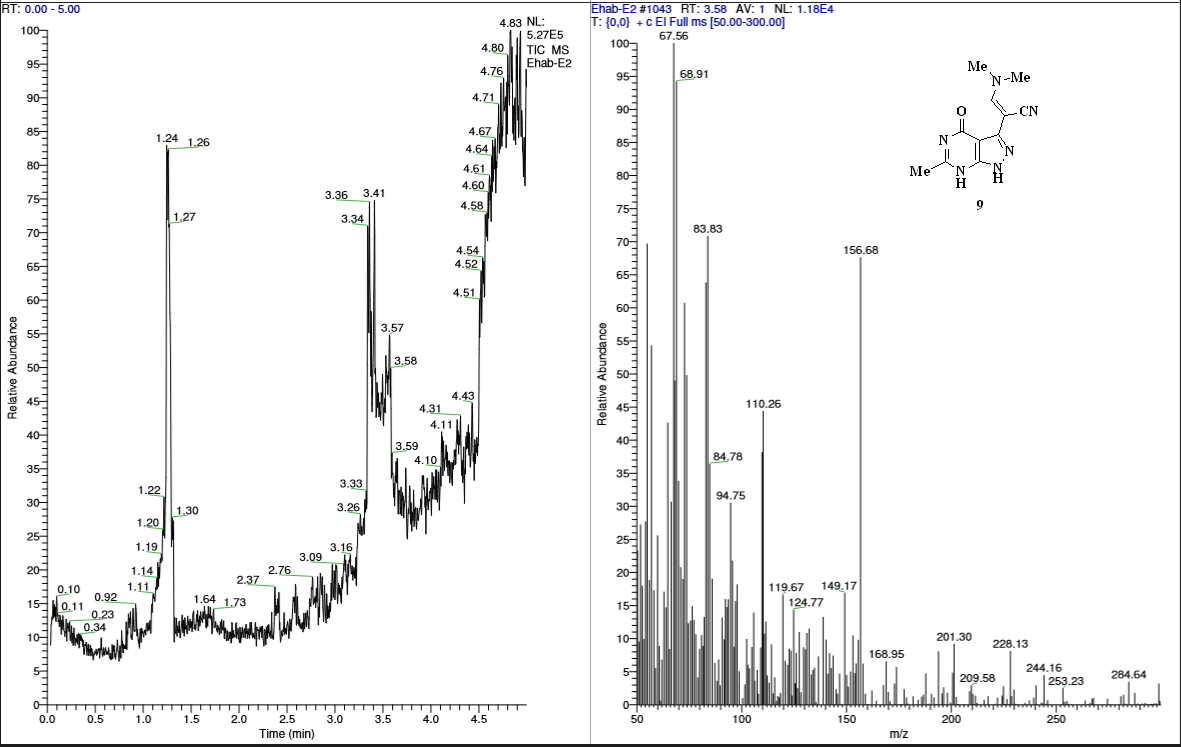


**S39. MS of compound 9**


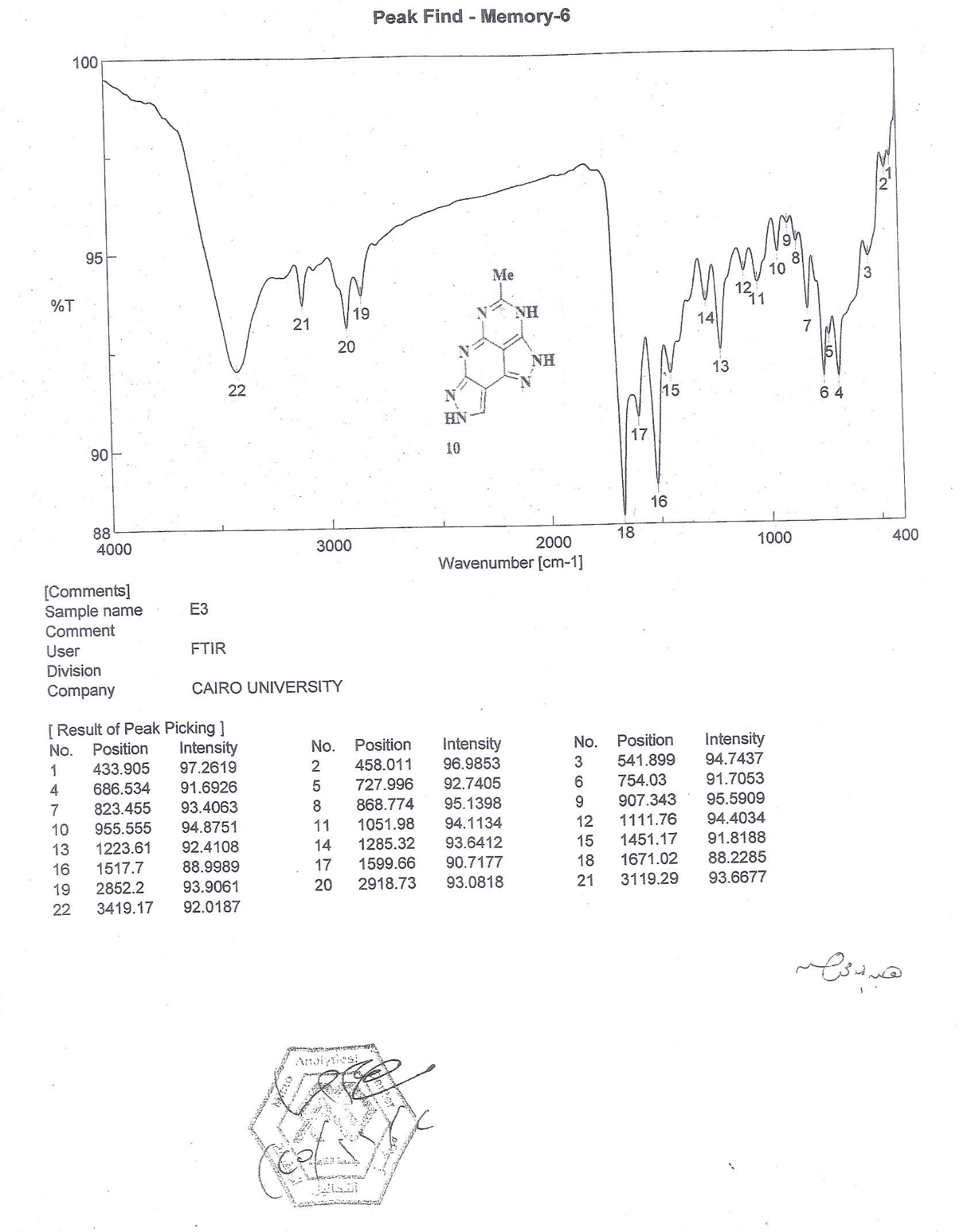


**S40. IR of Compound 10**


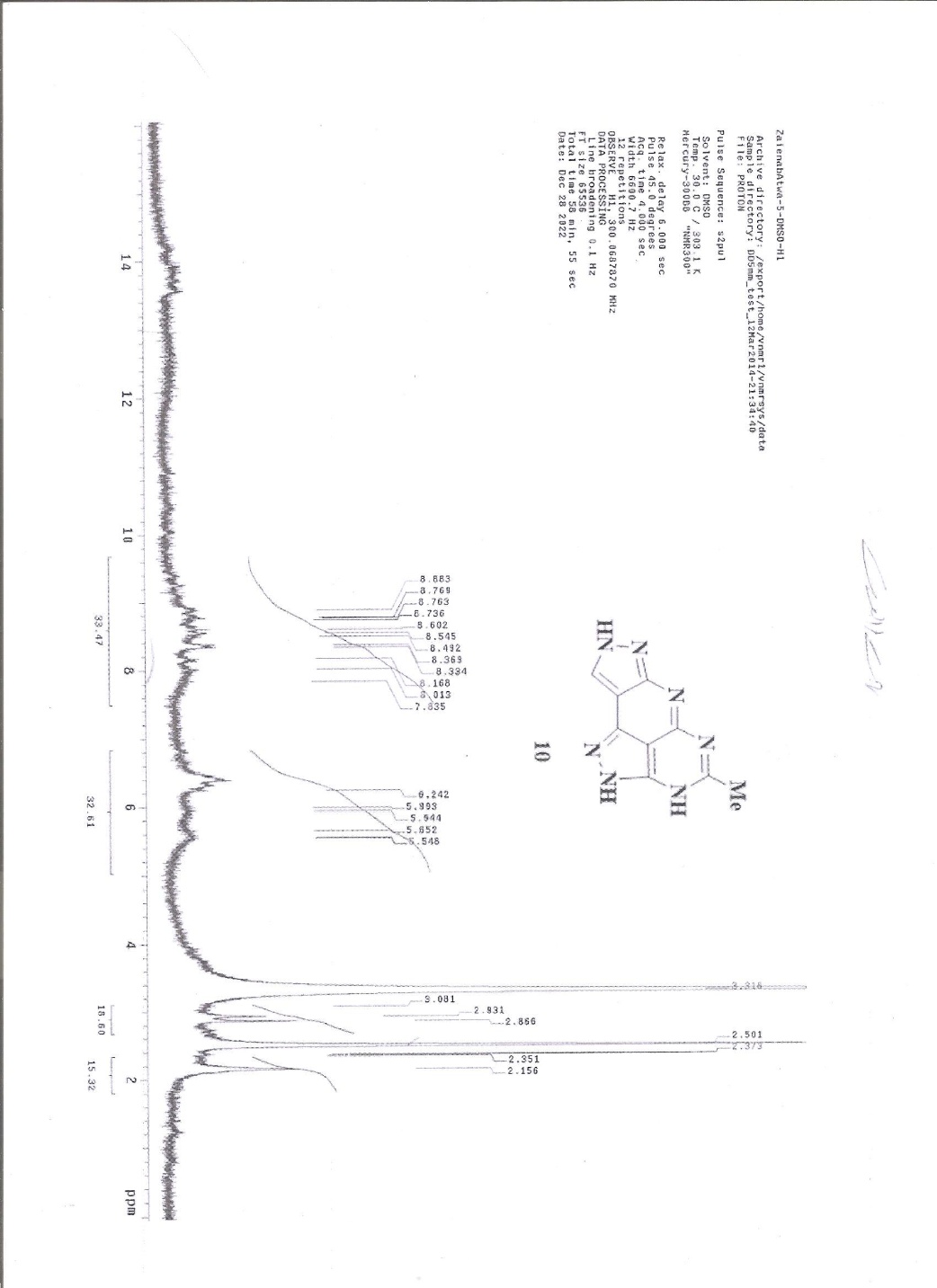


**S41. ^1^H NMR of Compound 10**


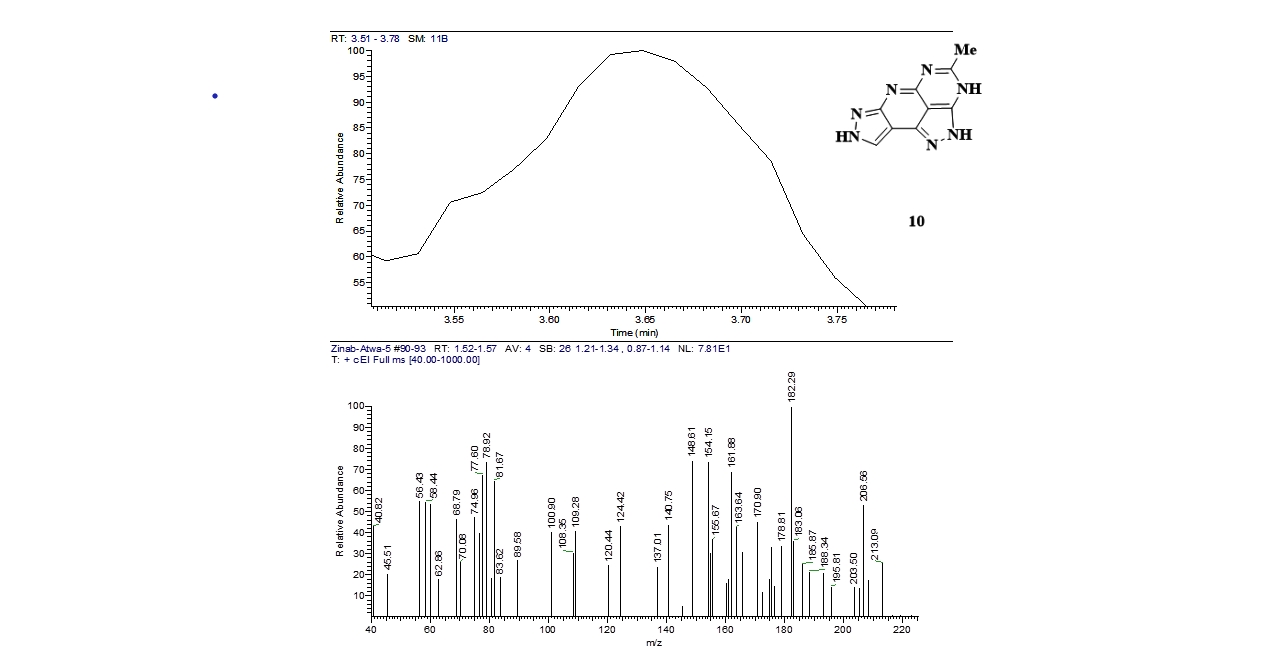


**S42. MS of compound 10**


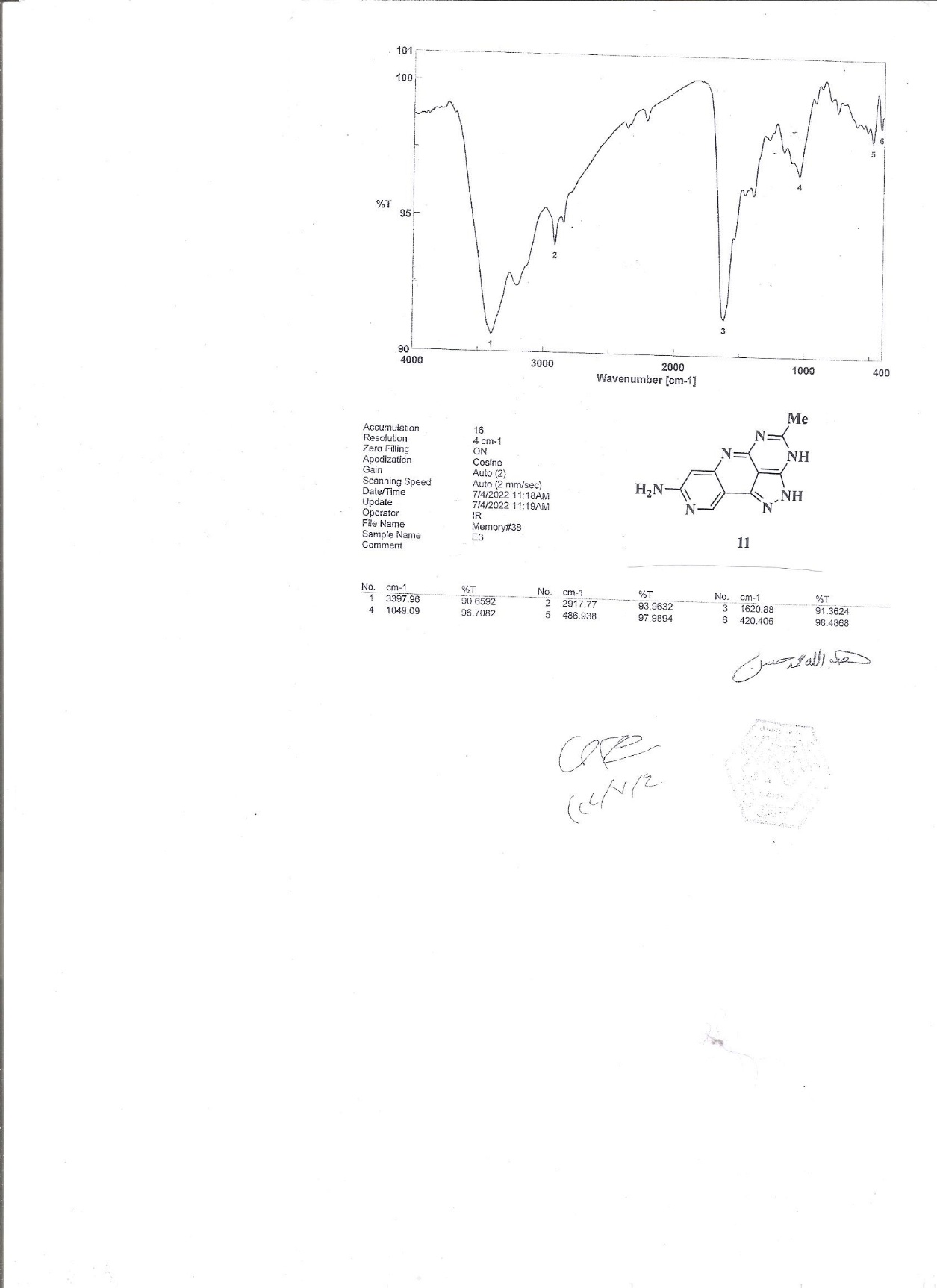


**S43. IR of compound 11**


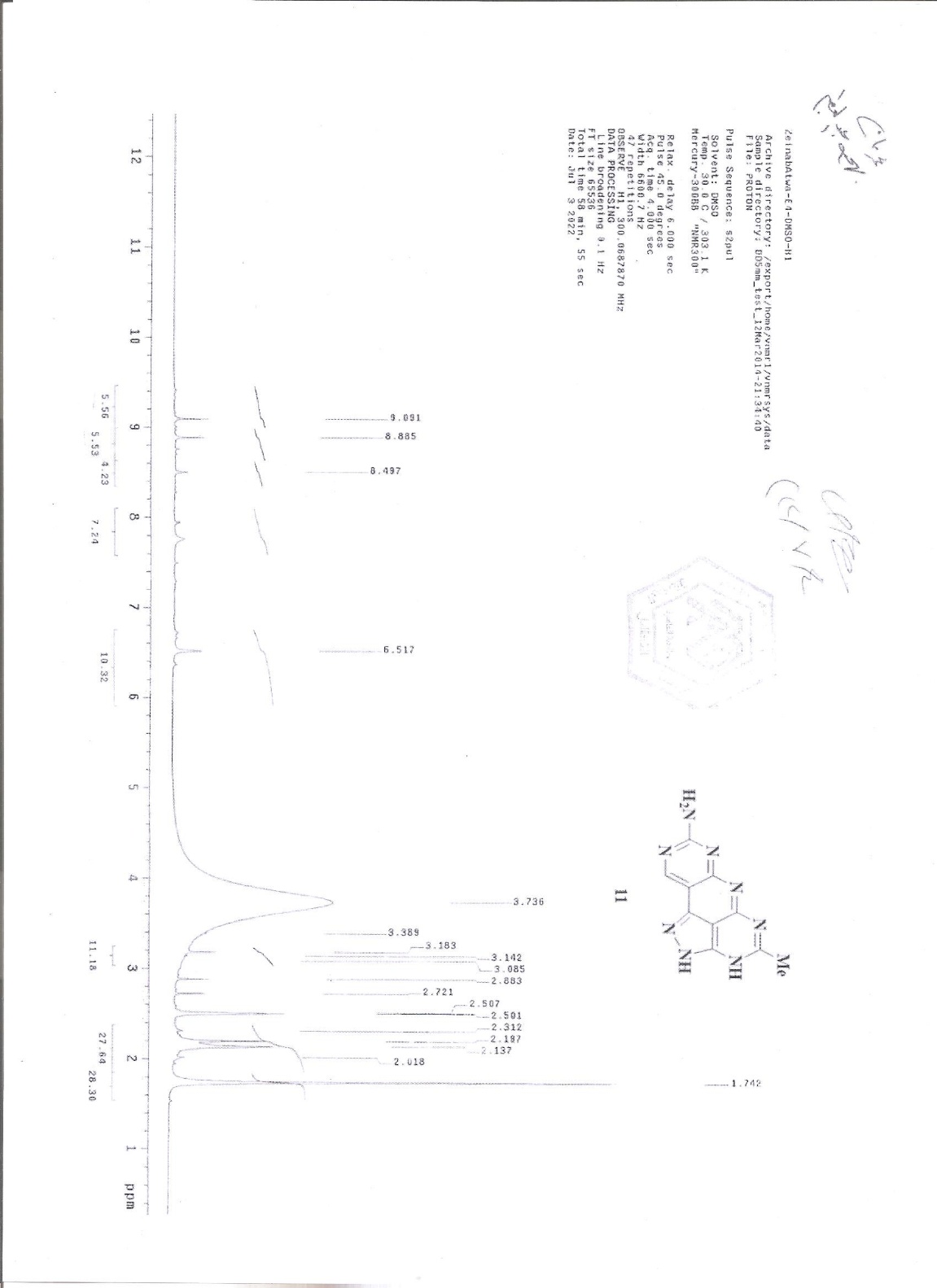


**S44. ^1^H NMR of compound 11**


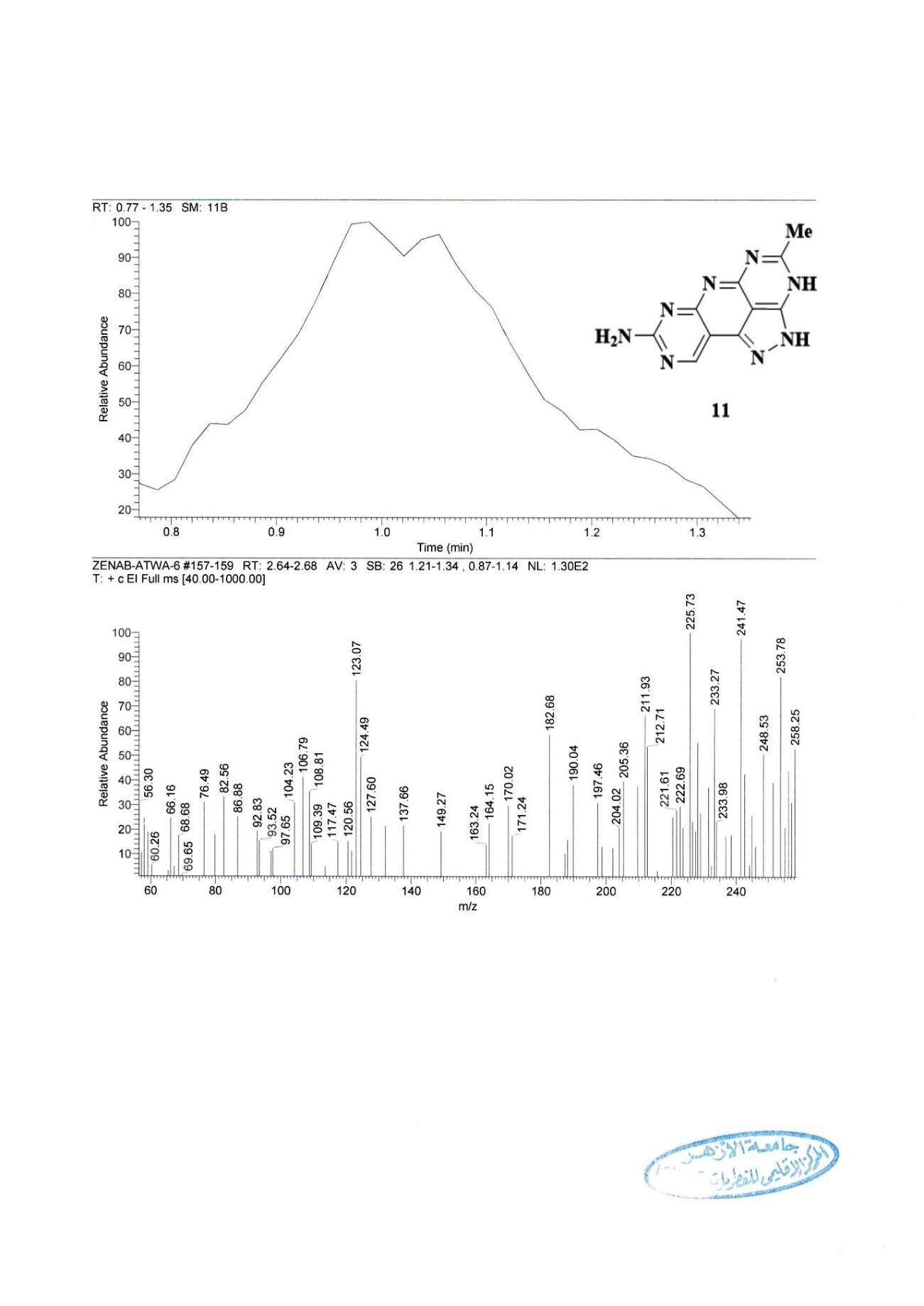


**S45. MS of compound 11**


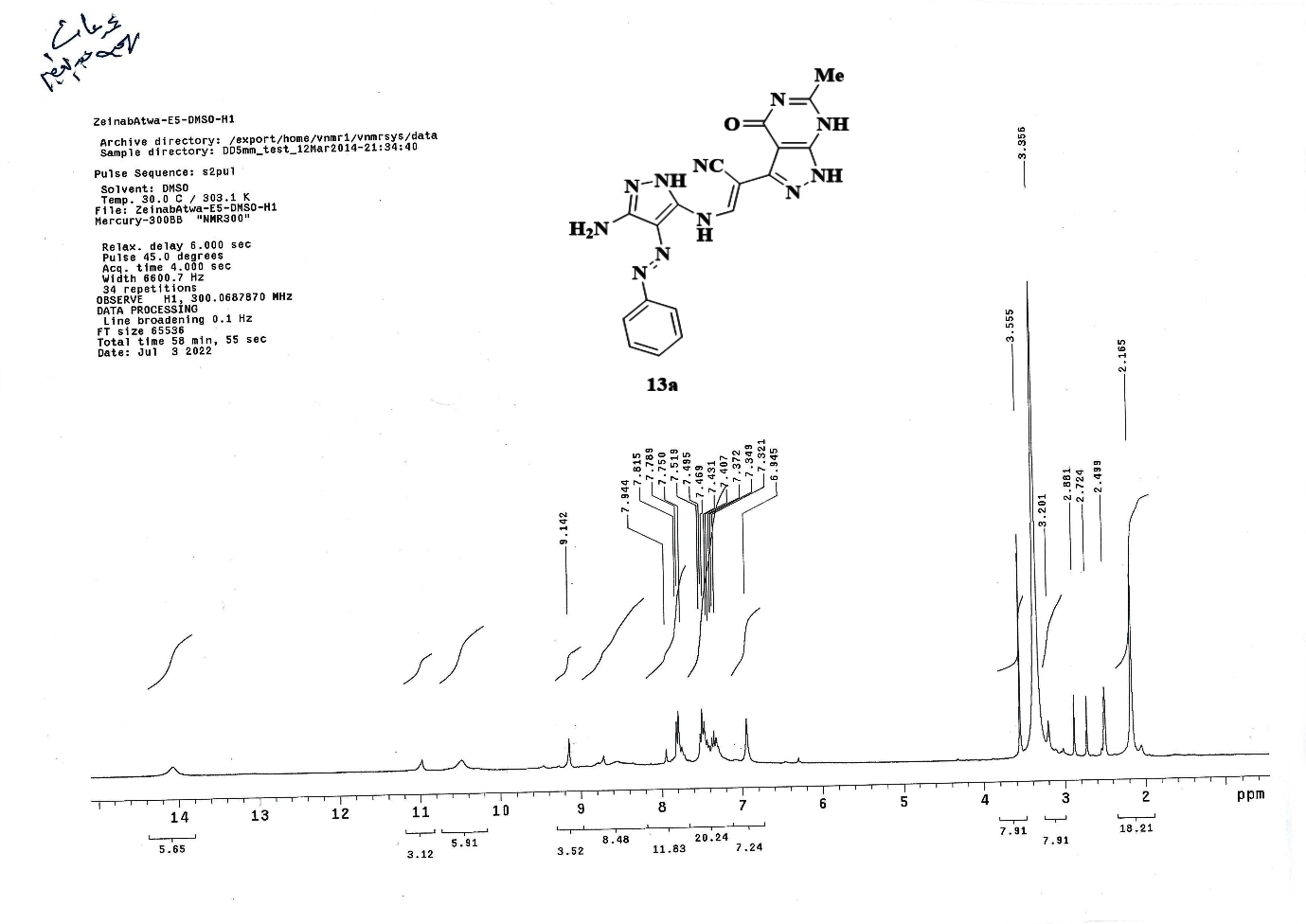


**S46. ^1^H NMR of 13a**


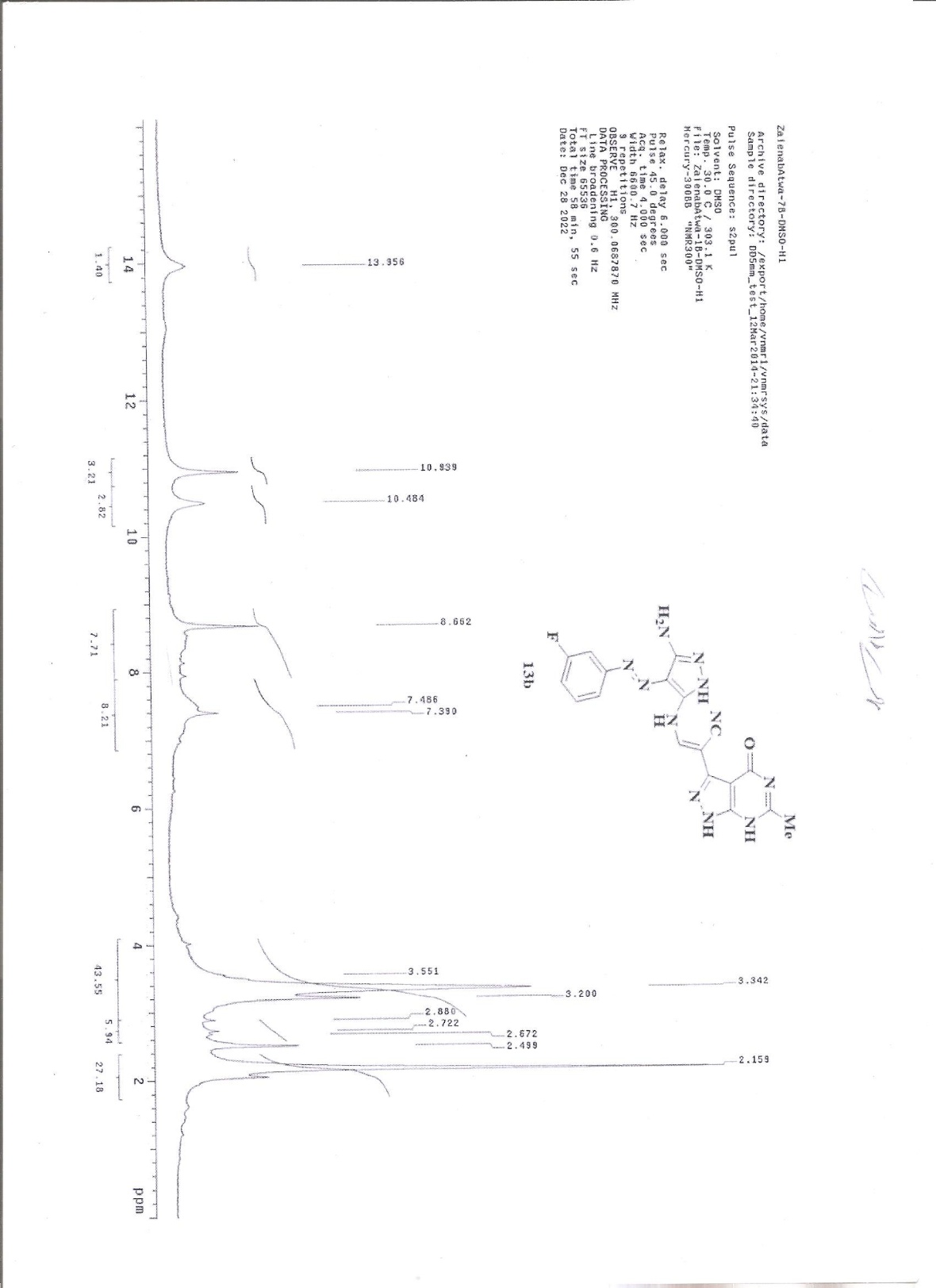


**S47. ^1^H NMR of 13b**


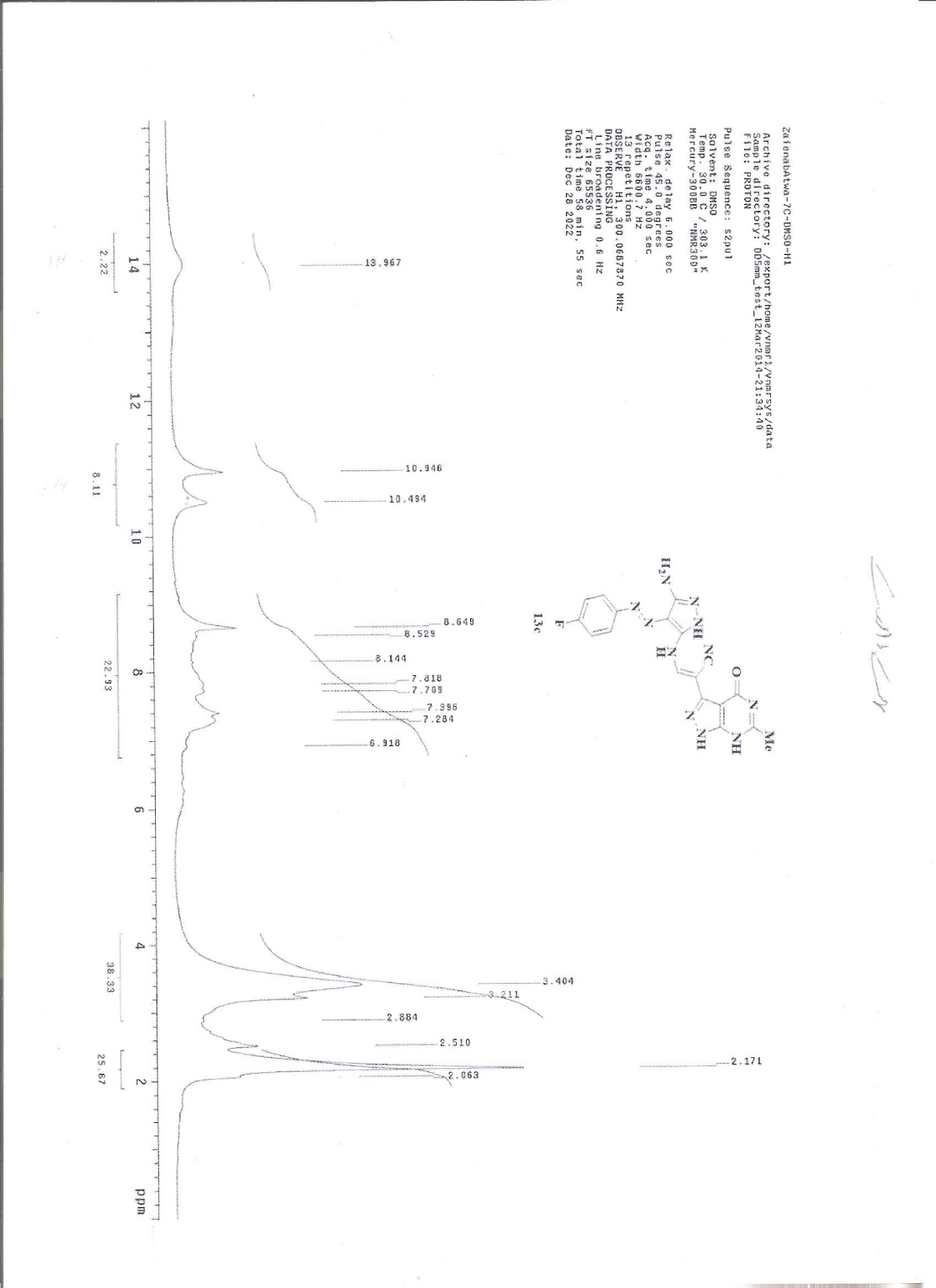


**S48. ^1^H NMR of 13c**


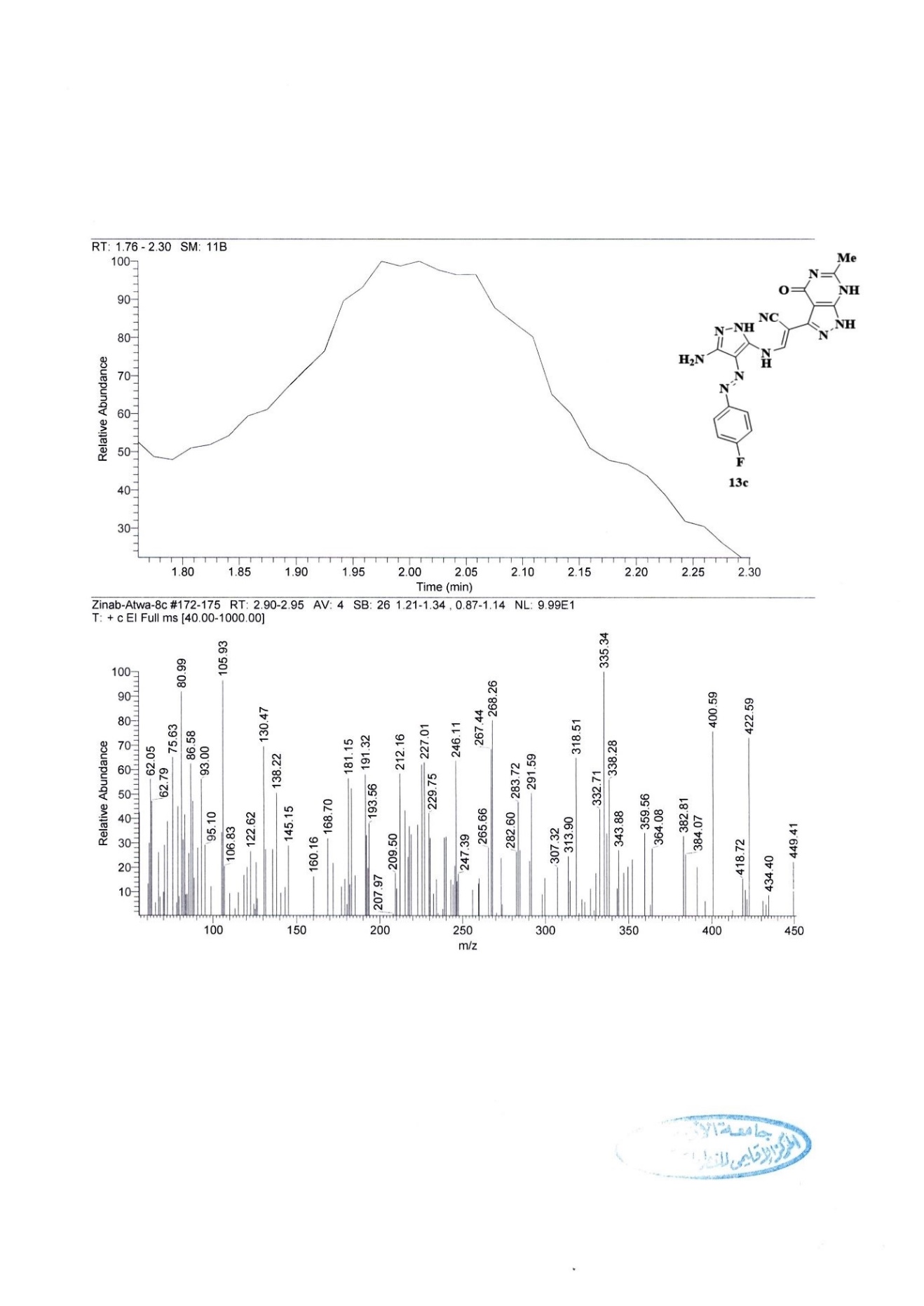


**S49. MS of compound 13c**
