# Supplementary material for: Synthesis of new pyrazolo[3,4-d]pyrimidines as potential mutant EGFR/HER2 and Bcl2 inhibitors: anticancer evaluation, DFT, molecular docking and ADME studies
Source: BMC Chem. 2026 May 18;20(1):104. doi: 10.1186/s13065-026-01773-6 (PMC13188323; doi:10.1186/s13065-026-01773-6)
Supplement: Supplementary file 2 — Supplementary Material 2. [file 13065_2026_1773_MOESM2_ESM.docx]

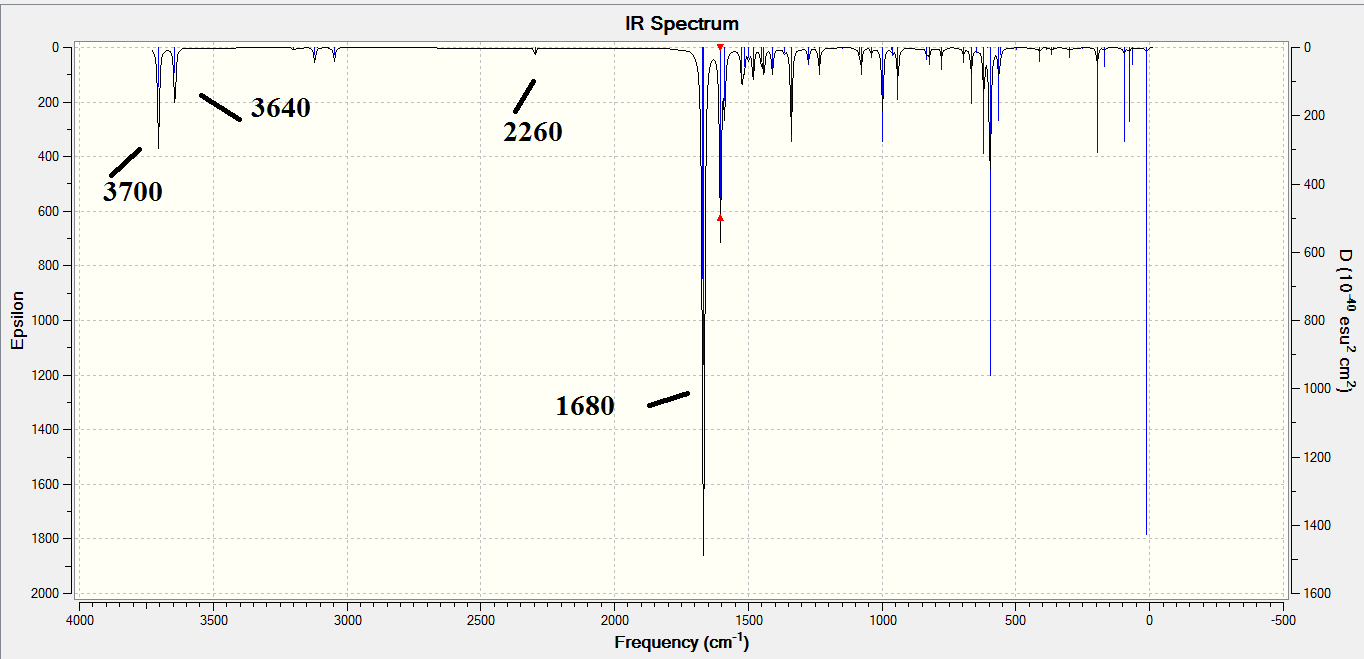


The theoretical IR spectrum of **4**

| Experimental | Theoretical | Assignment |
| --- | --- | --- |
| 3290 | 3700 | NH |
| 3250 | 3640 | NH |
| 2232 | 2260 | CN |
| 1693 | 1680 | CO |


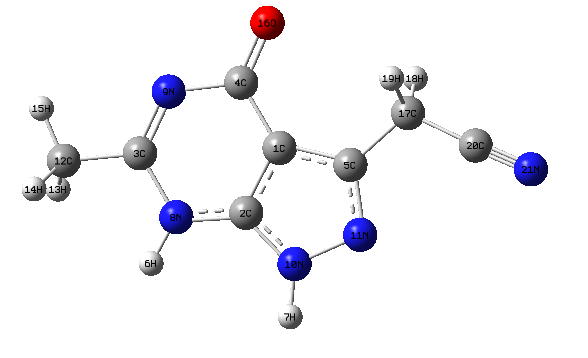


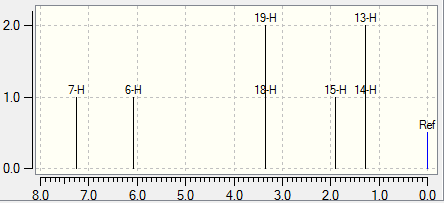


The theoretical HNMR spectrum of **4**


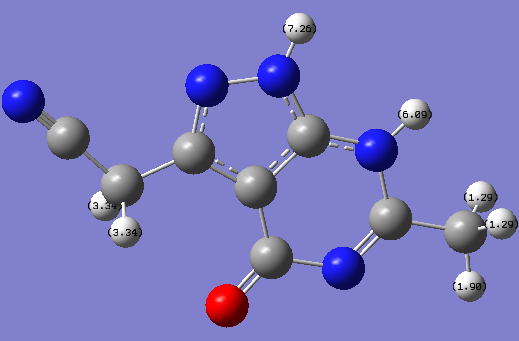


| Experimental | Theoretical | Assignment |
| --- | --- | --- |
| 2.11 (same environment) | 1.29  1.90 | 13H,14H  15H |
| 4.14 | 3.34 | 18H  19H |
| 11.21 | 6.09 | 6H |
| 13.41 | 7.26 | 7H |


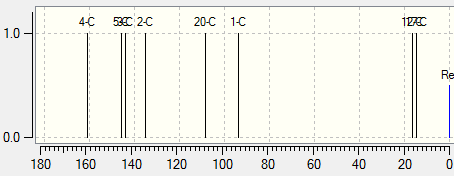


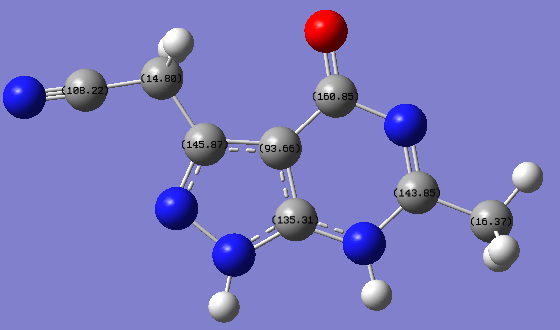


| Experimental | Theoretical | Assignment |
| --- | --- | --- |
| 16.26 | 14.88 | 17C |
| 22.80 | 16.37 | 12C |
| 78.36 | 93.66 | 1C |
| 112.56 | 108.22 | 20C |
| 116.71 | 135.31 | 2C |
| 143.35 | 143.85 | 3C |
| 143.83 | 145.87 | 5C |
| 169.04 | 160.85 | 4C |

**Lab Report**

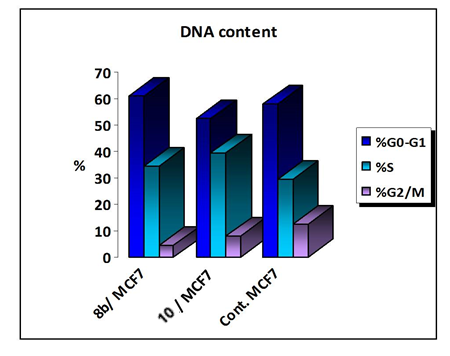


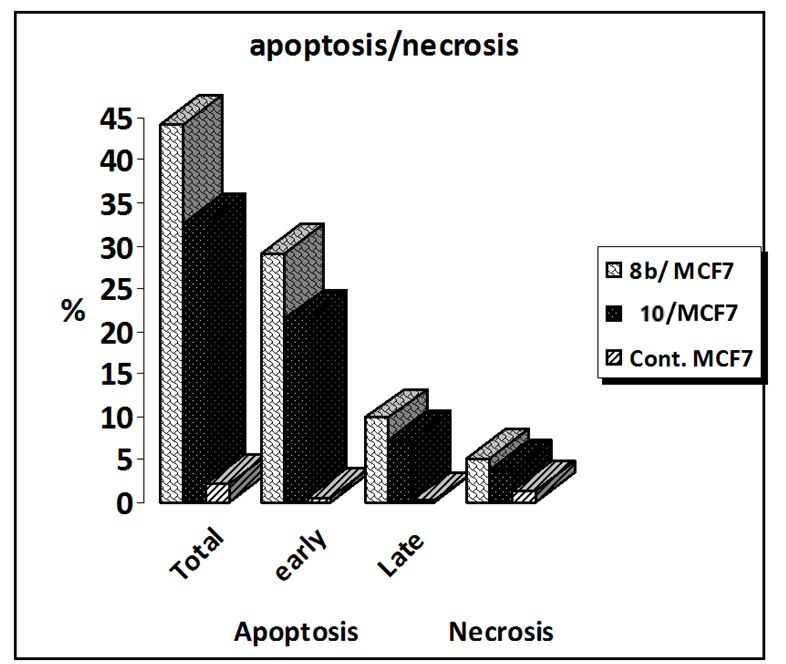


**Detailed results**


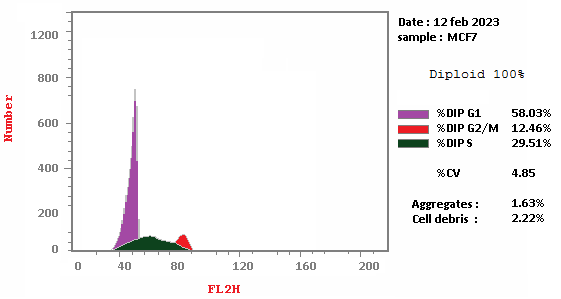


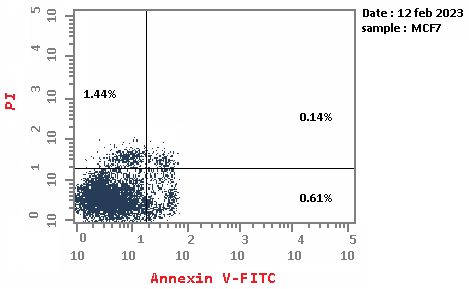


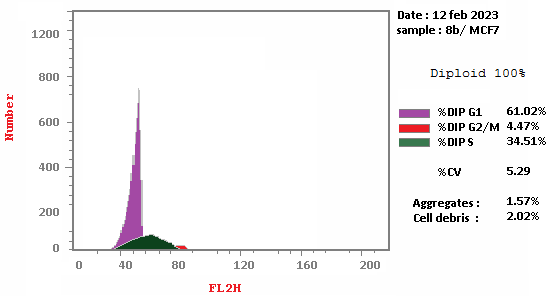


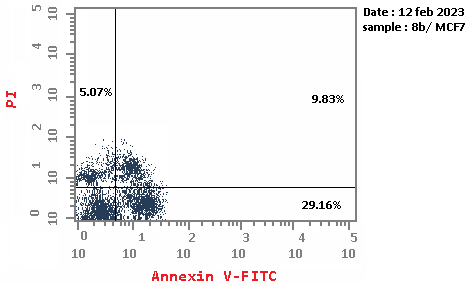


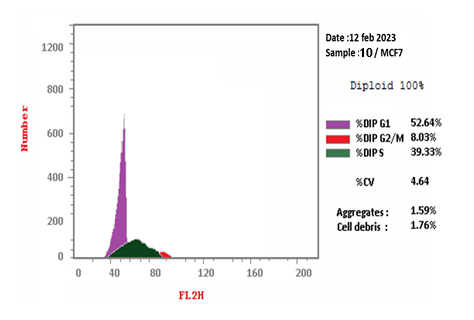

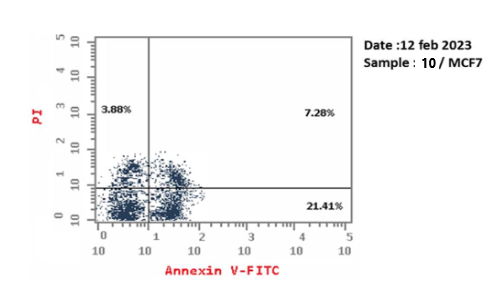


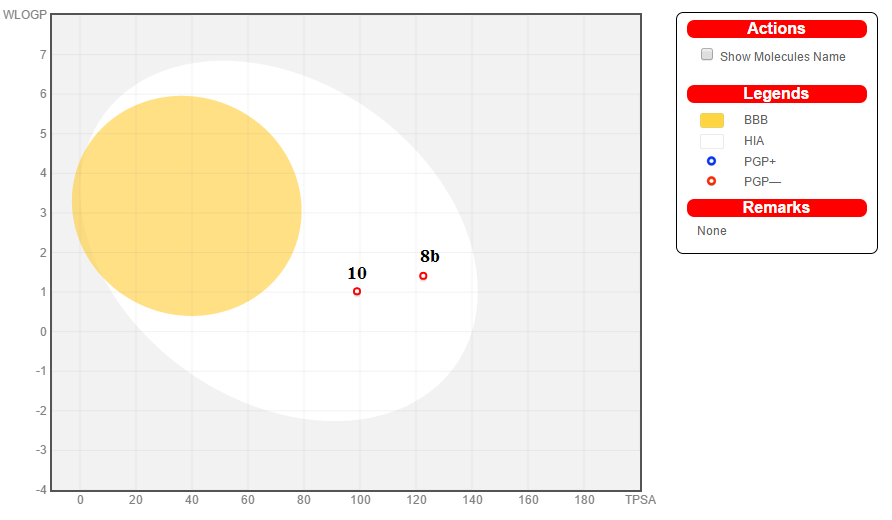


Boiled egg chart showing the gastrointestinal absorption and BBB penetration capability of compounds **8b** and **10**

**
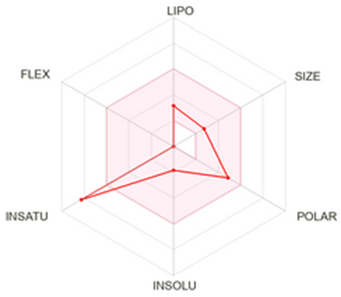

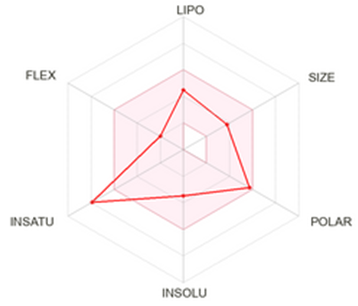

 8b 10**

The bioavailability radar chart of **8b** and **10**, the pink zone signifies the range of the values of the optimal oral bioavailability range and the predicted properties of compounds were demonstrated as red lines. Lipophilicity (LIPO), Flexibility (FLEX), Insolubility (INSOLU), SIZE and Polarity (POLAR), Instauration (INSATU).
