# Supplementary material for: Synthesis of new pyrazolo[3,4-d]pyrimidines as potential mutant EGFR/HER2 and Bcl2 inhibitors: anticancer evaluation, DFT, molecular docking and ADME studies
Source: BMC Chem. 2026 May 18;20(1):104. doi: 10.1186/s13065-026-01773-6 (PMC13188323; doi:10.1186/s13065-026-01773-6)
Supplement: Supplementary file 3 — Supplementary Material 3. [file 13065_2026_1773_MOESM3_ESM.docx]

HePG2

ONEWAY Staph BY test

/STATISTICS DESCRIPTIVES

/MISSING ANALYSIS

/POSTHOC=LSD ALPHA(0.05).

**One way**

| **Notes** | | |
| --- | --- | --- |
| Output Created | | 25-OCT-2025 14:53:18 |
| Comments | |  |
| Input | Data | C:\Users\dr\Desktop\my work. sav |
|  | Active Dataset | DataSet1 |
|  | Filter | <none> |
|  | Weight | <none> |
|  | Split File | <none> |
|  | N of Rows in Working Data File | 75 |
| Missing Value Handling | Definition of Missing | User-defined missing values are treated as missing. |
|  | Cases Used | Statistics for each analysis are based on cases with no missing data for any variable in the analysis. |
| Syntax | | ONEWAY Staph BY test  /STATISTICS DESCRIPTIVES  /MISSING ANALYSIS  /POSTHOC=LSD ALPHA(0.05). |
| Resources | Processor Time | 00:00:00.20 |
|  | Elapsed Time | 00:00:00.30 |

[DataSet1] C:\Users\dr\Desktop\my work.sav

| **Descriptives** | | | | | | |
| --- | --- | --- | --- | --- | --- | --- |
| Staph | | | | | | |
|  | N | Mean | Std. Deviation | Std. Error | 95% Confidence Interval for Mean | |
|  |  |  |  |  | Lower Bound | Upper Bound |
| 4 | 3 | 33.45000 | 2.305971 | 1.331353 | 27.72165 | 39.17835 |
| 6a | 3 | 71.22667 | 2.300464 | 1.328173 | 65.51200 | 76.94134 |
| 6b | 3 | 50.91000 | 3.140430 | 1.813128 | 43.10874 | 58.71126 |
| 6c | 3 | 12.68333 | 1.075097 | .620707 | 10.01264 | 15.35402 |
| 6d | 3 | 51.84000 | 3.209174 | 1.852818 | 43.86797 | 59.81203 |
| 6e | 3 | 62.81000 | 3.451565 | 1.992762 | 54.23584 | 71.38416 |
| 6f | 3 | 25.75000 | 1.829617 | 1.056330 | 21.20498 | 30.29502 |
| 8a | 3 | 19.79333 | 1.580548 | .912530 | 15.86703 | 23.71963 |
| 8b | 3 | 10.38333 | .889288 | .513431 | 8.17422 | 12.59245 |
| 8c | 3 | 40.80000 | 2.700000 | 1.558846 | 34.09283 | 47.50717 |
| 10 | 3 | 11.61000 | .900167 | .519711 | 9.37386 | 13.84614 |
| 11 | 3 | 72.51000 | 3.816582 | 2.203505 | 63.02908 | 81.99092 |
| 13b | 3 | 37.31000 | 2.147114 | 1.239637 | 31.97627 | 42.64373 |
| 13c | 3 | 19.96000 | 1.420422 | .820081 | 16.43147 | 23.48853 |
| erlotinib | 3 | 11.30667 | .602522 | .347867 | 9.80992 | 12.80342 |
| Total | 45 | 35.48956 | 21.652484 | 3.227762 | 28.98443 | 41.99468 |

| **Descriptives** | | |
| --- | --- | --- |
| Staph | | |
|  | Minimum | Maximum |
|  |  |  |
| 4 | 30.800 | 35.000 |
| 6a | 68.700 | 73.200 |
| 6b | 48.100 | 54.300 |
| 6c | 11.500 | 13.600 |
| 6d | 48.500 | 54.900 |
| 6e | 59.180 | 66.050 |
| 6f | 23.850 | 27.500 |
| 8a | 18.380 | 21.500 |
| 8b | 9.600 | 11.350 |
| 8c | 38.100 | 43.500 |
| 10 | 10.630 | 12.400 |
| 11 | 69.300 | 76.730 |
| 13b | 35.110 | 39.400 |
| 13c | 18.520 | 21.360 |
| erlotinib | 10.910 | 12.000 |
| Total | 9.600 | 76.730 |

| **ANOVA** | | | | | |
| --- | --- | --- | --- | --- | --- |
| Staph | | | | | |
|  | Sum of Squares | df | Mean Square | F | Sig. |
| Between Groups | 20468.255 | 14 | 1462.018 | 273.671 | .000 |
| Within Groups | 160.267 | 30 | 5.342 |  |  |
| Total | 20628.523 | 44 |  |  |  |

**Post Hoc Tests**

| **Multiple Comparisons** | | | | | | |
| --- | --- | --- | --- | --- | --- | --- |
| Dependent Variable: Staph  LSD | | | | | | |
| (I) test | (J) test | Mean Difference (I-J) | Std. Error | Sig. | 95% Confidence Interval | |
|  |  |  |  |  | Lower Bound | Upper Bound |
| 1 | 2 | -37.776667^*^ | 1.887193 | .000 | -41.63083 | -33.92250 |
|  | 3 | -17.460000^*^ | 1.887193 | .000 | -21.31416 | -13.60584 |
|  | 4 | 20.766667^*^ | 1.887193 | .000 | 16.91250 | 24.62083 |
|  | 5 | -18.390000^*^ | 1.887193 | .000 | -22.24416 | -14.53584 |
|  | 6 | -29.360000^*^ | 1.887193 | .000 | -33.21416 | -25.50584 |
|  | 7 | 7.700000^*^ | 1.887193 | .000 | 3.84584 | 11.55416 |
|  | 8 | 13.656667^*^ | 1.887193 | .000 | 9.80250 | 17.51083 |
|  | 9 | 23.066667^*^ | 1.887193 | .000 | 19.21250 | 26.92083 |
|  | 10 | -7.350000^*^ | 1.887193 | .001 | -11.20416 | -3.49584 |
|  | 11 | 21.840000^*^ | 1.887193 | .000 | 17.98584 | 25.69416 |
|  | 12 | -39.060000^*^ | 1.887193 | .000 | -42.91416 | -35.20584 |
|  | 13 | -3.860000^*^ | 1.887193 | .050 | -7.71416 | -.00584 |
|  | 14 | 13.490000^*^ | 1.887193 | .000 | 9.63584 | 17.34416 |
|  | 15 | 22.143333^*^ | 1.887193 | .000 | 18.28917 | 25.99750 |
| 2 | 1 | 37.776667^*^ | 1.887193 | .000 | 33.92250 | 41.63083 |
|  | 3 | 20.316667^*^ | 1.887193 | .000 | 16.46250 | 24.17083 |
|  | 4 | 58.543333^*^ | 1.887193 | .000 | 54.68917 | 62.39750 |
|  | 5 | 19.386667^*^ | 1.887193 | .000 | 15.53250 | 23.24083 |
|  | 6 | 8.416667^*^ | 1.887193 | .000 | 4.56250 | 12.27083 |
|  | 7 | 45.476667^*^ | 1.887193 | .000 | 41.62250 | 49.33083 |
|  | 8 | 51.433333^*^ | 1.887193 | .000 | 47.57917 | 55.28750 |
|  | 9 | 60.843333^*^ | 1.887193 | .000 | 56.98917 | 64.69750 |
|  | 10 | 30.426667^*^ | 1.887193 | .000 | 26.57250 | 34.28083 |
|  | 11 | 59.616667^*^ | 1.887193 | .000 | 55.76250 | 63.47083 |
|  | 12 | -1.283333 | 1.887193 | .502 | -5.13750 | 2.57083 |
|  | 13 | 33.916667^*^ | 1.887193 | .000 | 30.06250 | 37.77083 |
|  | 14 | 51.266667^*^ | 1.887193 | .000 | 47.41250 | 55.12083 |
|  | 15 | 59.920000^*^ | 1.887193 | .000 | 56.06584 | 63.77416 |
| 3 | 1 | 17.460000^*^ | 1.887193 | .000 | 13.60584 | 21.31416 |
|  | 2 | -20.316667^*^ | 1.887193 | .000 | -24.17083 | -16.46250 |
|  | 4 | 38.226667^*^ | 1.887193 | .000 | 34.37250 | 42.08083 |
|  | 5 | -.930000 | 1.887193 | .626 | -4.78416 | 2.92416 |
|  | 6 | -11.900000^*^ | 1.887193 | .000 | -15.75416 | -8.04584 |

| **Multiple Comparisons** | | | | | | |
| --- | --- | --- | --- | --- | --- | --- |
| Dependent Variable: Staph  LSD | | | | | | |
| (I) test | (J) test | Mean Difference (I-J) | Std. Error | Sig. | 95% Confidence Interval | |
|  |  |  |  |  | Lower Bound | Upper Bound |
| 3 | 7 | 25.160000^*^ | 1.887193 | .000 | 21.30584 | 29.01416 |
|  | 8 | 31.116667^*^ | 1.887193 | .000 | 27.26250 | 34.97083 |
|  | 9 | 40.526667^*^ | 1.887193 | .000 | 36.67250 | 44.38083 |
|  | 10 | 10.110000^*^ | 1.887193 | .000 | 6.25584 | 13.96416 |
|  | 11 | 39.300000^*^ | 1.887193 | .000 | 35.44584 | 43.15416 |
|  | 12 | -21.600000^*^ | 1.887193 | .000 | -25.45416 | -17.74584 |
|  | 13 | 13.600000^*^ | 1.887193 | .000 | 9.74584 | 17.45416 |
|  | 14 | 30.950000^*^ | 1.887193 | .000 | 27.09584 | 34.80416 |
|  | 15 | 39.603333^*^ | 1.887193 | .000 | 35.74917 | 43.45750 |
| 4 | 1 | -20.766667^*^ | 1.887193 | .000 | -24.62083 | -16.91250 |
|  | 2 | -58.543333^*^ | 1.887193 | .000 | -62.39750 | -54.68917 |
|  | 3 | -38.226667^*^ | 1.887193 | .000 | -42.08083 | -34.37250 |
|  | 5 | -39.156667^*^ | 1.887193 | .000 | -43.01083 | -35.30250 |
|  | 6 | -50.126667^*^ | 1.887193 | .000 | -53.98083 | -46.27250 |
|  | 7 | -13.066667^*^ | 1.887193 | .000 | -16.92083 | -9.21250 |
|  | 8 | -7.110000^*^ | 1.887193 | .001 | -10.96416 | -3.25584 |
|  | 9 | 2.300000^*^ | 1.887193 | .232 | -1.55416 | 6.15416 |
|  | 10 | -28.116667^*^ | 1.887193 | .000 | -31.97083 | -24.26250 |
|  | 11 | 1.073333^*^ | 1.887193 | .574 | -2.78083 | 4.92750 |
|  | 12 | -59.826667^*^ | 1.887193 | .000 | -63.68083 | -55.97250 |
|  | 13 | -24.626667^*^ | 1.887193 | .000 | -28.48083 | -20.77250 |
|  | 14 | -7.276667^*^ | 1.887193 | .001 | -11.13083 | -3.42250 |
|  | 15 | 1.376667^*^ | 1.887193 | .471 | -2.47750 | 5.23083 |
| 5 | 1 | 18.390000^*^ | 1.887193 | .000 | 14.53584 | 22.24416 |
|  | 2 | -19.386667 | 1.887193 | .000 | -23.24083 | -15.53250 |
|  | 3 | .930000^*^ | 1.887193 | .626 | -2.92416 | 4.78416 |
|  | 4 | 39.156667^*^ | 1.887193 | .000 | 35.30250 | 43.01083 |
|  | 6 | -10.970000^*^ | 1.887193 | .000 | -14.82416 | -7.11584 |
|  | 7 | 26.090000^*^ | 1.887193 | .000 | 22.23584 | 29.94416 |
|  | 8 | 32.046667^*^ | 1.887193 | .000 | 28.19250 | 35.90083 |
|  | 9 | 41.456667^*^ | 1.887193 | .000 | 37.60250 | 45.31083 |
|  | 10 | 11.040000 | 1.887193 | .000 | 7.18584 | 14.89416 |
|  | 11 | 40.230000^*^ | 1.887193 | .000 | 36.37584 | 44.08416 |

| **Multiple Comparisons** | | | | | | |
| --- | --- | --- | --- | --- | --- | --- |
| Dependent Variable: Staph  LSD | | | | | | |
| (I) test | (J) test | Mean Difference (I-J) | Std. Error | Sig. | 95% Confidence Interval | |
|  |  |  |  |  | Lower Bound | Upper Bound |
| 5 | 12 | -20.670000^*^ | 1.887193 | .000 | -24.52416 | -16.81584 |
|  | 13 | 14.530000^*^ | 1.887193 | .000 | 10.67584 | 18.38416 |
|  | 14 | 31.880000^*^ | 1.887193 | .000 | 28.02584 | 35.73416 |
|  | 15 | 40.533333^*^ | 1.887193 | .000 | 36.67917 | 44.38750 |
| 6 | 1 | 29.360000^*^ | 1.887193 | .000 | 25.50584 | 33.21416 |
|  | 2 | -8.416667^*^ | 1.887193 | .000 | -12.27083 | -4.56250 |
|  | 3 | 11.900000^*^ | 1.887193 | .000 | 8.04584 | 15.75416 |
|  | 4 | 50.126667^*^ | 1.887193 | .000 | 46.27250 | 53.98083 |
|  | 5 | 10.970000^*^ | 1.887193 | .000 | 7.11584 | 14.82416 |
|  | 7 | 37.060000^*^ | 1.887193 | .000 | 33.20584 | 40.91416 |
|  | 8 | 43.016667^*^ | 1.887193 | .000 | 39.16250 | 46.87083 |
|  | 9 | 52.426667^*^ | 1.887193 | .000 | 48.57250 | 56.28083 |
|  | 10 | 22.010000^*^ | 1.887193 | .000 | 18.15584 | 25.86416 |
|  | 11 | 51.200000^*^ | 1.887193 | .000 | 47.34584 | 55.05416 |
|  | 12 | -9.700000^*^ | 1.887193 | .000 | -13.55416 | -5.84584 |
|  | 13 | 25.500000^*^ | 1.887193 | .000 | 21.64584 | 29.35416 |
|  | 14 | 42.850000^*^ | 1.887193 | .000 | 38.99584 | 46.70416 |
|  | 15 | 51.503333^*^ | 1.887193 | .000 | 47.64917 | 55.35750 |
| 7 | 1 | -7.700000^*^ | 1.887193 | .000 | -11.55416 | -3.84584 |
|  | 2 | -45.476667^*^ | 1.887193 | .000 | -49.33083 | -41.62250 |
|  | 3 | -25.160000^*^ | 1.887193 | .000 | -29.01416 | -21.30584 |
|  | 4 | 13.066667^*^ | 1.887193 | .000 | 9.21250 | 16.92083 |
|  | 5 | -26.090000^*^ | 1.887193 | .000 | -29.94416 | -22.23584 |
|  | 6 | -37.060000^*^ | 1.887193 | .000 | -40.91416 | -33.20584 |
|  | 8 | 5.956667 | 1.887193 | .004 | 2.10250 | 9.81083 |
|  | 9 | 15.366667^*^ | 1.887193 | .000 | 11.51250 | 19.22083 |
|  | 10 | -15.050000^*^ | 1.887193 | .000 | -18.90416 | -11.19584 |
|  | 11 | 14.140000^*^ | 1.887193 | .000 | 10.28584 | 17.99416 |
|  | 12 | -46.760000^*^ | 1.887193 | .000 | -50.61416 | -42.90584 |
|  | 13 | -11.560000^*^ | 1.887193 | .000 | -15.41416 | -7.70584 |
|  | 14 | 5.790000^*^ | 1.887193 | .005 | 1.93584 | 9.64416 |
|  | 15 | 14.443333 | 1.887193 | .000 | 10.58917 | 18.29750 |
| 8 | 1 | -13.656667^*^ | 1.887193 | .000 | -17.51083 | -9.80250 |

| **Multiple Comparisons** | | | | | | |
| --- | --- | --- | --- | --- | --- | --- |
| Dependent Variable: Staph  LSD | | | | | | |
| (I) test | (J) test | Mean Difference (I-J) | Std. Error | Sig. | 95% Confidence Interval | |
|  |  |  |  |  | Lower Bound | Upper Bound |
| 8 | 2 | -51.433333^*^ | 1.887193 | .000 | -55.28750 | -47.57917 |
|  | 3 | -31.116667^*^ | 1.887193 | .000 | -34.97083 | -27.26250 |
|  | 4 | 7.110000^*^ | 1.887193 | .001 | 3.25584 | 10.96416 |
|  | 5 | -32.046667^*^ | 1.887193 | .000 | -35.90083 | -28.19250 |
|  | 6 | -43.016667^*^ | 1.887193 | .000 | -46.87083 | -39.16250 |
|  | 7 | -5.956667^*^ | 1.887193 | .004 | -9.81083 | -2.10250 |
|  | 9 | 9.410000^*^ | 1.887193 | .000 | 5.55584 | 13.26416 |
|  | 10 | -21.006667^*^ | 1.887193 | .000 | -24.86083 | -17.15250 |
|  | 11 | 8.183333^*^ | 1.887193 | .000 | 4.32917 | 12.03750 |
|  | 12 | -52.716667^*^ | 1.887193 | .000 | -56.57083 | -48.86250 |
|  | 13 | -17.516667^*^ | 1.887193 | .000 | -21.37083 | -13.66250 |
|  | 14 | -.166667^*^ | 1.887193 | .930 | -4.02083 | 3.68750 |
|  | 15 | 8.486667^*^ | 1.887193 | .000 | 4.63250 | 12.34083 |
| 9 | 1 | -23.066667^*^ | 1.887193 | .000 | -26.92083 | -19.21250 |
|  | 2 | -60.843333^*^ | 1.887193 | .000 | -64.69750 | -56.98917 |
|  | 3 | -40.526667^*^ | 1.887193 | .000 | -44.38083 | -36.67250 |
|  | 4 | -2.300000^*^ | 1.887193 | .232 | -6.15416 | 1.55416 |
|  | 5 | -41.456667^*^ | 1.887193 | .000 | -45.31083 | -37.60250 |
|  | 6 | -52.426667^*^ | 1.887193 | .000 | -56.28083 | -48.57250 |
|  | 7 | -15.366667^*^ | 1.887193 | .000 | -19.22083 | -11.51250 |
|  | 8 | -9.410000^*^ | 1.887193 | .000 | -13.26416 | -5.55584 |
|  | 10 | -30.416667^*^ | 1.887193 | .000 | -34.27083 | -26.56250 |
|  | 11 | -1.226667^*^ | 1.887193 | .521 | -5.08083 | 2.62750 |
|  | 12 | -62.126667^*^ | 1.887193 | .000 | -65.98083 | -58.27250 |
|  | 13 | -26.926667 | 1.887193 | .000 | -30.78083 | -23.07250 |
|  | 14 | -9.576667^*^ | 1.887193 | .000 | -13.43083 | -5.72250 |
|  | 15 | -.923333^*^ | 1.887193 | .628 | -4.77750 | 2.93083 |
| 10 | 1 | 7.350000^*^ | 1.887193 | .001 | 3.49584 | 11.20416 |
|  | 2 | -30.426667^*^ | 1.887193 | .000 | -34.28083 | -26.57250 |
|  | 3 | -10.110000^*^ | 1.887193 | .000 | -13.96416 | -6.25584 |
|  | 4 | 28.116667^*^ | 1.887193 | .000 | 24.26250 | 31.97083 |
|  | 5 | -11.040000 | 1.887193 | .000 | -14.89416 | -7.18584 |
|  | 6 | -22.010000^*^ | 1.887193 | .000 | -25.86416 | -18.15584 |

| **Multiple Comparisons** | | | | | | |
| --- | --- | --- | --- | --- | --- | --- |
| Dependent Variable: Staph  LSD | | | | | | |
| (I) test | (J) test | Mean Difference (I-J) | Std. Error | Sig. | 95% Confidence Interval | |
|  |  |  |  |  | Lower Bound | Upper Bound |
| 10 | 7 | 15.050000^*^ | 1.887193 | .000 | 11.19584 | 18.90416 |
|  | 8 | 21.006667^*^ | 1.887193 | .000 | 17.15250 | 24.86083 |
|  | 9 | 30.416667^*^ | 1.887193 | .000 | 26.56250 | 34.27083 |
|  | 11 | 29.190000^*^ | 1.887193 | .000 | 25.33584 | 33.04416 |
|  | 12 | -31.710000^*^ | 1.887193 | .000 | -35.56416 | -27.85584 |
|  | 13 | 3.490000^*^ | 1.887193 | .074 | -.36416 | 7.34416 |
|  | 14 | 20.840000^*^ | 1.887193 | .000 | 16.98584 | 24.69416 |
|  | 15 | 29.493333^*^ | 1.887193 | .000 | 25.63917 | 33.34750 |
| 11 | 1 | -21.840000^*^ | 1.887193 | .000 | -25.69416 | -17.98584 |
|  | 2 | -59.616667^*^ | 1.887193 | .000 | -63.47083 | -55.76250 |
|  | 3 | -39.300000^*^ | 1.887193 | .000 | -43.15416 | -35.44584 |
|  | 4 | -1.073333^*^ | 1.887193 | .574 | -4.92750 | 2.78083 |
|  | 5 | -40.230000^*^ | 1.887193 | .000 | -44.08416 | -36.37584 |
|  | 6 | -51.200000^*^ | 1.887193 | .000 | -55.05416 | -47.34584 |
|  | 7 | -14.140000^*^ | 1.887193 | .000 | -17.99416 | -10.28584 |
|  | 8 | -8.183333^*^ | 1.887193 | .000 | -12.03750 | -4.32917 |
|  | 9 | 1.226667^*^ | 1.887193 | .521 | -2.62750 | 5.08083 |
|  | 10 | -29.190000^*^ | 1.887193 | .000 | -33.04416 | -25.33584 |
|  | 12 | -60.900000^*^ | 1.887193 | .000 | -64.75416 | -57.04584 |
|  | 13 | -25.700000^*^ | 1.887193 | .000 | -29.55416 | -21.84584 |
|  | 14 | -8.350000^*^ | 1.887193 | .000 | -12.20416 | -4.49584 |
|  | 15 | .303333^*^ | 1.887193 | .873 | -3.55083 | 4.15750 |
| 12 | 1 | 39.060000^*^ | 1.887193 | .000 | 35.20584 | 42.91416 |
|  | 2 | 1.283333^*^ | 1.887193 | .502 | -2.57083 | 5.13750 |
|  | 3 | 21.600000 | 1.887193 | .000 | 17.74584 | 25.45416 |
|  | 4 | 59.826667^*^ | 1.887193 | .000 | 55.97250 | 63.68083 |
|  | 5 | 20.670000^*^ | 1.887193 | .000 | 16.81584 | 24.52416 |
|  | 6 | 9.700000^*^ | 1.887193 | .000 | 5.84584 | 13.55416 |
|  | 7 | 46.760000^*^ | 1.887193 | .000 | 42.90584 | 50.61416 |
|  | 8 | 52.716667^*^ | 1.887193 | .000 | 48.86250 | 56.57083 |
|  | 9 | 62.126667^*^ | 1.887193 | .000 | 58.27250 | 65.98083 |
|  | 10 | 31.710000 | 1.887193 | .000 | 27.85584 | 35.56416 |
|  | 11 | 60.900000^*^ | 1.887193 | .000 | 57.04584 | 64.75416 |

| **Multiple Comparisons** | | | | | | |
| --- | --- | --- | --- | --- | --- | --- |
| Dependent Variable: Staph  LSD | | | | | | |
| (I) test | (J) test | Mean Difference (I-J) | Std. Error | Sig. | 95% Confidence Interval | |
|  |  |  |  |  | Lower Bound | Upper Bound |
| 12 | 13 | 35.200000^*^ | 1.887193 | .000 | 31.34584 | 39.05416 |
|  | 14 | 52.550000^*^ | 1.887193 | .000 | 48.69584 | 56.40416 |
|  | 15 | 61.203333^*^ | 1.887193 | .000 | 57.34917 | 65.05750 |
| 13 | 1 | 3.860000^*^ | 1.887193 | .050 | .00584 | 7.71416 |
|  | 2 | -33.916667^*^ | 1.887193 | .000 | -37.77083 | -30.06250 |
|  | 3 | -13.600000^*^ | 1.887193 | .000 | -17.45416 | -9.74584 |
|  | 4 | 24.626667^*^ | 1.887193 | .000 | 20.77250 | 28.48083 |
|  | 5 | -14.530000^*^ | 1.887193 | .000 | -18.38416 | -10.67584 |
|  | 6 | -25.500000^*^ | 1.887193 | .000 | -29.35416 | -21.64584 |
|  | 7 | 11.560000^*^ | 1.887193 | .000 | 7.70584 | 15.41416 |
|  | 8 | 17.516667^*^ | 1.887193 | .000 | 13.66250 | 21.37083 |
|  | 9 | 26.926667^*^ | 1.887193 | .000 | 23.07250 | 30.78083 |
|  | 10 | -3.490000^*^ | 1.887193 | .074 | -7.34416 | .36416 |
|  | 11 | 25.700000^*^ | 1.887193 | .000 | 21.84584 | 29.55416 |
|  | 12 | -35.200000^*^ | 1.887193 | .000 | -39.05416 | -31.34584 |
|  | 14 | 17.350000^*^ | 1.887193 | .000 | 13.49584 | 21.20416 |
|  | 15 | 26.003333^*^ | 1.887193 | .000 | 22.14917 | 29.85750 |
| 14 | 1 | -13.490000^*^ | 1.887193 | .000 | -17.34416 | -9.63584 |
|  | 2 | -51.266667^*^ | 1.887193 | .000 | -55.12083 | -47.41250 |
|  | 3 | -30.950000^*^ | 1.887193 | .000 | -34.80416 | -27.09584 |
|  | 4 | 7.276667^*^ | 1.887193 | .001 | 3.42250 | 11.13083 |
|  | 5 | -31.880000^*^ | 1.887193 | .000 | -35.73416 | -28.02584 |
|  | 6 | -42.850000^*^ | 1.887193 | .000 | -46.70416 | -38.99584 |
|  | 7 | -5.790000^*^ | 1.887193 | .005 | -9.64416 | -1.93584 |
|  | 8 | .166667 | 1.887193 | .930 | -3.68750 | 4.02083 |
|  | 9 | 9.576667^*^ | 1.887193 | .000 | 5.72250 | 13.43083 |
|  | 10 | -20.840000^*^ | 1.887193 | .000 | -24.69416 | -16.98584 |
|  | 11 | 8.350000^*^ | 1.887193 | .000 | 4.49584 | 12.20416 |
|  | 12 | -52.550000^*^ | 1.887193 | .000 | -56.40416 | -48.69584 |
|  | 13 | -17.350000^*^ | 1.887193 | .000 | -21.20416 | -13.49584 |
|  | 15 | 8.653333^*^ | 1.887193 | .000 | 4.79917 | 12.50750 |
| 15 | 1 | -22.143333 | 1.887193 | .000 | -25.99750 | -18.28917 |
|  | 2 | -59.920000^*^ | 1.887193 | .000 | -63.77416 | -56.06584 |

| **Multiple Comparisons** | | | | | | |
| --- | --- | --- | --- | --- | --- | --- |
| Dependent Variable: Staph  LSD | | | | | | |
| (I) test | (J) test | Mean Difference (I-J) | Std. Error | Sig. | 95% Confidence Interval | |
|  |  |  |  |  | Lower Bound | Upper Bound |
| 15 | 3 | -39.603333^*^ | 1.887193 | .000 | -43.45750 | -35.74917 |
|  | 4 | -1.376667^*^ | 1.887193 | .471 | -5.23083 | 2.47750 |
|  | 5 | -40.533333^*^ | 1.887193 | .000 | -44.38750 | -36.67917 |
|  | 6 | -51.503333^*^ | 1.887193 | .000 | -55.35750 | -47.64917 |
|  | 7 | -14.443333^*^ | 1.887193 | .000 | -18.29750 | -10.58917 |
|  | 8 | -8.486667^*^ | 1.887193 | .000 | -12.34083 | -4.63250 |
|  | 9 | .923333^*^ | 1.887193 | .628 | -2.93083 | 4.77750 |
|  | 10 | -29.493333^*^ | 1.887193 | .000 | -33.34750 | -25.63917 |
|  | 11 | -.303333^*^ | 1.887193 | .873 | -4.15750 | 3.55083 |
|  | 12 | -61.203333^*^ | 1.887193 | .000 | -65.05750 | -57.34917 |
|  | 13 | -26.003333^*^ | 1.887193 | .000 | -29.85750 | -22.14917 |
|  | 14 | -8.653333^*^ | 1.887193 | .000 | -12.50750 | -4.79917 |

| *. The mean difference is significant at the 0.05 level. |
| --- |

MCF-7

ONEWAY Staph BY test

/STATISTICS DESCRIPTIVES

/MISSING ANALYSIS

/POSTHOC=LSD ALPHA(0.05).

**One way**

| **Notes** | | |
| --- | --- | --- |
| Output Created | | 25-OCT-2025 23:06:14 |
| Comments | |  |
| Input | Data | C:\Users\dr\Desktop\my work.sav |
|  | Active Dataset | DataSet1 |
|  | Filter | <none> |
|  | Weight | <none> |
|  | Split File | <none> |
|  | N of Rows in Working Data File | 75 |
| Missing Value Handling | Definition of Missing | User-defined missing values are treated as missing. |
|  | Cases Used | Statistics for each analysis are based on cases with no missing data for any variable in the analysis. |
| Syntax | | ONEWAY Staph BY test  /STATISTICS DESCRIPTIVES  /MISSING ANALYSIS  /POSTHOC=LSD ALPHA(0.05). |
| Resources | Processor Time | 00:00:00.16 |
|  | Elapsed Time | 00:00:00.20 |

[DataSet1] C:\Users\dr\Desktop\my work .sav

| **Descriptives** | | | | | | |
| --- | --- | --- | --- | --- | --- | --- |
| Staph | | | | | | |
|  | N | Mean | Std. Deviation | Std. Error | 95% Confidence Interval for Mean | |
|  |  |  |  |  | Lower Bound | Upper Bound |
| 4 | 3 | 43.37000 | 2.620000 | 1.512658 | 36.86156 | 49.87844 |
| 6a | 3 | 68.39000 | 3.555151 | 2.052567 | 59.55851 | 77.22149 |
| 6b | 3 | 29.74333 | 2.135330 | 1.232833 | 24.43888 | 35.04779 |
| 6c | 3 | 16.26333 | 1.265003 | .730350 | 13.12089 | 19.40578 |
| 6d | 3 | 60.61000 | 3.548422 | 2.048683 | 51.79523 | 69.42477 |
| 6e | 3 | 56.44667 | 3.168996 | 1.829620 | 48.57445 | 64.31889 |
| 6f | 3 | 21.25000 | 1.837634 | 1.060959 | 16.68506 | 25.81494 |
| 8a | 3 | 35.61667 | 2.596311 | 1.498981 | 29.16707 | 42.06626 |
| 8b | 3 | 9.37667 | .715146 | .412890 | 7.60015 | 11.15319 |
| 8c | 3 | 28.89000 | 2.100071 | 1.212477 | 23.67313 | 34.10687 |
| 10 | 3 | 17.70000 | 1.420000 | .819837 | 14.17252 | 21.22748 |
| 11 | 3 | 78.37000 | 4.180000 | 2.413324 | 67.98630 | 88.75370 |
| 13b | 3 | 42.37000 | 2.530000 | 1.460696 | 36.08513 | 48.65487 |
| 13c | 3 | 7.87000 | .670000 | .386825 | 6.20563 | 9.53437 |
| erlotinib | 3 | 4.27333 | .260832 | .150591 | 3.62539 | 4.92128 |
| Total | 45 | 34.70267 | 22.667614 | 3.379088 | 27.89256 | 41.51277 |

| **Descriptives** | | |
| --- | --- | --- |
| Staph | | |
|  | Minimum | Maximum |
|  |  |  |
| 4 | 40.750 | 45.990 |
| 6a | 65.000 | 72.090 |
| 6b | 27.630 | 31.900 |
| 6c | 15.000 | 17.530 |
| 6d | 56.850 | 63.900 |
| 6e | 52.900 | 59.000 |
| 6f | 19.220 | 22.800 |
| 8a | 32.850 | 38.000 |
| 8b | 8.670 | 10.100 |
| 8c | 26.800 | 31.000 |
| 10 | 16.280 | 19.120 |
| 11 | 74.190 | 82.550 |
| 13b | 39.840 | 44.900 |
| 13c | 7.200 | 8.540 |
| erlotinib | 4.050 | 4.560 |
| Total | 4.050 | 82.550 |

| **ANOVA** | | | | | |
| --- | --- | --- | --- | --- | --- |
| Staph | | | | | |
|  | Sum of Squares | df | Mean Square | F | Sig. |
| Between Groups | 22428.626 | 14 | 1602.045 | 267.771 | .000 |
| Within Groups | 179.487 | 30 | 5.983 |  |  |
| Total | 22608.112 | 44 |  |  |  |

**Post Hoc Tests**

| **Multiple Comparisons** | | | | | | |
| --- | --- | --- | --- | --- | --- | --- |
| Dependent Variable: Staph  LSD | | | | | | |
| (I) test | (J) test | Mean Difference (I-J) | Std. Error | Sig. | 95% Confidence Interval | |
|  |  |  |  |  | Lower Bound | Upper Bound |
| 1 | 2 | -25.020000^*^ | 1.997146 | .000 | -29.09872 | -20.94128 |
|  | 3 | 13.626667^*^ | 1.997146 | .000 | 9.54795 | 17.70538 |
|  | 4 | 27.106667^*^ | 1.997146 | .000 | 23.02795 | 31.18538 |
|  | 5 | -17.240000^*^ | 1.997146 | .000 | -21.31872 | -13.16128 |
|  | 6 | -13.076667^*^ | 1.997146 | .000 | -17.15538 | -8.99795 |
|  | 7 | 22.120000^*^ | 1.997146 | .000 | 18.04128 | 26.19872 |
|  | 8 | 7.753333^*^ | 1.997146 | .001 | 3.67462 | 11.83205 |
|  | 9 | 33.993333^*^ | 1.997146 | .000 | 29.91462 | 38.07205 |
|  | 10 | 14.480000^*^ | 1.997146 | .000 | 10.40128 | 18.55872 |
|  | 11 | 25.670000^*^ | 1.997146 | .000 | 21.59128 | 29.74872 |
|  | 12 | -35.000000^*^ | 1.997146 | .000 | -39.07872 | -30.92128 |
|  | 13 | 1.000000 | 1.997146 | .620 | -3.07872 | 5.07872 |
|  | 14 | 35.500000^*^ | 1.997146 | .000 | 31.42128 | 39.57872 |
|  | 15 | 39.096667^*^ | 1.997146 | .000 | 35.01795 | 43.17538 |
| 2 | 1 | 25.020000^*^ | 1.997146 | .000 | 20.94128 | 29.09872 |
|  | 3 | 38.646667^*^ | 1.997146 | .000 | 34.56795 | 42.72538 |
|  | 4 | 52.126667^*^ | 1.997146 | .000 | 48.04795 | 56.20538 |
|  | 5 | 7.780000^*^ | 1.997146 | .001 | 3.70128 | 11.85872 |
|  | 6 | 11.943333^*^ | 1.997146 | .000 | 7.86462 | 16.02205 |
|  | 7 | 47.140000^*^ | 1.997146 | .000 | 43.06128 | 51.21872 |
|  | 8 | 32.773333^*^ | 1.997146 | .000 | 28.69462 | 36.85205 |
|  | 9 | 59.013333^*^ | 1.997146 | .000 | 54.93462 | 63.09205 |
|  | 10 | 39.500000^*^ | 1.997146 | .000 | 35.42128 | 43.57872 |
|  | 11 | 50.690000^*^ | 1.997146 | .000 | 46.61128 | 54.76872 |
|  | 12 | -9.980000^*^ | 1.997146 | .000 | -14.05872 | -5.90128 |
|  | 13 | 26.020000^*^ | 1.997146 | .000 | 21.94128 | 30.09872 |
|  | 14 | 60.520000^*^ | 1.997146 | .000 | 56.44128 | 64.59872 |
|  | 15 | 64.116667^*^ | 1.997146 | .000 | 60.03795 | 68.19538 |
| 3 | 1 | -13.626667^*^ | 1.997146 | .000 | -17.70538 | -9.54795 |
|  | 2 | -38.646667^*^ | 1.997146 | .000 | -42.72538 | -34.56795 |
|  | 4 | 13.480000^*^ | 1.997146 | .000 | 9.40128 | 17.55872 |
|  | 5 | -30.866667^*^ | 1.997146 | .000 | -34.94538 | -26.78795 |
|  | 6 | -26.703333^*^ | 1.997146 | .000 | -30.78205 | -22.62462 |

| **Multiple Comparisons** | | | | | | |
| --- | --- | --- | --- | --- | --- | --- |
| Dependent Variable: Staph  LSD | | | | | | |
| (I) test | (J) test | Mean Difference (I-J) | Std. Error | Sig. | 95% Confidence Interval | |
|  |  |  |  |  | Lower Bound | Upper Bound |
| 3 | 7 | 8.493333^*^ | 1.997146 | .000 | 4.41462 | 12.57205 |
|  | 8 | -5.873333^*^ | 1.997146 | .006 | -9.95205 | -1.79462 |
|  | 9 | 20.366667^*^ | 1.997146 | .000 | 16.28795 | 24.44538 |
|  | 10 | .853333^*^ | 1.997146 | .672 | -3.22538 | 4.93205 |
|  | 11 | 12.043333^*^ | 1.997146 | .000 | 7.96462 | 16.12205 |
|  | 12 | -48.626667^*^ | 1.997146 | .000 | -52.70538 | -44.54795 |
|  | 13 | -12.626667^*^ | 1.997146 | .000 | -16.70538 | -8.54795 |
|  | 14 | 21.873333^*^ | 1.997146 | .000 | 17.79462 | 25.95205 |
|  | 15 | 25.470000^*^ | 1.997146 | .000 | 21.39128 | 29.54872 |
| 4 | 1 | -27.106667^*^ | 1.997146 | .000 | -31.18538 | -23.02795 |
|  | 2 | -52.126667^*^ | 1.997146 | .000 | -56.20538 | -48.04795 |
|  | 3 | -13.480000 | 1.997146 | .000 | -17.55872 | -9.40128 |
|  | 5 | -44.346667^*^ | 1.997146 | .000 | -48.42538 | -40.26795 |
|  | 6 | -40.183333^*^ | 1.997146 | .000 | -44.26205 | -36.10462 |
|  | 7 | -4.986667^*^ | 1.997146 | .018 | -9.06538 | -.90795 |
|  | 8 | -19.353333^*^ | 1.997146 | .000 | -23.43205 | -15.27462 |
|  | 9 | 6.886667^*^ | 1.997146 | .002 | 2.80795 | 10.96538 |
|  | 10 | -12.626667^*^ | 1.997146 | .000 | -16.70538 | -8.54795 |
|  | 11 | -1.436667^*^ | 1.997146 | .477 | -5.51538 | 2.64205 |
|  | 12 | -62.106667^*^ | 1.997146 | .000 | -66.18538 | -58.02795 |
|  | 13 | -26.106667^*^ | 1.997146 | .000 | -30.18538 | -22.02795 |
|  | 14 | 8.393333^*^ | 1.997146 | .000 | 4.31462 | 12.47205 |
|  | 15 | 11.990000^*^ | 1.997146 | .000 | 7.91128 | 16.06872 |
| 5 | 1 | 17.240000^*^ | 1.997146 | .000 | 13.16128 | 21.31872 |
|  | 2 | -7.780000^*^ | 1.997146 | .001 | -11.85872 | -3.70128 |
|  | 3 | 30.866667^*^ | 1.997146 | .000 | 26.78795 | 34.94538 |
|  | 4 | 44.346667^*^ | 1.997146 | .000 | 40.26795 | 48.42538 |
|  | 6 | 4.163333^*^ | 1.997146 | .046 | .08462 | 8.24205 |
|  | 7 | 39.360000^*^ | 1.997146 | .000 | 35.28128 | 43.43872 |
|  | 8 | 24.993333^*^ | 1.997146 | .000 | 20.91462 | 29.07205 |
|  | 9 | 51.233333^*^ | 1.997146 | .000 | 47.15462 | 55.31205 |
|  | 10 | 31.720000^*^ | 1.997146 | .000 | 27.64128 | 35.79872 |
|  | 11 | 42.910000^*^ | 1.997146 | .000 | 38.83128 | 46.98872 |

| **Multiple Comparisons** | | | | | | |
| --- | --- | --- | --- | --- | --- | --- |
| Dependent Variable: Staph  LSD | | | | | | |
| (I) test | (J) test | Mean Difference (I-J) | Std. Error | Sig. | 95% Confidence Interval | |
|  |  |  |  |  | Lower Bound | Upper Bound |
| 5 | 12 | -17.760000^*^ | 1.997146 | .000 | -21.83872 | -13.68128 |
|  | 13 | 18.240000^*^ | 1.997146 | .000 | 14.16128 | 22.31872 |
|  | 14 | 52.740000^*^ | 1.997146 | .000 | 48.66128 | 56.81872 |
|  | 15 | 56.336667^*^ | 1.997146 | .000 | 52.25795 | 60.41538 |
| 6 | 1 | 13.076667^*^ | 1.997146 | .000 | 8.99795 | 17.15538 |
|  | 2 | -11.943333^*^ | 1.997146 | .000 | -16.02205 | -7.86462 |
|  | 3 | 26.703333^*^ | 1.997146 | .000 | 22.62462 | 30.78205 |
|  | 4 | 40.183333^*^ | 1.997146 | .000 | 36.10462 | 44.26205 |
|  | 5 | -4.163333^*^ | 1.997146 | .046 | -8.24205 | -.08462 |
|  | 7 | 35.196667^*^ | 1.997146 | .000 | 31.11795 | 39.27538 |
|  | 8 | 20.830000^*^ | 1.997146 | .000 | 16.75128 | 24.90872 |
|  | 9 | 47.070000 | 1.997146 | .000 | 42.99128 | 51.14872 |
|  | 10 | 27.556667^*^ | 1.997146 | .000 | 23.47795 | 31.63538 |
|  | 11 | 38.746667^*^ | 1.997146 | .000 | 34.66795 | 42.82538 |
|  | 12 | -21.923333^*^ | 1.997146 | .000 | -26.00205 | -17.84462 |
|  | 13 | 14.076667^*^ | 1.997146 | .000 | 9.99795 | 18.15538 |
|  | 14 | 48.576667^*^ | 1.997146 | .000 | 44.49795 | 52.65538 |
|  | 15 | 52.173333^*^ | 1.997146 | .000 | 48.09462 | 56.25205 |
| 7 | 1 | -22.120000^*^ | 1.997146 | .000 | -26.19872 | -18.04128 |
|  | 2 | -47.140000^*^ | 1.997146 | .000 | -51.21872 | -43.06128 |
|  | 3 | -8.493333^*^ | 1.997146 | .000 | -12.57205 | -4.41462 |
|  | 4 | 4.986667^*^ | 1.997146 | .018 | .90795 | 9.06538 |
|  | 5 | -39.360000^*^ | 1.997146 | .000 | -43.43872 | -35.28128 |
|  | 6 | -35.196667^*^ | 1.997146 | .000 | -39.27538 | -31.11795 |
|  | 8 | -14.366667^*^ | 1.997146 | .000 | -18.44538 | -10.28795 |
|  | 9 | 11.873333^*^ | 1.997146 | .000 | 7.79462 | 15.95205 |
|  | 10 | -7.640000^*^ | 1.997146 | .001 | -11.71872 | -3.56128 |
|  | 11 | 3.550000^*^ | 1.997146 | .086 | -.52872 | 7.62872 |
|  | 12 | -57.120000^*^ | 1.997146 | .000 | -61.19872 | -53.04128 |
|  | 13 | -21.120000^*^ | 1.997146 | .000 | -25.19872 | -17.04128 |
|  | 14 | 13.380000^*^ | 1.997146 | .000 | 9.30128 | 17.45872 |
|  | 15 | 16.976667^*^ | 1.997146 | .000 | 12.89795 | 21.05538 |
| 8 | 1 | -7.753333^*^ | 1.997146 | .001 | -11.83205 | -3.67462 |

| **Multiple Comparisons** | | | | | | |
| --- | --- | --- | --- | --- | --- | --- |
| Dependent Variable: Staph  LSD | | | | | | |
| (I) test | (J) test | Mean Difference (I-J) | Std. Error | Sig. | 95% Confidence Interval | |
|  |  |  |  |  | Lower Bound | Upper Bound |
| 8 | 2 | -32.773333^*^ | 1.997146 | .000 | -36.85205 | -28.69462 |
|  | 3 | 5.873333^*^ | 1.997146 | .006 | 1.79462 | 9.95205 |
|  | 4 | 19.353333^*^ | 1.997146 | .000 | 15.27462 | 23.43205 |
|  | 5 | -24.993333^*^ | 1.997146 | .000 | -29.07205 | -20.91462 |
|  | 6 | -20.830000^*^ | 1.997146 | .000 | -24.90872 | -16.75128 |
|  | 7 | 14.366667^*^ | 1.997146 | .000 | 10.28795 | 18.44538 |
|  | 9 | 26.240000^*^ | 1.997146 | .000 | 22.16128 | 30.31872 |
|  | 10 | 6.726667^*^ | 1.997146 | .002 | 2.64795 | 10.80538 |
|  | 11 | 17.916667^*^ | 1.997146 | .000 | 13.83795 | 21.99538 |
|  | 12 | -42.753333^*^ | 1.997146 | .000 | -46.83205 | -38.67462 |
|  | 13 | -6.753333^*^ | 1.997146 | .002 | -10.83205 | -2.67462 |
|  | 14 | 27.746667 | 1.997146 | .000 | 23.66795 | 31.82538 |
|  | 15 | 31.343333^*^ | 1.997146 | .000 | 27.26462 | 35.42205 |
| 9 | 1 | -33.993333^*^ | 1.997146 | .000 | -38.07205 | -29.91462 |
|  | 2 | -59.013333^*^ | 1.997146 | .000 | -63.09205 | -54.93462 |
|  | 3 | -20.366667^*^ | 1.997146 | .000 | -24.44538 | -16.28795 |
|  | 4 | -6.886667^*^ | 1.997146 | .002 | -10.96538 | -2.80795 |
|  | 5 | -51.233333^*^ | 1.997146 | .000 | -55.31205 | -47.15462 |
|  | 6 | -47.070000^*^ | 1.997146 | .000 | -51.14872 | -42.99128 |
|  | 7 | -11.873333^*^ | 1.997146 | .000 | -15.95205 | -7.79462 |
|  | 8 | -26.240000^*^ | 1.997146 | .000 | -30.31872 | -22.16128 |
|  | 10 | -19.513333^*^ | 1.997146 | .000 | -23.59205 | -15.43462 |
|  | 11 | -8.323333^*^ | 1.997146 | .000 | -12.40205 | -4.24462 |
|  | 12 | -68.993333^*^ | 1.997146 | .000 | -73.07205 | -64.91462 |
|  | 13 | -32.993333^*^ | 1.997146 | .000 | -37.07205 | -28.91462 |
|  | 14 | 1.506667^*^ | 1.997146 | .456 | -2.57205 | 5.58538 |
|  | 15 | 5.103333^*^ | 1.997146 | .016 | 1.02462 | 9.18205 |
| 10 | 1 | -14.480000^*^ | 1.997146 | .000 | -18.55872 | -10.40128 |
|  | 2 | -39.500000^*^ | 1.997146 | .000 | -43.57872 | -35.42128 |
|  | 3 | -.853333^*^ | 1.997146 | .672 | -4.93205 | 3.22538 |
|  | 4 | 12.626667^*^ | 1.997146 | .000 | 8.54795 | 16.70538 |
|  | 5 | -31.720000^*^ | 1.997146 | .000 | -35.79872 | -27.64128 |
|  | 6 | -27.556667^*^ | 1.997146 | .000 | -31.63538 | -23.47795 |

| **Multiple Comparisons** | | | | | | |
| --- | --- | --- | --- | --- | --- | --- |
| Dependent Variable: Staph  LSD | | | | | | |
| (I) test | (J) test | Mean Difference (I-J) | Std. Error | Sig. | 95% Confidence Interval | |
|  |  |  |  |  | Lower Bound | Upper Bound |
| 10 | 7 | 7.640000^*^ | 1.997146 | .001 | 3.56128 | 11.71872 |
|  | 8 | -6.726667^*^ | 1.997146 | .002 | -10.80538 | -2.64795 |
|  | 9 | 19.513333^*^ | 1.997146 | .000 | 15.43462 | 23.59205 |
|  | 11 | 11.190000^*^ | 1.997146 | .000 | 7.11128 | 15.26872 |
|  | 12 | -49.480000^*^ | 1.997146 | .000 | -53.55872 | -45.40128 |
|  | 13 | -13.480000^*^ | 1.997146 | .000 | -17.55872 | -9.40128 |
|  | 14 | 21.020000^*^ | 1.997146 | .000 | 16.94128 | 25.09872 |
|  | 15 | 24.616667^*^ | 1.997146 | .000 | 20.53795 | 28.69538 |
| 11 | 1 | -25.670000^*^ | 1.997146 | .000 | -29.74872 | -21.59128 |
|  | 2 | -50.690000^*^ | 1.997146 | .000 | -54.76872 | -46.61128 |
|  | 3 | -12.043333^*^ | 1.997146 | .000 | -16.12205 | -7.96462 |
|  | 4 | 1.436667 | 1.997146 | .477 | -2.64205 | 5.51538 |
|  | 5 | -42.910000^*^ | 1.997146 | .000 | -46.98872 | -38.83128 |
|  | 6 | -38.746667^*^ | 1.997146 | .000 | -42.82538 | -34.66795 |
|  | 7 | -3.550000^*^ | 1.997146 | .086 | -7.62872 | .52872 |
|  | 8 | -17.916667^*^ | 1.997146 | .000 | -21.99538 | -13.83795 |
|  | 9 | 8.323333^*^ | 1.997146 | .000 | 4.24462 | 12.40205 |
|  | 10 | -11.190000^*^ | 1.997146 | .000 | -15.26872 | -7.11128 |
|  | 12 | -60.670000^*^ | 1.997146 | .000 | -64.74872 | -56.59128 |
|  | 13 | -24.670000^*^ | 1.997146 | .000 | -28.74872 | -20.59128 |
|  | 14 | 9.830000^*^ | 1.997146 | .000 | 5.75128 | 13.90872 |
|  | 15 | 13.426667^*^ | 1.997146 | .000 | 9.34795 | 17.50538 |
| 12 | 1 | 35.000000^*^ | 1.997146 | .000 | 30.92128 | 39.07872 |
|  | 2 | 9.980000^*^ | 1.997146 | .000 | 5.90128 | 14.05872 |
|  | 3 | 48.626667^*^ | 1.997146 | .000 | 44.54795 | 52.70538 |
|  | 4 | 62.106667^*^ | 1.997146 | .000 | 58.02795 | 66.18538 |
|  | 5 | 17.760000^*^ | 1.997146 | .000 | 13.68128 | 21.83872 |
|  | 6 | 21.923333^*^ | 1.997146 | .000 | 17.84462 | 26.00205 |
|  | 7 | 57.120000^*^ | 1.997146 | .000 | 53.04128 | 61.19872 |
|  | 8 | 42.753333^*^ | 1.997146 | .000 | 38.67462 | 46.83205 |
|  | 9 | 68.993333^*^ | 1.997146 | .000 | 64.91462 | 73.07205 |
|  | 10 | 49.480000^*^ | 1.997146 | .000 | 45.40128 | 53.55872 |
|  | 11 | 60.670000^*^ | 1.997146 | .000 | 56.59128 | 64.74872 |

| **Multiple Comparisons** | | | | | | |
| --- | --- | --- | --- | --- | --- | --- |
| Dependent Variable: Staph  LSD | | | | | | |
| (I) test | (J) test | Mean Difference (I-J) | Std. Error | Sig. | 95% Confidence Interval | |
|  |  |  |  |  | Lower Bound | Upper Bound |
| 12 | 13 | 36.000000^*^ | 1.997146 | .000 | 31.92128 | 40.07872 |
|  | 14 | 70.500000^*^ | 1.997146 | .000 | 66.42128 | 74.57872 |
|  | 15 | 74.096667^*^ | 1.997146 | .000 | 70.01795 | 78.17538 |
| 13 | 1 | -1.000000^*^ | 1.997146 | .620 | -5.07872 | 3.07872 |
|  | 2 | -26.020000^*^ | 1.997146 | .000 | -30.09872 | -21.94128 |
|  | 3 | 12.626667^*^ | 1.997146 | .000 | 8.54795 | 16.70538 |
|  | 4 | 26.106667^*^ | 1.997146 | .000 | 22.02795 | 30.18538 |
|  | 5 | -18.240000^*^ | 1.997146 | .000 | -22.31872 | -14.16128 |
|  | 6 | -14.076667^*^ | 1.997146 | .000 | -18.15538 | -9.99795 |
|  | 7 | 21.120000^*^ | 1.997146 | .000 | 17.04128 | 25.19872 |
|  | 8 | 6.753333^*^ | 1.997146 | .002 | 2.67462 | 10.83205 |
|  | 9 | 32.993333 | 1.997146 | .000 | 28.91462 | 37.07205 |
|  | 10 | 13.480000^*^ | 1.997146 | .000 | 9.40128 | 17.55872 |
|  | 11 | 24.670000^*^ | 1.997146 | .000 | 20.59128 | 28.74872 |
|  | 12 | -36.000000^*^ | 1.997146 | .000 | -40.07872 | -31.92128 |
|  | 14 | 34.500000^*^ | 1.997146 | .000 | 30.42128 | 38.57872 |
|  | 15 | 38.096667^*^ | 1.997146 | .000 | 34.01795 | 42.17538 |
| 14 | 1 | -35.500000^*^ | 1.997146 | .000 | -39.57872 | -31.42128 |
|  | 2 | -60.520000^*^ | 1.997146 | .000 | -64.59872 | -56.44128 |
|  | 3 | -21.873333^*^ | 1.997146 | .000 | -25.95205 | -17.79462 |
|  | 4 | -8.393333^*^ | 1.997146 | .000 | -12.47205 | -4.31462 |
|  | 5 | -52.740000^*^ | 1.997146 | .000 | -56.81872 | -48.66128 |
|  | 6 | -48.576667^*^ | 1.997146 | .000 | -52.65538 | -44.49795 |
|  | 7 | -13.380000^*^ | 1.997146 | .000 | -17.45872 | -9.30128 |
|  | 8 | -27.746667^*^ | 1.997146 | .000 | -31.82538 | -23.66795 |
|  | 9 | -1.506667^*^ | 1.997146 | .456 | -5.58538 | 2.57205 |
|  | 10 | -21.020000^*^ | 1.997146 | .000 | -25.09872 | -16.94128 |
|  | 11 | -9.830000^*^ | 1.997146 | .000 | -13.90872 | -5.75128 |
|  | 12 | -70.500000^*^ | 1.997146 | .000 | -74.57872 | -66.42128 |
|  | 13 | -34.500000^*^ | 1.997146 | .000 | -38.57872 | -30.42128 |
|  | 15 | 3.596667^*^ | 1.997146 | .082 | -.48205 | 7.67538 |
| 15 | 1 | -39.096667^*^ | 1.997146 | .000 | -43.17538 | -35.01795 |
|  | 2 | -64.116667^*^ | 1.997146 | .000 | -68.19538 | -60.03795 |

| **Multiple Comparisons** | | | | | | |
| --- | --- | --- | --- | --- | --- | --- |
| Dependent Variable: Staph  LSD | | | | | | |
| (I) test | (J) test | Mean Difference (I-J) | Std. Error | Sig. | 95% Confidence Interval | |
|  |  |  |  |  | Lower Bound | Upper Bound |
| 15 | 3 | -25.470000^*^ | 1.997146 | .000 | -29.54872 | -21.39128 |
|  | 4 | -11.990000^*^ | 1.997146 | .000 | -16.06872 | -7.91128 |
|  | 5 | -56.336667^*^ | 1.997146 | .000 | -60.41538 | -52.25795 |
|  | 6 | -52.173333^*^ | 1.997146 | .000 | -56.25205 | -48.09462 |
|  | 7 | -16.976667^*^ | 1.997146 | .000 | -21.05538 | -12.89795 |
|  | 8 | -31.343333^*^ | 1.997146 | .000 | -35.42205 | -27.26462 |
|  | 9 | -5.103333^*^ | 1.997146 | .016 | -9.18205 | -1.02462 |
|  | 10 | -24.616667^*^ | 1.997146 | .000 | -28.69538 | -20.53795 |
|  | 11 | -13.426667^*^ | 1.997146 | .000 | -17.50538 | -9.34795 |
|  | 12 | -74.096667^*^ | 1.997146 | .000 | -78.17538 | -70.01795 |
|  | 13 | -38.096667^*^ | 1.997146 | .000 | -42.17538 | -34.01795 |
|  | 14 | -3.596667 | 1.997146 | .082 | -7.67538 | .48205 |

| *. The mean difference is significant at the 0.05 level. |
| --- |

HeLa

ONEWAY Staph BY test

/STATISTICS DESCRIPTIVES

/MISSING ANALYSIS

/POSTHOC=LSD ALPHA(0.05).

**One way**

| **Notes** | | |
| --- | --- | --- |
| Output Created | | 26-OCT-2025 01:14:10 |
| Comments | |  |
| Input | Data | C:\Users\dr\Desktop\my work.sav |
|  | Active Dataset | DataSet1 |
|  | Filter | <none> |
|  | Weight | <none> |
|  | Split File | <none> |
|  | N of Rows in Working Data File | 75 |
| Missing Value Handling | Definition of Missing | User-defined missing values are treated as missing. |
|  | Cases Used | Statistics for each analysis are based on cases with no missing data for any variable in the analysis. |
| Syntax | | ONEWAY Staph BY test  /STATISTICS DESCRIPTIVES  /MISSING ANALYSIS  /POSTHOC=LSD ALPHA(0.05). |
| Resources | Processor Time | 00:00:00.16 |
|  | Elapsed Time | 00:00:00.17 |

[DataSet1] C:\Users\dr\Desktop\my work.sav

| **Descriptives** | | | | | | |
| --- | --- | --- | --- | --- | --- | --- |
| Staph | | | | | | |
|  | N | Mean | Std. Deviation | Std. Error | 95% Confidence Interval for Mean | |
|  |  |  |  |  | Lower Bound | Upper Bound |
| 4 | 3 | 38.67000 | 2.520000 | 1.454923 | 32.40997 | 44.93003 |
| 6a | 3 | 58.79333 | 3.280081 | 1.893756 | 50.64516 | 66.94151 |
| 6b | 3 | 45.28000 | 2.810000 | 1.622354 | 38.29957 | 52.26043 |
| 6c | 3 | 23.80000 | 1.800000 | 1.039230 | 19.32855 | 28.27145 |
| 6d | 3 | 84.81000 | 4.510000 | 2.603850 | 73.60654 | 96.01346 |
| 6e | 3 | 49.33333 | 3.069484 | 1.772168 | 41.70831 | 56.95835 |
| 6f | 3 | 44.24000 | 2.600058 | 1.501144 | 37.78110 | 50.69890 |
| 8a | 3 | 27.34667 | 2.175554 | 1.256056 | 21.94229 | 32.75104 |
| 8b | 3 | 13.89000 | 1.005037 | .580259 | 11.39335 | 16.38665 |
| 8c | 3 | 38.05667 | 2.485485 | 1.434995 | 31.88238 | 44.23095 |
| 10 | 3 | 32.92000 | 2.205017 | 1.273067 | 27.44243 | 38.39757 |
| 11 | 3 | 63.03000 | 3.680000 | 2.124649 | 53.88837 | 72.17163 |
| 13b | 3 | 56.59000 | 3.295011 | 1.902376 | 48.40474 | 64.77526 |
| 13c | 3 | 23.69000 | 1.895020 | 1.094090 | 18.98251 | 28.39749 |
| erlotinib | 3 | 8.27000 | .384318 | .221886 | 7.31530 | 9.22470 |
| Total | 45 | 40.58133 | 19.886927 | 2.964568 | 34.60664 | 46.55603 |

| **Descriptives** | | |
| --- | --- | --- |
| Staph | | |
|  | Minimum | Maximum |
|  |  |  |
| 4 | 36.150 | 41.190 |
| 6a | 55.500 | 62.060 |
| 6b | 42.470 | 48.090 |
| 6c | 22.000 | 25.600 |
| 6d | 80.300 | 89.320 |
| 6e | 46.020 | 52.080 |
| 6f | 41.650 | 46.850 |
| 8a | 25.200 | 29.550 |
| 8b | 12.890 | 14.900 |
| 8c | 35.600 | 40.570 |
| 10 | 30.710 | 35.120 |
| 11 | 59.350 | 66.710 |
| 13b | 53.290 | 59.880 |
| 13c | 21.800 | 25.590 |
| erlotinib | 8.000 | 8.710 |
| Total | 8.000 | 89.320 |

| **ANOVA** | | | | | |
| --- | --- | --- | --- | --- | --- |
| Staph | | | | | |
|  | Sum of Squares | df | Mean Square | F | Sig. |
| Between Groups | 17182.176 | 14 | 1227.298 | 167.834 | .000 |
| Within Groups | 219.377 | 30 | 7.313 |  |  |
| Total | 17401.554 | 44 |  |  |  |

**Post Hoc Tests**

| **Multiple Comparisons** | | | | | | |
| --- | --- | --- | --- | --- | --- | --- |
| Dependent Variable: Staph  LSD | | | | | | |
| (I) test | (J) test | Mean Difference (I-J) | Std. Error | Sig. | 95% Confidence Interval | |
|  |  |  |  |  | Lower Bound | Upper Bound |
| 1 | 2 | -20.123333^*^ | 2.207953 | .000 | -24.63257 | -15.61409 |
|  | 3 | -6.610000^*^ | 2.207953 | .005 | -11.11924 | -2.10076 |
|  | 4 | 14.870000^*^ | 2.207953 | .000 | 10.36076 | 19.37924 |
|  | 5 | -46.140000^*^ | 2.207953 | .000 | -50.64924 | -41.63076 |
|  | 6 | -10.663333^*^ | 2.207953 | .000 | -15.17257 | -6.15409 |
|  | 7 | -5.570000^*^ | 2.207953 | .017 | -10.07924 | -1.06076 |
|  | 8 | 11.323333^*^ | 2.207953 | .000 | 6.81409 | 15.83257 |
|  | 9 | 24.780000^*^ | 2.207953 | .000 | 20.27076 | 29.28924 |
|  | 10 | .613333 | 2.207953 | .783 | -3.89591 | 5.12257 |
|  | 11 | 5.750000^*^ | 2.207953 | .014 | 1.24076 | 10.25924 |
|  | 12 | -24.360000^*^ | 2.207953 | .000 | -28.86924 | -19.85076 |
|  | 13 | -17.920000^*^ | 2.207953 | .000 | -22.42924 | -13.41076 |
|  | 14 | 14.980000^*^ | 2.207953 | .000 | 10.47076 | 19.48924 |
|  | 15 | 30.400000^*^ | 2.207953 | .000 | 25.89076 | 34.90924 |
| 2 | 1 | 20.123333^*^ | 2.207953 | .000 | 15.61409 | 24.63257 |
|  | 3 | 13.513333^*^ | 2.207953 | .000 | 9.00409 | 18.02257 |
|  | 4 | 34.993333^*^ | 2.207953 | .000 | 30.48409 | 39.50257 |
|  | 5 | -26.016667^*^ | 2.207953 | .000 | -30.52591 | -21.50743 |
|  | 6 | 9.460000^*^ | 2.207953 | .000 | 4.95076 | 13.96924 |
|  | 7 | 14.553333^*^ | 2.207953 | .000 | 10.04409 | 19.06257 |
|  | 8 | 31.446667^*^ | 2.207953 | .000 | 26.93743 | 35.95591 |
|  | 9 | 44.903333^*^ | 2.207953 | .000 | 40.39409 | 49.41257 |
|  | 10 | 20.736667^*^ | 2.207953 | .000 | 16.22743 | 25.24591 |
|  | 11 | 25.873333^*^ | 2.207953 | .000 | 21.36409 | 30.38257 |
|  | 12 | -4.236667 | 2.207953 | .065 | -8.74591 | .27257 |
|  | 13 | 2.203333 | 2.207953 | .326 | -2.30591 | 6.71257 |
|  | 14 | 35.103333^*^ | 2.207953 | .000 | 30.59409 | 39.61257 |
|  | 15 | 50.523333^*^ | 2.207953 | .000 | 46.01409 | 55.03257 |
| 3 | 1 | 6.610000^*^ | 2.207953 | .005 | 2.10076 | 11.11924 |
|  | 2 | -13.513333^*^ | 2.207953 | .000 | -18.02257 | -9.00409 |
|  | 4 | 21.480000^*^ | 2.207953 | .000 | 16.97076 | 25.98924 |
|  | 5 | -39.530000^*^ | 2.207953 | .000 | -44.03924 | -35.02076 |
|  | 6 | -4.053333 | 2.207953 | .076 | -8.56257 | .45591 |

| **Multiple Comparisons** | | | | | | |
| --- | --- | --- | --- | --- | --- | --- |
| Dependent Variable: Staph  LSD | | | | | | |
| (I) test | (J) test | Mean Difference (I-J) | Std. Error | Sig. | 95% Confidence Interval | |
|  |  |  |  |  | Lower Bound | Upper Bound |
| 3 | 7 | 1.040000^*^ | 2.207953 | .641 | -3.46924 | 5.54924 |
|  | 8 | 17.933333^*^ | 2.207953 | .000 | 13.42409 | 22.44257 |
|  | 9 | 31.390000^*^ | 2.207953 | .000 | 26.88076 | 35.89924 |
|  | 10 | 7.223333^*^ | 2.207953 | .003 | 2.71409 | 11.73257 |
|  | 11 | 12.360000^*^ | 2.207953 | .000 | 7.85076 | 16.86924 |
|  | 12 | -17.750000^*^ | 2.207953 | .000 | -22.25924 | -13.24076 |
|  | 13 | -11.310000^*^ | 2.207953 | .000 | -15.81924 | -6.80076 |
|  | 14 | 21.590000^*^ | 2.207953 | .000 | 17.08076 | 26.09924 |
|  | 15 | 37.010000 | 2.207953 | .000 | 32.50076 | 41.51924 |
| 4 | 1 | -14.870000^*^ | 2.207953 | .000 | -19.37924 | -10.36076 |
|  | 2 | -34.993333^*^ | 2.207953 | .000 | -39.50257 | -30.48409 |
|  | 3 | -21.480000^*^ | 2.207953 | .000 | -25.98924 | -16.97076 |
|  | 5 | -61.010000^*^ | 2.207953 | .000 | -65.51924 | -56.50076 |
|  | 6 | -25.533333^*^ | 2.207953 | .000 | -30.04257 | -21.02409 |
|  | 7 | -20.440000^*^ | 2.207953 | .000 | -24.94924 | -15.93076 |
|  | 8 | -3.546667^*^ | 2.207953 | .119 | -8.05591 | .96257 |
|  | 9 | 9.910000^*^ | 2.207953 | .000 | 5.40076 | 14.41924 |
|  | 10 | -14.256667^*^ | 2.207953 | .000 | -18.76591 | -9.74743 |
|  | 11 | -9.120000^*^ | 2.207953 | .000 | -13.62924 | -4.61076 |
|  | 12 | -39.230000^*^ | 2.207953 | .000 | -43.73924 | -34.72076 |
|  | 13 | -32.790000^*^ | 2.207953 | .000 | -37.29924 | -28.28076 |
|  | 14 | .110000^*^ | 2.207953 | .961 | -4.39924 | 4.61924 |
|  | 15 | 15.530000^*^ | 2.207953 | .000 | 11.02076 | 20.03924 |
| 5 | 1 | 46.140000^*^ | 2.207953 | .000 | 41.63076 | 50.64924 |
|  | 2 | 26.016667 | 2.207953 | .000 | 21.50743 | 30.52591 |
|  | 3 | 39.530000 | 2.207953 | .000 | 35.02076 | 44.03924 |
|  | 4 | 61.010000^*^ | 2.207953 | .000 | 56.50076 | 65.51924 |
|  | 6 | 35.476667^*^ | 2.207953 | .000 | 30.96743 | 39.98591 |
|  | 7 | 40.570000^*^ | 2.207953 | .000 | 36.06076 | 45.07924 |
|  | 8 | 57.463333^*^ | 2.207953 | .000 | 52.95409 | 61.97257 |
|  | 9 | 70.920000^*^ | 2.207953 | .000 | 66.41076 | 75.42924 |
|  | 10 | 46.753333^*^ | 2.207953 | .000 | 42.24409 | 51.26257 |
|  | 11 | 51.890000 | 2.207953 | .000 | 47.38076 | 56.39924 |

| **Multiple Comparisons** | | | | | | |
| --- | --- | --- | --- | --- | --- | --- |
| Dependent Variable: Staph  LSD | | | | | | |
| (I) test | (J) test | Mean Difference (I-J) | Std. Error | Sig. | 95% Confidence Interval | |
|  |  |  |  |  | Lower Bound | Upper Bound |
| 5 | 12 | 21.780000^*^ | 2.207953 | .000 | 17.27076 | 26.28924 |
|  | 13 | 28.220000^*^ | 2.207953 | .000 | 23.71076 | 32.72924 |
|  | 14 | 61.120000^*^ | 2.207953 | .000 | 56.61076 | 65.62924 |
|  | 15 | 76.540000^*^ | 2.207953 | .000 | 72.03076 | 81.04924 |
| 6 | 1 | 10.663333^*^ | 2.207953 | .000 | 6.15409 | 15.17257 |
|  | 2 | -9.460000^*^ | 2.207953 | .000 | -13.96924 | -4.95076 |
|  | 3 | 4.053333^*^ | 2.207953 | .076 | -.45591 | 8.56257 |
|  | 4 | 25.533333^*^ | 2.207953 | .000 | 21.02409 | 30.04257 |
|  | 5 | -35.476667 | 2.207953 | .000 | -39.98591 | -30.96743 |
|  | 7 | 5.093333^*^ | 2.207953 | .028 | .58409 | 9.60257 |
|  | 8 | 21.986667^*^ | 2.207953 | .000 | 17.47743 | 26.49591 |
|  | 9 | 35.443333^*^ | 2.207953 | .000 | 30.93409 | 39.95257 |
|  | 10 | 11.276667^*^ | 2.207953 | .000 | 6.76743 | 15.78591 |
|  | 11 | 16.413333^*^ | 2.207953 | .000 | 11.90409 | 20.92257 |
|  | 12 | -13.696667^*^ | 2.207953 | .000 | -18.20591 | -9.18743 |
|  | 13 | -7.256667^*^ | 2.207953 | .003 | -11.76591 | -2.74743 |
|  | 14 | 25.643333^*^ | 2.207953 | .000 | 21.13409 | 30.15257 |
|  | 15 | 41.063333^*^ | 2.207953 | .000 | 36.55409 | 45.57257 |
| 7 | 1 | 5.570000^*^ | 2.207953 | .017 | 1.06076 | 10.07924 |
|  | 2 | -14.553333^*^ | 2.207953 | .000 | -19.06257 | -10.04409 |
|  | 3 | -1.040000^*^ | 2.207953 | .641 | -5.54924 | 3.46924 |
|  | 4 | 20.440000^*^ | 2.207953 | .000 | 15.93076 | 24.94924 |
|  | 5 | -40.570000^*^ | 2.207953 | .000 | -45.07924 | -36.06076 |
|  | 6 | -5.093333^*^ | 2.207953 | .028 | -9.60257 | -.58409 |
|  | 8 | 16.893333 | 2.207953 | .000 | 12.38409 | 21.40257 |
|  | 9 | 30.350000 | 2.207953 | .000 | 25.84076 | 34.85924 |
|  | 10 | 6.183333^*^ | 2.207953 | .009 | 1.67409 | 10.69257 |
|  | 11 | 11.320000^*^ | 2.207953 | .000 | 6.81076 | 15.82924 |
|  | 12 | -18.790000^*^ | 2.207953 | .000 | -23.29924 | -14.28076 |
|  | 13 | -12.350000^*^ | 2.207953 | .000 | -16.85924 | -7.84076 |
|  | 14 | 20.550000^*^ | 2.207953 | .000 | 16.04076 | 25.05924 |
|  | 15 | 35.970000^*^ | 2.207953 | .000 | 31.46076 | 40.47924 |
| 8 | 1 | -11.323333 | 2.207953 | .000 | -15.83257 | -6.81409 |

| **Multiple Comparisons** | | | | | | |
| --- | --- | --- | --- | --- | --- | --- |
| Dependent Variable: Staph  LSD | | | | | | |
| (I) test | (J) test | Mean Difference (I-J) | Std. Error | Sig. | 95% Confidence Interval | |
|  |  |  |  |  | Lower Bound | Upper Bound |
| 8 | 2 | -31.446667^*^ | 2.207953 | .000 | -35.95591 | -26.93743 |
|  | 3 | -17.933333^*^ | 2.207953 | .000 | -22.44257 | -13.42409 |
|  | 4 | 3.546667^*^ | 2.207953 | .119 | -.96257 | 8.05591 |
|  | 5 | -57.463333^*^ | 2.207953 | .000 | -61.97257 | -52.95409 |
|  | 6 | -21.986667^*^ | 2.207953 | .000 | -26.49591 | -17.47743 |
|  | 7 | -16.893333^*^ | 2.207953 | .000 | -21.40257 | -12.38409 |
|  | 9 | 13.456667^*^ | 2.207953 | .000 | 8.94743 | 17.96591 |
|  | 10 | -10.710000^*^ | 2.207953 | .000 | -15.21924 | -6.20076 |
|  | 11 | -5.573333 | 2.207953 | .017 | -10.08257 | -1.06409 |
|  | 12 | -35.683333^*^ | 2.207953 | .000 | -40.19257 | -31.17409 |
|  | 13 | -29.243333^*^ | 2.207953 | .000 | -33.75257 | -24.73409 |
|  | 14 | 3.656667^*^ | 2.207953 | .108 | -.85257 | 8.16591 |
|  | 15 | 19.076667^*^ | 2.207953 | .000 | 14.56743 | 23.58591 |
| 9 | 1 | -24.780000^*^ | 2.207953 | .000 | -29.28924 | -20.27076 |
|  | 2 | -44.903333^*^ | 2.207953 | .000 | -49.41257 | -40.39409 |
|  | 3 | -31.390000^*^ | 2.207953 | .000 | -35.89924 | -26.88076 |
|  | 4 | -9.910000^*^ | 2.207953 | .000 | -14.41924 | -5.40076 |
|  | 5 | -70.920000^*^ | 2.207953 | .000 | -75.42924 | -66.41076 |
|  | 6 | -35.443333^*^ | 2.207953 | .000 | -39.95257 | -30.93409 |
|  | 7 | -30.350000^*^ | 2.207953 | .000 | -34.85924 | -25.84076 |
|  | 8 | -13.456667^*^ | 2.207953 | .000 | -17.96591 | -8.94743 |
|  | 10 | -24.166667^*^ | 2.207953 | .000 | -28.67591 | -19.65743 |
|  | 11 | -19.030000^*^ | 2.207953 | .000 | -23.53924 | -14.52076 |
|  | 12 | -49.140000^*^ | 2.207953 | .000 | -53.64924 | -44.63076 |
|  | 13 | -42.700000 | 2.207953 | .000 | -47.20924 | -38.19076 |
|  | 14 | -9.800000 | 2.207953 | .000 | -14.30924 | -5.29076 |
|  | 15 | 5.620000^*^ | 2.207953 | .016 | 1.11076 | 10.12924 |
| 10 | 1 | -.613333^*^ | 2.207953 | .783 | -5.12257 | 3.89591 |
|  | 2 | -20.736667^*^ | 2.207953 | .000 | -25.24591 | -16.22743 |
|  | 3 | -7.223333^*^ | 2.207953 | .003 | -11.73257 | -2.71409 |
|  | 4 | 14.256667^*^ | 2.207953 | .000 | 9.74743 | 18.76591 |
|  | 5 | -46.753333^*^ | 2.207953 | .000 | -51.26257 | -42.24409 |
|  | 6 | -11.276667 | 2.207953 | .000 | -15.78591 | -6.76743 |

| **Multiple Comparisons** | | | | | | |
| --- | --- | --- | --- | --- | --- | --- |
| Dependent Variable: Staph  LSD | | | | | | |
| (I) test | (J) test | Mean Difference (I-J) | Std. Error | Sig. | 95% Confidence Interval | |
|  |  |  |  |  | Lower Bound | Upper Bound |
| 10 | 7 | -6.183333^*^ | 2.207953 | .009 | -10.69257 | -1.67409 |
|  | 8 | 10.710000^*^ | 2.207953 | .000 | 6.20076 | 15.21924 |
|  | 9 | 24.166667^*^ | 2.207953 | .000 | 19.65743 | 28.67591 |
|  | 11 | 5.136667^*^ | 2.207953 | .027 | .62743 | 9.64591 |
|  | 12 | -24.973333^*^ | 2.207953 | .000 | -29.48257 | -20.46409 |
|  | 13 | -18.533333^*^ | 2.207953 | .000 | -23.04257 | -14.02409 |
|  | 14 | 14.366667^*^ | 2.207953 | .000 | 9.85743 | 18.87591 |
|  | 15 | 29.786667^*^ | 2.207953 | .000 | 25.27743 | 34.29591 |
| 11 | 1 | -5.750000 | 2.207953 | .014 | -10.25924 | -1.24076 |
|  | 2 | -25.873333^*^ | 2.207953 | .000 | -30.38257 | -21.36409 |
|  | 3 | -12.360000^*^ | 2.207953 | .000 | -16.86924 | -7.85076 |
|  | 4 | 9.120000^*^ | 2.207953 | .000 | 4.61076 | 13.62924 |
|  | 5 | -51.890000^*^ | 2.207953 | .000 | -56.39924 | -47.38076 |
|  | 6 | -16.413333^*^ | 2.207953 | .000 | -20.92257 | -11.90409 |
|  | 7 | -11.320000^*^ | 2.207953 | .000 | -15.82924 | -6.81076 |
|  | 8 | 5.573333^*^ | 2.207953 | .017 | 1.06409 | 10.08257 |
|  | 9 | 19.030000^*^ | 2.207953 | .000 | 14.52076 | 23.53924 |
|  | 10 | -5.136667^*^ | 2.207953 | .027 | -9.64591 | -.62743 |
|  | 12 | -30.110000^*^ | 2.207953 | .000 | -34.61924 | -25.60076 |
|  | 13 | -23.670000^*^ | 2.207953 | .000 | -28.17924 | -19.16076 |
|  | 14 | 9.230000^*^ | 2.207953 | .000 | 4.72076 | 13.73924 |
|  | 15 | 24.650000^*^ | 2.207953 | .000 | 20.14076 | 29.15924 |
| 12 | 1 | 24.360000^*^ | 2.207953 | .000 | 19.85076 | 28.86924 |
|  | 2 | 4.236667^*^ | 2.207953 | .065 | -.27257 | 8.74591 |
|  | 3 | 17.750000 | 2.207953 | .000 | 13.24076 | 22.25924 |
|  | 4 | 39.230000 | 2.207953 | .000 | 34.72076 | 43.73924 |
|  | 5 | -21.780000^*^ | 2.207953 | .000 | -26.28924 | -17.27076 |
|  | 6 | 13.696667^*^ | 2.207953 | .000 | 9.18743 | 18.20591 |
|  | 7 | 18.790000^*^ | 2.207953 | .000 | 14.28076 | 23.29924 |
|  | 8 | 35.683333^*^ | 2.207953 | .000 | 31.17409 | 40.19257 |
|  | 9 | 49.140000^*^ | 2.207953 | .000 | 44.63076 | 53.64924 |
|  | 10 | 24.973333^*^ | 2.207953 | .000 | 20.46409 | 29.48257 |
|  | 11 | 30.110000 | 2.207953 | .000 | 25.60076 | 34.61924 |

| **Multiple Comparisons** | | | | | | |
| --- | --- | --- | --- | --- | --- | --- |
| Dependent Variable: Staph  LSD | | | | | | |
| (I) test | (J) test | Mean Difference (I-J) | Std. Error | Sig. | 95% Confidence Interval | |
|  |  |  |  |  | Lower Bound | Upper Bound |
| 12 | 13 | 6.440000^*^ | 2.207953 | .007 | 1.93076 | 10.94924 |
|  | 14 | 39.340000^*^ | 2.207953 | .000 | 34.83076 | 43.84924 |
|  | 15 | 54.760000^*^ | 2.207953 | .000 | 50.25076 | 59.26924 |
| 13 | 1 | 17.920000^*^ | 2.207953 | .000 | 13.41076 | 22.42924 |
|  | 2 | -2.203333^*^ | 2.207953 | .326 | -6.71257 | 2.30591 |
|  | 3 | 11.310000^*^ | 2.207953 | .000 | 6.80076 | 15.81924 |
|  | 4 | 32.790000^*^ | 2.207953 | .000 | 28.28076 | 37.29924 |
|  | 5 | -28.220000^*^ | 2.207953 | .000 | -32.72924 | -23.71076 |
|  | 6 | 7.256667 | 2.207953 | .003 | 2.74743 | 11.76591 |
|  | 7 | 12.350000^*^ | 2.207953 | .000 | 7.84076 | 16.85924 |
|  | 8 | 29.243333^*^ | 2.207953 | .000 | 24.73409 | 33.75257 |
|  | 9 | 42.700000^*^ | 2.207953 | .000 | 38.19076 | 47.20924 |
|  | 10 | 18.533333^*^ | 2.207953 | .000 | 14.02409 | 23.04257 |
|  | 11 | 23.670000^*^ | 2.207953 | .000 | 19.16076 | 28.17924 |
|  | 12 | -6.440000^*^ | 2.207953 | .007 | -10.94924 | -1.93076 |
|  | 14 | 32.900000^*^ | 2.207953 | .000 | 28.39076 | 37.40924 |
|  | 15 | 48.320000^*^ | 2.207953 | .000 | 43.81076 | 52.82924 |
| 14 | 1 | -14.980000^*^ | 2.207953 | .000 | -19.48924 | -10.47076 |
|  | 2 | -35.103333^*^ | 2.207953 | .000 | -39.61257 | -30.59409 |
|  | 3 | -21.590000^*^ | 2.207953 | .000 | -26.09924 | -17.08076 |
|  | 4 | -.110000^*^ | 2.207953 | .961 | -4.61924 | 4.39924 |
|  | 5 | -61.120000^*^ | 2.207953 | .000 | -65.62924 | -56.61076 |
|  | 6 | -25.643333^*^ | 2.207953 | .000 | -30.15257 | -21.13409 |
|  | 7 | -20.550000^*^ | 2.207953 | .000 | -25.05924 | -16.04076 |
|  | 8 | -3.656667 | 2.207953 | .108 | -8.16591 | .85257 |
|  | 9 | 9.800000 | 2.207953 | .000 | 5.29076 | 14.30924 |
|  | 10 | -14.366667^*^ | 2.207953 | .000 | -18.87591 | -9.85743 |
|  | 11 | -9.230000^*^ | 2.207953 | .000 | -13.73924 | -4.72076 |
|  | 12 | -39.340000^*^ | 2.207953 | .000 | -43.84924 | -34.83076 |
|  | 13 | -32.900000^*^ | 2.207953 | .000 | -37.40924 | -28.39076 |
|  | 15 | 15.420000^*^ | 2.207953 | .000 | 10.91076 | 19.92924 |
| 15 | 1 | -30.400000^*^ | 2.207953 | .000 | -34.90924 | -25.89076 |
|  | 2 | -50.523333 | 2.207953 | .000 | -55.03257 | -46.01409 |

| **Multiple Comparisons** | | | | | | |
| --- | --- | --- | --- | --- | --- | --- |
| Dependent Variable: Staph  LSD | | | | | | |
| (I) test | (J) test | Mean Difference (I-J) | Std. Error | Sig. | 95% Confidence Interval | |
|  |  |  |  |  | Lower Bound | Upper Bound |
| 15 | 3 | -37.010000^*^ | 2.207953 | .000 | -41.51924 | -32.50076 |
|  | 4 | -15.530000^*^ | 2.207953 | .000 | -20.03924 | -11.02076 |
|  | 5 | -76.540000^*^ | 2.207953 | .000 | -81.04924 | -72.03076 |
|  | 6 | -41.063333^*^ | 2.207953 | .000 | -45.57257 | -36.55409 |
|  | 7 | -35.970000^*^ | 2.207953 | .000 | -40.47924 | -31.46076 |
|  | 8 | -19.076667^*^ | 2.207953 | .000 | -23.58591 | -14.56743 |
|  | 9 | -5.620000^*^ | 2.207953 | .016 | -10.12924 | -1.11076 |
|  | 10 | -29.786667^*^ | 2.207953 | .000 | -34.29591 | -25.27743 |
|  | 11 | -24.650000 | 2.207953 | .000 | -29.15924 | -20.14076 |
|  | 12 | -54.760000^*^ | 2.207953 | .000 | -59.26924 | -50.25076 |
|  | 13 | -48.320000^*^ | 2.207953 | .000 | -52.82924 | -43.81076 |
|  | 14 | -15.420000^*^ | 2.207953 | .000 | -19.92924 | -10.91076 |

| *. The mean difference is significant at the 0.05 level. |
| --- |

WI38

ONEWAY Staph BY test

/STATISTICS DESCRIPTIVES

/MISSING ANALYSIS

/POSTHOC=LSD ALPHA(0.05).

**One way**

| **Notes** | | |
| --- | --- | --- |
| Output Created | | 26-OCT-2025 22:35:01 |
| Comments | |  |
| Input | Data | C:\Users\dr\Desktop\my work.sav |
|  | Active Dataset | DataSet1 |
|  | Filter | <none> |
|  | Weight | <none> |
|  | Split File | <none> |
|  | N of Rows in Working Data File | 60 |
| Missing Value Handling | Definition of Missing | User-defined missing values are treated as missing. |
|  | Cases Used | Statistics for each analysis are based on cases with no missing data for any variable in the analysis. |
| Syntax | | ONEWAY Staph BY test  /STATISTICS DESCRIPTIVES  /MISSING ANALYSIS  /POSTHOC=LSD ALPHA(0.05). |
| Resources | Processor Time | 00:00:00.03 |
|  | Elapsed Time | 00:00:00.03 |

[DataSet1] C:\Users\dr\Desktop\my work. sav

| **Descriptives** | | | | | | |
| --- | --- | --- | --- | --- | --- | --- |
| Staph | | | | | | |
|  | N | Mean | Std. Deviation | Std. Error | 95% Confidence Interval for Mean | |
|  |  |  |  |  | Lower Bound | Upper Bound |
| 6c | 3 | 47.92000 | 2.756592 | 1.591519 | 41.07225 | 54.76775 |
| 8b | 3 | 61.48000 | 3.643789 | 2.103743 | 52.42833 | 70.53167 |
| 10 | 3 | 46.40667 | 3.861105 | 2.229210 | 36.81515 | 55.99818 |
| 13c | 3 | 59.78333 | 3.246665 | 1.874463 | 51.71817 | 67.84850 |
| Total | 12 | 53.89750 | 7.655012 | 2.209811 | 49.03374 | 58.76126 |

| **Descriptives** | | | | | | | | |
| --- | --- | --- | --- | --- | --- | --- | --- | --- |
| Staph | | | | | | | | |
|  | | Minimum | | | | Maximum | | |
|  |  |  |  |  |  |  |  |  |
| 6c | | 45.280 | | | | 50.780 | | |
| 8b | | 58.100 | | | | 65.340 | | |
| 10 | | 42.600 | | | | 50.320 | | |
| 13c | | 57.500 | | | | 63.500 | | |
| Total | | 42.600 | | | | 65.340 | | |
| **ANOVA** | | | | | | | |  |
| Staph | | | | | | | |  |
|  | Sum of Squares | | df | Mean Square | F | | Sig. |  |
| Between Groups | 551.941 | | 3 | 183.980 | 15.886 | | .001 |  |
| Within Groups | 92.650 | | 8 | 11.581 |  | |  |  |
| Total | 644.591 | | 11 |  |  | |  |  |

**Post Hoc Tests**

| **Multiple Comparisons** | | | | | | |
| --- | --- | --- | --- | --- | --- | --- |
| Dependent Variable: Staph  LSD | | | | | | |
| (I) test | (J) test | Mean Difference (I-J) | Std. Error | Sig. | 95% Confidence Interval | |
|  |  |  |  |  | Lower Bound | Upper Bound |
| 1 | 2 | -13.560000^*^ | 2.778638 | .001 | -19.96755 | -7.15245 |
|  | 3 | 1.513333 | 2.778638 | .601 | -4.89422 | 7.92088 |
|  | 4 | -11.863333^*^ | 2.778638 | .003 | -18.27088 | -5.45578 |
| 2 | 1 | 13.560000^*^ | 2.778638 | .001 | 7.15245 | 19.96755 |
|  | 3 | 15.073333^*^ | 2.778638 | .001 | 8.66578 | 21.48088 |
|  | 4 | 1.696667 | 2.778638 | .558 | -4.71088 | 8.10422 |
| 3 | 1 | -1.513333 | 2.778638 | .601 | -7.92088 | 4.89422 |
|  | 2 | -15.073333^*^ | 2.778638 | .001 | -21.48088 | -8.66578 |
|  | 4 | -13.376667^*^ | 2.778638 | .001 | -19.78422 | -6.96912 |
| 4 | 1 | 11.863333^*^ | 2.778638 | .003 | 5.45578 | 18.27088 |
|  | 2 | -1.696667 | 2.778638 | .558 | -8.10422 | 4.71088 |
|  | 3 | 13.376667^*^ | 2.778638 | .001 | 6.96912 | 19.78422 |

| *. The mean difference is significant at the 0.05 level. |
| --- |

**Lab Report**

| **ser** | **Compound** | | EGFR_T790M | **SD**  ± |
| --- | --- | --- | --- | --- |
|  | **code** | **MW**  **g/mol** | **IC50**  **ug/ml** |  |
| 1 | **6c** | --- | **0.493** | 0.02 |
| 2 | **8b** | --- | **0.151** | 0.006 |
| 3 | **10** | --- | **0.19** | 0.008 |
| 4 | **13c** | --- | **0.376** | 0.015 |
| *** | **Erlotenib** | --- | **0.049** | 0.002 |

| **ser** | **Compound** | | **Her2** | **SD**  ± |
| --- | --- | --- | --- | --- |
|  | **code** | **MW**  **g/mol** | **IC50**  **ug/ml** |  |
| 1 | **6c** | --- | **0.186** | 0.011 |
| 2 | **8b** | --- | **0.075** | 0.005 |
| 3 | **10** | --- | **0.091** | 0.006 |
| 4 | **13c** | --- | **0.204** | 0.012 |
| *** | **Erlotenib** | --- | **0.034** | 0.002 |

**Detailed Results**

| **EGFR_T790M** | |  |  |  |  |  |  |  |  |  |  |  |
| --- | --- | --- | --- | --- | --- | --- | --- | --- | --- | --- | --- | --- |
| code | IC50 | conc | log | %inh | T2 | T1 | ∆T | RFU2 | RFU1 | ∆RFU | slope | K.Activity |
| 6c |  | 100 | 2 | 91 | 30 | 0 | 30 | 9.28 | 0 | 9.28 | 3.33333 | 11.13601 |
|  |  | 10 | 1 | 83 | 30 | 0 | 30 | 17.29 | 0 | 17.29 | 3.33333 | 20.74802 |
| 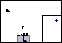   \|  \| \| --- \| |  | 1 | 0 | 57 | 30 | 0 | 30 | 42.55 | 0 | 42.55 | 3.33333 | 51.06005 |
|  |  | 0.1 | -1 | 31 | 30 | 0 | 30 | 69.38 | 0 | 69.38 | 3.33333 | 83.25608 |
|  |  | 0.01 | -2 | 19 | 30 | 0 | 30 | 81.36 | 0 | 81.36 | 3.33333 | 97.6321 |
| EC |  |  |  | 0 | 30 | 0 | 30 | 100 | 0 | 100 | 3.33333 | 120 |
|  |  |  |  |  |  |  |  |  |  |  |  |  |
| code | IC50 | conc | log | %inh | T2 | T1 | ∆T | RFU2 | RFU1 | ∆RFU | slope | K.Activity |
| 8b |  | 100 | 2 | 94 | 30 | 0 | 30 | 5.77 | 0 | 5.77 | 3.33333 | 6.924007 |
| 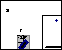   \|  \| \| --- \| |  | 10 | 1 | 89 | 30 | 0 | 30 | 11.39 | 0 | 11.39 | 3.33333 | 13.66801 |
|  |  | 1 | 0 | 73 | 30 | 0 | 30 | 27.16 | 0 | 27.16 | 3.33333 | 32.59203 |
|  |  | 0.1 | -1 | 41 | 30 | 0 | 30 | 59.41 | 0 | 59.41 | 3.33333 | 71.29207 |
|  |  | 0.01 | -2 | 28 | 30 | 0 | 30 | 72.11 | 0 | 72.11 | 3.33333 | 86.53209 |
| EC |  |  |  | 0 | 30 | 0 | 30 | 100 | 0 | 100 | 3.33333 | 120 |
|  |  |  |  |  |  |  |  |  |  |  |  |  |
| code | IC50 | conc | log | %inh | T2 | T1 | ∆T | RFU2 | RFU1 | ∆RFU | slope | K.Activity |
| 10 |  | 100 | 2 | 93 | 30 | 0 | 30 | 7.47 | 0 | 7.47 | 3.33333 | 8.964009 |
|  |  | 10 | 1 | 87 | 30 | 0 | 30 | 13.26 | 0 | 13.26 | 3.33333 | 15.91202 |
| 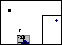   \|  \| \| --- \| |  | 1 | 0 | 69 | 30 | 0 | 30 | 31.09 | 0 | 31.09 | 3.33333 | 37.30804 |
|  |  | 0.1 | -1 | 42 | 30 | 0 | 30 | 58.16 | 0 | 58.16 | 3.33333 | 69.79207 |
|  |  | 0.01 | -2 | 25 | 30 | 0 | 30 | 75.13 | 0 | 75.13 | 3.33333 | 90.15609 |
| EC |  |  |  | 0 | 30 | 0 | 30 | 100 | 0 | 100 | 3.33333 | 120 |
|  |  |  |  |  |  |  |  |  |  |  |  |  |
| code | IC50 | conc | log | %inh | T2 | T1 | ∆T | RFU2 | RFU1 | ∆RFU | slope | K.Activity |
| 13c |  | 100 | 2 | 91 | 30 | 0 | 30 | 9.12 | 0 | 9.12 | 3.33333 | 10.94401 |
| 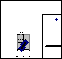   \|  \| \| --- \| |  | 10 | 1 | 82 | 30 | 0 | 30 | 17.62 | 0 | 17.62 | 3.33333 | 21.14402 |
|  |  | 1 | 0 | 67 | 30 | 0 | 30 | 32.82 | 0 | 32.82 | 3.33333 | 39.38404 |
|  |  | 0.1 | -1 | 33 | 30 | 0 | 30 | 66.95 | 0 | 66.95 | 3.33333 | 80.34008 |
|  |  | 0.01 | -2 | 18 | 30 | 0 | 30 | 82.02 | 0 | 82.02 | 3.33333 | 98.4241 |
| EC |  |  |  | 0 | 30 | 0 | 30 | 100 | 0 | 100 | 3.33333 | 120 |
|  |  |  |  |  |  |  |  |  |  |  |  |  |
| code | IC50 | conc | log | %inh | T2 | T1 | ∆T | RFU2 | RFU1 | ∆RFU | slope | K.Activity |
| 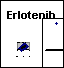   \| **Erlotenib** \| \| --- \| |  | 100 | 2 | 95 | 30 | 0 | 30 | 5.45 | 0 | 5.45 | 3.33333 | 6.540007 |
|  |  | 10 | 1 | 89 | 30 | 0 | 30 | 11.02 | 0 | 11.02 | 3.33333 | 13.22401 |
|  |  | 1 | 0 | 76 | 30 | 0 | 30 | 23.51 | 0 | 23.51 | 3.33333 | 28.21203 |
|  |  | 0.1 | -1 | 51 | 30 | 0 | 30 | 49.12 | 0 | 49.12 | 3.33333 | 58.94406 |
|  |  | 0.01 | -2 | 38 | 30 | 0 | 30 | 62.42 | 0 | 62.42 | 3.33333 | 74.90407 |
| EC |  |  |  | 0 | 30 | 0 | 30 | 100 | 0 | 100 | 3.33333 | 120 |
|  |  |  |  |  |  |  |  |  |  |  |  |  |

| 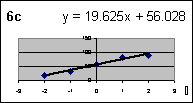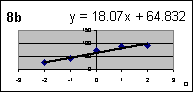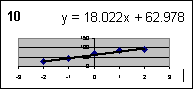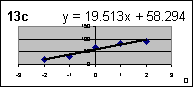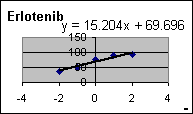   \|  \| \| --- \| |  |  |
| --- | --- | --- | --- |
|  |  |  |
|  |  |  |
|  |  |  |
|  |  |  |
|  |  |  |
|  |  |  |
|  |  |  |
|  |  |  |
|  |  |  |
|  |  |  |
|  |  |  |
|  |  |  |

**Detailed Results**

| **Her2** | |  |  |  |  |  |  |  |  |  |  |  |
| --- | --- | --- | --- | --- | --- | --- | --- | --- | --- | --- | --- | --- |
| code | IC50 | conc | log | %inh | T2 | T1 | ∆T | RFU2 | RFU1 | ∆RFU | slope | K.Activity |
| 6c |  | 100 | 2 | 93 | 30 | 0 | 30 | 6.85 | 0 | 6.85 | 3.33333 | 8.220008 |
|  |  | 10 | 1 | 85 | 30 | 0 | 30 | 15.22 | 0 | 15.22 | 3.33333 | 18.26402 |
| 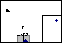 |  | 1 | 0 | 65 | 30 | 0 | 30 | 35.28 | 0 | 35.28 | 3.33333 | 42.33604 |
|  |  | 0.1 | -1 | 43 | 30 | 0 | 30 | 56.91 | 0 | 56.91 | 3.33333 | 68.29207 |
|  |  | 0.01 | -2 | 28 | 30 | 0 | 30 | 72.48 | 0 | 72.48 | 3.33333 | 86.97609 |
| EC |  |  |  | 0 | 30 | 0 | 30 | 100 | 0 | 100 | 3.33333 | 120 |
|  |  |  |  |  |  |  |  |  |  |  |  |  |
| code | IC50 | conc | log | %inh | T2 | T1 | ∆T | RFU2 | RFU1 | ∆RFU | slope | K.Activity |
| 8b |  | 100 | 2 | 94 | 30 | 0 | 30 | 6.07 | 0 | 6.07 | 3.33333 | 7.284007 |
| 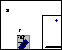 |  | 10 | 1 | 88 | 30 | 0 | 30 | 11.83 | 0 | 11.83 | 3.33333 | 14.19601 |
|  |  | 1 | 0 | 73 | 30 | 0 | 30 | 26.55 | 0 | 26.55 | 3.33333 | 31.86003 |
|  |  | 0.1 | -1 | 48 | 30 | 0 | 30 | 52.02 | 0 | 52.02 | 3.33333 | 62.42406 |
|  |  | 0.01 | -2 | 35 | 30 | 0 | 30 | 64.84 | 0 | 64.84 | 3.33333 | 77.80808 |
| EC |  |  |  | 0 | 30 | 0 | 30 | 100 | 0 | 100 | 3.33333 | 120 |
|  |  |  |  |  |  |  |  |  |  |  |  |  |
| code | IC50 | conc | log | %inh | T2 | T1 | ∆T | RFU2 | RFU1 | ∆RFU | slope | K.Activity |
| 10 |  | 100 | 2 | 94 | 30 | 0 | 30 | 6.26 | 0 | 6.26 | 3.33333 | 7.512008 |
|  |  | 10 | 1 | 88 | 30 | 0 | 30 | 11.51 | 0 | 11.51 | 3.33333 | 13.81201 |
| 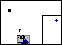 |  | 1 | 0 | 77 | 30 | 0 | 30 | 22.93 | 0 | 22.93 | 3.33333 | 27.51603 |
|  |  | 0.1 | -1 | 48 | 30 | 0 | 30 | 51.61 | 0 | 51.61 | 3.33333 | 61.93206 |
|  |  | 0.01 | -2 | 32 | 30 | 0 | 30 | 68.19 | 0 | 68.19 | 3.33333 | 81.82808 |
| EC |  |  |  | 0 | 30 | 0 | 30 | 100 | 0 | 100 | 3.33333 | 120 |
|  |  |  |  |  |  |  |  |  |  |  |  |  |
| code | IC50 | conc | log | %inh | T2 | T1 | ∆T | RFU2 | RFU1 | ∆RFU | slope | K.Activity |
| 13c |  | 100 | 2 | 92 | 30 | 0 | 30 | 8.13 | 0 | 8.13 | 3.33333 | 9.75601 |
| 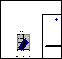 |  | 10 | 1 | 87 | 30 | 0 | 30 | 12.96 | 0 | 12.96 | 3.33333 | 15.55202 |
|  |  | 1 | 0 | 73 | 30 | 0 | 30 | 26.72 | 0 | 26.72 | 3.33333 | 32.06403 |
|  |  | 0.1 | -1 | 41 | 30 | 0 | 30 | 59.17 | 0 | 59.17 | 3.33333 | 71.00407 |
|  |  | 0.01 | -2 | 21 | 30 | 0 | 30 | 78.55 | 0 | 78.55 | 3.33333 | 94.26009 |
| EC |  |  |  | 0 | 30 | 0 | 30 | 100 | 0 | 100 | 3.33333 | 120 |
|  |  |  |  |  |  |  |  |  |  |  |  |  |
| code | IC50 | conc | log | %inh | T2 | T1 | ∆T | RFU2 | RFU1 | ∆RFU | slope | K.Activity |
| **Erlotenib** |  | 100 | 2 | 94 | 30 | 0 | 30 | 5.86 | 0 | 5.86 | 3.33333 | 7.032007 |
| 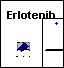 |  | 10 | 1 | 90 | 30 | 0 | 30 | 9.88 | 0 | 9.88 | 3.33333 | 11.85601 |
|  |  | 1 | 0 | 75 | 30 | 0 | 30 | 24.73 | 0 | 24.73 | 3.33333 | 29.67603 |
|  |  | 0.1 | -1 | 55 | 30 | 0 | 30 | 44.82 | 0 | 44.82 | 3.33333 | 53.78405 |
|  |  | 0.01 | -2 | 40 | 30 | 0 | 30 | 59.72 | 0 | 59.72 | 3.33333 | 71.66407 |
| EC |  |  |  | 0 | 30 | 0 | 30 | 100 | 0 | 100 | 3.33333 | 120 |
|  |  |  |  |  |  |  |  |  |  |  |  |  |


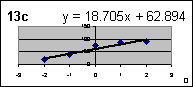

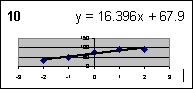

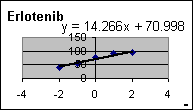


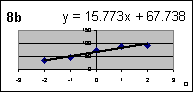

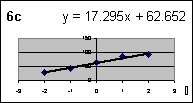


**Lab Report**

| **ser** | **Sample** | | **DNA content** | | | |  |
| --- | --- | --- | --- | --- | --- | --- | --- |
|  | **code** | **IC50**  **uM** | **%G0-G1** | **%S** | **%G2/M** | **Comment** |  |
| **1** | **8b/ MCF7** | 9.38 | **61.02** | **34.51** | **4.47** | cell growth arrest@ G1/S |  |
| **2** | **10/ MCF7** | 17.7 | **52.64** | **39.33** | **8.03** | cell growth arrest@ S |  |
| **3** | **Cont. MCF7** | **---** | **58.03** | **29.51** | **12.46** | --- |  |

| **s** | **code** | **conc** | **Apoptosis** | | | **Necrosis** |
| --- | --- | --- | --- | --- | --- | --- |
|  |  |  | Total | Early | Late |  |
| 1 | **8b/ MCF7** | --- | **44.06** | **29.16** | **9.83** | **5.07** |
| 2 | **10/ MCF7** | --- | **32.57** | **21.41** | **7.28** | **3.88** |
| 3 | **Cont. MCF7** | --- | **2.19** | **0.61** | **0.14** | **1.44** |

**Bcl2 Elisa**

| **group** | **protein quantification** | **O.D**  **(R1)** | **O.D**  **(R2)** | **O.D**  **(R3)** | **Aver. abs** | **Log**  **(aver. abs)** | **conc.**  **(ng/mg)** | **conc.**  **(ng/mg/protein)** |
| --- | --- | --- | --- | --- | --- | --- | --- | --- |
| Untreat/MCF7 | 0.61 | 1.92 | 1.99 | 2.07 | 1.99 | 0.445952 | 2.79 | 4.57 |
| Treated **8b**/MCF7 | 0.63 | 1.87 | 1.95 | 1.99 | 1.93 | 0.432367 | 2.70 | 4.29 |
| treated **10**/MCF7 | 0.6 | 1.83 | 2.06 | 1.79 | 1.89 | 0.423073 | 2.64 | 4.41 |

**Standard curve**

| conc | abs | log conc | log abs |
| --- | --- | --- | --- |
| 0.156 | 0.13 | -0.80688 | -0.88606 |
| 0.312 | 0.21 | -0.50585 | -0.67778 |
| 0.625 | 0.44 | -0.20412 | -0.35655 |
| 1.25 | 0.89 | 0.09691 | -0.05061 |
| 2.5 | 1.99 | 0.39794 | 0.298853 |
| 5 | 3.39 | 0.69897 | 0.5302 |

**Note:**

It is highly recommended to use the remaining reagents within 1 month provided this is prior to the expiration dateof the kit. For the expiration date of the kit, please refer to the label on the kit box. All components are stable up to the expiration date.

**[ SAMPLE COLLECTION AND STORAGE ]**

**Serum** - Use a serum separator tube and allow samples to clot for two hours at room temperature or overnight at4oC before centrifugation for 20 minutes at approximately 1,000×g. Assay freshlyprepared serum immediately or store samples in aliquot at -20oC or -80oC for later use. Avoid repeatedfreeze/thaw cycles.

**Plasma** - Collect plasma using EDTA or heparin as an anticoagulant. Centrifuge samples for 15 minutes at1,000×g at 2-8oC within 30 minutes of collection. Remove plasma and assay immediately or store samples in aliquot at -20oC or -80oC for later use. Avoid repeated freeze/thaw cycles.

**Tissue homogenates -** The preparation of tissue homogenates will vary depending upon tissue type.

1. Tissues were rinsed in ice-cold PBS to remove excess blood thoroughly and weighed before homogenization.

2. Minced the tissues to small pieces and homogenized them in fresh lysis buffer (catalog: IS007, different lysis buffer needs to be chosen based on subcellular location of the target protein) (w:v = 1:20-1:50, e.g. 1mL lysis buffer is added in 20-50mg tissue sample) with a glass homogenizer on ice (Micro Tissue Grinders woks, too).

3. The resulting suspension was sonicated with an ultrasonic cell disrupter till the solution is clarified.

4. Then, the homogenates were centrifuged for 5 minutes at 10,000×g. Collect the supernates and assay immediately or aliquot and store at ≤-20oC.

**Cell Lysates -** Cells need to be lysed before assaying according to the following directions.

1. Adherent cells should be washed by cold PBS gently, and then detached with trypsin, and collected by centrifugation at 1,000×g for 5 minutes (suspension cells can be collected by centrifugation directly).

2. Wash cells three times in cold PBS.

3. Resuspend cells in fresh lysis buffer with concentration of 107 cells/mL. If it is necessary, the cells could subjected to ultrasonication till the solution is clarified.

4. Centrifuge at 1,500×g for 10 minutes at 2-8oC to remove cellular debris. Assay immediately or aliquot and store at ≤-20oC.

**Cell culture supernates and other biological fluids -** Centrifuge samples for 20 minutes at 1,000×g. Collect the supernates and assay immediately or store samples in aliquot at -20oC or -80oC for later use. Avoid repeated freeze/thaw cycles.

**Note:**

1. Samples to be used within 5 days may be stored at 4oC, otherwise samples must be stored at -20oC (≤1 month) or -80oC (≤2 months) to avoid loss of bioactivity and contamination.

2. Sample hemolysis will influence the result, so hemolytic specimen should not be used.

3. When performing the assay, bring samples to room temperature.

4. It is highly recommended to use serum instead of plasma for the detection based on quantity of our in-house data.

**[ REAGENT PREPARATION ]**

1. Bring all kit components and samples to room temperature (18-25oC) before use. If the kit will not be used up in one time, please only take out strips and reagents for present experiment, and leave the remaining strips and reagents in required condition.

2. **Standard -** Reconstitute the **Standard** with 1.0mL of **Standard Diluent,** kept for 10 minutes at room temperature, shake gently(not to foam). The concentration of the standard in the stock solution is 4,000pg/mL. Please prepare 7 tubes containing 0.5mL Standard Diluent and produce a double dilution series according to the picture shown below. Mix each tube thoroughly before the next transfer. Set up 7 points of diluted standard such as 4,000pg/mL, 2,000pg/mL, 1,000pg/mL, 500pg/mL, 250pg/mL, 125pg/mL,

62.5pg/mL, and the last EP tubes with **Standard Diluent** is the blank as 0pg/mL.

Tube 1 2 3 4 5 6 7 8 pg/mL 4,000 2,000 1,000 500 250 125 62.5 0

3. **Detection Reagent A and Detection Reagent B -** Briefly spin or centrifuge the stock Detection A and Detection B before use. Dilute them to the working concentration 100-fold with **Assay Diluent A** and **B**, respectively.

4. **Wash Solution** - Dilute 20mL of Wash Solution concentrate (30×) with 580mL of deionized or distilled water to prepare 600mL of Wash Solution (1×).

5. **TMB substrate** - Aspirate the needed dosage of the solution with sterilized tips and do not dump the residual solution into the vial again.

**Note:**

1. Making serial dilution in the wells directly is not permitted.

2. Prepare standards within 15 minutes before assay. Please do not dissolve the reagents at 37oC directly.

3. Please carefully reconstitute Standards or working Detection Reagent A and B according to the instruction, and avoid foaming and mix gently until the crystals are completely dissolved. To minimize imprecision caused by pipetting, use small volumes and ensure that pipettors are calibrated. It is recommended to suck more than 10μL for one pipetting.

4. The reconstituted Standards, Detection Reagent A and Detection Reagent B can be **used only once**.

5. If crystals have formed in the Wash Solution concentrate (30×), warm to room temperature and mix gently until the crystals are completely dissolved.

6. Contaminated water or container for reagent preparation will influence the detection result.

**[ SAMPLE PREPARATION ]**

1. We are only responsible for the kit itself, but not for the samples consumed during the assay. The user should

calculate the possible amount of the samples used in the whole test. Please reserve sufficient samples in

advance.

2. Please predict the concentration before assaying. If values for these are not within the range of the standard

curve, users must determine the optimal sample dilutions for their particular experiments. Sample should be

diluted by PBS.

3. If the samples are not indicated in the manual, a preliminary experiment to determine the validity of the kit is

necessary.

4. Tissue or cell extraction samples prepared by chemical lysis buffer may cause unexpected ELISA results due

to the impacts from certain chemicals.

5. Due to the possibility of mismatching between antigen from other origin and antibody used in our kits (e.g.,

antibody targets conformational epitope rather than linear epitope), some native or recombinant proteins

from other manufacturers may not be recognized by our products.

6. Influenced by the factors including cell viability, cell number or sampling time, samples from cell culture

supernates may not be detected by the kit.

7. Fresh samples without long time storage is recommended for the test. Otherwise, protein degradation and

denaturalization may occur in those samples and finally lead to wrong results.

**[ ASSAY PROCEDURE ]**

1. Determine wells for diluted standard, blank and sample. Prepare 7 wells for standard, 1 well for blank. Add 100μL each of dilutions of standard (read Reagent Preparation), blank and samples into the appropriate wells. Cover with the Plate sealer. Incubate for 1 hour at 37oC.

2. Remove the liquid of each well, don’t wash.

3. Add 100μL of **Detection Reagent A** working solution to each well, cover the wells with the plate sealer and incubate for 1 hour at 37oC.

4. Aspirate the solution and wash with 350μL of 1× Wash Solution to each well using a squirt bottle, multi-channel pipette, manifold dispenser or autowasher, and let it sit for 1~2 minutes. Remove the remaining liquid from all wells completely by snapping the plate onto absorbent paper. Totally wash 3 times. After the last wash, remove any remaining Wash Buffer by aspirating or decanting. Invert the plate and blot it against absorbent paper.

5. Add 100μL of **Detection Reagent B** working solution to each well, cover the wells with the plate sealer and incubate for 30 minutes at 37oC.

6. Repeat the aspiration/wash process for total 5 times as conducted in step 4.

7. Add 90μL of **Substrate Solution** to each well. Cover with a new Plate sealer. Incubate for 10 - 20 minutes at 37oC (Don't exceed 30 minutes). Protect from light. The liquid will turn blue by the addition of Substrate Solution.

8. Add 50μL of **Stop Solution** to each well. The liquid will turn yellow by the addition of Stop solution. Mix the liquid by tapping the side of the plate. If color change does not appear uniform, gently tap the plate to ensure thorough mixing.

9. Remove any drop of water and fingerprint on the bottom of the plate and confirm there is no bubble on the surface of the liquid. Then, run the microplate reader and conduct measurement at 450 nm immediately.

**Note:**

1. **Assay preparation:** Keep appropriate numbers of wells for each experiment and remove extra wells from microplate. Rest wells should be resealed and stored at -20oC.

2. **Samples or reagents addition**：**Please use the freshly prepared Standard.** Please carefully add samples to wells and mix gently to avoid foaming. Do not touch the well wall. For each step in the procedure, total dispensing time for addition of reagents or samples to the assay plate should not exceed 10 minutes. This

will ensure equal elapsed time for each pipetting step, without interruption. Duplication of all standards and specimens, although not required, is recommended. To avoid cross-contamination, change pipette tips between additions of standards, samples, and reagents. Also, use separated reservoirs for each reagent.

3. **Incubation:** To ensure accurate results, proper adhesion of plate sealers during incubation steps is necessary. Do not allow wells to sit uncovered for extended periods between incubation steps. Once reagents are added to the well strips, DO NOT let the strips DRY at any time during the assay. Incubation time and temperature must be controlled.

4. **Washing:** The wash procedure is critical. Complete removal of liquid at each step is essential for good performance. After the last wash, remove any remaining Wash Solution by aspirating or decanting and remove any drop of water and fingerprint on the bottom of the plate. Insufficient washing will result in poor precision and false elevated absorbance reading.

5. **Controlling of reaction time:** Observe the change of color after adding **TMB Substrate** (e.g. observation once every 10 minutes), if the color is too deep, add **Stop Solution** in advance to avoid excessively strong reaction which will result in inaccurate absorbance reading.

6. **TMB Substrate** is easily contaminated. Please protect it from light.

7. The environment humidity which is less than 60% might have some effects on the final performance, therefore, a humidifier is recommended to be used at that condition.

**[ TEST PRINCIPLE ]**

The microplate provided in this kit has been pre-coated with an antibody specific to Bax. Standards or samples are then added to the appropriate microplate wells with a biotin-conjugated antibody specific to Bax. Next, Avidin conjugated to Horseradish Peroxidase (HRP) is added to each microplate well and incubated. After TMB substrate solution is added, only those wells that contain Bax, biotin-conjugated antibody and enzyme-conjugated Avidin will exhibit a change in color. The enzyme-substrate reaction is terminated by the addition of sulphuric acid solution and the color change is measured spectrophotometrically at a wavelength of 450nm ± 10nm. The

concentration of Bax in the samples is then determined by comparing the O.D. of the samples to the standard curve.

**[ CALCULATION OF RESULTS ]**

Average the duplicate readings for each standard, control, and samples and subtract the average zero standard optical density. Construct a standard curve by plotting the mean O.D. and concentration for each standard and draw a best fit curve through the points on the graph or create a standard curve on log-log graph paper with Bax concentration on the y-axis and absorbance on the x-axis. Using some plot software, for instance, curve expert

1.30, is also recommended. If samples have been diluted, the concentration read from the standard curve must be multiplied by the dilution factor.

**[ TYPICAL DATA ]**

In order to make the calculation easier, we plot the O.D. value of the standard (X-axis) against the known concentration of the standard (Y-axis), although concentration is the independent variable and O.D. value is the dependent variable. However, the O.D. values of the standard curve may vary according to the conditions of assay performance (e.g. operator, pipetting technique, washing technique or temperature effects), plotting log of the data to establish standard curve for each test is recommended. Typical standard curve below is provided for

Reference only.

**Typical Standard**

**[ DETECTION RANGE ]**

62.5-4,000pg/mL. The standard curve concentrations used for the ELISA’s were 4,000pg/mL, 2,000pg/mL, 1,000pg/mL, 500pg/mL, 250pg/mL, 125pg/mL, 62.5pg/mL.

**[ SENSITIVITY ]**

The minimum detectable dose of Bax is typically less than 28.2pg/mL.

The sensitivity of this assay, or Lower Limit of Detection (LLD) was defined as the lowest protein concentration that could be differentiated from zero. It was determined by adding two standard deviations to the mean optical density value of twenty zero standard replicates and calculating the corresponding concentration.

**[ SPECIFICITY ]**

This assay has high sensitivity and excellent specificity for detection of Bax.

No significant cross-reactivity or interference between Bax and analogues was observed.

**Note:**

Limited by current skills and knowledge, it is impossible for us to complete the cross- reactivity detection between Bax and all the analogues, therefore, cross reaction may still exist.

**[ RECOVERY ]**

Matrices listed below were spiked with certain level of recombinant Bax and the recovery rates were calculated by comparing the measured value to the expected amount of Bax in samples.

Matrix Recovery range (%) Average(%) serum(n=5) 90-99 95 EDTA plasma(n=5) 80-96 90 heparin plasma(n=5) 92-105 101

**[ LINEARITY ]**

The linearity of the kit was assayed by testing samples spiked with appropriate concentration of Bax and their serial dilutions. The results were demonstrated by the percentage of calculated concentration to the expected. Sample 1：2 1：4 1：8 1：16

serum(n=5) 90-99% 93-101% 89-105% 85-93%

EDTA plasma(n=5) 92-106% 81-90% 84-98% 92-102%

heparin plasma(n=5) 78-97% 84-104% 90-102% 87-97%

**[ PRECISION ]**

Intra-assay Precision (Precision within an assay): 3 samples with low, middle and high level Bax were tested 20 times on one plate, respectively.

Inter-assay Precision (Precision between assays): 3 samples with low, middle and high level Bax were tested on 3 different plates, 8 replicates in each plate.

CV(%) = SD/meanX100

Intra-Assay: CV<10%

Inter-Assay: CV<12%

**[ STABILITY ]**

The stability of ELISA kit is determined by the loss rate of activity. The loss rate of this kit is less than 5% prior to the expiration date under appropriate storage condition. To minimize extra influence on the performance, operation procedures and lab conditions, especially room temperature, air humidity, incubator temperature should be strictly monitored. It is also strongly suggested that the assay is performed by the same operator from the beginning to the end.

**[ SAMPLE VALUE ]**

**Serum/Plasma** - Twenty-four serum and plasma samples from apparently healthy volunteers were evaluated in this assay. All samples measured less than the lowest Bax standard.

**Cell Lysates** - Cell lysates prepared according to protocol shown in [ SAMPLE COLLECTION AND STORAGE ]

section on page 2 were assayed for Bax levels. Results are shown in the table below.

Samples O.D. Range

A549 cells 0.51-0.87

**These data are our in-house data, only for reference.**

**[ ASSAY PROCEDURE SUMMARY ]**

1. Prepare all reagents, samples and standards;

2. Add 100μL standard or sample to each well. Incubate 1 hour at 37oC;

3. Aspirate and add 100μL prepared Detection Reagent A. Incubate 1 hour at 37oC;

4. Aspirate and wash 3 times;

5. Add 100μL prepared Detection Reagent B. Incubate 30 minutes at 37oC;

6. Aspirate and wash 5 times;

7. Add 90μL Substrate Solution. Incubate 10-20 minutes at 37oC;

8. Add 50μL Stop Solution. Read at 450nm immediately.

**[ IMPORTANT NOTE ]**

1. Limited by the current conditions and scientific technology, we can't completely conduct the comprehensive identification and analysis on the raw material provided by suppliers. So there might be some qualitative and technical risks to use the kit.

2. The final experimental results will be closely related to validity of the products, so the kit should be used prior to the expiration date. And please store the kits exactly according to the instruction.

3. Kits from different batches may be a little different in detection range, sensitivity and color developing time. Please perform the experiment exactly according to the instruction attached in kit while electronic ones from our website is only for reference.

4. Do not mix or substitute reagents from one kit lot to another. Use only the reagents supplied by manufacturer.

5. Protect all reagents from strong light during storage and incubation. All the bottle caps of reagents should be covered tightly to prevent the evaporation and contamination of microorganism. TMB Substrate should remain colorless till it is reacted with the enzyme which binds to the microplate.

6. There may be some foggy substance in the wells when the plate is opened at the first time. It will not have any effect on the final assay results. Do not remove microplate from the storage bag until needed.

7. Wrong operations during the reagents preparation and loading, as well as incorrect parameter setting for the plate reader may lead to incorrect results. A microplate reader with a bandwidth of 10nm or less and anoptical density range of 0-3 O.D. at 450 ± 10nm wavelength is acceptable for use in absorbance pread the instruction carefully and adjust the instrument prior to the experiment.

8. Variation in sample preparation and each step of experimental operation may cause different results. In order to get better reproducible results, the operation of each step in the assay should be controlled.

9. Each kit has been strictly passed Q.C test. However, results from end users might be inconsistent with our in-house data due to some unexpected transportation conditions or different lab equipments. Intra-assay variance among kits from different batches might arise from above factors, too.

10. Kits from different manufacturers with the same item might produce different results, since we haven’t compared our products with other manufacturers.

11. The standard of the kit and immunogen used for antibody preparation are commonly recombinant proteins, as different fragments, expression systems, purification methods might be used in recombinant protein preparation, we can not guarantee the kit could detect recombinant protein from other companies. So, it is not recommended to use the kit for the detection of recombinant protein.

12. Please predict the concentration of target molecules in samples, or arrange a preliminary experiment, it is a good way to solve specific problem, e.g. the concentration of samples are beyond the detection range of the kit.

13. The kit might not be suitable for detection of samples from some special experiment, for instance, knock-out, due to their uncertainty of effectiveness.

14. The instruction manual is also for the kit of 48T, but all reagents of 48T kit are reduced by half.

15. The kit is designed for research use only, we will not be responsible for any issue if the kit was used in clinical diagnostic or any other procedures.

**[ PRECAUTION ]**

The Stop Solution suggested for use with this kit is an acid solution. Wear eye, hand, face, and clothing protection when using this material.

and Add adequate reagents

Incorrect incubation times Ensure sufficient incubation times

Incorrect incubation temperature Reagents balanced to room temperature

Conjugate or substrate reagent failure Mix conjugate & substrate, color should develop immediately

No stop solution added Follow the assay protocol in the kit manual

Read beyond suggested reading time Read within the time recommended in the manual

**Lab Report**

*** Cytotoxicity results**

| **ser** | **Sample** | | **Cytotoxicity**  **IC50**  **uM/ml** | | | SD |
| --- | --- | --- | --- | --- | --- | --- |
|  | **code** | **MW**  **g/mol** | **mcf7** | **hepg2** | **Hela** |  |
| 1 | **Erlotinib** | **---** | **4.277**±0.26 | **11.314**±0.66 | **8.27**±0.42 |  |

| 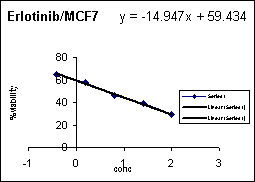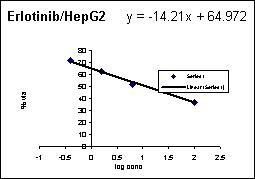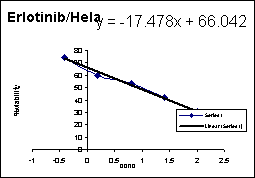 |  |  |  |
| --- | --- | --- | --- |
|  |  |  |  |
|  |  |  |  |
|  |  |  |  |
|  |  |  |  |
|  |  |  |  |
|  |  |  |  |
|  |  |  |  |
|  |  |  |  |
|  |  |  |  |
|  |  |  |  |
|  |  |  |  |
|  |  |  |  |
|  |  |  |  |

Detailed results:

|  | researcher | |  | assay |  |  | Date |
| --- | --- | --- | --- | --- | --- | --- | --- |
|  | Dr.Nadia Hanafi | |  | MTT |  |  | 16-Jan |
|  |  |  |  |  |  |  |  |
|  | **Blank** | **CC** | **Sample No. Erlotinib/MCF7** | | | | |
|  | **1** | **2** | **3** | **4** | **5** | **6** | **7** |
| A | B | C | 100uM | 25uM | 6.3uM | 1.6uM | 0.4uM |
| B | B | C | 100uM | 25uM | 6.3uM | 1.6uM | 0.4uM |
| C | B | C | 100uM | 25uM | 6.3uM | 1.6uM | 0.4uM |
| ROBONIK P2000 Eia reader  Wave length: 450 nm  Reference: 630 nm | | | | | | | |
|  | **1** | **2** | **3** | **4** | **5** | **6** | **7** |
|  |  |  |  |  |  |  |  |
| A | 0.001 | 0.592 | 0.177 | 0.231 | 0.279 | 0.334 | 0.387 |
| B | 0.001 | 0.579 | 0.184 | 0.227 | 0.277 | 0.352 | 0.391 |
| C | 0.001 | 0.603 | 0.165 | 0.231 | 0.261 | 0.346 | 0.375 |
| mean | 0.0004 | 0.5913 | 0.1753 | 0.23 | 0.2723 | 0.344 | 0.3843 |
| % |  | ` | 29.651 | 38.84 | 46.054 | 58.174 | 64.994 |
| Erlotinib/MCF7 | | 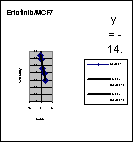 | | | | | |
| log conc. | % viability |  |  |  |  |  |  |
| 2 | 29.651 |  |  |  |  |  |  |
| 1.3979 | 38.839 |  |  |  |  |  |  |
| 0.7959 | 46.054 |  |  |  |  |  |  |
| 0.1931 | 58.174 |  |  |  |  |  |  |
| -0.4089 | 64.994 |  |  |  |  |  |  |
|  |  |  |  |  |  |  |  |
| IC50= |  |  |  |  |  |  |  |
|  |  |  |  |  |  |  |  |
|  |  |  |  |  |  |  |  |
|  | **Blank** | **CC** | **Sample No. Erlotinib/HepG2** | | | | |
|  | **1** | **2** | **3** | **4** | **5** | **6** | **7** |
| A | B | C | 100uM | 25uM | 6.3uM | 1.6uM | 0.4uM |
| B | B | C | 100uM | 25uM | 6.3uM | 1.6uM | 0.4uM |
| C | B | C | 100uM | 25uM | 6.3uM | 1.6uM | 0.4uM |
| ROBONIK P2000 Eia reader  Wave length: 450 nm  Reference: 630 nm | | | | | | | |
|  | **1** | **2** | **3** | **4** | **5** | **6** | **7** |
|  |  |  |  |  |  |  |  |
| A | 0.001 | 0.577 | 0.207 | 0.272 | 0.314 | 0.377 | 0.422 |
| B | 0.001 | 0.583 | 0.215 | 0.266 | 0.292 | 0.361 | 0.413 |
| C | 0.001 | 0.602 | 0.222 | 0.281 | 0.304 | 0.357 | 0.425 |
| mean | 0.001 | 0.5873 | 0.2147 | 0.273 | 0.3033 | 0.365 | 0.42 |
| % viability |  |  | 36.549 | 46.48 | 51.646 | 62.145 | 71.51 |
| Erlotinib/HepG2 | | 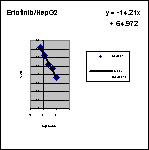 | | | | | |
| log conc. | % viability |  |  |  |  |  |  |
| 2 | 36.549 |  |  |  |  |  |  |
| 1.3979 | 46.481 |  |  |  |  |  |  |
| 0.7959 | 51.646 |  |  |  |  |  |  |
| 0.1931 | 62.145 |  |  |  |  |  |  |
| -0.4089 | 71.51 |  |  |  |  |  |  |
|  |  |  |  |  |  |  |  |
| IC50= |  |  |  |  |  |  |  |
|  |  |  |  |  |  |  |  |
|  |  |  |  |  |  |  |  |
|  | **Blank** | **CC** | **Sample No. Erlotinib/Hela** | | | | |
|  | **1** | **2** | **3** | **4** | **5** | **6** | **7** |
| A | B | C | 100ug | 25ug | 6.3ug | 1.6ug | 0.4ug |
| B | B | C | 100ug | 25ug | 6.3ug | 1.6ug | 0.4ug |
| C | B | C | 100ug | 25ug | 6.3ug | 1.6ug | 0.4ug |
| ROBONIK P2000 Eia reader  Wave length: 450 nm  Reference: 630 nm | | | | | | | |
|  | **1** | **2** | **3** | **4** | **5** | **6** | **7** |
|  |  |  |  |  |  |  |  |
| A | 0.001 | 0.558 | 0.188 | 0.234 | 0.287 | 0.331 | 0.422 |
| B | 0.003 | 0.542 | 0.167 | 0.229 | 0.291 | 0.329 | 0.407 |
| C | 0.001 | 0.571 | 0.162 | 0.235 | 0.316 | 0.343 | 0.415 |
| mean | 0.0017 | 0.557 | 0.1723 | 0.233 | 0.298 | 0.3343 | 0.4147 |
| % viability |  |  | 30.94 | 41.77 | 53.501 | 60.024 | 74.446 |
| Erlotinib/Hela | | 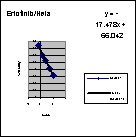 | | | | | |
| 2 | 30.94 |  |  |  |  |  |  |
| 1.3979 | 41.771 |  |  |  |  |  |  |
| 0.7959 | 53.501 |  |  |  |  |  |  |
| 0.1931 | 60.024 |  |  |  |  |  |  |
| -0.4089 | 74.446 |  |  |  |  |  |  |
|  |  |  |  |  |  |  |  |
| IC50= |  |  |  |  |  |  |  |
|  |  |  |  |  |  |  |  |
